# Supplementary material for: Beyond the words: Exploring individual differences in the evaluation of poetic creativity
Source: PLoS One. 2024 Oct 3;19(10):e0307298. doi: 10.1371/journal.pone.0307298 (PMC11449365; doi:10.1371/journal.pone.0307298)
Supplement: Attachment — Submitted filename: Response to Reviewers.pdf [file pone.0307298.s002.pdf]

Our Responses to the Editor and the Reviewer(s)

Thank you very much for giving us an opportunity to revise the manuscript. Below are our responses to each comment of the Academic editor and the Reviewer. The Editor's and Reviewers' comments are shown in red, our responses are in regular font, and the inclusions are in **bold green.**

Our responses to the Editor's comments

1. Lines 28-29: "...of a broad range of English language poems ... rated each poem..." You have not stated yet how many poems were used. Instead of 'broad range', state the number

Our response: Thank you for your suggestion. The number of poems has now been mentioned in the manuscript, as follows: "This study investigated how personality traits, specifically openness, intellect, awe-proneness, and epistemic curiosity, shape the implicit associations between subjective experiences and the judgment of creativity of **thirty-six** English language poems."

2. Line 106: "meter (36, 40), , metaphors (35), (41,42,43), and" either there is bad formatting, or something is missing on this line.

Our response: Thank you. Formatting has been revised.

3. Line 117: "we considered both dimensions of emotion – valence and arousal." Both 'valence' and 'arousal' are technical term in psychology and related fields. Please be kind enough to explain those terms to the general audience (a footnote may suffice). Moreover, since 'felt valence' was found to be important in your work, explain to readers what it is.

Our response: Thank you for the suggestion. We explained the terms in the revised manuscript as suggested. The lines read as follows:

"According to the two-dimensional circumplex model of emotion, emotional states are represented along two orthogonal dimensions: valence (pleasure-displeasure: horizontal axis) and arousal (degree of arousal: vertical axis) (Russell, 1980). Poetry evokes strong emotions (Wassiliwizky et al., 2017) and emotional states can influence creativity evaluation (Mastria et al., 2019)…. Of note, perceived and felt emotions may be distinct (Gabrielsson, 2001; Marin & Bhattacharya, 2010). Perceived emotions are the emotions evoked by the stimuli whereas felt emotions are the emotions felt by the perceivers. In our study, we focused on the felt emotions, i.e., the emotions felt by the reader while reading the poem, rather than the perceived emotion, i.e., the emotion recognized in the poem. Therefore, felt valence reveals the extent to which the readers felt positive or negative emotions while reading the poems, whereas felt arousal here reveals how intense it was felt by the readers."

4. Lines 122-125: “Surprise is considered as a stronger predictor of creativity than value after...” The description of 'Surprise' dimension is very dry and technical (unlike the other descriptions), and it does not explain what is 'Surprise' in this context. Also what is 'value' in the above sentence? Did you mean valence?

Our response: Thank you very much for the comment. We have now expanded our explanation to clarify the specific meaning of “surprise’ within the context of our study. The section reads as: **“Surprise is usually a short-lived emotion elicited by events that deviate from an established schema (Meyer et al., 1997; Meyer et al., 1991; Noordewier & Breugelmans, 2013). Of note, a schema, in this sense, refers to a component of the organism’s knowledge structure, activated by a specific stimulus (Rumelhart, 1984). Surprise is recognized as a key predictor of creativity (Simonton, 2012; Acar et al., 2017) and is also a robust predictor of aesthetic judgment of artwork (Pietras & Ganczarek, 2022). In the context of our study, the term “surprise” specifically denotes the degree to which there is a sudden and unexpected change in the context or theme of the poem.”**

Response to your next question on “value”: Despite the lack of an agreed-upon definition, a general consensus over a standard definition of creativity (Runco & Jaeger, 2012; see also Stein, 1953; Barron, 1955) involves two factors: The first factor consists of characteristics such as novelty, originality, infrequency, or unusualness; and the second factor is related to usefulness, value, utility, effectiveness, adaptability, or appropriateness (Acar et al., 2017). **However, we excluded the line containing “value” to avoid confusion of readers as we did not explore any potential consequences of this term later in the article.**

Line 127: “It is a broad range of traits...” What is “It”? Why do you begin with “It”? Please formulate the description clearly.

Our response: The line has been amended as: **Openness to experience** is a broad range of traits, from intellectual abilities to aesthetic and artistic interests (Oleynick et al., 2017;DeYoung et al., 2012; Chamorro-Premuzic et al., 2009) and is most robustly associated with measures of creativity (McCrae, 1996).

Lines 130-132: “Openness/intellect … can be differentiated into two major aspects (64): openness and intellect.” Really? Isn't it a bit tautological? Please clarify – is it one predictor or two? If those are two predictors, why are they bundled together?

Our response: Actually, Costa Jr & McCrae, (1992) first introduced Openness to Experience as the fifth factor of personality traits. Later, DeYoung et al created the Openness/Intellect (O/I) model, which proposed that Openness to Experience has two major facets– the two “related but separable trait dimensions” (DeYoung et al., 2007, p.883): openness and intellect. Based on earlier work on the neurocognitive correlates of Openness to Experience (DeYoung et al., 2005), DeYoung et al., 2009 hypothesized that brain regions associated with working memory would predict the intellect

facets but not the openness facets. Showing distinct effects of openness and intellect would provide evidence for the validity of both constructs (see Nusbaum & Silvia, 2011).

Hence, in our study, we are interested to explore the distinct influence of openness and intellect on the evaluation of creativity of poems.

5. Lines 152-153: "Earlier research has shown that openness influences attitudes toward targets that violate established schemas (68)." This is absolutely unclear, needs some elaboration. What are 'targets'? Targets of what? Which established schemas? OF behavior? Of Social norms? Of poems? Of poetic structure? What is meant by 'violate' here?

Our response: We appreciate your comment. We revised the lines as: **"As surprise is often experienced when individuals encounter unexpected or schema-discrepant events (Meyer et al., 1997; Meyer et al., 1991), we assumed that the assessment of expectancy violations in the judgment of poetic creativity necessitates a substantial degree of cognitive engagement. Consequently, we predicted that both Openness and Intellect would moderate the relationship between surprise and creativity. "**

6. Line 168: "aesthetic chill" Please explain what is aesthetic chill.

Our response: We explained the term. The line now reads: **"Aesthetic chills are transient emotional responses (McCrae, 2007) to aesthetical stimuli. They can be described as experiencing a chill or wave of excitement when engaging with poetry or looking at a work of art (Costa & McCrae, 2008)."**

7. Line 196: Please explain what is "Prolific®"

Our response: Explained as suggested: **"The experiment was created using Qualtrics®, and the link was disseminated through Prolific®, a platform for participant recruitment."**

8. Section "Materials and methods" This section starts with 'Participants' section and it's quite awkward. The 'Participants' section should be moved after the 'Materials' section.

Our response: Thank you. "Participants" section is now shifted after the "Materials" section, as suggested.

9. Lines 194-196: The 'Participants' section begins with a description of minimum sample size. This section needs to be clarified. What led you to focus on "80% power for a medium effect (^2 = 0.15) with squared multiple correlation ^2 = 0.13." It seems rather arbitrary.

Our response: The section has been clarified as suggested. The section now reads as:

“By using the G*Power software (v. 3.1.9.4), (Faul et al., 2007) we found that a minimum sample size of 98 was required to detect a medium effect size (f^2 = 0.15) in a multiple linear regression, assuming a significance level of 0.05 and a statistical power of 80%. The criteria we used are widely-used conventional figures when estimating sample sizes. Additionally, we used the ‘samplesize_mixed’ function in R, which estimates the required sample size for linear mixed models (two-level-designs), based on power-calculation for standard design and adjusted for design effect for 2-level-designs (https://strengejacke.github.io/sjstats/). For our multilevel model, which considered 96 cluster groups and a small to medium effect size (Cohen’s d = 0.3), with 36 observations per cluster group (level-2-unit), we determined that a total of 965 observations would be necessary. This translates to a minimum of 27 participants (965/36).”

10. Also, you did not specify where the participants came from, in terms of education, profession, English language proficiency (native/non-native) and age groups. Would it be possible to have some indications about those background variables?

Our response: As per the self-reported demographic questionnaires, all 96 participants (32 males, 63 females, 1 preferred not to say; mean age = 31.94 years, SD = 13.09) were fluent in English and came from a variety of educational backgrounds, with each holding at least a bachelor’s degree in any discipline. Additionally, our sample represented a diverse range of professions and ethnicities.

We included this information in the “Participants” section.

11. Line 196: “healthy participants” What does 'healthy' mean here? Maybe specify in a footnote. What criteria were used? Is this aspect really important?

Our response: Thank you for your comment. Considering that the “health” aspect was not a significant focus of our study, we omitted the term “healthy.”

12. Line 202: “We described the main experimental procedures for this research to the participants” It is not clear what was explained. Maybe add an appendix? How did you explain 'valence' and 'arousal' to the participants?

Our response: The instructions to the participants are now included in the main manuscript. The full paragraph now reads:

“Participants were briefed about the experimental procedure, which involved the assessment of a set of English poems on a 7-point Likert rating scale (1=extremely low; 7=extremely high) across various constructs, including clarity, aesthetic appeal, felt valence, arousal, surprise, and overall creativity. Additionally, participants were instructed to complete demographic and personality-related questions. We assured participants of the full confidentiality of their data, in compliance with the General Data Protection Regulation, and clarified that any published results would be non-identifiable. All participants provided informed consent (online) before data collection. Participants received a cash incentive of £7.50 per hour. The data collection period spanned from 27 January 2022 to 23 June 2022, and the data were accessed for research purposes only after this period.”

How did you explain 'valence' and 'arousal' to the participants?

For felt valence, participants were asked: “How positive (higher Scores) or negative (lower Scores) did you feel when you read the poem?” For arousal, they were asked: “How stimulating (higher Scores) or relaxing (lower Scores) did you feel when you read the poem?”

13. Section 'Materials': The procedure of poem selection is not described well. Selection sources (lines 224-227) should be described before the lexical metrics of selected poems (lines 213-223).

Our response: As suggested, the sources for poem selection have been described in the “Material” section:

“Initially, we selected 108 poems from various online resources, including Poetry.org (http://www.poetry.org/), the Poetry Foundation (https://www.poetryfoundation.org/), and the Academy of American Poets (https://poets.org/). These poems were subsequently rated for "surprise" on a scale of 1 to 7 by M.D, one of the co-authors who is a Professor of Creative Writing with domain-specific expertise and an award-winning poet. Following this evaluation, we shortlisted 36 as the experimental stimuli: 18 with low surprise ratings (4 or lower) and 18 with high surprise ratings (6 or above). The chosen poems varied in structure, contents, lines, and word count (mean number of lines = 11, SD = 3.2; mean word count = 71.3, SD = 29). To represent a broad spectrum of English poems, we consciously chose not to limit our selection to a particular genre or form, like haiku or sonnets as done in previous studies (Belfi et al., 2018**;** Hitsuwari & Nomura, 2022**;** (Papp-Zipernovszky et al., 2021)**.**

13. How did you converge on the genres and themes that are reported in Table 1? Who provided the labels of genres and themes?

M.D, a co-author of our study who is a Professor of Creative Writing with domain-specific expertise and an award-winning poet, validated the genre and theme-specific information about the stimuli.

14. Lines 218-221. Concerning “lexical diversity (LD)”. Explain to readers what is LD, and why it is relevant for this study. There are many metrics for LD. Please describe which one you used. The legend of table 1 says “(LD = number of unique words/ total characters).” That should be explained in the text. Also, that is not a common metric of LD. Where did you adopt it from? “The average (SD) LD score for our stimuli set was 14.30 (1.7).” Switch “(SD) LD” to “LD (DS)”. Also explain to readers: is 14.30 high or low? What is the scale and what does the value mean?

Our response: Thanks for this valuable comment. While lexical diversity and semantic diversity may not be directly pertinent to our study, we decided to include them to capture the variedness and richness of vocabulary and semantics in our chosen stimuli. Of note, we rectified the lexical diversity scores (computed in R). Table1, originally presenting stimulus details, has been modified to incorporate updated Lexical Diversity and is now designated as Table S1. We intend to include it in the Supplementary section.The paragraph describing the diversity metrics has been modified as follows:

“Lexical diversity (LD) of a text refers to its lexical richness, indicating range and variety of vocabulary deployed in a text (McCarthy & Jarvis, 2007). For computing the LD of the poems, we

used the type-token ratio (TTR) method, which calculates the ratio of unique words (types) to the total word count (tokens) (Chotlos, 1944). It ranges from 0 to 1: a higher TTR indicates a greater lexical diversity. The average lexical diversity across the poems is 0.77 with a standard deviation of 0.09, suggesting that, on average, about 77% of the words used in the poems are unique or different. Semantic diversity, on the other hand, refers to the range of contexts (i.e., semantic richness) in which words are used (Johnson et al., 2022). To calculate the semantic diversity, we used divergent semantic integration (DSI) (http://semdis.wlu.psu.edu/), representing the mean semantic distance between all word pairs in a text; DSI varies from 0 to 1, where a higher score indicates that a broader collection of divergent ideas. The average DSI of our stimuli is 0.80 with a standard deviation of 0.03, indicating a high degree of semantic variety (see Table S1 in the Supplementary section for details).”

In the “Limitations” section, we added the limitations of TTR method for short texts as follows: **“it is important to acknowledge that given the small word count of some of our poems and the implied limited vocabulary, the Type-Token Ratio (TTR) method might not yield reliable results due to constrained variability in word usage within small texts (Malvern & Richards, 1997; McCarthy & Jarvis, 2010).”**

The table (**Table S1 in the Supplementary section**) containing the details of the stimuli will be submitted in the Supplementary section and OSF (https://osf.io/9mw7r/?view_only=07137f4871d146c790501f22bc7743d5).

TableS1. The details of the stimuli

Poem Title	Poet	Theme	Genre	Word Count	No. of lines	Lexical Diversity(TTR)	DSI_mean
Funeral Blues	W. H. Auden	Grief	Modernist	136	16	0.742647059	0.80700305
At the Same Time	W.S. Merwin	Transience of existence	Contemporary	39	10	0.794871795	0.81102673
The Supreme Moment	Charles Simic	Powerlessness of humans	Contemporary	63	16	0.888888889	0.83547634
Peace	Langston Hughes	Futility of war	War poetry	26	8	0.884615385	0.77777005
A Peck of Gold	Robert Frost	Illusion	Modernist	86	12	0.569767442	0.76179961
The Freedom of the Moon	Robert Frost	Awe inspiring nature	Contemporary	91	12	0.769230769	0.80886736
Stopping by Woods on a Snowy Ev	Robert Frost	Contemplation	Modernist	108	16	0.694444444	0.78485471
When You Come	Maya Angelou	Nostalgia	Contemporary	41	10	0.829268293	0.82847741
Sonnet 116	William Shakespear	Eternity of love	Romantic	109	14	0.752293578	0.80245092
Apparently with no Surprise	Emily Dickinson	Transience of life	Nature poetry	36	8	0.916666667	0.82995713
Will there really be a "Morning"?	Emily Dickinson	Wonder and curiosity	Lyric	76	12	0.710526316	0.77878051
Unable are the Loved to die	Emily Dickinson	Immortality of love	Lyric	26	6	0.8	0.79995234
How happy is the little Stone	Emily Dickinson	Independence and contentment	Nature	46	10	0.869565217	0.83859107
Ah! Sun-flower	William Blake	Individualism	Romantic	50	8	0.84	0.81487795
The Smile	William Blake	Love and deceit	Romantic	106	16	0.528301887	0.74556787
Love after Love	Derek Walcott	Self-love, self-discovery	Contemporary	98	15	0.673469388	0.78304363
The Last Laugh	Wilfred Owen	Brutality of war	War poem	90	15	0.833333333	0.84464281
Clown in the Moon	Dylan Thomas	Melancholy	Lyric	48	8	0.770833333	0.77714294
Remember	Christina Rossetti	Love, death, remembrance	Lyric	111	14	0.630630631	0.73742609
When You Are Old	W. B. Yeats	True love	Romantic	100	12	0.7	0.78672981
Aedh wishes for the Cloths of Hea	W. B. Yeats	Love and dreams	Romantic	60	9	0.7	0.78105161
Memory	W.B. Yeats	Transience of youth and beauty	Lyric	33	6	0.818181818	0.79140706
The Arrow and the Song	Henry Wadsworth L	Power of words and friendship	Lyric	88	12	0.613636364	0.75714821
There Will Come Soft Rains	Sara Teasdale	War	Lyric	91	12	0.747252747	0.81156949
Alone	Sara Teasdale	Solitude	Romantic	80	12	0.7	0.77622178
Ozymandias	P. B. Shelley	Transience of power	Romantic	111	14	0.787610619	0.8292663
Good-Night	P. B. Shelley	Longing for togetherness	Romantic	75	12	0.805194805	0.77736462
A Lament	P. B. Shelley	Nostalgia, sadness	Lyric	65	10	0.815384615	0.80580131
Alas! This Is Not What I Thought L	P. B. Shelley	Challenges of life	Lyric	75	9	0.815789474	0.79610722
Fragment: A Wanderer	P. B. Shelley	Imagination	Romantic	25	4	0.88	0.83606831
Fragment: Apostrophe to Silence	P. B. Shelley	Existential longing	Romantic	69	9	0.819444444	0.8170317
MEDITATION XVII	John Donne	Interconnectedness of humanity	Romantic	81	13	0.728395062	0.77863012
Nothing Gold Can Stay	Robert Frost	Impermanence of beauty	Nature	40	8	0.85	0.81230023
My Heart Leaps Up	William Wordswort	Ecstasy with nature’s beauty	Nature	61	9	0.704918033	0.75068272
I taste a liquor never brewed –	Emily Dickinson	Joy of Nature	Lyric	81	16	0.80952381	0.83325713
If You Should Go	Countee Cullen	Transience of love and life	Romantic	44	8	0.818181818	0.76151851

15. Lines 222-224: Explain to readers what is DSI, and why it is relevant for this study. What is the scale and where on the scale is your value (0.8) located?

Our response: We explained DSI in the "Materials" section as follows:

"Semantic diversity, on the other hand, refers to the range of contexts (i.e., semantic richness) in which words are used (Johnson et al., 2022). To calculate the semantic diversity, we used divergent semantic integration (DSI) (http://semdis.wlu.psu.edu/), representing the mean semantic distance between all word pairs in a text; DSI varies from 0 to 1, where a higher score indicates that a broader collection of divergent ideas. The average DSI of our stimuli is 0.80 with a standard deviation of 0.03, indicating a high degree of semantic variety (see Table S1 in the Supplementary section for details)."

16. Lines 241-242: "Participants read a poem for 30 seconds" Explain why such a time limit was set? Was the poem still visible during creativity rating?

Our response: Given the concise nature of the poems (average number of lines = 11), we opted for a minimum reading time of 30 seconds. Participants were given a minimum of 30 seconds to read each poem. Following this period, they were allowed to proceed to the rating task. They were asked to rate the poems on six dimensions in the following order: clarity, aesthetic appeal, felt valence, felt arousal, surprise, and creativity, using a 7-point Likert scale (1: "Extremely Low" and 7: "Extremely High"). There was no time limit imposed for rating the poems. Of note, the poems remained visible during the rating process. This information has been included in the "Procedure" section.

17. Regarding the questions/measures on four personality traits: as those were shown to be correlated with creativity (Table 3). Please briefly explain the scales and value ranges for the personality traits measures. Moreover, please describe the distributions of values for all personality measures in your study. For example, As described in Table 2, it seems the four personality traits are also on 7-point scale. Can you establish that you had sufficient variability on the personality scores? It is not clear whether you participants represent sufficient difference on an Openness scale (and other scales). The SD for Openness is just 0.74 – are all participants approximately equally 'open'?. Do you have sufficient distribution for levels of intellect? Sufficient in terms of representing the general public?

Our response:

Of note, there was a typo in our curiosity rating data. We have rectified this in the revised manuscript. Therefore, the curiosity data in the Tables had undergone some minor changes, but without any significant impact on the findings and their implications.

scales for the personality traits measures: All scales are 1-7

value ranges:

Openness: min = 3.0; max = 6.4; mean = 5.02; SD = 0.74

Intellect: min = 2.7; max = 6.4; mean = 4.7; SD = 0.9

Awe-proneness: min = 1.83; max = 7; mean = 5.11; SD = 1.14

Curiosity: min = 3.5; max = 7; mean = 5.58; SD = 0.86

Variability in openness scores: 0.55 (moderate, suggesting a reasonable spread of scores among participants.)

Variability in intellect scores: 0.81 (high, indicating a broader range of scores among participants.)

Variability in awe-proneness scores: 1.31 (high, suggesting a diverse range of responses among participants.)

Variability in curiosity scores: 0.76 (moderate, indicating a reasonable distribution of scores.)

Therefore, personality measures capture overall diverse range which indicates that participants do not uniformly represent specific traits.

18. Variable names for personality traits are different in Table 2 and 3. Please adjust.

Our response: Thanks for pointing this. The Tables have been rectified accordingly.

19. Table 5 includes a variable "Expertise" but that variable was not described in the texts. Please describe appropriately.

Our response: We apologize for this inadvertent inclusion. The variable "Expertise" has been excluded from Table 5.

20. Lines 431-433: "These results suggest that individuals with higher levels of imagination, a greater appreciation for aesthetic experiences, and a strong inclination towards art demonstrated..." This claim is not quite warranted. Did you measure imagination? "appreciation for aesthetic experiences" ? Did you measure inclination towards art? It seems you did not measure those, so how can you make any claims about those?

Our response: Thanks for your valuable comment. Openness, as operationalised by the NEO Personality Inventory-Revised (Costa & McCrae, 2008), is characterized by a range of attributes: a vivid imagination (O1: Fantasy), an appreciation of art and beauty (O2: Aesthetics), depth of emotions (O3: Feelings), an eagerness to try new things (O4: Actions), intellectual curiosity (O5: Ideas), and being liberal (O6: Values). Previous research has shown that openness involves a deep engagement with perceptual and aesthetic domains (DeYoung et al., 2014). Further, it is the best predictor of positive attitudes towards aesthetics and active participation in aesthetic activities, such as visiting museums, reading literature, and creating artwork (Meyer et al., 2023; McCrae & Costa, 1997; Chamorro-Premuzic & Furnham, 2004; Kuiken et al., 2021). Notably, greater involvement in aesthetic activities is often related to higher scores in the openness trait (McManus & Furnham, 2006).

Our findings reveal that individuals with higher levels of openness place more emphasis on aesthetic appeal when assessing the creativity of poems, in contrast to those with lower levels of openness.

21. Line 465-466; "Our study suggests that as the level of ambiguity increased with the rise in the aesthetic appeal of a poem,..." You did not measure levels of ambiguity. So why do you make such unwarranted claims?

Our response: We appreciate your comment. We removed the line: "In addition….appreciation of poems" from the Discussion section.

22. Lines 485-486: "may have been more fostered by the intrinsic drive of highly curious readers to explore the unknown" To 'explore the unknown' by reading a short poem in 30 seconds? Are you serious?

Our response: Thank you for your comment. However, we would like to clarify that the 30-second timeframe set in our study is the minimum, rather than the maximum, duration allotted for reading each poem. This approach ensures that participants must have spent at least 30 sec or more to engage with the poem. During the evaluation phase, there was no imposed time limit, and the poem remained visible, facilitating a more thorough assessment and appreciation of the poem's nuances without time pressure. Hence, this extended interaction provides an opportunity for the participants to delve into aspects of the unfamiliar depicted in the poem, potentially stimulating their natural curiosity and desire for knowledge.

Furthermore, our study focuses on epistemic curiosity, a personality trait, characterized by a desire for new information that motivates exploratory behaviour and knowledge acquisition (Berlyne, 1954; Loewenstein, 1994; Litman & Spielberger, 2003; Litman et al., 2005). This is distinct from curiosity as a transient state, which is a stimulus-driven motivational state to acquire new information(Berlyne, 1954; Collins et al., 2004). Therefore, while a brief poem may not necessarily provoke a perceptual or stimulus-driven state of curiosity in the participants, it has the potential to activate their intrinsic exploratory behavioral traits, encouraging a deeper intellectual engagement with the content.

Hence we rewrote the line as: "**The contribution of aesthetic appeal appreciation to the creativity judgment may have been more fostered by the intrinsic behavioural traits of highly curious readers which compel them to delve more deeply into the unfamiliar aspects of the poems."**

23. Lines 488-489 "the positive influence of surprise on creativity..." 'Positive influence' is a very ambiguous and term. Consider rephrasing.

Our response: Thanks for this suggestion. We have now modified the line as: **"the influence of surprise on creativity judgment was more pronounced among individuals with lower levels of curiosity."**

24. Lines 494-496: "Firstly, it suggests that emotions such as amazement, wonder, information-seeking curiosity, and knowledge hunger, when experienced at higher levels, have a stronger impact" You did not measure any of those. Why are you extrapolating?

Our response: We agree with your comment. We computed personality traits related to those emotions using self-reports on valid questionnaires. A substantial body of research supports the method of self-reports in personality research (Shiota et al., 2007; Belfi et al., 2018; Hitsuwari & Nomura, 2022; Paulhus & Vazire, 2007; Robins et al., 2007). However, we omitted those lines in the revised manuscript.

25. Please revise the Discussion section with relevant claims and avoid any unwarranted and whimsical claims. Please try to make the discussion more scientific and less poetic.

Our response: The "Discussion" section has been thoroughly revised and modified as suggested.

26. Regarding the lack of effect of 'arousal'. Could it be that participants did not understand what it is supposed to be?

Our response: In the instruction page, for arousal, participants were asked: "How stimulating (higher Scores) or relaxing (lower Scores) did you feel when you read the poem?" (Belfi et al., 2018; Mehl et al., 2023; Hitsuwari & Nomura, 2022; Frame et al., 2023). Thus, we anticipated that participants comprehended the concept of arousal.

Of note, despite exhibiting significance (b = 0.07, SE = 0.02, t = 4.28, p < 0.001), the inclusion of arousal did not substantially improve the explanatory predictive power of the model which led to its exclusion as a viable predictor (see Model 4 in the Table 3 below for comparison of models). Therefore, the lack of a univariate effect for arousal effect was not the actual reason to exclude arousal as a potential predictor.

Table 3. Model comparison to identify the best model fit comprising aesthetic appeal, felt valence, and surprise.

Information Criteria	Null Model	Model 1	Model 2	Model 3	Model 4	Model 5
AIC	11160.3	9586.5	9325.2	9032.7	9016.8	9018.4
BIC	11178.7	9611.1	9356	9069.6	9059.8	9067.6
R^2	0	0.26	0.29	0.33	0.33	0.33
$\Delta\chi^2$		1575.73***	263.277***	294.514***	17.962***	0.401

Note: Aesthetic appeal, felt valence, surprise, arousal and clarity are included sequentially to Model 1 to Model 5; all models are compared hierarchically, i.e., Model 1 is compared to Null Model, Model 2 is compared to Model 1 and so on ; AIC = Akaike Information Criterion; BIC = Bayesian Information Criterion; R^2 = proportion of variation explained by fixed effects (Nakagawa & Schielzeth, 2013); Δχ2 = Likelihood ratio test statistic for comparison of models. Significance codes: 0 '***' 0.001 '**' 0.01 '*' 0.05 '.' 0.1 ' ' 1

27. About limitations (from line 524) – please set a new section with title "Limitations"

Our response: Thanks for the suggestion. We set a new section with the title "Limitations" as advised.

Our responses to the Reviewer's comments

1: Used terminology: Most problematic is, in my view, the use of the term "creativity". In my opinion, creativity is a state of mind or a quality of people (or – more precisely – a quality of humans that allows them to create something new). Weisberg – for example – defines "creativity" as (a) "the capacity to produce [novel] works [with value]" and (b) "the activity of generating such products" (in: Robert W. Weisberg: Creativity, p. 4). But it is NOT a property of works of art. However, in the sentence: "... different individuals may hold contrasting views on the creativity of a particular poem ...", creativity is spoken of as if it were an inherent property of poems, not an ability of the person who wrote the poem. Similar is the use of the word throughout the article. This is relevant to the article in that it is not entirely clear what exactly is being measured. Particularly, as the concept and its operationalisation are not explained anywhere. The item used in the questionnaire to assess "creativity" again treats it as an inherent quality of the poems. For a better understanding of the study, it would therefore be imperative to discuss the unconventional use of the term, its implied meaning and its possible impact on the participants of the study. In the literature cited in the manuscript "creativity" is - as far as I know – also seen as a characteristic of people (i.e., the ability of people to be creative). The connection between creativity and basic character traits found in other studies is therefore only indirectly related to this study, if at all. In other studies, various characteristics of an individual were related to each other (e.g., is extroversion related to creativity?). The present study, on the other hand, investigated which character traits of the viewers had an influence on the evaluation of the poems as ... - well, what exactly? I think it is therefore urgent to **(a) explain how the term "creativity" is used in the article, (b) critically analyse the relationship between the use of the term in the article and in the cited literature**, and **(c) discuss in detail the potential effects of its unconventional operationalisation on the results of the study**. Note: Despite my reservations, I use the term "creativity" in a similar way as it is used in the manuscript. However, this is simply for lack of alternatives, as I am not quite sure what exactly was measured in the study.

Our response: We would like to thank you for your insightful comments. We understand your concern regarding the operationalization of creativity in our study. Let us provide further clarifications as follows.

The concept of creativity as articulated by Mel Rhodes in 1961, through his 4-P model of creativity, offers a multifaceted perspective. Rhodes described creativity as a phenomenon where an individual (Person) communicates a new concept (Product), underpinned by mental activity (Process) and environmental factors (Press) (Rhodes, 1961). In this model, creativity is defined through four lenses: person, product, process, and press. In our study, we specifically focused on the 'product' aspect of creativity, specifically poetry, and operationalized "creativity" as the poem's creative potential. Of note, our approach is in line with numerous past studies which have investigated the creativity evaluation of various types of products/artefacts, such as ideas (Lloyd-Cox et al., 2022) musical compositions (Pinho et al., 2014; Zioga et al., 2020) short stories (Toivainen et al., 2021) and product concepts (Guenther et al., 2021), to name a few. This approach enabled us to explore the internal

model of how individuals judge the creativity of poems, recognizing that such judgments may be inherently subjective and influenced by individual personality traits.

We acknowledge that this operationalization the reviewer has found is different from the more traditional view of creativity as a characteristic of individuals. This choice was made to expand the understanding of creativity beyond the creator to the creation itself. In this study (and in numerous others which have looked at the creativity evaluation of a diverse range of artefacts/ideas), the adopted approach seeks to explore how creativity can also be perceived as an inherent quality of artistic works, in this case, poems. This perspective aligns with our aim to understand how different individuals perceive and evaluate the creativity of a poem based on their personality traits.

Further, we believe that viewing creativity as a characteristic of poems rather than solely a human trait offers a novel insight into how aesthetic appreciation and personality traits interplay in the perception and evaluation of poems. Further, this approach also allowed us to investigate how individual differences in traits like openness, intellect, awe-proneness, and epistemic curiosity influence the creativity evaluation of poems.

In conclusion, our study is a deliberate choice aimed at enriching the understanding of creativity evaluation in the context of poetry. By focusing on the product and the individual's interaction with it, we aimed to uncover nuanced aspects of how creativity is perceived and evaluated in the realm of English poems.

We deeply acknowledge Reviewer's comment and included this point in the "Limitations" section as follows:

"Finally, we acknowledge that the operationalization of 'creativity' is different from the more traditional view of creativity as a characteristic of individuals. Considering that creativity, as per the 4P Model (Rhodes, 1961), is defined through four lenses: person, product, process, and press, we strategically focused on the 'product' aspect of creativity, particularly in the context of poetry. We, here operationalized 'creativity' as the poem's creative potential. This choice was made to expand the understanding of creativity beyond the creator to the creation itself. Our approach is in line with numerous past studies which have investigated the creativity evaluation of various types of products/artefacts, such as ideas (Lloyd-Cox et al., 2022), musical compositions (Pinho et al., 2014; Zioga et al., 2020) short stories (Toivainen et al., 2021), and product concepts (Guenther et al., 2021), to name a few. This approach enabled us to explore the internal model of how individuals judge the creativity of poems, recognizing that such judgments may be inherently subjective and influenced by individual personality traits."

2: Operationalisation of the factors: The other variables for recording the aesthetic quality of the poems were also - if I understand correctly - assessed by only one item. Here, too, the question arises as to whether the operationalisation of the question is appropriate. Do the study participants understand what is meant when they have to evaluate whether a poem is "clear"? To what extent does the interpretation of the question vary individually? Are there studies that have examined the validity of these items to capture the underlying concept?

Our response: We appreciate your concern about the operationalization of variables and the potential variability in how study participants interpreted single-item assessments. In our study, participants were asked to evaluate each poem based on the following single-item measures:

1. Clarity: How clear was the content of the poem?

2. Aesthetic appeal: How aesthetically appealing was the poem?

3. Felt valence: How positive (higher scores) or negative (lower scores) did you feel when you read the poem?

4. Felt arousal: How stimulating (higher scores) or relaxing (lower scores) did you feel when you read the poem?

5. Surprise: How Surprising was this poem? By "Surprise" we mean a contrast to expectation in the concept of the poem.

6. Creativity: How Creative was this poem? Please answer based on your intuitive understanding of creativity of poem.

We acknowledge that the individual interpretation of these questions was not explicitly studied in our experiment. However, the use of such single-tem measures is a common practice in the psychometrics of creativity and aesthetics (Lloyd-Cox et al., 2022; Chamberlain et al., 2018; Strijbosch et al., 2022); importantly, single-item measures have also been routinely used in the evaluation of poetry (Belfi et al., 2018; Frame et al., 2023; Mehl et al., 2023; Hitsuwari & Nomura, 2022a, 2022b, 2022c; Papp-Zipernovszky et al., 2021). Nevertheless, we have discussed this issue in the "Limitations" section of the manuscript as follows:

"…we assessed the variables using single-item measures, a common practice in assessing aesthetics in visual art (Hassenzahl, 2004; Chamberlain et al., 2018; Strijbosch et al., 2022), poetry (Belfi et al., 2018; Frame et al., 2023; Mehl et al., 2023; Hitsuwari & Nomura, 2022a, 2022b, 2022c; Papp-Zipernovszky et al., 2021), and music (Zhang & Schubert, 2019). However, we also recognize the potential variability in individual interpretation of the questions remains unexplored. Employing multiple items for variable assessment could have offered psychometric advantages, particularly in enhancing reliability and validity (Bergkvist & Rossiter, 2007)."

3: Selection of poems: The number of poems used for the study is rather small, 36, and - probably - not representative. Especially since the poems were "carefully selected" (by whom, actually?), it is to be feared that the choice of poems might have influenced the result. I understand that this is a necessary sacrifice to make in empirical studies. Especially since the study design almost certainly excludes an influence of the selection of the poems on the result. But I think the issue should at least be discussed as one of the shortcomings of the study. It would also be helpful if it could be shown what measures were taken to ensure that the selection was not biased.

Our response: Many thanks for this comment with which we agree. The chosen set of poems in our study does not constitute a fully representative sample of English poems, which could limit the generalizability of the results to a certain extent. In the revised manuscript, we have now included full details of the poem selection procedure as follows:

“Initially, we selected 108 poems from various online resources, including Poetry.org (http://www.poetry.org/), the Poetry Foundation (https://www.poetryfoundation.org/), and the Academy of American Poets (https://poets.org/). These poems were subsequently rated for "surprise" on a scale of 1 to 7 by M.D, one of the co-authors who is a Professor of Creative Writing with domain-specific expertise and an award-winning poet. Following this evaluation, we shortlisted 36 as the experimental stimuli: 18 with low surprise ratings (4 or lower) and 18 with high surprise ratings (6 or above). The chosen poems varied in structure, contents, lines, and word count (mean number of lines = 11, SD = 3.24; mean word count = 71.25, SD = 28.99). To represent a broad spectrum of English poems, we consciously chose not to limit our selection to a particular genre or form, like haiku or sonnets as done in previous studies (Belfi et al., 2018; Hitsuwari & Nomura, 2022; Papp-Zipernovszky et al., 2021).”

Minor issues: 1. Line 49: It is not clear to me what you mean by “poetry and its creative evaluation has not been adequately characterized”. First of all, it seems to me that it is not the "creative evaluation" that needs further investigation, but rather the "evaluation of creativity". It also seems that the authors have not considered a whole field of science that deals with this issue of creativity in poems. The authors could look at studies on foregrounding or the field of stylistics. Another research approach that would be relevant in this context is the work of Menninghaus and colleagues.

Our response: Thanks – you are right. We have changed the phrase “creative evaluation” to “evaluation of creativity”.

We appreciate your suggestion to explore foregrounding and the domain of stylistics. In future, conducting studies to investigate the influence of poetry affinity (Kraxenberger & Menninghaus, 2017), or the use of poetic language on various dimensions of emotional responses (e.g., joy, sadness, being moved, intensity, and positive affect), as well as aesthetic appreciation (e.g., beauty, liking, and melodiousness) would be valuable (Menninghaus et al., 2017).

2. Line 50 to 54: It is also not entirely clear to me why the subjectivity of aesthetic evaluation should be more pronounced in poetics than in other forms of art. Personal experience always plays a role in the reception of art, regardless of the medium or art form, doesn't it? Furthermore, it seems to me that the introduction does not clearly separate the concepts of "beauty", "aesthetics" and "creativity". “Beauty” is in the eyes of the beholder. Why then do different observers have different views of the “creativity” with which a poem was written? Are "beauty" and "creativity" the same thing? Or are they causally linked? And if we already know that what is the point of the study?

Our response: We highly appreciate and acknowledge your comment on the fundamental aspect of aesthetics. As mentioned in the article, the main focus of the study is to investigate how individual difference in specific personality traits, specifically Openness, Intellect, Awe-proneness, and Epistemic Curiosity, shape the implicit associations between subjective experiences and the judgment of creativity of English poems.

While we acknowledge the importance of the age-old questions mentioned by the reviewer, we have purposefully avoided answering them as they are beyond the scope of this current study.

3. Line 62-64: In much of the introduction, the authors make assertions without citing any valid references. For example, I do not understand why "creativity" should be a less tangible construct than "preference".

Our response: We acknowledge your comment. We omitted the lines in the revised manuscript.

4. Line 79 (and others): Some sentences end without a period or have commas before the period (line 102).

Our response: Thanks. We have corrected as appropriate.

5. Line 457-467: In the discussion, an indirect connection is made between the ambiguity of expression in a poem and aesthetic appeal via the evaluation of "awe-proneness". It is stated that "other studies" have reported a connection between "awe-proneness" and the ability to tolerate ambiguity. But, on the one hand, this seems to concern only one study, and on the other hand, this is an indirectly established connection. I therefore think it is appropriate to formulate the conclusion (which I do not want to doubt) more cautiously (lines 465 and 466). In order to substantiate the thesis a little, it might be helpful to support it a little with findings from the studies on "foregrounding" (British stylistics or Russian formalism).

Our response: We acknowledge your valuable advice. We have now excluded the lines reporting a connection between awe-proneness and ambiguity tolerance.

References:

Acar, S., Burnett, C., & Cabra, J. F. (2017). Ingredients of creativity: Originality and more. *Creativity Research Journal*, *29*(2), 133–144. https://doi.org/10.1080/10400419.2017.1302776

Barron, F. (1955). The disposition toward originality. *The Journal of Abnormal and Social Psychology*, *51*, 478–485. https://doi.org/10.1037/h0048073

Belfi, A. M., Vessel, E. A., & Starr, G. G. (2018). Individual ratings of vividness predict aesthetic appeal in poetry. *Psychology of Aesthetics, Creativity, and the Arts*, *12*(3), 341–350. https://doi.org/doi:10.1037/aca0000153

Bergkvist, L., & Rossiter, J. R. (2007). The predictive validity of multiple-item versus single-item measures of the same constructs. *Journal of Marketing Research*, *44*(2), 175–184. https://doi.org/https://doi.org/10.1509/jmkr.44.2.175

Berlyne, D. E. (1954). A theory of human curiosity. *British Journal of Psychology*, *45*(3), 180.

Chamberlain, R., Mullin, C., Scheerlinck, B., & Wagemans, J. (2018). Putting the art in artificial: Aesthetic responses to computer-generated art. *Psychology of Aesthetics, Creativity, and the*

Arts, *12*(2), 177. https://doi.org/https://psycnet.apa.org/doi/10.1037/aca0000136

Chamorro-Premuzic, T., & Furnham, A. (2004). Art judgment: a measure related to both personality and intelligence? *Imagination, Cognition and Personality*, *24*(1), 3–24. https://doi.org/https://doi.org/10.2190/U4LW-TH9X-80M3-NJ54

Chamorro-Premuzic, T., Reimers, S., Hsu, A., & Ahmetoglu, G. (2009). Who art thou? Personality predictors of artistic preferences in a large UK sample: The importance of openness. *British Journal of Psychology*, *100*(3), 501–516. https://doi.org/https://doi.org/10.1348/000712608X366867

Chotlos, J. W. (1944). IV. A statistical and comparative analysis of individual written language samples. *Psychological Monographs*, *56*(2), 75. https://doi.org/https://psycnet.apa.org/doi/10.1037/h0093511

Collins, R. P., Litman, J. A., & Spielberger, C. D. (2004). The measurement of perceptual curiosity. *Personality and Individual Differences*, *36*(5), 1127–1141. https://doi.org/https://doi.org/10.1016/S0191-8869(03)00205-8

Costa Jr, P. T., & McCrae, R. R. (1992). Four ways five factors are basic. *Personality and Individual Differences*, *13*(6), 653–665. https://doi.org/https://doi.org/10.1016/0191-8869(92)90236-I

Costa, P. T., & McCrae, R. R. (2008). The revised neo personality inventory (neo-pi-r). *The SAGE Handbook of Personality Theory and Assessment*, *2*(2), 179–198.

DeYoung, C. G., Shamosh, N. A., Green, A. E., Braver, T. S., & Gray, J. R. (2009). Intellect as distinct from openness: Differences revealed by fMRI of working memory. *Ournal of Personality and Social Psychology*, *97*(5), 883–892. https://doi.org/https://psycnet.apa.org/doi/10.1037/a0016615

DeYoung, C. G., Grazioplene, R. G., & Peterson, J. B. (2012). From madness to genius: The Openness/Intellect trait domain as a paradoxical simplex. *Journal of Research in Personality*, *46*(1), 63–78. https://doi.org/https://doi.org/10.1016/j.jrp.2011.12.003

DeYoung, C. G., Peterson, J. B., & Higgins, D. M. (2005). Sources of openness/intellect: Cognitive and neuropsychological correlates of the fifth factor of personality. *Journal of Personality*, *73*(4), 825–858. https://doi.org/https://doi.org/10.1111/j.1467-6494.2005.00330.x

DeYoung, C. G., Quilty, L. C., & Peterson, J. B. (2007). Between facets and domains: 10 aspects of the Big Five. *Journal of Personality and Social Psychology*, *93*(5), 880.

DeYoung, C. G., Quilty, L. C., Peterson, J. B., & Gray, J. R. (2014). Openness to Experience, Intellect, and Cognitive Ability. *Journal of Personality Assessment*, *96*(1), 46–52. https://doi.org/10.1080/00223891.2013.806327

Faul, F., Erdfelder, E., Lang, A.-G., & Buchner, A. (2007). G* Power 3: A flexible statistical power analysis program for the social, behavioral, and biomedical sciences. *Behavior Research Methods*, *39*(2), 175–191.

Frame, J., Mehl, K., Head, K., & Belfi, A. (2023). *The influence of sensory modality on aesthetic judgments of poetry*. https://doi.org/https://doi.org/10.31234/osf.io/cv6px

Gabrielsson, A. (2001). Emotion perceived and emotion felt: Same or different? *Musicae Scientiae*, *5*(1_suppl), 123–147. https://doi.org/10.1177/10298649020050S105

Guenther, A., Eisenbart, B., & Dong, A. (2021). Creativity and successful product concept selection for innovation. *International Journal of Design Creativity and Innovation*, *9*(1), 3–19. https://doi.org/https://doi.org/10.1080/21650349.2020.1858970

Hassenzahl, M. (2004). The interplay of beauty, goodness, and usability in interactive products. *Human–Computer Interaction*, *19*(4), 319–349. https://doi.org/https://doi.org/10.1207/s15327051hci1904_2

Hitsuwari, J., & Nomura, M. (2022). How individual states and traits predict aesthetic appreciation of haiku poetry. *Empirical Studies of the Arts*, *40*(1), 81–99. https://doi.org/10.1177/0276237420986420

Jacobs, A. M. (2017). Quantifying the beauty of words: A neurocognitive poetics perspective. In *Frontiers in Human Neuroscience* (Vol. 11). https://www.frontiersin.org/articles/10.3389/fnhum.2017.00622

Jacobs, A. M., & Kinder, A. (2018). What makes a metaphor literary? Answers from two computational studies. *Metaphor and Symbol*, *33*(2), 85–100.

Johnson, D. R., Kaufman, J. C., Baker, B. S., Patterson, J. D., Barbot, B., Green, A. E., van Hell, J., Kennedy, E., Sullivan, G. F., Taylor, C. L., Ward, T., & Beaty, R. E. (2022). Divergent semantic integration (DSI): Extracting creativity from narratives with distributional semantic modeling. *Behavior Research Methods*. https://doi.org/10.3758/s13428-022-01986-2

Kraxenberger, M., & Menninghaus, W. (2017). Affinity for poetry and aesthetic appreciation of joyful and sad poems. In *Frontiers in Psychology* (Vol. 7). https://www.frontiersin.org/articles/10.3389/fpsyg.2016.02051

Kuiken, D., Douglas, S., & Kuijpers, M. (2021). Openness to experience, absorption-like states, and the aesthetic, explanatory, and pragmatic effects of literary reading. *Scientific Study of Literature*, *11*(2), 148–195. https://doi.org/https://doi.org/10.1075/ssol.21007.kui

Lau, J. H., Cohn, T., Baldwin, T., Brooke, J., & Hammond, A. (2018). *Deep-speare: A joint neural model of poetic language, meter and rhyme*. https://doi.org/10.48550/arxiv.1807.03491

Litman, J. A., & Spielberger, C. D. (2003). Measuring epistemic curiosity and its diversive and specific components. *Journal of Personality Assessment*, *80*(1), 75–86. https://doi.org/10.1207/S15327752JPA8001_16

Litman, J., Hutchins, T., & Russon, R. (2005). Epistemic curiosity, feeling-of-knowing, and exploratory behaviour. *Cognition and Emotion*, *19*(4), 559–582. https://doi.org/10.1080/02699930441000427

Lloyd-Cox, J., Pickering, A., & Bhattacharya, J. (2022). Evaluating creativity: How idea context and rater personality affect considerations of novelty and usefulness. *Creativity Research Journal*, *34*(4), 373–390. https://doi.org/10.1080/10400419.2022.2125721

Loewenstein, G. (1994). The psychology of curiosity: A review and reinterpretation. *Psychological Bulletin*, *116*(1), 75.

Malvern, D. D., & Richards, B. J. (1997). A new measure of lexical diversity. *British Studies in Applied Linguistics*, *12*, 58–71.

Marin, M. M., & Bhattacharya, J. (2010). Music induced emotions: some current issues and cross-modal comparisons. In J. Hermida & M. Ferrero (Eds.), *Music education* (pp. 1–38). Hauppauge, NY: Nova Science Publishers.

Mastria, S., Agnoli, S., & Corazza, G. E. (2019). How does emotion influence the creativity evaluation of exogenous alternative ideas? *PLOS ONE*, *14*(7), e0219298. https://doi.org/10.1371/journal.pone.0219298

McCarthy, P. M., & Jarvis, S. (2007). vocd: A theoretical and empirical evaluation. *Language Testing*,

24(4), 459–488. https://doi.org/https://doi.org/10.1177/0265532207080767

McCarthy, P. M., & Jarvis, S. (2010). MTLD, vocd-D, and HD-D: A validation study of sophisticated approaches to lexical diversity assessment. *Behavior Research Methods*, *42*(2), 381–392. https://doi.org/10.3758/BRM.42.2.381

McCrae, R. R., & Costa Jr, P. T. (1997). Conceptions and correlates of openness to experience. In *Handbook of personality psychology*. Academic Press. https://doi.org/https://doi.org/10.1016/B978-012134645-4/50032-9

McCrae, R. R. (1996). Social consequences of experiential openness. *Psychological Bulletin*, *120*(3), 323–337. https://doi.org/https://psycnet.apa.org/doi/10.1037/0033-2909.120.3.323

McCrae, Robert R. (2007). Aesthetic chills as a universal marker of openness to experience. *Motivation and Emotion*, *31*(1), 5–11. https://doi.org/10.1007/s11031-007-9053-1

McManus, I. C., & Furnham, A. (2006). Aesthetic activities and aesthetic attitudes: Influences of education, background and personality on interest and involvement in the arts. *British Journal of Psychology*, *97*(4), 555–587. https://doi.org/https://doi.org/10.1348/000712606X101088

Mehl, K., Gugliano, M., & Belfi, A. M. (2023). The role of imagery and emotion in the aesthetic appeal of music, poetry, and paintings. *Psychology of Aesthetics, Creativity, and the Arts*, No Pagination Specified-No Pagination Specified. https://doi.org/10.1037/aca0000623

Menninghaus, W., Wagner, V., Wassiliwizky, E., Jacobsen, T., & Knoop, C. A. (2017). The emotional and aesthetic powers of parallelistic diction. *Poetics*, *63*, 47–59. https://doi.org/https://doi.org/10.1016/j.poetic.2016.12.001

Meyer, W. U., Reisenzein, R., & Schützwohl, A. (1997). Toward a process analysis of emotions: The case of surprise. *Motivation and Emotion*, *21*(3), 251–274. https://doi.org/https://doi.org/10.1023/A:1024422330338

Meyer, J., Thoma, G.-B., Kampschulte, L., & Köller, O. (2023). Openness to experience and museum visits: Intellectual curiosity, aesthetic sensitivity, and creative imagination predict the frequency of visits to different types of museums. *Journal of Research in Personality*, *103*, 104352. https://doi.org/https://doi.org/10.1016/j.jrp.2023.104352

Meyer, W.-U., Niepel, M., Rudolph, U., & Schützwohl, A. (1991). An experimental analysis of surprise. *Cognition & Emotion*, *5*(4), 295–311.

Meyer, W.-U., Reisenzein, R., & Schützwohl, A. (1997). Toward a process analysis of emotions: The case of surprise. *Motivation and Emotion*, *21*(3), 251–274. https://doi.org/10.1023/A:1024422330338

Nakagawa, S., & Schielzeth, H. (2013). A general and simple method for obtaining R2 from generalized linear mixed-effects models. *Methods in Ecology and Evolution*, *4*(2), 133–142.

Noordewier, M. K., & Breugelmans, S. M. (2013). On the valence of surprise. *Cognition and Emotion*, *27*(7), 1326–1334. https://doi.org/10.1080/02699931.2013.777660

Nusbaum, E. C., & Silvia, P. J. (2011). Are Openness and Intellect distinct aspects of Openness to Experience? A test of the O/I model. *Personality and Individual Differences*, *51*(5), 571–574. https://doi.org/https://doi.org/10.1016/j.paid.2011.05.013

Obermeier, C., Menninghaus, W., von Koppenfels, M., Raettig, T., Schmidt-Kassow, M., Otterbein, S., & Kotz, S. (2013). Aesthetic and emotional effects of meter and rhyme in poetry. In *Frontiers in Psychology* (Vol. 4). https://www.frontiersin.org/articles/10.3389/fpsyg.2013.00010

Oleynick, V. C., DeYoung, C. G., Hyde, E., Kaufman, S. B., Beaty, R. E., & Silvia, P. J. (2017). Openness/intellect: The core of the creative personality. In *The Cambridge handbook of creativity and personality research.* (pp. 9–27). Cambridge University Press. https://doi.org/10.1017/9781316228036.002

Papp-Zipernovszky, O., Mangen, A., Jacobs, A., & Lüdtke, J. (2021). Shakespeare sonnet reading: An empirical study of emotional responses. *Language and Literature*, *31*(3), 296–324. https://doi.org/10.1177/09639470211054647

Paulhus, D. L., & Vazire, S. (2007). The self-report method. *Handbook of Research Methods in Personality Psychology*, *1*(2007), 224–239.

Pietras, K., & Ganczarek, J. (2022). Aesthetic reactions to violations in contemporary art: The role of expertise and individual differences. *Creativity Research Journal*, *34*(3), 324–338.

Pinho, A. L., de Manzano, Ö., Fransson, P., Eriksson, H., & Ullén, F. (2014). Connecting to create: expertise in musical improvisation is associated with increased functional connectivity between premotor and prefrontal areas. *Journal of Neuroscience*, *34*(18), 6156–6163. https://doi.org/https://doi.org/10.1523/JNEUROSCI.4769-13.2014

Rasse, C., Onysko, A., & Citron, F. M. M. (2020). Conceptual metaphors in poetry interpretation: A psycholinguistic approach. *Language and Cognition*, *12*(2), 310–342. https://doi.org/10.1017/langcog.2019.47

Rhodes, M. (1961). An analysis of creativity. *The Phi Delta Kappan*, *42*(7), 305–310. https://doi.org/https://www.jstor.org/stable/20342603

Robins, R. W., Tracy, J. L., & Sherman, J. W. (2007). What kinds of methods do personality psychologists use. *Handbook of Research Methods in Personality Psychology*, 673–678.

Rumelhart, D. E. (1984). *Schemata and the cognitive system.*

Runco, M. A., & Jaeger, G. J. (2012). The Standard Definition of Creativity. *Creativity Research Journal*, *24*(1), 92–96. https://doi.org/10.1080/10400419.2012.650092

Russell, J. A. (1980). A circumplex model of affect. *Journal of Personality and Social Psychology*, *39*(6), 1161.

Shiota, M. N., Keltner, D., & Mossman, A. (2007). The nature of awe: Elicitors, appraisals, and effects on self-concept. *Cognition and Emotion*, *21*(5), 944–963. https://doi.org/10.1080/02699930600923668

Simonton, D. K. (2012). Taking the U.S. patent office criteria seriously: A quantitative three-criterion creativity definition and its implications. *Creativity Research Journal*, *24*(2–3), 97–106. https://doi.org/10.1080/10400419.2012.676974

Steen, G. J. (2009). From linguistic form to conceptual structure in five steps: Analyzing metaphor in poetry. *Cognitive Poetics*, 197–226.

Stein, M. I. (1953). Creativity and culture. *The Journal of Psychology*, *36*(2), 311–322. https://doi.org/http://dx.doi.org/10.1080/00223980.1953.9712897

Strijbosch, W., Vessel, E. A., Welke, D., Mitas, O., Gelissen, J., & Bastiaansen, M. (2022). On the neuronal dynamics of aesthetic experience: Evidence from electroencephalographic oscillatory dynamics. *Journal of Cognitive Neuroscience*, *34*(3), 461–479. https://doi.org/https://doi.org/10.1162/jocn_a_01812

Toivainen, T., Madrid-Valero, J. J., Chapman, R., McMillan, A., Oliver, B. R., & Kovas, Y. (2021).

Creative expressiveness in childhood writing predicts educational achievement beyond motivation and intelligence: A longitudinal, genetically informed study. *British Journal of Educational Psychology*, *91*(4), 1395–1413. https://doi.org/https://doi.org/10.1111/bjep.12423

Wassiliwizky, E., Koelsch, S., Wagner, V., Jacobsen, T., & Menninghaus, W. (2017). The emotional power of poetry: neural circuitry, psychophysiology and compositional principles. *Social Cognitive and Affective Neuroscience*, *12*(8), 1229–1240. https://doi.org/10.1093/scan/nsx069

Zhang, J. D., & Schubert, E. (2019). A Single Item Measure for Identifying Musician and Nonmusician Categories Based on Measures of Musical Sophistication. *Music Perception*, *36*(5), 457–467. https://doi.org/10.1525/mp.2019.36.5.457

Zioga, I., Harrison, P. M. C., Pearce, M. T., Bhattacharya, J., & Di Bernardi Luft, C. (2020). From learning to creativity: Identifying the behavioural and neural correlates of learning to predict human judgements of musical creativity. *NeuroImage*, *206*, 116311. https://doi.org/https://doi.org/10.1016/j.neuroimage.2019.116311
