# Supplementary material for: Beyond the words: Exploring individual differences in the evaluation of poetic creativity
Source: PLoS One. 2024 Oct 3;19(10):e0307298. doi: 10.1371/journal.pone.0307298 (PMC11449365; doi:10.1371/journal.pone.0307298)
Supplement: Attachment — Submitted filename: PLOS ONE-D-23-18669_R1_reviewer-poetry-commentsFeb2024.pdf [file pone.0307298.s003.pdf]

PLOS ONE

Beyond the words: Exploring individual differences in the evaluation of poetic creativity

--Manuscript Draft--

**Manuscript Number:**	PONE-D-23-18669R1
**Article Type:**	Research Article
**Full Title:**	Beyond the words: Exploring individual differences in the evaluation of poetic creativity
**Short Title:**	The moderating role of individual differences in the evaluation of poetic creativity
**Corresponding Author:**	SOMA CHAUDHURI, MSc<br>Goldsmiths University of London Department of Psychology<br>LONDON, Select State UNITED KINGDOM
**Keywords:**	poetry, creativity, evaluation, personality traits, moderation
**Abstract:**	Poetry is arguably the most creative expression of language that can evoke diverse subjective experiences, such as emotions and aesthetic responses, in different individuals, subsequently influencing their judgment of the creativity of poem. This study investigated how personality traits, specifically openness, intellect, awe-proneness, and epistemic curiosity, shape the implicit associations between subjective experiences and the judgment of creativity of thirty-six English language poems. One hundred and twenty-nine participants rated each poem across six dimensions: clarity, aesthetic appeal, felt valence, felt arousal, surprise, and overall creativity. First, we obtained a parsimonious model of creativity comprising aesthetic appeal, felt valence, and surprise as potential predictors of poetic creativity. Subsequently, using multilevel analysis, we investigated the interactions between the four personality traits and these three predictors. Among the personality traits, openness emerged as the primary moderator in predicting judgments of poetic creativity, followed by curiosity and awe-proneness. Among the predictors, aesthetic appeal was moderated by all four personality traits, while surprise was moderated by openness, awe-proneness, and curiosity. Valence, on the other hand, was moderated by openness only. Our findings provide novel insights into how individual differences influence the evaluation of the creativity of poems.
**Order of Authors:**	SOMA CHAUDHURI, MSc
	ALAN PICKERING, PhD
	MAURA DOOLEY, PhD
	JOYDEEP BHATTACHARYA, PhD
**Response to Reviewers:**	Our responses to the Editor’s comments<br><br>1. Lines 28-29: “...of a broad range of English language poems ... rated each poem...” You have not stated yet how many poems were used. Instead of 'broad range', state the number<br>Our response: Thank you for your suggestion. The number of poems has now been mentioned in the manuscript, as follows: “This study investigated how personality traits, specifically openness, intellect, awe-proneness, and epistemic curiosity, shape the implicit associations between subjective experiences and the judgment of creativity of thirty-six English language poems.”<br><br>2. Line 106: “meter (36, 40), , metaphors (35), (41,42,43), and” either there is bad formatting, or something is missing on this line.<br>Our response: Thank you. Formatting has been revised.<br><br>3. Line 117: “we considered both dimensions of emotion – valence and arousal.” Both 'valence' and 'arousal' are technical term in psychology and related fields. Please be kind enough to explain those terms to the general audience (a footnote may suffice). Moreover, since 'felt valence' was found to be important in your work, explain to readers what it is.<br>Our response: Thank you for the suggestion. We explained the terms in the revised manuscript as suggested. The lines read as follows:

Powered by Editorial Manager® and ProduXion Manager® from Aries Systems Corporation

“According to the two-dimensional circumplex model of emotion, emotional states are represented along two orthogonal dimensions: valence (pleasure-displeasure: horizontal axis) and arousal (degree of arousal: vertical axis) (Russell, 1980). Poetry evokes strong emotions (Wassiliwizky et al., 2017) and emotional states can influence creativity evaluation (Mastria et al., 2019)…. Of note, perceived and felt emotions may be distinct (Gabrielsson, 2001; Marin & Bhattacharya, 2010). Perceived emotions are the emotions evoked by the stimuli whereas felt emotions are the emotions felt by the perceivers. In our study, we focused on the felt emotions, i.e., the emotions felt by the reader while reading the poem, rather than the perceived emotion, i.e., the emotion recognized in the poem. Therefore, felt valence reveals the extent to which the readers felt positive or negative emotions while reading the poems, whereas felt arousal here reveals how intense it was felt by the readers.”

4. Lines 122-125: “Surprise is considered as a stronger predictor of creativity than value after...” The description of 'Surprise' dimension is very dry and technical (unlike the other descriptions), and it does not explain what is 'Surprise' in this context. Also what is 'value' in the above sentence? Did you mean valence?
Our response: Thank you very much for the comment. We have now expanded our explanation to clarify the specific meaning of “surprise’ within the context of our study. The section reads as: “Surprise is usually a short-lived emotion elicited by events that deviate from an established schema (Meyer et al., 1997; Meyer et al., 1991; Noordewier & Breugelmans, 2013). Of note, a schema, in this sense, refers to a component of the organism’s knowledge structure, activated by a specific stimulus (Rumelhart, 1984). Surprise is recognized as a key predictor of creativity (Simonton, 2012; Acar et al., 2017) and is also a robust predictor of aesthetic judgment of artwork (Pietras & Ganczarek, 2022). In the context of our study, the term “surprise” specifically denotes the degree to which there is a sudden and unexpected change in the context or theme of the poem.”

Response to your next question on “value”: Despite the lack of an agreed-upon definition, a general consensus over a standard definition of creativity (Runco & Jaeger, 2012; see also Stein, 1953; Barron, 1955) involves two factors: The first factor consists of characteristics such as novelty, originality, infrequency, or unusualness; and the second factor is related to usefulness, value, utility, effectiveness, adaptability, or appropriateness (Acar et al., 2017). However, we excluded the line containing “value” to avoid confusion of readers as we did not explore any potential consequences of this term later in the article.

Line 127: “It is a broad range of traits...” What is “It”? Why do you begin with “It”? Please formulate the description clearly.
Our response: The line has been amended as: Openness to experience is a broad range of traits, from intellectual abilities to aesthetic and artistic interests (Oleynick et al., 2017;DeYoung et al., 2012; Chamorro-Premuzic et al., 2009) and is most robustly associated with measures of creativity (McCrae, 1996).

Lines 130-132: “Openness/intellect … can be differentiated into two major aspects (64): openness and intellect.” Really? Isn't it a bit tautological? Please clarify – is it one predictor or two? If those are two predictors, why are they bundled together?
Our response: Actually, Costa Jr & McCrae, (1992) first introduced Openness to Experience as the fifth factor of personality traits. Later, DeYoung et al created the Openness/Intellect (O/I) model, which proposed that Openness to Experience has two major facets– the two “related but separable trait dimensions” (DeYoung et al., 2007, p.883): openness and intellect. Based on earlier work on the neurocognitive correlates of Openness to Experience (DeYoung et al., 2005), DeYoung et al., 2009 hypothesized that brain regions associated with working memory would predict the intellect facets but not the openness facets. Showing distinct effects of openness and intellect would provide evidence for the validity of both constructs (see Nusbaum & Silvia, 2011).
Hence, in our study, we are interested to explore the distinct influence of openness and intellect on the evaluation of creativity of poems.

5. Lines 152-153: “Earlier research has shown that openness influences attitudes toward targets that violate established schemas (68).” This is absolutely unclear, needs some elaboration. What are 'targets'? Targets of what? Which established schemas?

OF behavior? Of Social norms? Of poems? Of poetic structure? What is meant by 'violate' here?
Our response: We appreciate your comment. We revised the lines as: “As surprise is often experienced when individuals encounter unexpected or schema-discrepant events (Meyer et al., 1997; Meyer et al., 1991), we assumed that the assessment of expectancy violations in the judgment of poetic creativity necessitates a substantial degree of cognitive engagement. Consequently, we predicted that both Openness and Intellect would moderate the relationship between surprise and creativity. ”

6. Line 168: “aesthetic chill” Please explain what is aesthetic chill.
Our response: We explained the term. The line now reads: “Aesthetic chills are transient emotional responses (McCrae, 2007) to aesthetical stimuli. They can be described as experiencing a chill or wave of excitement when engaging with poetry or looking at a work of art (Costa & McCrae, 2008).”

7. Line 196: Please explain what is “Prolific®”
Our response: Explained as suggested: “The experiment was created using Qualtrics®, and the link was disseminated through Prolific®, a platform for participant recruitment.”

8. Section “Materials and methods” This section starts with 'Participants' section and it’s quite awkward. The 'Participants' section should be moved after the 'Materials' section.
Our response: Thank you. “Participants” section is now shifted after the “Materials” section, as suggested.

9. Lines 194-196: The 'Participants' section begins with a description of minimum sample size. This section needs to be clarified. What led you to focus on “80% power for a medium effect (^2 = 0.15) with squared multiple correlation ^2 = 0.13.” It seems rather arbitrary.
Our response: The section has been clarified as suggested. The section now reads as: “By using the G*Power software (v. 3.1.9.4), (Faul et al., 2007) we found that a minimum sample size of 98 was required to detect a medium effect size (f2 = 0.15) in a multiple linear regression, assuming a significance level of 0.05 and a statistical power of 80%. The criteria we used are widely-used conventional figures when estimating sample sizes. Additionally, we used the ‘samplesize_mixed’ function in R, which estimates the required sample size for linear mixed models (two-level-designs), based on power-calculation for standard design and adjusted for design effect for 2-level-designs (https://strengejacke.github.io/sjstats/). For our multilevel model, which considered 96 cluster groups and a small to medium effect size (Cohen’s d = 0.3), with 36 observations per cluster group (level-2-unit), we determined that a total of 965 observations would be necessary. This translates to a minimum of 27 participants (965/36).”

10. Also, you did not specify where the participants came from, in terms of education, profession, English language proficiency (native/non-native) and age groups. Would it be possible to have some indications about those background variables?
Our response: As per the self-reported demographic questionnaires, all 96 participants (32 males, 63 females, 1 preferred not to say; mean age = 31.94 years, SD = 13.09) were fluent in English and came from a variety of educational backgrounds, with each holding at least a bachelor’s degree in any discipline. Additionally, our sample represented a diverse range of professions and ethnicities.
We included this information in the “Participants” section.

11. Line 196: “healthy participants” What does 'healthy' mean here? Maybe specify in a footnote. What criteria were used? Is this aspect really important?
Our response: Thank you for your comment. Considering that the “health” aspect was not a significant focus of our study, we omitted the term “healthy.”

12. Line 202: “We described the main experimental procedures for this research to the participants” It is not clear what was explained. Maybe add an appendix? How did you explain 'valence' and 'arousal' to the participants?
Our response: The instructions to the participants are now included in the main manuscript. The full paragraph now reads:

Powered by Editorial Manager® and ProduXion Manager® from Aries Systems Corporation

“Participants were briefed about the experimental procedure, which involved the assessment of a set of English poems on a 7-point Likert rating scale (1=extremely low; 7=extremely high) across various constructs, including clarity, aesthetic appeal, felt valence, arousal, surprise, and overall creativity. Additionally, participants were instructed to complete demographic and personality-related questions. We assured participants of the full confidentiality of their data, in compliance with the General Data Protection Regulation, and clarified that any published results would be non-identifiable. All participants provided informed consent (online) before data collection. Participants received a cash incentive of £7.50 per hour. The data collection period spanned from 27 January 2022 to 23 June 2022, and the data were accessed for research purposes only after this period.”
How did you explain 'valence' and 'arousal' to the participants?
For felt valence, participants were asked: “How positive (higher Scores) or negative (lower Scores) did you feel when you read the poem?” For arousal, they were asked: “How stimulating (higher Scores) or relaxing (lower Scores) did you feel when you read the poem?”

13. Section 'Materials': The procedure of poem selection is not described well. Selection sources (lines 224-227) should be described before the lexical metrics of selected poems (lines 213-223).
Our response: As suggested, the sources for poem selection have been described in the “Material” section:
“Initially, we selected 108 poems from various online resources, including Poetry.org (http://www.poetry.org/), the Poetry Foundation (https://www.poetryfoundation.org/), and the Academy of American Poets (https://poets.org/). These poems were subsequently rated for "surprise" on a scale of 1 to 7 by M.D, one of the co-authors who is a Professor of Creative Writing with domain-specific expertise and an award-winning poet. Following this evaluation, we shortlisted 36 as the experimental stimuli: 18 with low surprise ratings (4 or lower) and 18 with high surprise ratings (6 or above). The chosen poems varied in structure, contents, lines, and word count (mean number of lines = 11, SD = 3.2; mean word count = 71.3, SD = 29). To represent a broad spectrum of English poems, we consciously chose not to limit our selection to a particular genre or form, like haiku or sonnets as done in previous studies (Belfi et al., 2018; Hitsuwari & Nomura, 2022; (Papp-Zipernovszky et al., 2021).
13. How did you converge on the genres and themes that are reported in Table 1? Who provided the labels of genres and themes?
M.D, a co-author of our study who is a Professor of Creative Writing with domain-specific expertise and an award-winning poet, validated the genre and theme-specific information about the stimuli.

14. Lines 218-221. Concerning “lexical diversity (LD)”. Explain to readers what is LD, and why it is relevant for this study. There are many metrics for LD. Please describe which one you used. The legend of table 1 says “(LD = number of unique words/ total characters).” That should be explained in the text. Also, that is not a common metric of LD. Where did you adopt it from? “The average (SD) LD score for our stimuli set was 14.30 (1.7).” Switch “(SD) LD” to “LD (DS)”. Also explain to readers: is 14.30 high or low? What is the scale and what does the value mean?
Our response: Thanks for this valuable comment. While lexical diversity and semantic diversity may not be directly pertinent to our study, we decided to include them to capture the variedness and richness of vocabulary and semantics in our chosen stimuli. Of note, we rectified the lexical diversity scores (computed in R). Table1, originally presenting stimulus details, has been modified to incorporate updated Lexical Diversity and is now designated as Table S1. We intend to include it in the Supplementary section.The paragraph describing the diversity metrics has been modified as follows:
“Lexical diversity (LD) of a text refers to its lexical richness, indicating range and variety of vocabulary deployed in a text (McCarthy & Jarvis, 2007). For computing the LD of the poems, we used the type-token ratio (TTR) method, which calculates the ratio of unique words (types) to the total word count (tokens) (Chotlos, 1944). It ranges from 0 to 1: a higher TTR indicates a greater lexical diversity. The average lexical diversity across the poems is 0.77 with a standard deviation of 0.09, suggesting that, on average, about 77% of the words used in the poems are unique or different. Semantic diversity, on the other hand, refers to the range of contexts (i.e., semantic richness) in

Powered by Editorial Manager® and ProduXion Manager® from Aries Systems Corporation

which words are used (Johnson et al., 2022). To calculate the semantic diversity, we used divergent semantic integration (DSI) (http://semdis.wlu.psu.edu/), representing the mean semantic distance between all word pairs in a text; DSI varies from 0 to 1, where a higher score indicates that a broader collection of divergent ideas. The average DSI of our stimuli is 0.80 with a standard deviation of 0.03, indicating a high degree of semantic variety (see Table S1 in the Supplementary section for details)."
In the "Limitations" section, we added the limitations of TTR method for short texts as follows: "it is important to acknowledge that given the small word count of some of our poems and the implied limited vocabulary, the Type-Token Ratio (TTR) method might not yield reliable results due to constrained variability in word usage within small texts (Malvern & Richards, 1997; McCarthy & Jarvis, 2010)."
The table (Table S1 in the Supplementary section) containing the details of the stimuli will be submitted in the Supplementary section and OSF (https://osf.io/9mw7r/?view_only=07137f4871d146c790501f22bc7743d5).

TableS1. The details of the stimuli

15. Lines 222-224: Explain to readers what is DSI, and why it is relevant for this study. What is the scale and where on the scale is your value (0.8) located?
Our response: We explained DSI in the "Materials" section as follows:
"Semantic diversity, on the other hand, refers to the range of contexts (i.e., semantic richness) in which words are used (Johnson et al., 2022). To calculate the semantic diversity, we used divergent semantic integration (DSI) (http://semdis.wlu.psu.edu/), representing the mean semantic distance between all word pairs in a text; DSI varies from 0 to 1, where a higher score indicates that a broader collection of divergent ideas. The average DSI of our stimuli is 0.80 with a standard deviation of 0.03, indicating a high degree of semantic variety (see Table S1 in the Supplementary section for details)."

16. Lines 241-242: "Participants read a poem for 30 seconds" Explain why such a time limit was set? Was the poem still visible during creativity rating?
Our response: Given the concise nature of the poems (average number of lines = 11), we opted for a minimum reading time of 30 seconds. Participants were given a minimum of 30 seconds to read each poem. Following this period, they were allowed to proceed to the rating task. They were asked to rate the poems on six dimensions in the following order: clarity, aesthetic appeal, felt valence, felt arousal, surprise, and creativity, using a 7-point Likert scale (1: "Extremely Low" and 7: "Extremely High"). There was no time limit imposed for rating the poems. Of note, the poems remained visible during the rating process. This information has been included in the "Procedure" section.

17. Regarding the questions/measures on four personality traits: as those were shown to be correlated with creativity (Table 3). Please briefly explain the scales and value ranges for the personality traits measures. Moreover, please describe the distributions of values for all personality measures in your study. For example, As described in Table 2, it seems the four personality traits are also on 7-point scale. Can you establish that you had sufficient variability on the personality scores? It is not clear whether you participants represent sufficient difference on an Openness scale (and other scales). The SD for Openness is just 0.74 – are all participants approximately equally 'open'?. Do you have sufficient distribution for levels of intellect? Sufficient in terms of representing the general public?
Our response:
Of note, there was a typo in our curiosity rating data. We have rectified this in the revised manuscript. Therefore, the curiosity data in the Tables had undergone some minor changes, but without any significant impact on the findings and their implications.
scales for the personality traits measures: All scales are 1-7
value ranges:
Openness: min = 3.0; max = 6.4; mean = 5.02; SD = 0.74
Intellect: min = 2.7; max = 6.4; mean = 4.7; SD = 0.9
Awe-proneness: min = 1.83; max = 7; mean = 5.11; SD = 1.14
Curiosity: min = 3.5; max = 7; mean = 5.58; SD = 0.86

Variability in openness scores: 0.55 (moderate, suggesting a reasonable spread of scores among participants.)
Variability in intellect scores: 0.81 (high, indicating a broader range of scores among participants.)
Variability in awe-proneness scores: 1.31 (high, suggesting a diverse range of responses among participants.)
Variability in curiosity scores: 0.76 (moderate, indicating a reasonable distribution of scores.)
Therefore, personality measures capture overall diverse range which indicates that participants do not uniformly represent specific traits.

18. Variable names for personality traits are different in Table 2 and 3. Please adjust.
Our response: Thanks for pointing this. The Tables have been rectified accordingly.

19. Table 5 includes a variable "Expertise" but that variable was not described in the texts. Please describe appropriately.
Our response: We apologize for this inadvertent inclusion. The variable "Expertise" has been excluded from Table 5.

20. Lines 431-433: "These results suggest that individuals with higher levels of imagination, a greater appreciation for aesthetic experiences, and a strong inclination towards art demonstrated..." This claim is not quite warranted. Did you measure imagination? "appreciation for aesthetic experiences" ? Did you measure inclination towards art? It seems you did not measure those, so how can you make any claims about those?
Our response: Thanks for your valuable comment. Openness, as operationalised by the NEO Personality Inventory-Revised (Costa & McCrae, 2008), is characterized by a range of attributes: a vivid imagination (O1: Fantasy), an appreciation of art and beauty (O2: Aesthetics), depth of emotions (O3: Feelings), an eagerness to try new things (O4: Actions), intellectual curiosity (O5: Ideas), and being liberal (O6: Values). Previous research has shown that openness involves a deep engagement with perceptual and aesthetic domains (DeYoung et al., 2014). Further, it is the best predictor of positive attitudes towards aesthetics and active participation in aesthetic activities, such as visiting museums, reading literature, and creating artwork (Meyer et al., 2023; McCrae & Costa, 1997; Chamorro-Premuzic & Furnham, 2004; Kuiken et al., 2021). Notably, greater involvement in aesthetic activities is often related to higher scores in the openness trait (McManus & Furnham, 2006).
Our findings reveal that individuals with higher levels of openness place more emphasis on aesthetic appeal when assessing the creativity of poems, in contrast to those with lower levels of openness.

21. Line 465-466; "Our study suggests that as the level of ambiguity increased with the rise in the aesthetic appeal of a poem,..." You did not measure levels of ambiguity. So why do you make such unwarranted claims?
Our response: We appreciate your comment. We removed the line: "In addition….appreciation of poems" from the Discussion section.

22. Lines 485-486: "may have been more fostered by the intrinsic drive of highly curious readers to explore the unknown" To 'explore the unknown' by reading a short poem in 30 seconds? Are you serious?
Our response: Thank you for your comment. However, we would like to clarify that the 30-second timeframe set in our study is the minimum, rather than the maximum, duration allotted for reading each poem. This approach ensures that participants must have spent at least 30 sec or more to engage with the poem. During the evaluation phase, there was no imposed time limit, and the poem remained visible, facilitating a more thorough assessment and appreciation of the poem's nuances without time pressure. Hence, this extended interaction provides an opportunity for the participants to delve into aspects of the unfamiliar depicted in the poem, potentially stimulating their natural curiosity and desire for knowledge.
Furthermore, our study focuses on epistemic curiosity, a personality trait, characterized by a desire for new information that motivates exploratory behaviour and knowledge acquisition (Berlyne, 1954; Loewenstein, 1994; Litman & Spielberger, 2003; Litman et al., 2005). This is distinct from curiosity as a transient state, which is a stimulus-driven

motivational state to acquire new information(Berlyne, 1954; Collins et al., 2004). Therefore, while a brief poem may not necessarily provoke a perceptual or stimulus-driven state of curiosity in the participants, it has the potential to activate their intrinsic exploratory behavioral traits, encouraging a deeper intellectual engagement with the content.
Hence we rewrote the line as: “The contribution of aesthetic appeal appreciation to the creativity judgment may have been more fostered by the intrinsic behavioural traits of highly curious readers which compel them to delve more deeply into the unfamiliar aspects of the poems.”

23. Lines 488-489 “the positive influence of surprise on creativity...” 'Positive influence' is a very ambiguous and term. Consider rephrasing.
Our response: Thanks for this suggestion. We have now modified the line as: “the influence of surprise on creativity judgment was more pronounced among individuals with lower levels of curiosity.”

24. Lines 494-496: “Firstly, it suggests that emotions such as amazement, wonder, information-seeking curiosity, and knowledge hunger, when experienced at higher levels, have a stronger impact” You did not measure any of those. Why are you extrapolating?
Our response: We agree with your comment. We computed personality traits related to those emotions using self-reports on valid questionnaires. A substantial body of research supports the method of self-reports in personality research (Shiota et al., 2007; Belfi et al., 2018; Hitsuwari & Nomura, 2022; Paulhus & Vazire, 2007; Robins et al., 2007). However, we omitted those lines in the revised manuscript.
25. Please revise the Discussion section with relevant claims and avoid any unwarranted and whimsical claims. Please try to make the discussion more scientific and less poetic.
Our response: The “Discussion” section has been thoroughly revised and modified as suggested.
26. Regarding the lack of effect of 'arousal'. Could it be that participants did not understand what it is supposed to be?
Our response: In the instruction page, for arousal, participants were asked: “How stimulating (higher Scores) or relaxing (lower Scores) did you feel when you read the poem?” (Belfi et al., 2018; Mehl et al., 2023; Hitsuwari & Nomura, 2022; Frame et al., 2023). Thus, we anticipated that participants comprehended the concept of arousal. Of note, despite exhibiting significance ($b = 0.07$, $SE = 0.02$, $t = 4.28$, $p < 0.001$), the inclusion of arousal did not substantially improve the explanatory predictive power of the model which led to its exclusion as a viable predictor (see Model 4 in the Table 3 below for comparison of models). Therefore, the lack of a univariate effect for arousal effect was not the actual reason to exclude arousal as a potential predictor.
Table 3. Model comparison to identify the best model fit comprising aesthetic appeal, felt valence, and surprise.

Note: Aesthetic appeal, felt valence, surprise, arousal and clarity are included sequentially to Model 1 to Model 5; all models are compared hierarchically, i.e., Model 1 is compared to Null Model, Model 2 is compared to Model 1 and so on ; AIC = Akaike Information Criterion; BIC = Bayesian Information Criterion; R^2 = proportion of variation explained by fixed effects (Nakagawa & Schielzeth, 2013); $\Delta\chi2$ = Likelihood ratio test statistic for comparison of models. Significance codes: 0 ‘***’ 0.001 ‘**’ 0.01 ‘*’ 0.05 ‘.’ 0.1 ‘ ’ 1

27. About limitations (from line 524) – please set a new section with title “Limitations”
Our response: Thanks for the suggestion. We set a new section with the title "Limitations” as advised.

Our responses to the Reviewer’s comments

1: Used terminology: Most problematic is, in my view, the use of the term “creativity”. In my opinion, creativity is a state of mind or a quality of people (or – more precisely – a quality of humans that allows them to create something new). Weisberg – for example – defines “creativity” as (a) “the capacity to produce [novel] works [with value]” and (b) “the activity of generating such products” (in: Robert W. Weisberg: Creativity, p. 4). But

it is NOT a property of works of art. However, in the sentence: "... different individuals may hold contrasting views on the creativity of a particular poem ...", creativity is spoken of as if it were an inherent property of poems, not an ability of the person who wrote the poem. Similar is the use of the word throughout the article. This is relevant to the article in that it is not entirely clear what exactly is being measured. Particularly, as the concept and its operationalisation are not explained anywhere. The item used in the questionnaire to assess "creativity" again treats it as an inherent quality of the poems. For a better understanding of the study, it would therefore be imperative to discuss the unconventional use of the term, its implied meaning and its possible impact on the participants of the study. In the literature cited in the manuscript “creativity” is - as far as I know – also seen as a characteristic of people (i.e., the ability of people to be creative). The connection between creativity and basic character traits found in other studies is therefore only indirectly related to this study, if at all. In other studies, various characteristics of an individual were related to each other (e.g., is extroversion related to creativity?). The present study, on the other hand, investigated which character traits of the viewers had an influence on the evaluation of the poems as ... - well, what exactly? I think it is therefore urgent to (a) explain how the term "creativity" is used in the article, (b) critically analyse the relationship between the use of the term in the article and in the cited literature, and (c) discuss in detail the potential effects of its unconventional operationalisation on the results of the study. Note: Despite my reservations, I use the term "creativity" in a similar way as it is used in the manuscript. However, this is simply for lack of alternatives, as I am not quite sure what exactly was measured in the study.

Our response: We would like to thank you for your insightful comments. We understand your concern regarding the operationalization of creativity in our study. Let us provide further clarifications as follows.
The concept of creativity as articulated by Mel Rhodes in 1961, through his 4-P model of creativity, offers a multifaceted perspective. Rhodes described creativity as a phenomenon where an individual (Person) communicates a new concept (Product), underpinned by mental activity (Process) and environmental factors (Press) (Rhodes, 1961). In this model, creativity is defined through four lenses: person, product, process, and press. In our study, we specifically focused on the ‘product’ aspect of creativity, specifically poetry, and operationalized “creativity” as the poem’s creative potential. Of note, our approach is in line with numerous past studies which have investigated the creativity evaluation of various types of products/artefacts, such as ideas (Lloyd-Cox et al., 2022) musical compositions (Pinho et al., 2014; Zioga et al., 2020) short stories (Toivainen et al., 2021) and product concepts (Guenther et al., 2021), to name a few. This approach enabled us to explore the internal model of how individuals judge the creativity of poems, recognizing that such judgments may be inherently subjective and influenced by individual personality traits.
We acknowledge that this operationalization the reviewer has found is different from the more traditional view of creativity as a characteristic of individuals. This choice was made to expand the understanding of creativity beyond the creator to the creation itself. In this study (and in numerous others which have looked at the creativity evaluation of a diverse range of artefacts/ideas), the adopted approach seeks to explore how creativity can also be perceived as an inherent quality of artistic works, in this case, poems. This perspective aligns with our aim to understand how different individuals perceive and evaluate the creativity of a poem based on their personality traits.
Further, we believe that viewing creativity as a characteristic of poems rather than solely a human trait offers a novel insight into how aesthetic appreciation and personality traits interplay in the perception and evaluation of poems. Further, this approach also allowed us to investigate how individual differences in traits like openness, intellect, awe-proneness, and epistemic curiosity influence the creativity evaluation of poems.
In conclusion, our study is a deliberate choice aimed at enriching the understanding of creativity evaluation in the context of poetry. By focusing on the product and the individual’s interaction with it, we aimed to uncover nuanced aspects of how creativity is perceived and evaluated in the realm of English poems.
We deeply acknowledge Reviewer’s comment and included this point in the “Limitations” section as follows:
“Finally, we acknowledge that the operationalization of ‘creativity’ is different from the more traditional view of creativity as a characteristic of individuals. Considering that

creativity, as per the 4P Model (Rhodes, 1961), is defined through four lenses: person, product, process, and press, we strategically focused on the ‘product’ aspect of creativity, particularly in the context of poetry. We, here operationalized ‘creativity’ as the poem’s creative potential. This choice was made to expand the understanding of creativity beyond the creator to the creation itself. Our approach is in line with numerous past studies which have investigated the creativity evaluation of various types of products/artefacts, such as ideas (Lloyd-Cox et al., 2022), musical compositions (Pinho et al., 2014; Zioga et al., 2020) short stories (Toivainen et al., 2021), and product concepts (Guenther et al., 2021), to name a few. This approach enabled us to explore the internal model of how individuals judge the creativity of poems, recognizing that such judgments may be inherently subjective and influenced by individual personality traits.”

2: Operationalisation of the factors: The other variables for recording the aesthetic quality of the poems were also - if I understand correctly - assessed by only one item. Here, too, the question arises as to whether the operationalisation of the question is appropriate. Do the study participants understand what is meant when they have to evaluate whether a poem is "clear"? To what extent does the interpretation of the question vary individually? Are there studies that have examined the validity of these items to capture the underlying concept?

Our response: We appreciate your concern about the operationalization of variables and the potential variability in how study participants interpreted single-item assessments. In our study, participants were asked to evaluate each poem based on the following single-item measures:

1. Clarity: How clear was the content of the poem?
2. Aesthetic appeal: How aesthetically appealing was the poem?
3. Felt valence: How positive (higher scores) or negative (lower scores) did you feel when you read the poem?
4. Felt arousal: How stimulating (higher scores) or relaxing (lower scores) did you feel when you read the poem?
5. Surprise: How Surprising was this poem? By "Surprise" we mean a contrast to expectation in the concept of the poem.
6. Creativity: How Creative was this poem? Please answer based on your intuitive understanding of creativity of poem.

We acknowledge that the individual interpretation of these questions was not explicitly studied in our experiment. However, the use of such single-tem measures is a common practice in the psychometrics of creativity and aesthetics (Lloyd-Cox et al., 2022; Chamberlain et al., 2018; Strijbosch et al., 2022); importantly, single-item measures have also been routinely used in the evaluation of poetry (Belfi et al., 2018; Frame et al., 2023; Mehl et al., 2023; Hitsuwari & Nomura, 2022a, 2022b, 2022c; Papp-Zipernovszky et al., 2021). Nevertheless, we have discussed this issue in the “Limitations” section of the manuscript as follows:

“…we assessed the variables using single-item measures, a common practice in assessing aesthetics in visual art (Hassenzahl, 2004; Chamberlain et al., 2018; Strijbosch et al., 2022), poetry (Belfi et al., 2018; Frame et al., 2023; Mehl et al., 2023; Hitsuwari & Nomura, 2022a, 2022b, 2022c; Papp-Zipernovszky et al., 2021), and music (Zhang & Schubert, 2019). However, we also recognize the potential variability in individual interpretation of the questions remains unexplored. Employing multiple items for variable assessment could have offered psychometric advantages, particularly in enhancing reliability and validity (Bergkvist & Rossiter, 2007).”

3: Selection of poems: The number of poems used for the study is rather small, 36, and - probably - not representative. Especially since the poems were "carefully selected" (by whom, actually?), it is to be feared that the choice of poems might have influenced the result. I understand that this is a necessary sacrifice to make in empirical studies. Especially since the study design almost certainly excludes an influence of the selection of the poems on the result. But I think the issue should at least be discussed as one of the shortcomings of the study. It would also be helpful if it could be shown what measures were taken to ensure that the selection was not biased.

Our response: Many thanks for this comment with which we agree. The chosen set of poems in our study does not constitute a fully representative sample of English poems, which could limit the generalizability of the results to a certain extent. In the revised manuscript, we have now included full details of the poem selection procedure as

follows:
“Initially, we selected 108 poems from various online resources, including Poetry.org (http://www.poetry.org/), the Poetry Foundation (https://www.poetryfoundation.org/), and the Academy of American Poets (https://poets.org/). These poems were subsequently rated for "surprise" on a scale of 1 to 7 by M.D, one of the co-authors who is a Professor of Creative Writing with domain-specific expertise and an award-winning poet. Following this evaluation, we shortlisted 36 as the experimental stimuli: 18 with low surprise ratings (4 or lower) and 18 with high surprise ratings (6 or above). The chosen poems varied in structure, contents, lines, and word count (mean number of lines = 11, SD = 3.24; mean word count = 71.25, SD = 28.99). To represent a broad spectrum of English poems, we consciously chose not to limit our selection to a particular genre or form, like haiku or sonnets as done in previous studies (Belfi et al., 2018; Hitsuwari & Nomura, 2022; Papp-Zipernovszky et al., 2021).”

Minor issues: 1. Line 49: It is not clear to me what you mean by “poetry and its creative evaluation has not been adequately characterized”. First of all, it seems to me that it is not the "creative evaluation" that needs further investigation, but rather the "evaluation of creativity". It also seems that the authors have not considered a whole field of science that deals with this issue of creativity in poems. The authors could look at studies on foregrounding or the field of stylistics. Another research approach that would be relevant in this context is the work of Menninghaus and colleagues.
Our response: Thanks – you are right. We have changed the phrase “creative evaluation” to “evaluation of creativity”.
We appreciate your suggestion to explore foregrounding and the domain of stylistics. In future, conducting studies to investigate the influence of poetry affinity (Kraxenberger & Menninghaus, 2017), or the use of poetic language on various dimensions of emotional responses (e.g., joy, sadness, being moved, intensity, and positive affect), as well as aesthetic appreciation (e.g., beauty, liking, and melodiousness) would be valuable (Menninghaus et al., 2017).

2. Line 50 to 54: It is also not entirely clear to me why the subjectivity of aesthetic evaluation should be more pronounced in poetics than in other forms of art. Personal experience always plays a role in the reception of art, regardless of the medium or art form, doesn't it? Furthermore, it seems to me that the introduction does not clearly separate the concepts of "beauty", "aesthetics" and "creativity". “Beauty” is in the eyes of the beholder. Why then do different observers have different views of the “creativity” with which a poem was written? Are "beauty" and "creativity" the same thing? Or are they causally linked? And if we already know that what is the point of the study?
Our response: We highly appreciate and acknowledge your comment on the fundamental aspect of aesthetics. As mentioned in the article, the main focus of the study is to investigate how individual difference in specific personality traits, specifically Openness, Intellect, Awe-proneness, and Epistemic Curiosity, shape the implicit associations between subjective experiences and the judgment of creativity of English poems.
While we acknowledge the importance of the age-old questions mentioned by the reviewer, we have purposefully avoided answering them as they are beyond the scope of this current study.

3. Line 62-64: In much of the introduction, the authors make assertions without citing any valid references. For example, I do not understand why "creativity" should be a less tangible construct than "preference".
Our response: We acknowledge your comment. We omitted the lines in the revised manuscript.

4. Line 79 (and others): Some sentences end without a period or have commas before the period (line 102).
Our response: Thanks. We have corrected as appropriate.

5. Line 457-467: In the discussion, an indirect connection is made between the ambiguity of expression in a poem and aesthetic appeal via the evaluation of "awe-proneness". It is stated that "other studies" have reported a connection between "awe-proneness" and the ability to tolerate ambiguity. But, on the one hand, this seems to concern only one study, and on the other hand, this is an indirectly established connection. I therefore think it is appropriate to formulate the conclusion (which I do not

want to doubt) more cautiously (lines 465 and 466). In order to substantiate the thesis a little, it might be helpful to support it a little with findings from the studies on "foregrounding" (British stylistics or Russian formalism).

Our response: We acknowledge your valuable advice. We have now excluded the lines reporting a connection between awe-proneness and ambiguity tolerance.

References:

Acar, S., Burnett, C., & Cabra, J. F. (2017). Ingredients of creativity: Originality and more. Creativity Research Journal, 29(2), 133–144. https://doi.org/10.1080/10400419.2017.1302776

Barron, F. (1955). The disposition toward originality. The Journal of Abnormal and Social Psychology, 51, 478–485. https://doi.org/10.1037/h0048073

Belfi, A. M., Vessel, E. A., & Starr, G. G. (2018). Individual ratings of vividness predict aesthetic appeal in poetry. Psychology of Aesthetics, Creativity, and the Arts, 12(3), 341–350. https://doi.org/doi:10.1037/aca0000153

Bergkvist, L., & Rossiter, J. R. (2007). The predictive validity of multiple-item versus single-item measures of the same constructs. Journal of Marketing Research, 44(2), 175–184. https://doi.org/https://doi.org/10.1509/jmkr.44.2.175

Berlyne, D. E. (1954). A theory of human curiosity. British Journal of Psychology, 45(3), 180.

Chamberlain, R., Mullin, C., Scheerlinck, B., & Wagemans, J. (2018). Putting the art in artificial: Aesthetic responses to computer-generated art. Psychology of Aesthetics, Creativity, and the Arts, 12(2), 177. https://doi.org/https://psycnet.apa.org/doi/10.1037/aca0000136

Chamorro-Premuzic, T., & Furnham, A. (2004). Art judgment: a measure related to both personality and intelligence? Imagination, Cognition and Personality, 24(1), 3–24. https://doi.org/https://doi.org/10.2190/U4LW-TH9X-80M3-NJ54

Chamorro-Premuzic, T., Reimers, S., Hsu, A., & Ahmetoglu, G. (2009). Who art thou? Personality predictors of artistic preferences in a large UK sample: The importance of openness. British Journal of Psychology, 100(3), 501–516. https://doi.org/https://doi.org/10.1348/000712608X366867

Chotlos, J. W. (1944). IV. A statistical and comparative analysis of individual written language samples. Psychological Monographs, 56(2), 75. https://doi.org/https://psycnet.apa.org/doi/10.1037/h0093511

Collins, R. P., Litman, J. A., & Spielberger, C. D. (2004). The measurement of perceptual curiosity. Personality and Individual Differences, 36(5), 1127–1141. https://doi.org/https://doi.org/10.1016/S0191-8869(03)00205-8

Costa Jr, P. T., & McCrae, R. R. (1992). Four ways five factors are basic. Personality and Individual Differences, 13(6), 653–665. https://doi.org/https://doi.org/10.1016/0191-8869(92)90236-I

Costa, P. T., & McCrae, R. R. (2008). The revised neo personality inventory (neo-pi-r). The SAGE Handbook of Personality Theory and Assessment, 2(2), 179–198.

DeYoung, C. G., Shamosh, N. A., Green, A. E., Braver, T. S., & Gray, J. R. (2009). Intellect as distinct from openness: Differences revealed by fMRI of working memory. Ournal of Personality and Social Psychology, 97(5), 883–892. https://doi.org/https://psycnet.apa.org/doi/10.1037/a0016615

DeYoung, C. G., Grazioplene, R. G., & Peterson, J. B. (2012). From madness to genius: The Openness/Intellect trait domain as a paradoxical simplex. Journal of Research in Personality, 46(1), 63–78. https://doi.org/https://doi.org/10.1016/j.jrp.2011.12.003

DeYoung, C. G., Peterson, J. B., & Higgins, D. M. (2005). Sources of openness/intellect: Cognitive and neuropsychological correlates of the fifth factor of personality. Journal of Personality, 73(4), 825–858. https://doi.org/https://doi.org/10.1111/j.1467-6494.2005.00330.x

DeYoung, C. G., Quilty, L. C., & Peterson, J. B. (2007). Between facets and domains: 10 aspects of the Big Five. Journal of Personality and Social Psychology, 93(5), 880.

DeYoung, C. G., Quilty, L. C., Peterson, J. B., & Gray, J. R. (2014). Openness to Experience, Intellect, and Cognitive Ability. Journal of Personality Assessment, 96(1), 46–52. https://doi.org/10.1080/00223891.2013.806327

Faul, F., Erdfelder, E., Lang, A.-G., & Buchner, A. (2007). G* Power 3: A flexible statistical power analysis program for the social, behavioral, and biomedical sciences. Behavior Research Methods, 39(2), 175–191.

Frame, J., Mehl, K., Head, K., & Belfi, A. (2023). The influence of sensory modality on aesthetic judgments of poetry. https://doi.org/https://doi.org/10.31234/osf.io/cv6px

Gabrielsson, A. (2001). Emotion perceived and emotion felt: Same or different? Musicae Scientiae, 5(1_suppl), 123–147. https://doi.org/10.1177/10298649020050S105
Guenther, A., Eisenbart, B., & Dong, A. (2021). Creativity and successful product concept selection for innovation. International Journal of Design Creativity and Innovation, 9(1), 3–19. https://doi.org/https://doi.org/10.1080/21650349.2020.1858970
Hassenzahl, M. (2004). The interplay of beauty, goodness, and usability in interactive products. Human–Computer Interaction, 19(4), 319–349. https://doi.org/https://doi.org/10.1207/s15327051hci1904_2
Hitsuwari, J., & Nomura, M. (2022). How individual states and traits predict aesthetic appreciation of haiku poetry. Empirical Studies of the Arts, 40(1), 81–99. https://doi.org/10.1177/0276237420986420
Jacobs, A. M. (2017). Quantifying the beauty of words: A neurocognitive poetics perspective. In Frontiers in Human Neuroscience (Vol. 11). https://www.frontiersin.org/articles/10.3389/fnhum.2017.00622
Jacobs, A. M., & Kinder, A. (2018). What makes a metaphor literary? Answers from two computational studies. Metaphor and Symbol, 33(2), 85–100.
Johnson, D. R., Kaufman, J. C., Baker, B. S., Patterson, J. D., Barbot, B., Green, A. E., van Hell, J., Kennedy, E., Sullivan, G. F., Taylor, C. L., Ward, T., & Beaty, R. E. (2022). Divergent semantic integration (DSI): Extracting creativity from narratives with distributional semantic modeling. Behavior Research Methods. https://doi.org/10.3758/s13428-022-01986-2
Kraxenberger, M., & Menninghaus, W. (2017). Affinity for poetry and aesthetic appreciation of joyful and sad poems. In Frontiers in Psychology (Vol. 7). https://www.frontiersin.org/articles/10.3389/fpsyg.2016.02051
Kuiken, D., Douglas, S., & Kuijpers, M. (2021). Openness to experience, absorption-like states, and the aesthetic, explanatory, and pragmatic effects of literary reading. Scientific Study of Literature, 11(2), 148–195. https://doi.org/https://doi.org/10.1075/ssol.21007.kui
Lau, J. H., Cohn, T., Baldwin, T., Brooke, J., & Hammond, A. (2018). Deep-speare: A joint neural model of poetic language, meter and rhyme. https://doi.org/10.48550/arxiv.1807.03491
Litman, J. A., & Spielberger, C. D. (2003). Measuring epistemic curiosity and its diversive and specific components. Journal of Personality Assessment, 80(1), 75–86. https://doi.org/10.1207/S15327752JPA8001_16
Litman, J., Hutchins, T., & Russon, R. (2005). Epistemic curiosity, feeling-of-knowing, and exploratory behaviour. Cognition and Emotion, 19(4), 559–582. https://doi.org/10.1080/02699930441000427
Lloyd-Cox, J., Pickering, A., & Bhattacharya, J. (2022). Evaluating creativity: How idea context and rater personality affect considerations of novelty and usefulness. Creativity Research Journal, 34(4), 373–390. https://doi.org/10.1080/10400419.2022.2125721
Loewenstein, G. (1994). The psychology of curiosity: A review and reinterpretation. Psychological Bulletin, 116(1), 75.
Malvern, D. D., & Richards, B. J. (1997). A new measure of lexical diversity. British Studies in Applied Linguistics, 12, 58–71.
Marin, M. M., & Bhattacharya, J. (2010). Music induced emotions: some current issues and cross-modal comparisons. In J. Hermida & M. Ferrero (Eds.), Music education (pp. 1–38). Hauppauge, NY: Nova Science Publishers.
Mastria, S., Agnoli, S., & Corazza, G. E. (2019). How does emotion influence the creativity evaluation of exogenous alternative ideas? PLOS ONE, 14(7), e0219298. https://doi.org/10.1371/journal.pone.0219298
McCarthy, P. M., & Jarvis, S. (2007). vocd: A theoretical and empirical evaluation. Language Testing, 24(4), 459–488. https://doi.org/https://doi.or...

Additional Information:	
**Question**	**Response**
**Financial Disclosure**<br><br>Enter a financial disclosure statement that describes the sources of funding for the work included in this submission. Review the submission guidelines for detailed	The author(s) received no specific funding for this work.

requirements. View published research articles from *PLOS ONE* for specific examples.<br><br>This statement is required for submission and **will appear in the published article** if the submission is accepted. Please make sure it is accurate.<br><br>**Unfunded studies**<br>Enter: *The author(s) received no specific funding for this work.*<br><br>**Funded studies**<br>Enter a statement with the following details:<br>• Initials of the authors who received each award<br>• Grant numbers awarded to each author<br>• The full name of each funder<br>• URL of each funder website<br>• Did the sponsors or funders play any role in the study design, data collection and analysis, decision to publish, or preparation of the manuscript?<br>• **NO** - Include this sentence at the end of your statement: *The funders had no role in study design, data collection and analysis, decision to publish, or preparation of the manuscript.*<br>• **YES** - Specify the role(s) played.<br><br>* typeset	
**Competing Interests**<br><br>Use the instructions below to enter a competing interest statement for this submission. On behalf of all authors, disclose any competing interests that could be perceived to bias this work—acknowledging all financial support and any other relevant financial or non-financial competing interests.<br><br>This statement is required for submission and **will appear in the published article** if the submission is accepted. Please make sure it is accurate and that any funding sources listed in your Funding Information later in the submission form are also declared in your Financial Disclosure	The authors have declared that no competing interests exist.

statement.<br><br>View published research articles from *PLOS ONE* for specific examples.<br><br>**NO authors have competing interests**<br><br>Enter: *The authors have declared that no competing interests exist.*<br><br>**Authors with competing interests**<br><br>Enter competing interest details beginning with this statement:<br><br>*I have read the journal's policy and the authors of this manuscript have the following competing interests: [insert competing interests here]*<br><br>* typeset	
**Ethics Statement**<br><br>Enter an ethics statement for this submission. This statement is required if the study involved:<br><br>• Human participants<br>• Human specimens or tissue<br>• Vertebrate animals or cephalopods<br>• Vertebrate embryos or tissues<br>• Field research<br><br>Write "N/A" if the submission does not require an ethics statement.<br><br>General guidance is provided below. Consult the submission guidelines for detailed instructions. **Make sure that all information entered here is included in the Methods section of the manuscript.**	The study protocol was approved by the local Ethics Committee of the Department of Psychology, Goldsmiths University of London.<br><br>Reference Number: psy27012022SC<br><br>We described the main experimental procedures for this research to the participants. They were also informed that their data will be treated with full confidentiality (the study complied with the General Data Protection Regulation), and that, if published, it would not be identifiable as theirs. All participants provided informed consent (online) prior to data collection.<br>(This has been mentioned in the "Methods" section in the manuscript)<br><br>We also mentioned that participants may withdraw from the research at any time and for any reason. We also debriefed participants at the end of their participation.<br><br>The URL of our Ethics Approval Form is as follows: (Uploaded)<br><br>https://psy770.gold.ac.uk/student_ethics_student/view_ethics_form.php?form_id=1575

**Format for specific study types**<br><br>**Human Subject Research (involving human participants and/or tissue)**<br>• Give the name of the institutional review board or ethics committee that approved the study<br>• Include the approval number and/or a statement indicating approval of this research<br>• Indicate the form of consent obtained (written/oral) or the reason that consent was not obtained (e.g. the data were analyzed anonymously)<br><br>**Animal Research (involving vertebrate animals, embryos or tissues)**<br>• Provide the name of the Institutional Animal Care and Use Committee (IACUC) or other relevant ethics board that reviewed the study protocol, and indicate whether they approved this research or granted a formal waiver of ethical approval<br>• Include an approval number if one was obtained<br>• If the study involved *non-human primates*, add *additional details* about animal welfare and steps taken to ameliorate suffering<br>• If anesthesia, euthanasia, or any kind of animal sacrifice is part of the study, include briefly which substances and/or methods were applied<br><br>**Field Research**<br><br>Include the following details if this study involves the collection of plant, animal, or other materials from a natural setting:<br>• Field permit number<br>• Name of the institution or relevant body that granted permission	
**Data Availability**<br><br>Authors are required to make all data underlying the findings described fully available, without restriction, and from the time of publication. PLOS allows rare exceptions to address legal and ethical concerns. See the PLOS Data Policy and FAQ for detailed information.	Yes - all data are fully available without restriction

Powered by Editorial Manager® and ProduXion Manager® from Aries Systems Corporation

A Data Availability Statement describing where the data can be found is required at submission. Your answers to this question constitute the Data Availability Statement and **will be published in the article**, if accepted.<br><br>**Important:** Stating 'data available on request from the author' is not sufficient. If your data are only available upon request, select 'No' for the first question and explain your exceptional situation in the text box.<br><br>Do the authors confirm that all data underlying the findings described in their manuscript are fully available without restriction?	
**Describe where the data may be found in full sentences. If you are copying our sample text, replace any instances of XXX with the appropriate details.**<br><br>• If the data are **held or will be held in a public repository**, include URLs, accession numbers or DOIs. If this information will only be available after acceptance, indicate this by ticking the box below. For example: *All XXX files are available from the XXX database (accession number(s) XXX, XXX.*).<br>• If the data are all contained **within the manuscript and/or Supporting Information files**, enter the following: *All relevant data are within the manuscript and its Supporting Information files.*<br>• If neither of these applies but you are able to provide **details of access elsewhere**, with or without limitations, please do so. For example:<br><br>*Data cannot be shared publicly because of [XXX]. Data are available from the XXX Institutional Data Access / Ethics Committee (contact via XXX) for researchers who meet the criteria for access to confidential data.*<br><br>*The data underlying the results presented in the study are available from (include the name of the third party*	The Open Science Framework (OSF):<br><br>https://osf.io/9mw7r/?view_only=07137f4871d146c790501f22bc7743d5

and contact information or URL).<br>• This text is appropriate if the data are owned by a third party and authors do not have permission to share the data.<br><br>* typeset	
Additional data availability information:	

Revised Manuscript
Click here to access/download;Manuscript;Revised Manuscript_Clean.docx

Beyond the words: Exploring individual differences in the evaluation of poetic creativity

Soma Chaudhuri[1], Alan Pickering[1], Maura Dooley[2], and Joydeep Bhattacharya[1]

[1]Department of Psychology, Goldsmiths, University of London

[2]Department of English and Creative Writing, Goldsmiths, University of London

*Corresponding author

Email: schau002@gold.ac.uk

Author Contributions

Conceptualization: Soma Chaudhuri, Joydeep Bhattacharya.

Data curation: Soma Chaudhuri.

Formal analysis: Soma Chaudhuri.

Methodology: Soma Chaudhuri, Alan Pickering, Joydeep Bhattacharya.

Supervision: Joydeep Bhattacharya.

Writing – original draft: Soma Chaudhuri

Writing - review and editing: Joydeep Bhattacharya, Alan Pickering, Maura Dooley

Authors have no known conflict of interest to disclose.

Abstract

Poetry is arguably the most creative expression of language that can evoke diverse subjective experiences, such as emotions and aesthetic responses, in different individuals, subsequently influencing their judgment of the creativity of poem. This study investigated how personality traits, specifically openness, intellect, awe-proneness, and epistemic curiosity, shape the implicit associations between subjective experiences and the judgment of creativity of thirty-six English language poems. One hundred and twenty-nine participants rated each poem across six dimensions: clarity, aesthetic appeal, felt valence, felt arousal, surprise, and overall creativity. First, we obtained a parsimonious model of creativity comprising aesthetic appeal, felt valence, and surprise as potential predictors of poetic creativity. Subsequently, using multilevel analysis, we investigated the interactions between the four personality traits and these three predictors. Among the personality traits, openness emerged as the primary moderator in predicting judgments of poetic creativity, followed by curiosity and awe-proneness. Among the predictors, aesthetic appeal was moderated by all four personality traits, while surprise was moderated by openness, awe-proneness, and curiosity. Valence, on the other hand, was moderated by openness only. Our findings provide novel insights into how individual differences influence the evaluation of the creativity of poems.

Keywords: poetry, creativity, evaluation, personality traits, moderation

Introduction

Poetry is one of the most creative forms of linguistic expression used since ancient times to communicate emotions, thoughts, and ideas. However, despite its unique status in human lives, evaluation of creativity of poems has not been adequately characterized. The inherent subjectivity of poetry as an abstract form of literary art may be a possible reason for this limitation. The adage "Beauty is in the eye of the beholder," (sourced from Margaret Wolfe Hungerford's novel "Molly Bawn" (1878)), aptly captures the subjective nature of aesthetics which also holds true for poetry. Individuals may possess divergent perspectives on creative potential of a particular poem, influenced by their subjective experiences. Readers comprehend the same poem differently depending on their knowledge and perceptual ability which leads to an idiosyncrasy in the assessment of its creativity. What one person perceives as creative, and captivating may be ordinary or unappealing to another. Therefore, individual differences in personality traits of readers are likely to cause variations in their assessments, subsequently influencing their overall creativity judgment of poetry. This study investigated how readers' internal models based on their personality attributes would impact their subjective feelings and experiences of reading poems while assessing poetic creativity.

Personality traits are basic dimensions on which people differ, reflecting their characteristic patterns of thoughts, feelings, and behaviours with consistency and stability (1,2). Several studies (3,4,5,6) have investigated the link between personality traits and creativity. Significant positive correlations have been observed between different measures of creativity and Big Five personality traits (7,8,9), especially openness to experience and extraversion (10,11,12). A meta-analysis (4) found openness to experience and extraversion to be the most prominent personality traits associated with creativity in the Arts and Sciences.

When assessing creativity, raters often form their own mental criteria, which can vary depending on their personal preferences and individual differences (13). For example, recent research (14) suggests that openness to experience is associated with a higher discernment ability to distinguish creative from uncreative responses. Another study (15) highlights how an individual's consideration of the novelty and usefulness of creativity task responses is influenced by contextual factors and individual differences, such as openness and intellect, in overall creativity judgment. These studies focused primarily on the relationship between personality traits and various creative idea-generation processes, such as divergent thinking, everyday creative behaviour, creative achievement, and self-rated creativity. However, the influence of personality traits on the evaluation of creativity of poetry has not been adequately explored. Of note, some studies (16,17,18) have found that individual differences in visual imagery abilities, ambiguity tolerance, awe-proneness, and nostalgia-proneness predict the aesthetic appeal of specific forms of poems like haiku and sonnets. In the present study, we addressed how readers' personality traits interact with predictors selected from five factors - clarity, aesthetic appeal, felt valence, arousal, and surprise – in evaluating the creativity of English language poems. Specifically, we focused on four personality traits – openness, intellect, awe-proneness, and epistemic curiosity. We examined the moderating effects of these traits on the factors that predict the creativity judgment of a poem. In the subsequent sections, we provide a brief overview of these potential predictors, the personality traits themselves, and their prospective roles in evaluating creativity.

Clarity refers to whether the text is lucid, understandable, and comprehensible to the readers. Any valued written communication, like poetry, is functional when readers comprehend it; clarity is a relevant dimension for evaluating the creativity of a poem (19).

Aesthetic appeal refers to the artistic features, styles, and concepts of any artwork.. Research on the psychology of creativity and aesthetics has utilised stimuli like paintings and visual art (20,21,22,23,24), music (25, 26,27,28,29,30,31), films (32,33,34), poems (16,17,18,35,36,37,38,39). Previous empirical studies on poetry have primarily investigated aesthetic appreciation focusing on two broad aspects: (i) the objective properties of a poem and (ii) the subjective experiences during the perception of a poem. The former approach examines textual elements, e.g., rhythm, rhyme, meter (36, 40), metaphors (35, 41,42,43), and phonological constructs such as words and phrases (44, 45). The latter approach explores empathic reactions and emotional involvement (46), perceived emotional valence and vividness in imagery (16) cognitive and emotional ambiguity (e.g., awe and nostalgia) (38,17), openness to experience, visual imagery abilities, felt valence (17), expertise (47), gender and ethnicity (48). However, the potential interactions between these two approaches and how readers' characteristics influence their subjective evaluation of creativity remain unclear.

Felt valence refers to the extent to which an emotion felt by the perceiver is positive or negative, whereas arousal refers to its intensity, i.e., the strength of the associated emotional state. **According to the two-dimensional circumplex model of emotion, emotional states are represented along two orthogonal dimensions: valence (pleasure-displeasure: horizontal axis) and arousal (degree of arousal: vertical axis) (49). Poetry evokes strong emotions (50) and emotional states can influence creativity evaluation (51). A recent study suggests that poetry's content and prosodic features can evoke basic emotions, while the reader's intellectual evaluation of a poem evokes a complex aesthetic emotion that combines a basic emotion with their assessment of the poem (52). Of note, perceived and felt emotions may be distinct (53,54). Perceived emotions are the emotions evoked by the stimuli whereas felt emotions are the emotions felt by**

the perceivers. In our study, we focused on the felt emotions, i.e., the emotions felt by the reader while reading the poem, rather than the perceived emotion, i.e., the emotion recognized in the poem. Therefore, felt valence reveals the extent to which the readers felt positive or negative emotions while reading the poems, whereas felt arousal here reveals how intense it was felt by the readers.

Surprise is usually a short-lived emotion elicited by events that deviate from an established schema (55, 56, 57). Of note, a schema, in this sense, refers to a component of the organism's knowledge structure, activated by a specific stimulus (58). Surprise is recognized as a key predictor of creativity (59,60) and is also a robust predictor of aesthetic judgment of artwork (61). In the context of our study, the term "surprise" specifically denotes the degree to which there is a sudden and unexpected change in the context or theme of the poem.

Openness and Intellect

Openness to experience is a broad range of traits, from intellectual abilities to aesthetic and artistic interests (62,63,64) and is most robustly associated with measures of creativity (65). It influences vivid fantasy (66), artistic sensitivity, novelty in artworks, aesthetic emotions (67), intellectual curiosity (68), and unconventional attitudes (65). openness/intellect, though characterized as a single dimension of personality, can be differentiated into two major aspects: openness and intellect (69). Openness reflects the tendency to engage with aesthetic and sensory information, both in perception and imagination. On the other hand, intellect is a dispositional individual difference variable related to intellectual performance, such as problem-solving, thinking, information search, learning, or creativity (62, 70).Therefore, we predicted that openness and intellect would separately moderate the relationship between aesthetic appeal and creativity ratings of a

poem. Specifically, we predicted that individuals with higher levels of openness would experience a stronger positive impact of aesthetic appeal on creativity ratings compared to those with lower levels of openness.

Individuals with higher openness are reported to be more sensitive and attuned to their feelings (71). However, excessive emotional processes can inhibit higher cognitive functions in individuals with higher openness (72). Therefore, we predicted that openness would moderate the relationship between felt emotions (both valence and arousal) and creativity. Specifically, individuals with higher levels of openness would perceive the positive impact of felt emotions on creativity rating as less significant compared to those with lower levels of openness. Acknowledging that intellect is related to processing of complex and intellectual information (7,73), we assume that individuals with high intellect might rely less on emotional cues in their evaluation. Therefore, our prediction was~~,~~ intellect would not moderate the relationship between felt emotions and creativity. In other words, the positive impact of aesthetic appeal on creativity would remain unaffected by the levels of intellect in individuals.

Surprise, interpreted as a form of conflict, arises in response to "collative variables" like novelty, complexity, uncertainty, and conflict (74). **As surprise is often experienced when individuals encounter unexpected or schema-discrepant events** (55,56)**, we assumed that the assessment of expectancy violations in the judgment of poetic creativity necessitates a substantial degree of cognitive engagement. Consequently, we predicted that both openness and intellect would moderate the relationship between surprise and creativity**. Our specific prediction was that the positive impact of surprise on creativity scores would be more pronounced in individuals with higher openness and intellect. These individuals may be more receptive to and intrigued by unexpected elements

of a poem, leading to heightened creativity judgment in response to surprises encountered in poems.

Awe-proneness

Awe, classified as an epistemic emotion, is a distinct emotional response to perceiving a stimulus that is vast, both literally and figuratively, and requires cognitive accommodation (75). Poetry is likely to elicit awe due to its rich information content (76). Dispositional awe-proneness is significantly correlated with openness to experience (*r* = .49), indicating a link between awe-proneness and the tendency to push one's intellectual boundaries (76). Further, higher dispositional awe has been associated with aesthetic engagement and a tendency to experience aesthetic chill (77). **Aesthetic chills are transient emotional responses to aesthetical stimuli** (78)**. They can be described as experiencing a chill or wave of excitement when engaging with poetry or looking at a work of art** (79). Since awe is linked to surprise and amazement and is interpreted as a passive, receptive mode of attention in response to the unexpected (80,75) , we predicted that the awe-proneness would moderate the effect of aesthetic appeal and surprise on creativity scores of a poem. Specifically, we predicted that the impact of aesthetic appeal and surprise on creativity ratings would be much stronger in individuals with higher levels of awe-proneness. Due to their disposition towards experiencing awe, these individuals may be more receptive and responsive to a poem's aesthetic qualities and unexpected elements, thereby attributing higher levels of creativity to such poems.

Epistemic Curiosity

Curiosity is an intense desire to explore novel, complex and uncertain events (81). It is associated with learning and thinking processes and linked to various constructs such as interest, surprise, confusion, and awe (82,83). Curiosity can be categorized into two broad

types: perceptual curiosity and epistemic curiosity ; where perceptual curiosity leads to increased perception of stimuli, and epistemic curiosity is defined as a "drive to know" (84). Epistemic curiosity motivates individuals to engage in exploratory behaviours to bridge the gap between their existing knowledge and their desire for further understanding (85,86,87). Also, highly open individuals tend to be curious about the world, (88,89,90,91) Therefore, we predicted that epistemic curiosity would significantly moderate the relationship between aesthetic appeal, surprise, and creativity. Specifically, we predicted that the positive impact of aesthetic appeal and surprise on creativity scores would be more pronounced in individuals with higher levels of epistemic curiosity. These individuals, driven by their curiosity, would be more inclined to appreciate the aesthetic qualities and unexpected elements in a poem, thus attributing higher levels of creativity to such poems.

Materials and methods

Materials

Initially, we selected 108 poems from various online resources, including the Poetry.org (http://www.poetry.org/), the Poetry Foundation (https://www.poetryfoundation.org/), and the Academy of American Poets (https://poets.org/). These poems were subsequently rated for "surprise" on a scale of 1 to 7 by M.D, who is a Professor of English and Creative Writing with domain-specific expertise. Following this evaluation, we shortlisted 36 poems as the experimental stimuli: 18 with low surprise ratings (4 or lower) and 18 with high surprise ratings (6 or above). The chosen poems varied in structures, contents, lines, and word count (mean number of lines = 11, SD = 3.24; mean word count = 71.25, SD = 28.99). To represent a

broad spectrum of English poems, we consciously chose not to limit our selection to a particular genre or form, like haiku or sonnets as done in previous studies (16, 17, 92).

Lexical diversity (LD) of a text refers to its lexical richness, indicating the range and variety of vocabulary deployed in a text (93). For computing the LD of the poems, we used the type-token ratio (TTR) method which calculates the ratio of unique words (types) to the total word count (tokens) (94). It ranges from 0 to 1: a higher TTR indicates a greater lexical diversity. The average lexical diversity across the poems is 0.77 with ~~standard a~~ deviation of 0.09, suggesting that, on average, about 77% of the words used in the poems are unique or different. Semantic diversity, on the other hand, refers to the range of contexts (i.e., semantic richness) in which words are used (95). To calculate the semantic diversity, we used divergent semantic integration (DSI) (http://semdis.wlu.psu.edu/), representing the mean semantic distance between all word pairs in a text; DSI varies from 0 to 1, where a higher score indicates a broader collection of divergent ideas. The average DSI of our stimuli is 0.80 with a standard deviation of 0.03, indicating a high degree of semantic variety (see Table S1 in the Supplementary section for details).

Participants

By using the G*Power software (v. 3.1.9.4), (96) we found that a minimum sample size of 98 was required to detect a medium effect size ($f^2 = 0.15$) in a multiple linear regression, assuming a significance level of 0.05 and a statistical power of 80%. The criteria we used are widely-used conventional figures when estimating sample sizes. Additionally, we used the ‘samplesize_mixed’ function in R, which estimates the required sample size for linear mixed models (two-level-designs), based on power-

calculation for standard design and adjusted for design effect for 2-level-designs (https://strengejacke.github.io/sjstats/). For our multilevel model, which considered 96 cluster groups, and a small to medium effect size (Cohen's d = 0.3), with 36 observations per cluster group (level-2-unit), we determined that a total of 965 observations would be necessary. This translates to a minimum of 27 participants (965/36).

We recruited 129 adult participants via Prolific®, a participant-recruiting platform. As the task lasted approximately one hour, we excluded 30 participants who exceeded a two-hour time limit. Additionally, three participants were eliminated from the analyses due to their identical responses on the subjective rating measures across the poems. Our final sample consisted of 96 participants resulting in a total of 3456 observations, ensuring sufficient statistical power for our study. Participants (32 males, 63 females, 1 preferred not to say; mean age = 31.94 years, SD = 13.09) were fluent in English (self-reported) and from a variety of educational backgrounds holding at least a bachelor's degree in any discipline. Additionally, our sample represented a diverse range of professions, and ethnicities.

Participants were briefed about the experimental procedure, which involved the assessment of a set of English poems on a 7-point Likert scale (1 = extremely low; 7 = extremely high) across various constructs including clarity, aesthetic appeal, felt valence, arousal, surprise, and overall creativity. Additionally, participants were instructed to complete demographic and personality-related questions. We assured participants of the full confidentiality of their data, in compliance with the General Data Protection Regulation, and clarified that any published results would be non-identifiable. All participants provided informed consent (online) before ~~to~~ data collection. Participants received a cash incentive of £7.50 per hour. The data collection

period spanned from 27 January 2022 to 23 June 2022, and the data were accessed for research purposes only after this period.

Procedure

The experiment was created using Qualtrics®, and the link was disseminated through Prolific®, a platform for participant recruitment. Participants received a broad overview of the study and comprehensive instructions for ratings. In the beginning, a sample poem was provided to facilitate a clearer understanding of the process. **Participants were given a minimum of 30 seconds to read each poem. Following this period, they were allowed to proceed to the rating task. They were asked to rate the poems on six dimensions in the following order: clarity, aesthetic appeal, felt valence, felt arousal, surprise, and creativity, using a 7-point Likert scale (1: "Extremely Low" and 7: "Extremely High"). There was no time limit imposed for rating the poems. Of note, the poems remained visible during the rating process.** A brief demographic survey was conducted once 36 trials were finished. Finally, participants completed a set of questions onpersonality traits – Ten Item Personality Inventory (TIPI: (97), openness/intellect (69), awe-proneness (76), and epistemic curiosity (86). It took an hour on average to finish the whole experiment. The study protocol was approved by the local Ethics Committee of the Department of Psychology, Goldsmiths University of London.

Analysis

The primary aim of our study was to explore how four personality traits—openness, intellect, awe-proneness, and epistemic curiosity—moderate the impact of significant potential predictors on poetic creativity. First, we determined the significant predictors of the creativity of poems. To accomplish this task, five maximum likelihood linear mixed models

(predictor models) were executed using the *lme4* package (98) in R (version 4.0.3). The models were run hierarchically, with the five potential predictors (group mean centered) progressively included as fixed effects, creativity as the response variable, and participants as the grouping variable. We incorporated random effects intercepts for participants in the analysis. The best model fit results identified the potential predictors of poetic creativity.

The overall data visualisation confirmed that the response variable follows a normal distribution, and there is no significant multicollinearity among the independent variables (Variance Inflation Factor < 3). Furthermore, the reliability of the measurement was established by assessing the internal consistency across items (Cronbach's alpha = 0.80; McDonald's omega (Total) = 0.88).

The dataset comprised 3456 responses and exhibited a common multilevel structure, with individual responses (Level-1) nested within participants (Level-2). The null model revealed that a significant 54% of the variance was attributed to the grouping variable (participants), affirming the necessity of employing a linear mixed model to accommodate the hierarchical nature of the data, over standard regression models. Furthermore, the intraclass correlation coefficient (ICC = 0.28), signifying the Level-2 clustering, revealed a significant level of clustering in the data. This implies that the Level-1 dependent variable (creativity) was not independent of the Level-2 grouping variable (participants). Hence, the use of multilevel modeling was considered appropriate.

To accurately estimate the within-group effects, the predictors were centered within clusters (CWC) before entering the models (99). Finally, we examined the impact of four personality traits (e.g., openness, intellect, awe-proneness, and epistemic curiosity) on potential predictors by conducting four separate linear mixed models (personality traits models). In these models, the personality traits and their interactions with the potential

predictors were treated as fixed effects, with creativity as the response variable and participants as the grouping variable. To visualize the interaction effects of the moderators on the predictors, we followed the classical convention (100). Specifically, we plotted the mean value of the moderator and one standard deviation above and below the mean, allowing us to observe how the moderator influences the relationship between the predictors and creativity.

Results

Descriptive statistics

Descriptive statistics of the variables are shown in Table 1, including the mean and standard deviation (SD) for each variable. The variables include five predictors (clarity, aesthetic appeal, felt valence, felt arousal, surprise), four personality traits (openness, intellect, awe-proneness, and epistemic curiosity), and the outcome variable (creativity). Distribution of variables are marginally left-skewed (excepting openness with skewness of 0.12), with very low kurtosis values. Variation Inflation Factor (VIF<3) confirms the absence of multicollinearity among the variables. Of note, throughout the article, epistemic curiosity is referred to as curiosity for the sake of clarity and ease of comprehension.

Table 1. Descriptive statistics of the variables including mean, standard deviation, skewness, kurtosis, standard error, and variance inflation factor (VIF).

Variable	n	mean	sd	median	min	max	skewness	kurtosis	se	VIF
Clarity	3456	4.82	1.58	5	1	7	-0.46	-0.57	0.03	1.59
Aesthetic Appeal	3456	4.8	1.44	5	1	7	-0.48	-0.23	0.02	2.2
Felt Valence	3456	4.5	1.62	5	1	7	-0.41	-0.48	0.03	2.62
Felt Arousal	3456	3.86	1.73	4	1	7	-0.14	-0.92	0.03	2.01
Surprise	3456	3.78	1.68	4	1	7	-0.17	-0.92	0.03	1.69
Creativity	3456	4.91	1.38	5	1	7	-0.53	0.05	0.02	-
Openness	3456	5.02	0.74	4.9	3	6.4	0.12	-0.82	0.01	1.49
Intellect	3456	4.7	0.9	4.7	2.7	6.4	-0.04	-0.59	0.02	1.48
Awe-proneness	3456	5.11	1.14	5.17	1.83	7	-0.48	-0.04	0.02	1.76
Curiosity	3456	5.58	0.86	5.6	3.5	7	-0.19	-0.67	0.01	1.71

Table 2 shows the bivariate correlations between the predictors, personality traits, and creativity. Creativity was positively and significantly (all *p*<.01) correlated with five predictor variables: clarity (*r* = 0.42), aesthetic appeal (*r* = 0.66), felt valence (*r* = 0.60), arousal (*r* = 0.47), surprise (*r* = 0.52). Creativity was also significantly correlated (all *p*<.01), albeit ~~weaker~~, with four personality traits: openness (*r* = 0.17), intellect (*r* = 0.17), awe-proneness (*r* = 0.20), and curiosity (*r* =0.22). No significant correlation was observed between openness and arousal (*r* = 0 .02, *p* = 0.27), intellect and arousal (*r* = 0.03, *p* = 0.07), and intellect and surprise (*r* = -0.02, *p* = 0.33). Within personality measures, all were significantly correlated with each other, and the strongest correlation was observed between curiosity and awe-proneness (*r* = 0.57, *p*<.01).

Table 2. Bivariate correlation coefficients for creativity, its predictors, and the personality measures of the readers.

**Variable**	**M**	**SD**	**1**	**2**	**3**	**4**	**5**	**6**	**7**	**8**	**9**
1. Clarity	4.82	1.58									
2.Aesthetic Appeal	4.8	1.44	.57**								
3. Felt Valence	4.5	1.62	.54**	.68**							
4. Felt Arousal	3.86	1.73	.38**	.51**	.64**						
5. Surprise	3.78	1.68	.31**	.43**	.53**	.59**					
6. Creativity	4.91	1.38	.42**	.66**	.60**	.47**	.52**				
7. Openness	5.02	0.74	.09**	.12**	.04*	0.02	-.10**	.17**			
8. Intellect	4.7	0.9	.11**	.17**	.05**	0.03	-0.02	.17**	.43**		
9. Awe-proneness	5.11	1.14	.10**	.15**	.14**	.09**	.08**	.20**	.47**	.[illegible]6**	
10. Curiosity	5.58	0.86	.12**	.16**	.13**	.07**	.08**	.22**	.33**	.47**	.57**

Note. *M* and *SD* are used to represent mean and standard deviation, respectively. * indicates *p* < .05. ** indicates *p* < .01.

Parsimonious predictors

To determine the order of variable inclusion in the predictor model, we employed the forward selection method. The variables were added based on their correlation with the

outcome variable, i.e., creativity. The variable with the highest correlation was included first in the null model, followed by the other variables in the descending order of their correlations with creativity, as shown in Table 2. Consequently, the predictor variables were entered into the model in the following order: aesthetic appeal, felt valence, surprise, arousal, and clarity. To compare five linear mixed models, various criteria such as Information Criteria , such as Akaike information criterion (*AIC*), Bayesian information criterion (*BIC*), the proportion of variance explained by fixed effects (R^2), and Likelihood ratio test statistic ($\Delta\chi$^2) were used, and the model comparison was presented in Table 3. The model comprising aesthetic appeal, felt valence, and surprise demonstrated the optimal fit and parsimony as indicated by a significant likelihood ratio test statistic ($\Delta\chi$^2= 294.51, p<0.001) along with a lower Bayesian Information Criterion (*BIC*= 9069.6) compared to the alternative models. Therefore, the model that included aesthetic appeal, felt valence, and surprise, was selected as the most optimal for predicting creativity.

Table 3. Model comparison to identify the best model fit comprising aesthetic appeal, felt valence, and surprise.

Information Criteria	Null Model	Model 1	Model 2	Model 3	Model 4	Model 5
AIC	11160.26	9586.52	9325.25	9032.73	9016.77	9018.37
BIC	11178.7	9611.12	9356	9069.62	9059.81	9067.55
R^2	0	0.26	0.29	0.33	0.33	0.33
$\Delta\chi^2$		1575.73***	263.28***	294.51***	17.96***	0.4

Note: Aesthetic appeal, felt valence, surprise, arousal and clarity are included sequentially to Model 1 to Model 5; all models are compared hierarchically, i.e., Model 1 is compared to Null Model, Model 2 is compared to Model 1 and so on ; AIC = Akaike Information Criterion; BIC = Bayesian Information Criterion; R^2 = proportion of variation explained by fixed effects (101); Δχ2 = Likelihood ratio test statistic for comparison of models. Significance codes: 0 '***' 0.001 '**' 0.01 '*' 0.05 '.' 0.1 ' ' 1

The linear mixed model result for the best-fitting model is presented in Table 4. Aesthetic appeal was found to be the best predictor (b = 0.34, SE = 0.02, t = 22.14, p<0.001),

indicating a significant positive relationship with creativity. Following that, surprise significantly influenced creativity (*b* = 0.23, *SE* = 0.01, *t* = 17.54, *p*<0.001), showing a positive association with creativity. Felt valence, although demonstrating a relatively weaker but still significant effect on creativity (*b* = 0.16, *SE* = 0.01, *t* =11.56, *p*<0.001), was also positively associated with creativity. On the other hand, clarity did not significantly predict creativity (*b* = -0.01, *SE* = 0.01, *t* = - 0.63, *p* = 0.53) and was eliminated from subsequent analysis. Further, the model comparison revealed that felt arousal (*b* = 0.07, *SE* = 0.02, *t* =4.28, *p*<.001) did not contribute significantly to improving the model fit ($\Delta\chi^2$ =17.962 R^2 = 0.33) and was not considered to be the part of our parsimonious model. Of note, two alternative methods corroborated the appropriateness of this model. The first method involved a backward elimination approach, where the least correlated variable was progressively removed from the full model. The second approach involved variables entered in an arbitrary order (e.g., clarity, aesthetic appeal, felt valence, arousal, and surprise). Both methods consistently indicated that the model comprising aesthetic appeal, felt valence, and surprise yielded the best fit. Therefore, aesthetic appeal, surprise, and felt valence were identified as parsimonious predictors of poetic creativity. Next, we analysed the interaction of the four personality traits with these three predictors.

Table 4. The linear mixed model results for the best-fitting model, comprised of aesthetic appeal, surprise, and felt valence as the predictors of creativity judgment.

MODEL INFO:

Observations: 3456

Dependent Variable: Creativity

Type: Mixed effects linear regression

MODEL FIT:

AIC = 9032.7, *BIC* = 9069.6

Pseudo-R^2 *(fixed effects)* = 0.33

Pseudo-R^2 (total) = 0.62

Fixed Effects					
	Estimates	SE	df	t-value	p-value
Predictors					
(Intercept)	4.91	0.08	96	63.33	<0.001
Aesthetic appeal	0.34	0.02	3360	22.14	<0.001
Felt valence	0.16	0.01	3360	11.56	<0.001
Surprise	0.23	0.01	3360	17.54	<0.001
Random Effects					
Groups	Variance	SD			
Participants (Intercept)	0.56	0.75			
Residual	0.73	0.85			
ICC	0.43				
N(Participants)	96				
Observations	3456				
Marginal R^2	0.33				
Conditional R^2	0.62				

Note. ICC = Intraclass correlation coefficient

Moderating role of the personality traits

We explored the interaction of each of the four personality traits – openness, intellect, awe-proneness, and curiosity – with the three significant predictors of poetic creativity – aesthetic appeal, surprise, and felt valence. Table 5 displays the main effects of the moderators and their interactions with the predictors in the models involving four personality traits.

Table 5. Models with Main Effects and Interactions of Personality Traits with Respective Moderators.

Model	b	SE	t	p	Fit (R^2)
**Openness Model**					
Intercept	3.32	0.5	6.58	<0.001	
Openness	0.32	0.1	3.18	<0.001	
Aesthetic Appeal	-0.15	0.1	-1.46	0.14	
Felt Valence	0.47	0.1	4.89	<0.001	
Surprise	0.66	0.09	7.2	<0.001	
Openness*Aesthetic Appeal	0.1	0.02	4.83	<0.001	
Openness*Felt Valence	-0.06	0.02	-3.27	<0.001	
Openness*Surprise	-0.08	0.02	-4.76	<0.001	.36**
**Intellect Model**					
Intercept	3.67	0.39	9.35	<0.001	
Intellect	0.26	0.08	3.21	<0.001	
Aesthetic Appeal	-0.03	0.08	-0.35	0.72	
Felt Valence	0.22	0.08	2.8	0.01	
Surprise	0.31	0.08	3.92	<0.001	
Intellect*Aesthetic Appeal	0.08	0.02	4.51	<0.001	
Intellect*Felt Valence	-0.01	0.02	-0.74	0.46	
Intellect*Surprise	-0.02	0.02	-1	0.32	.36**
**Awe-proneness Model**					
Intercept	3.69	0.33	11.1	<0.001	
Awe-proneness Model	0.24	0.06	3.77	<0.001	
Aesthetic Appeal	0.17	0.06	2.71	0.01	
Felt Valence	0.18	0.06	2.96	<0.001	
Surprise	0.37	0.06	6.32	<0.001	
Awe-proneness*Aesthetic Appeal	0.03	0.01	2.67	0.01	
Awe-proneness*Felt Valence	0	0.01	-0.3	0.76	
Awe-proneness*Surprise	-0.03	0.01	-2.48	0.01	.37**
**Curiosity Model**					
Intercept	2.9	0.46	6.29	<0.001	
Curiosity	0.36	0.08	4.39	<0.001	
Aesthetic Appeal	0.06	0.1	0.63	0.53	
Felt Valence	0.03	0.09	0.37	0.71	
Surprise	0.5	0.08	5.99	<0.001	
Curiosity*Aesthetic Appeal	0.05	0.02	2.96	<0.001	
Curiosity*Felt Valence	0.02	0.02	1.43	0.15	
Curiosity*Surprise	-0.05	0.01	-3.27	<0.001	.38**

Openness exhibited significant moderation effect on aesthetic appeal (b = 0.10, SE = 0.02, t = 4.83, p<.001), felt valence (b = -0.06, SE= 0.02, t = -3.27, p<.001), and surprise (b = -0.08, SE=0.02, t = -4.76, p<.001) (Fig 1). A significant moderation of intellect was observed on aesthetic appeal (b = 0.08, SE= 0.02, t = 4.51, p<.001) with valence (b = -0.01, SE = 0.02, t = -0.74, p = 0.46) and surprise (b = -0.02, SE = 0.02, t = -1.00, p = 0.32) being unmoderated (Fig 2).

Figure 1. Simple slopes showing the interactions between openness as the moderator and aesthetic appeal, felt valence, and surprise as the predictors.

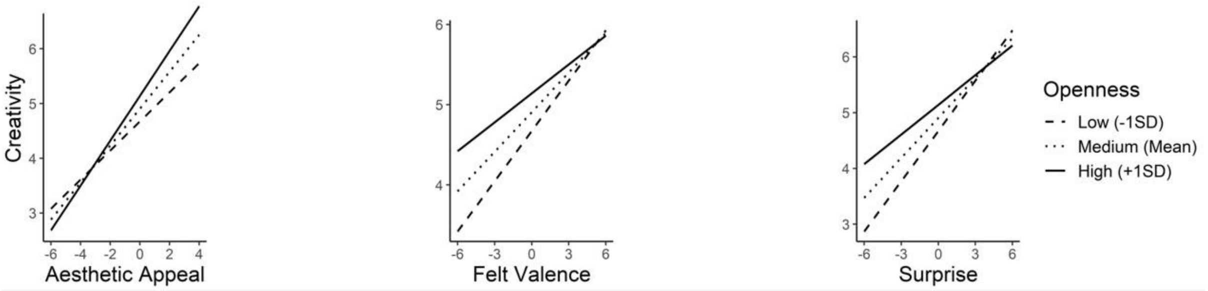

Figure 2. Simple slopes showing the significant interactions between intellect as the moderator and aesthetic appeal as the predictor.

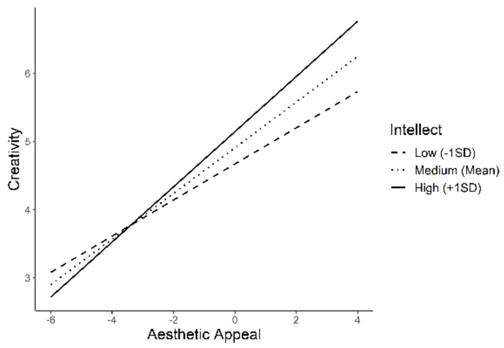

Awe-proneness was found to be a significant moderator on the relationship between creativity and aesthetic appeal (*b* = 0.03, *SE*= 0.01, *t* = 2.67, *p* = 0.01), and surprise (*b* = -0.03, *SE* = 0.01, t = -2.48, *p*= 0.01), whereas no significant moderation with valence was observed (*b* = -0.00, *SE*= 0.01, *t* = -0.30, *p* = 0.76) (Fig 3). Finally, curiosity was found to significantly moderate aesthetic appeal (*b* = 0.04, *SE*= 0.02, *t* = 2.46, *p* = 0.01), and surprise (*b* = -0.05, *SE* = 0.01, *t* = -3.72, *p*<0.001), leaving felt valence unmoderated (*b* = 0.01, *SE* = 0.02, *t* = 0.84, *p* = 0.40) (Fig 4).

Figure 3. Simple slopes showing the interactions between awe-proneness as the moderator and aesthetic appeal and surprise as the predictors.

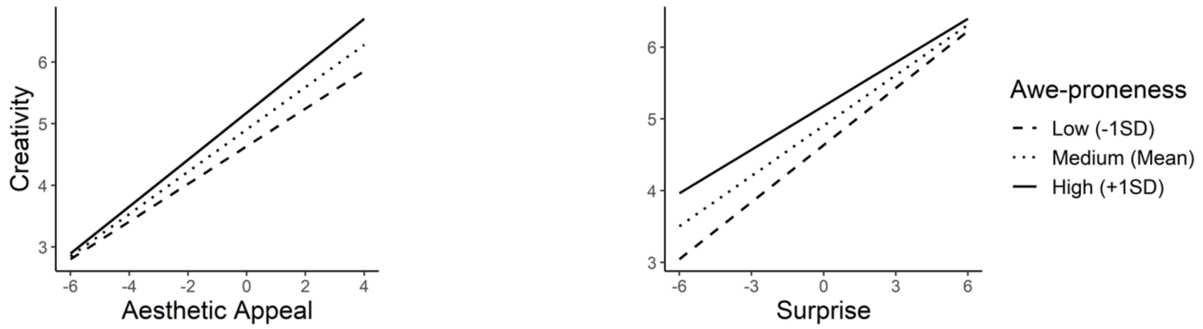

Figure 4. Simple slopes showing the interactions between curiosity as the moderator and aesthetic appealand surprise as the predictors.

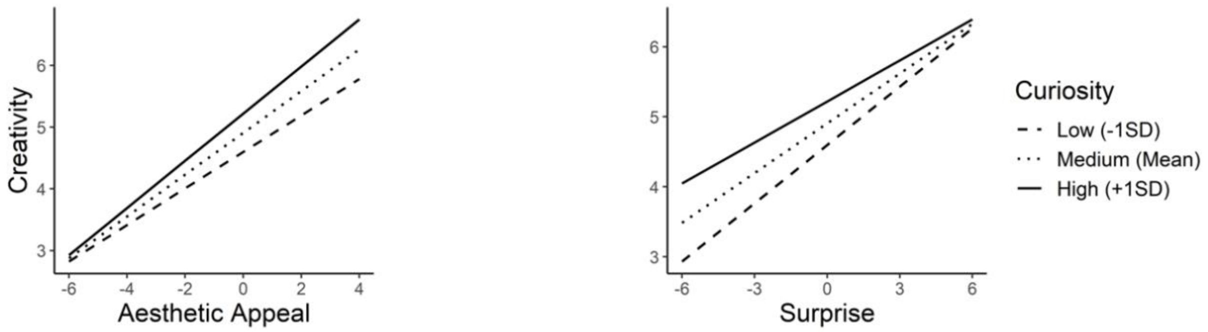

Consequently, all four personality traits exhibited significant moderation effects on both aesthetic appeal and surprise. However, distinct patterns were observed in the

moderation effects between these two predictors. The linear positive impact of aesthetic appeal on creativity was strengthened to a greater extent for higher values of the moderators. In contrast, the positive effect of surprise on creativity was attenuated for the higher moderator values The simple slopes analyses results are depicted in in Table 6.

Table 6. Results of simple slopes analyses of the predictors for the high and low levels of the moderators.

		High (+1 SD)				Low (-1SD)			
Predictor	Moderator	Est	*SE*	*t* -value	*p* -value	Est	*SE*	*t* -value	*p* -value
	Openness								
Aesthetic Appeal		0.41	0.02	19.51	<0.001	0.27	0.02	12.25	<0.001
Felt Valence		0.12	0.02	6.55	<0.001	0.21	0.02	9.92	<0.001
Surprise		0.18	0.02	10.55	<0.001	0.3	0.02	14.87	<0.001
	Intellect								
Aesthetic Appeal		0.41	0.02	19.31	<0.001	0.27	0.02	11.76	<0.001
	Awe-proneness								
Aesthetic Appeal		0.38	0.02	17.51	<0.001	0.31	0.02	15.18	<0.001
Surprise		0.2	0.02	11.73	<0.001	0.27	0.02	13.86	<0.001
	Curiosity								
Aesthetic Appeal		0.38	0.02	18.15	<0.001	0.3	0.02	13.91	<0.001
Surprise		0.2	0.02	11.48	<0.001	0.28	0.02	14.33	<0.001

Arousal was not included in our parsimonious model as a potential predictor of creativity judgment of poetry. Nevertheless, we recognized the possibility that a predictor might not demonstrate main effect but could still show significant interaction when combined with another factor. Therefore, we examined the interaction effects on arousal. Results are as follows: openness interaction: $b = -0.01$, $SE = 0.02$, $t = -0.46$, $p = 0.64$; intellect interaction: $b = 0.03$, $SE = 0.02$, $t = 1.86$, $p = 0.06$; awe-proneness interaction: $b = -0.01$, $SE = 0.01$, $t = -0.99$, $p = 0.32$; curiosity interaction: $b = 0.00$, $SE = 0.02$, $t = 0.17$, $p = 0.86$). The findings indicated that influence of arousal on creativity remained unaltered by any of the four moderators.

Discussion

The current study investigated the moderating effects of four personality traits – openness, intellect, awe-proneness, and curiosity - on the evaluation of creativity in English language poems:~~ openness.~~ Initially, we identified three predictors, out of the potential five, of creativity judgment of poems, as follows: aesthetic appeal, felt valence, and surprise. Subsequently, we investigated how these predictors varied concerning the participant's personality traits. We observed that individuals with higher levels of openness, intellect, curiosity, and awe-proneness gave more importance to aesthetic appeal, in descending order while assessing the creativity of poems. Notably, only the openness trait showed a moderating effect on felt valence, while the other traits did not demonstrate a significant effect.

We identified distinct moderation effects of openness and intellect on the assessment of poetic creativity. Individuals with higher levels of both traits demonstrated a pronounced emphasis on a poem's aesthetic appeal when evaluating poetic creativity, compared to those with lower levels of openness and intellect. Despite being separate traits with a correlation ranging from 0.3 to 0.5 (69), openness and intellect exhibited a shared tendency in appreciating a poem's aesthetic appeal. As aesthetic experience is both style-related and art-specific, involving cognitive and affective processing (102), individuals with higher levels of openness and intellect engaged more deeply with both the cognitive and affective aspects during the evaluation process. We postulate that this heightened engagement led them to assign greater significance to the aesthetic appeal of poems in their creativity assessments. In line with prior research (67), we found a stable and distinct connection between openness, Intellect, and aesthetic engagement; both openness and intellect [illegible]lect a general inclination towards aesthetic experiences—whether it be through sensory and aesthetic information

processing (associated with openness) or abstract and complex semantic information processing (associated with intellect) (62). Open individuals, i.e., who were assumed to be more unconventional, imaginative, and creative, (102,10) exhibited a more pronounced preference for aesthetic appeal in their evaluation of poetic creativity than those with lower levels.

Interestingly, we observed that individuals with lower levels of openness were more influenced by felt valence in their creativity ratings of poems than those with higher levels of openness. Valence is a core aspect of emotion (103,104,105), and aesthetic experience involves a cognitive process accompanied by evolving affective states, resulting in an aesthetic emotion (102). The distinct moderating effects of openness on aesthetic appeal and felt valence in our study suggest that the aesthetic emotions evoked through affective processing of poetry might differ from the emotional valence experienced during the reading of poetry. Notably, levels of intellect did not exert any influence on felt valence.

Individuals with lower levels of openness were found to be more influenced by surprise in their creativity ratings of poems than their higher counterparts. Surprise is often recognized as an interruption mechanism and a short-lived emotion (55), with an unclear positive or negative valence. Therefore, for more open individuals, the transient and ambiguous nature of surprise might disrupt their affective states, leading to a diminished impact of surprise on their creativity judgment. In contrast, less open individuals perceived surprise as a more significant factor in their evaluation of poetic creativity than their higher counterparts. This finding speculates that the traditional 3-criterion definition of creativity (59), which includes surprise as a key component, in the context of poetry may resonate more with readers with lower levels of openness.Thus supporting the fact that openness/intellect is an aesthetically sensitive personality domain (67) and consistently serves as a predictor of

both artistic creativity and aesthetic appreciation (4,26,106) across a diverse form of art (20, 64, 107), our study reveals that, individuals with higher openness and intellect, place particular emphasis on the positive impact of aesthetic appeal of poems when evaluating their creativity. However, our findings indicate distinct differences in the moderation effects of openness and intellect when assessing felt valence and surprise in poems during creativity evaluation emphasizing the nuanced distinction between openness and intellect (69).

Awe-proneness, in our study, demonstrated significant interactions with aesthetic appeal and surprise, but not with felt valence. Awe, a specific emotional response often triggered by beauty, is considered a key member of the self-transcendent emotions (108). In line with the model of apreciation of beauty and excellence (109), where the ability to perceive and appreciate beauty involves the experience of self-transcendent emotion like awe (108), our findings revealed that individuals with higher levels of awe-proneness placed greater emphasis on the aesthetic appeal of a poem when evaluating its creative potential. This suggests that readers predisposed to feeling awe might be more sensitive to the artistic and moral beauty of the poems (110). Further, we observed a significant correlation (r = .47) between awe-proneness and openness, a finding corroborated by previous research (111). Our moderation results suggest that dispositional awe is linked to creativity judgment, and appreciation for beauty (109, 112). Interestingly, we observed that individuals with lower levels of awe-proneness were more influenced by surprise in their judgments of cretivity. Previous research suggests that awe experiences do not require intensive effortful, controlled processing (111), and further, dispositional awe is inversely correlated with the need for cognitive closure (76). Therefore, we suggest that in the evaluation of poetic creativity, individuals with higher awe-proneness would prioritize aesthetic appeal while adopting a more passive and receptive stance towards unexpected elements in poetry (80).

Curiosity exhibited significant moderating effects for aesthetic appeal and surprise, mirroring the interaction patterns of awe-proneness. Individuals with heightened curiosity, driven by a desire for new knowledge and experiences (113), demonstrated a more pronounced influence of the aesthetic appeal of a poem on its creativity. This reinforces the idea that curiosity is instrumental in facilitating aesthetic experiences and in the pursuit of understanding complex, abstract, and intellectually challenging stimuli (114). Additionally, our findings align with previous research indicating that individuals with high trait curiosity tend to find complex poems more comprehensible and engaging (115). **The contribution of aesthetic appeal appreciation to the creativity judgment may have been ~~more~~ fostered by the intrinsic behavioural traits of highly curious readers which compel them to delve more deeply into the unfamiliar aspects of the poems.** In contrast, **the influence of surprise on creativity judgment was more pronounced in those individuals with lower levels of curiosity**, indicating that surprise, as a short-lived emotion and interruption mechanism (55), may interrupt the information-seeking tendencies of highly curious readers during their evaluation of poetic creativity.

The observed similarities in the interaction patterns of openness and curiosity underscore a well-established link between openness and curiosity (89, 116, 117, 118), indicating that individuals with high openness are more motivated to learn, inclined to explore, and interested in acquiring information. These tendencies might enhance their semantic knowledge (119), and subsequently, their aesthetical experiences (114) and the judgment of poetic creativity. Our results also suggest that awe-prone individuals are more curious (120), and that awe itself can stimulate curiosity (121). Altogether, these suggest that higher levels of awe-proneness and curiosity might amplify the perceived ability to comprehend complex stimuli like poetry (115).

Limitations

The current study is not without limitations. First, we focused on felt emotions experienced by participants while reading poems, rather than perceived emotions, which are the perceived emotional quality of the poems. Perceived and felt emotions are not necessarily identical, as highlighted in studies on music (53,54,122). We suggest that this is also likely to be the case for poems. For instance, a poem with a ‘sad’ theme may not necessarily induce sadness in the reader. Of note, previous research has reported an association between perceived valence and aesthetic appeal of poetry (16). Therefore, future work could investigate perceived emotions’ predictive power on a poem’s creativity and the potential moderating role of traits like intellect. Second, we focused on trait-level personality characteristics rather than state-level personality features. However, contextualized personality traits are crucial for capturing within-individual variability (123), indicating a need for future studies to include state-level individual differences for a more comprehensive understanding of poetry evaluation. Third, we did not control for various structural elements of poems such as rhythm, form, and genre**.** We did not restrict the poems to specific lengths, rhythmic patterns, or specific forms or genres, like sonnets, haiku, limericks, or others. Exploring the specific effects of genres and forms was not feasible due to the limited number of poems in our study, so the potential influence of these objective features inherent on the creativity assessment could not be ruled out. Fourth, the representativeness of the selected poems may also be limited, potentially impacting the generalizability of our findings. Fifth, concerning the diversity measures of the stimuli, **it is important to acknowledge that given the small word count of some of our poems and the implied limited vocabulary, the Type-Token Ratio (TTR) method might not yield reliable results due to constrained variability in word usage within small texts (124, 125)**. Sixth, **we assessed the variables using single item measures, a common practice in assessing**

aesthetics in visual art (126, 127), poetry (16,17,92,128, 129) and music (130). However, we also recognize the potential variability in individual interpretation of the questions remains unexplored. Employing multiple items for variable assessment could have offered psychometric advantages, particularly in enhancing reliability and validity (131). Finally, we acknowledge that the operationalization of 'creativity' is different from the more traditional view of creativity as a characteristic of individuals. Considering that creativity, as per the 4P Model (132), is defined through four lenses: person, product, process, and press, we strategically focused on the 'product' aspect of creativity, particularly in the context of poetry. We, here operationalized 'creativity' as the poem's creative potential. This choice was made to expand the understanding of creativity beyond the creator to the creation itself. Our approach is in line with numerous past studies which have investigated the creativity evaluation of various types of products/artefacts, such as ideas (15), musical compositions (133, 25), short stories (134), and product concepts (135), to name a few. This approach enabled us to explore the internal model of how individuals judge the creativity of poems, recognizing that such judgments may be inherently subjective and influenced by individual personality traits.

Conclusions

In summary, our study investigated how specific personality traits, namely openness, intellect, awe-proneness, and epistemic curiosity, influence the evaluation of creativity of English language poetry. We focused on how these traits moderate the impact of three predictors - aesthetic appeal, felt valence, and surprise - in forming a parsimonious model for evaluating poetic creativity. Among four traits, openness exerted the most significant moderating effect on all three predictors, and among the predictors, aesthetic appeal was

significantly moderated by all personality traits in assessing the creativity of poems. These results altogether demonstrate how specific personality traits moderate the underlying model of creativity judgment of English poems, thereby explaining the variability in individual preferences and evaluations.

Author Contributions

Conceptualization: Soma Chaudhuri, Joydeep Bhattacharya.

Stimuli selection and editing: Soma Chaudhuri, Maura Dooley

Data curation: Soma Chaudhuri.

Formal analysis: Soma Chaudhuri.

Methodology: Soma Chaudhuri, Alan Pickering, Joydeep Bhattacharya.

Supervision: Joydeep Bhattacharya.

Editing: Joydeep Bhattacharya, Alan Pickering, Maura Dooley

Data availability

All data and code are available in the OSF at:

https://osf.io/9mw7r/?view_only=07137f4871d146c790501f22bc7743d5

References

1. Diener E, Lucas RE. Personality traits. Gen Psychol Required Read. 2019;278.

2. Matthews G, Deary IJ, Whiteman MC. Personality traits. Cambridge University Press; 2003.

3. Barron F, Harrington DM. Creativity, intelligence, and personality. Annu Rev Psychol. 1981;32(1):439–76.

4. Feist GJ. A meta-analysis of personality in scientific and artistic creativity. Personal Soc Psychol Rev. 1998;2(4):290–309.

5. Feist GJ, Barron FX. Predicting creativity from early to late adulthood: Intellect, potential, and personality. J Res Pers. 2003;37(2):62–88.

6. Batey M, Furnham A. Creativity, intelligence, and personality: A critical review of the scattered literature. Genet Soc Gen Psychol Monogr [Internet]. 2006 Nov 1;132(4):355–429. Available from: https://doi.org/10.3200/MONO.132.4.355-430

7. DeYoung CG. Openness/intellect: A dimension of personality reflecting cognitive exploration. In: APA handbook of personality and social psychology, Volume 4: Personality processes and individual differences. Washington, DC, US: American Psychological Association; 2015. p. 369–99. (APA handbooks in psychology®.).

8. Kaufman SB, Quilty LC, Grazioplene RG, Hirsh JB, Gray JR, Peterson JB, et al. Openness to experience and intellect differentially predict creative achievement in the arts and sciences. J Pers [Internet]. 2016 Apr 1;84(2):248–58. Available from: https://doi.org/10.1111/jopy.12156

9. Silvia PJ, Nusbaum EC, Berg C, Martin C, O'Connor A. Openness to experience, plasticity, and creativity: Exploring lower-order, high-order, and interactive effects. J Res Pers [Internet]. 2009;43(6):1087–90. Available from:

https://www.sciencedirect.com/science/article/pii/S0092656609001317

10. McCrae RR. Creativity, divergent thinking, and openness to experience. J Pers Soc Psychol. 1987;52:1258–65.

11. Tan C-S, Lau X-S, Kung Y-T, Kailsan RA. Openness to experience enhances creativity: The mediating role of intrinsic motivation and the creative process engagement. J Creat Behav [Internet]. 2019 Mar 1;53(1):109–19. Available from: https://doi.org/10.1002/jocb.170

12. Dollinger SJ, Urban KK, James TA. Creativity and openness: Further validation of two creative product measures. Creat Res J [Internet]. 2004 Mar 1;16(1):35–47. Available from: https://doi.org/10.1207/s15326934crj1601_4

13. Bejar II. Rater cognition: Implications for validity. Educ Meas Issues Pract [Internet]. 2012 Sep 1;31(3):2–9. Available from: https://doi.org/10.1111/j.1745-3992.2012.00238.x

14. Ceh SM, Edelmann C, Hofer G, Benedek M. Assessing raters: What factors predict discernment in novice creativity raters? J Creat Behav. 2022;56(1):41–54.

15. Lloyd-Cox J, Pickering A, Bhattacharya J. Evaluating creativity: How idea context and rater personality affect considerations of novelty and usefulness. Creat Res J [Internet]. 2022 Oct 2;34(4):373–90. Available from: https://doi.org/10.1080/10400419.2022.2125721

16. Belfi AM, Vessel EA, Starr GG. Individual ratings of vividness predict aesthetic appeal in poetry. Psychol Aesthetics, Creat Arts. 2018;12(3):341–50.

17. Hitsuwari J, Nomura M. How individual states and traits predict aesthetic appreciation of haiku poetry. Empir Stud Arts. 2022;40(1):81–99.

18. Hitsuwari J, Nomura M. Ambiguity tolerance can improve through poetry appreciation and creation. J Creat Behav [Internet]. 2022 Dec 7;n/a(n/a). Available from: https://doi.org/10.1002/jocb.574

19. Amabile TM. Social psychology of creativity: A consensual assessment technique. J Pers Soc Psychol. 1982;43:997–1013.

20. Chamorro-Premuzic T, Burke C, Hsu A, Swami V. Personality predictors of artistic preferences as a function of the emotional valence and perceived complexity of paintings. Psychol Aesthetics, Creat Arts. 2010;4(4):196.

21. Tinio PPL. From artistic creation to aesthetic reception: The mirror model of art. Psychol Aesthetics, Creat Arts. 2013;7(3):265.

22. Cupchik GC, Vartanian O, Crawley A, Mikulis DJ. Viewing artworks: Contributions of cognitive control and perceptual facilitation to aesthetic experience. Brain Cogn [Internet]. 2009;70(1):84–91. Available from: https://www.sciencedirect.com/science/article/pii/S0278262609000098

23. Bao Y, Yang T, Lin X, Fang Y, Wang Y, Pöppel E, et al. Aesthetic preferences for Eastern and Western traditional visual art: identity matters. Front Psychol. 2016;7:1596.

24. Hagtvedt H, Patrick VM, Hagtvedt R. The perception and evaluation of visual art. Empir Stud Arts [Internet]. 2008 Jul 1;26(2):197–218. Available from: https://doi.org/10.2190/EM.26.2.d

25. Zioga I, Harrison PMC, Pearce MT, Bhattacharya J, Di Bernardi Luft C. From learning to creativity: Identifying the behavioural and neural correlates of learning to predict human judgements of musical creativity. Neuroimage [Internet]. 2020;206:116311.

Available from: https://www.sciencedirect.com/science/article/pii/S1053811919309024

26. Silvia PJ, Fayn K, Nusbaum EC, Beaty RE. Openness to experience and awe in response to nature and music: Personality and profound aesthetic experiences. Psychol Aesthetics, Creat Arts. 2015;9:376–84.

27. Jackendoff R, Lerdahl F. The capacity for music: What is it, and what's special about it? Cognition [Internet]. 2006;100(1):33–72. Available from: https://www.sciencedirect.com/science/article/pii/S0010027705002210

28. Koelsch S. Brain correlates of music-evoked emotions. Nat Rev Neurosci [Internet]. 2014;15(3):170–80. Available from: https://doi.org/10.1038/nrn3666

29. Belfi AM. Emotional valence and vividness of imagery predict aesthetic appeal in music. . Psychomusicology Music Mind, Brain . 2019;29((2-3)):128–35.

30. Reybrouck M, Brattico E. Neuroplasticity beyond sounds: neural adaptations following long-term musical aesthetic experiences. Brain Sci. 2015;5(1):69–91.

31. Müller M, Höfel L, Brattico E, Jacobsen T. Aesthetic judgments of music in experts and laypersons — An ERP study. Int J Psychophysiol [Internet]. 2010;76(1):40–51. Available from: https://www.sciencedirect.com/science/article/pii/S0167876010000334

32. Hanich, J., Wagner, V., Shah, M., Jacobsen, T., & Menninghaus W. Why we like to watch sad films. The pleasure of being moved in aesthetic experiences. Psychol Aesthetics, Creat Arts. 2014;8(2):130–43.

33. Silvia PJ, Berg C. Finding movies interesting: How appraisals and expertise influence the aesthetic experience of film. Empir Stud Arts. 2011;29(1):73–88.

34. Plucker JA, Kaufman JC, Temple JS, Qian M. Do experts and novices evaluate movies the same way? Psychol Mark [Internet]. 2009 May 1;26(5):470–8. Available from: https://doi.org/10.1002/mar.20283

35. Jacobs AM, Kinder A. “The brain is the prisoner of thought”: A machine-learning assisted quantitative narrative analysis of literary metaphors for use in neurocognitive poetics. Metaphor Symb [Internet]. 2017 Jul 3;32(3):139–60. Available from: https://doi.org/10.1080/10926488.2017.1338015

36. Obermeier C, Menninghaus W, von Koppenfels M, Raettig T, Schmidt-Kassow M, Otterbein S, et al. Aesthetic and emotional effects of meter and rhyme in poetry [Internet]. Vol. 4, Frontiers in Psychology. 2013. Available from: https://www.frontiersin.org/articles/10.3389/fpsyg.2013.00010

37. Kraxenberger M, Menninghaus W. Affinity for poetry and aesthetic appreciation of joyful and sad poems [Internet]. Vol. 7, Frontiers in Psychology. 2017. Available from: https://www.frontiersin.org/articles/10.3389/fpsyg.2016.02051

38. Hitsuwari J, Nomura M. Ambiguity and beauty: Japanese-German cross-cultural comparisons on aesthetic evaluation of haiku poetry. Psychol Aesthetics, Creat Arts. 2022;No Pagination Specified-No Pagination Specified.

39. Scharinger M, Wagner V, Knoop CA, Menninghaus W. Melody in poems and songs: Fundamental statistical properties predict aesthetic evaluation. Psychol Aesthetics, Creat Arts. 2022;No Pagination Specified-No Pagination Specified.

40. Lau JH, Cohn T, Baldwin T, Brooke J, Hammond A. Deep-speare: A joint neural model of poetic language, meter and rhyme. 2018 Jul 10 [cited 2022 Nov 25]; Available from: http://arxiv.org/abs/1807.03491

41. Jacobs AM, Kinder A. What makes a metaphor literary? Answers from two computational studies. Metaphor Symb. 2018;33(2):85–100.

42. Steen GJ. From linguistic form to conceptual structure in five steps: Analyzing metaphor in poetry. Cogn Poet. 2009;197–226.

43. Rasse C, Onysko A, Citron FMM. Conceptual metaphors in poetry interpretation: A psycholinguistic approach. Lang Cogn [Internet]. 2020/02/28. 2020;12(2):310–42. Available from: https://www.cambridge.org/core/article/conceptual-metaphors-in-poetry-interpretation-a-psycholinguistic-approach/E022AA06246E014936DB9329D6B99009

44. Jacobs AM. Quantifying the beauty of words: A neurocognitive poetics perspective [Internet]. Vol. 11, Frontiers in Human Neuroscience. 2017. Available from: https://www.frontiersin.org/articles/10.3389/fnhum.2017.00622

45. Aryani A, Kraxenberger M, Ullrich S, Jacobs AM, Conrad M. Measuring the basic affective tone of poems via phonological saliency and iconicity. Psychol Aesthetics, Creat Arts. 2016;10:191–204.

46. Lüdtke J, Meyer-Sickendieck B, Jacobs AM. Immersing in the stillness of an early morning: Testing the mood empathy hypothesis of poetry reception. Psychol Aesthetics, Creat Arts. 2014;8(3):363.

47. Kaufman JC, Baer J, Cole JC, Sexton∗ JD. A comparison of expert and nonexpert raters using the consensual assessment technique. Creat Res J [Internet]. 2008 May 7;20(2):171–8. Available from: https://doi.org/10.1080/10400410802059929

48. Kaufman JC, Niu W, Sexton JD, Cole JC. In the eye of the beholder: differences across ethnicity and gender in evaluating creative work. J Appl Soc Psychol [Internet].

2010 Feb 1;40(2):496–511. Available from: https://doi.org/10.1111/j.1559-1816.2009.00584.x

49. Russell JA. A circumplex model of affect. J Pers Soc Psychol. 1980;39(6):1161.

50. Wassiliwizky E, Koelsch S, Wagner V, Jacobsen T, Menninghaus W. The emotional power of poetry: neural circuitry, psychophysiology and compositional principles. Soc Cogn Affect Neurosci [Internet]. 2017 Aug 1;12(8):1229–40. Available from: https://doi.org/10.1093/scan/nsx069

51. Mastria S, Agnoli S, Corazza GE. How does emotion influence the creativity evaluation of exogenous alternative ideas? PLoS One [Internet]. 2019 Jul 5;14(7):e0219298. Available from: https://doi.org/10.1371/journal.pone.0219298

52. Johnson-Laird PN, Oatley K. How poetry evokes emotions. Acta Psychol (Amst) [Internet]. 2022;224:103506. Available from: https://www.sciencedirect.com/science/article/pii/S000169182200021X

53. Gabrielsson A. Emotion perceived and emotion felt: Same or different? Music Sci [Internet]. 2001 Sep 1;5(1_suppl):123–47. Available from: https://doi.org/10.1177/10298649020050S105

54. Marin MM, Bhattacharya J. Music induced emotions: some current issues and cross-modal comparisons. In: Hermida J, Ferrero M, editors. Music education. Hauppauge, NY: Nova Science Publishers; 2010. p. 1–38.

55. Meyer, W. U., Reisenzein, R., & Schützwohl A. Toward a process analysis of emotions: The case of surprise. Motiv Emot. 1997;21(3):251–74.

56. Meyer W-U, Niepel M, Rudolph U, Schützwohl A. An experimental analysis of surprise. Cogn Emot. 1991;5(4):295–311.

57. Noordewier MK, Breugelmans SM. On the valence of surprise. Cogn Emot [Internet]. 2013 Nov 1;27(7):1326–34. Available from: https://doi.org/10.1080/02699931.2013.777660

58. Rumelhart DE. Schemata and the cognitive system. 1984;

59. Simonton DK. Taking the U.S. patent office criteria seriously: A quantitative three-criterion creativity definition and its implications. Creat Res J [Internet]. 2012 Apr 1;24(2–3):97–106. Available from: https://doi.org/10.1080/10400419.2012.676974

60. Acar S, Burnett C, Cabra JF. Ingredients of creativity: Originality and more. Creat Res J [Internet]. 2017 Apr 3;29(2):133–44. Available from: https://doi.org/10.1080/10400419.2017.1302776

61. Pietras K, Ganczarek J. Aesthetic reactions to violations in contemporary art: The role of expertise and individual differences. Creat Res J. 2022;34(3):324–38.

62. Oleynick VC, DeYoung CG, Hyde E, Kaufman SB, Beaty RE, Silvia PJ. Openness/intellect: The core of the creative personality. In: The Cambridge handbook of creativity and personality research. New York, NY, US: Cambridge University Press; 2017. p. 9–27. (Cambridge handbooks in psychology.).

63. DeYoung CG, Grazioplene RG, Peterson JB. From madness to genius: The Openness/Intellect trait domain as a paradoxical simplex. J Res Pers [Internet]. 2012;46(1):63–78. Available from: https://www.sciencedirect.com/science/article/pii/S0092656611001644

64. Chamorro-Premuzic T, Reimers S, Hsu A, Ahmetoglu G. Who art thou? Personality predictors of artistic preferences in a large UK sample: The importance of openness. Br J Psychol [Internet]. 2009 Aug 1;100(3):501–16. Available from:

https://doi.org/10.1348/000712608X366867

65. McCrae RR. Social consequences of experiential openness. Psychol Bull. 1996;120(3):323–337.

66. Sánchez-Bernardos ML, Avia MD. Personality correlates of fantasy proneness among adolescents. Pers Individ Dif [Internet]. 2004;37(5):1069–79. Available from: https://www.sciencedirect.com/science/article/pii/S0191886903004434

67. Fayn K, MacCann C, Tiliopoulos N, Silvia PJ. Aesthetic emotions and aesthetic people: Openness predicts sensitivity to novelty in the experiences of interest and pleasure [Internet]. Vol. 6, Frontiers in Psychology. 2015. Available from: https://www.frontiersin.org/articles/10.3389/fpsyg.2015.01877

68. Silvia PJ, Christensen AP. Looking up at the curious personality: individual differences in curiosity and openness to experience. Curr Opin Behav Sci [Internet]. 2020;35:1–6. Available from: https://www.sciencedirect.com/science/article/pii/S2352154620300863

69. DeYoung CG, Quilty LC, Peterson JB. Between facets and domains: 10 aspects of the Big Five. J Pers Soc Psychol. 2007;93(5):880.

70. Mussel P. Intellect: a theoretical framework for personality traits related to intellectual achievements. J Pers Soc Psychol. 2013;104(5):885.

71. Costa PT, McCrae RR. Four ways five factors are basic. Pers Individ Dif [Internet]. 1992;13(6):653–65. Available from: https://www.sciencedirect.com/science/article/pii/019188699290236I

72. Siegel DJ. The developing mind: How relationships and the brain interact to shape who we are (2. utg.). New York Guilford Publ. 2012;

73. DeYoung CG, Quilty LC, Peterson JB, Gray JR. Openness to Experience, Intellect, and Cognitive Ability. J Pers Assess [Internet]. 2014 Jan 2;96(1):46–52. Available from: https://doi.org/10.1080/00223891.2013.806327

74. Berlyne DE. Conflict and the orientation reaction. J Exp Psychol. 1961;62:476–83.

75. Keltner D, Haidt J. Approaching awe, a moral, spiritual, and aesthetic emotion. Cogn Emot [Internet]. 2003 Jan 1;17(2):297–314. Available from: https://doi.org/10.1080/02699930302297

76. Shiota MN, Keltner D, Mossman A. The nature of awe: Elicitors, appraisals, and effects on self-concept. Cogn Emot [Internet]. 2007 Aug 1;21(5):944–63. Available from: https://doi.org/10.1080/02699930600923668

77. Williams PG, Johnson KT, Bride DL, Baucom BRW, Crowell SE. Individual differences in aesthetic engagement and proneness to aesthetic chill: Associations with awe. Psychol Aesthetics, Creat Arts. 2022;No Pagination Specified-No Pagination Specified.

78. McCrae RR. Aesthetic chills as a universal marker of openness to experience. Motiv Emot [Internet]. 2007;31(1):5–11. Available from: https://doi.org/10.1007/s11031-007-9053-1

79. Costa PT, McCrae RR. The revised neo personality inventory (neo-pi-r). SAGE Handb Personal theory Assess. 2008;2(2):179–98.

80. Frijda NH. The emotions. Cambridge University Press; 1986.

81. Berlyne DE. Curiosity and exploration. Science (80-) [Internet]. 1966 Jul 1;153(3731):25–33. Available from: https://doi.org/10.1126/science.153.3731.25

82. Keltner D, Shiota MN. New displays and new emotions: a commentary on Rozin and Cohen (2003). 2003;

83. Kashdan TB, Silvia PJ. Curiosity and interest: The benefits of thriving on novelty and challenge. Oxford Handb Posit Psychol. 2009;2:367–74.

84. Berlyne DE. A theory of human curiosity. Br J Psychol. 1954;45(3):180.

85. Loewenstein G. The psychology of curiosity: A review and reinterpretation. Psychol Bull. 1994;116(1):75.

86. Litman JA, Spielberger CD. Measuring epistemic curiosity and its diversive and specific components. J Pers Assess [Internet]. 2003 Feb 1;80(1):75–86. Available from: https://doi.org/10.1207/S15327752JPA8001_16

87. Litman J, Hutchins T, Russon R. Epistemic curiosity, feeling-of-knowing, and exploratory behaviour. Cogn Emot [Internet]. 2005 Jun 1;19(4):559–82. Available from: https://doi.org/10.1080/02699930441000427

88. McCrae, R. R., & Costa Jr PT. Conceptions and correlates of openness to experience. Handbook of personality psychology. Academic Press; 1997. 825–847 p.

89. Kashdan TB, Rose P, Fincham FD. Curiosity and exploration: facilitating positive subjective experiences and personal growth opportunities. J Pers Assess [Internet]. 2004 Jun 1;82(3):291–305. Available from: https://doi.org/10.1207/s15327752jpa8203_05

90. Kashdan TB, Gallagher MW, Silvia PJ, Winterstein BP, Breen WE, Terhar D, et al. Curiosity and Exploration Inventory--II. J Res Pers. 2009;43(6):987–998.

91. Kashdan TB, Stiksma MC, Disabato DJ, McKnight PE, Bekier J, Kaji J, et al. The

five-dimensional curiosity scale: Capturing the bandwidth of curiosity and identifying four unique subgroups of curious people. J Res Pers [Internet]. 2018;73:130–49. Available from: https://www.sciencedirect.com/science/article/pii/S0092656617301149

92. Papp-Zipernovszky O, Mangen A, Jacobs A, Lüdtke J. Shakespeare sonnet reading: An empirical study of emotional responses. Lang Lit [Internet]. 2021 Dec 7;31(3):296–324. Available from: https://doi.org/10.1177/09639470211054647

93. McCarthy PM, Jarvis S. vocd: A theoretical and empirical evaluation. Lang Test. 2007;24(4):459–88.

94. Chotlos JW. IV. A statistical and comparative analysis of individual written language samples. Psychol Monogr. 1944;56(2):75.

95. Johnson DR, Kaufman JC, Baker BS, Patterson JD, Barbot B, Green AE, et al. Divergent semantic integration (DSI): Extracting creativity from narratives with distributional semantic modeling. Behav Res Methods [Internet]. 2022; Available from: https://doi.org/10.3758/s13428-022-01986-2

96. Faul F, Erdfelder E, Lang A-G, Buchner A. G* Power 3: A flexible statistical power analysis program for the social, behavioral, and biomedical sciences. Behav Res Methods. 2007;39(2):175–91.

97. Gosling SD, Rentfrow PJ, Swann WB. A very brief measure of the Big-Five personality domains. J Res Pers [Internet]. 2003;37(6):504–28. Available from: https://www.sciencedirect.com/science/article/pii/S0092656603000461

98. Bates, D., Mächler, M., Bolker, B., & Walker S. Fitting linear mixed-effects models using lme4. J Stat Softw. 2015;67:1–48.

99. Enders, C. K., & Tofighi D. Centering predictor variables in cross-sectional multilevel models: a new look at an old issue. Psychol Methods. 2007;12(2):121.

100. Cohen, J. & Cohen P. Applied multiple regression/correlation analysis for the behavioral sciences,. 2nd ed. Hillsdale, NJ: Erlbaum.; 1983.

101. Nakagawa S, Schielzeth H. A general and simple method for obtaining R2 from generalized linear mixed- effects models. Methods Ecol Evol. 2013;4(2):133–42.

102. Leder H, Belke B, Oeberst A, Augustin D. A model of aesthetic appreciation and aesthetic judgments. Br J Psychol [Internet]. 2004 Nov 1;95(4):489–508. Available from: https://doi.org/10.1348/0007126042369811

103. Johnston V. The origin and function of pleasure. Cogn Emot. 2003;17(2):167–79.

104. Russell JA. Core affect and the psychological construction of emotion. Psychol Rev. 2003;110(1):145.

105. Barrett LF, Mesquita B, Ochsner KN, Gross JJ. The experience of emotion. Annu Rev Psychol. 2007;58:373–403.

106. Vessel EA, Rubin N. Beauty and the beholder: Highly individual taste for abstract, but not real-world images. J Vis. 2010;10(2):18.

107. Furnham A, Walker J. The influence of personality traits, previous experience of art, and demographic variables on artistic preference. Pers Individ Dif. 2001;31(6):997–1017.

108. Haidt J, Keltner D, Peterson C, Seligman ME. Appreciation of beauty and excellence. Character strengths and virtues. 2004;537–51.

109. Peterson C, Seligman MEP. Character strengths and virtues: A handbook and

classification. Vol. 1. Oxford University Press; 2004.

110. Diessner R, Solom RC, Frost NK, Parsons L, Davidson J. Engagement with beauty: Appreciating natural, artistic, and moral beauty. J Psychol. 2008;142(3):303.

111. Shiota MN, Keltner D, John OP. Positive emotion dispositions differentially associated with Big Five personality and attachment style. J Posit Psychol. 2006;1(2):61–71.

112. Güsewell A, Ruch W. Are only emotional strengths emotional? Character strengths and disposition to positive emotions. Appl Psychol Heal Well- Being. 2012;4(2):218–39.

113. Gross ME, Zedelius CM, Schooler JW. Cultivating an understanding of curiosity as a seed for creativity. Curr Opin Behav Sci. 2020;35:77–82.

114. Kenett YN, Humphries S, Chatterjee A. A Thirst for Knowledge: Grounding Curiosity, Creativity, and Aesthetics in Memory and Reward Neural Systems. Creat Res J. 2023;1–15.

115. Silvia PJ. Appraisal components and emotion traits: Examining the appraisal basis of trait curiosity. Cogn Emot [Internet]. 2008 Jan 1;22(1):94–113. Available from: https://doi.org/10.1080/02699930701298481

116. Kashdan TB, Gallagher MW, Silvia PJ, Winterstein BP, Breen WE, Terhar D, et al. The curiosity and exploration inventory-II: Development, factor structure, and psychometrics. J Res Pers [Internet]. 2009;43(6):987–98. Available from: https://www.sciencedirect.com/science/article/pii/S0092656609001275

117. Mussel P. Epistemic curiosity and related constructs: Lacking evidence of discriminant validity. Pers Individ Dif. 2010;49(5):506–10.

118. Silvia PJ. Exploring the psychology of interest. oxford university Press; 2006.

119. Christensen AP, Kenett YN, Cotter KN, Beaty RE, Silvia PJ. Remotely close associations: Openness to experience and semantic memory structure. Eur J Pers. 2018;32(4):480–92.

120. Anderson CL, Dixson DD, Monroy M, Keltner D. Are awe-prone people more curious? The relationship between dispositional awe, curiosity, and academic outcomes. J Pers [Internet]. 2020 Aug 1;88(4):762–79. Available from: https://doi.org/10.1111/jopy.12524

121. Izard CE. Human Emotions. New York: Plenum; 1977.

122. Schubert E. Emotion felt by the listener and expressed by the music: literature review and theoretical perspectives. Front Psychol. 2013;4:837.

123. Fleeson W. Toward a structure-and process-integrated view of personality: Traits as density distributions of states. J Pers Soc Psychol. 2001;80(6):1011.

124. Malvern DD, Richards BJ. A new measure of lexical diversity. Br Stud Appl Linguist. 1997;12:58–71.

125. McCarthy PM, Jarvis S. MTLD, vocd-D, and HD-D: A validation study of sophisticated approaches to lexical diversity assessment. Behav Res Methods [Internet]. 2010;42(2):381–92. Available from: https://doi.org/10.3758/BRM.42.2.381

126. Hassenzahl M. The interplay of beauty, goodness, and usability in interactive products. Human–Computer Interact. 2004;19(4):319–49.

127. Chamberlain R, Mullin C, Scheerlinck B, Wagemans J. Putting the art in artificial: Aesthetic responses to computer-generated art. Psychol Aesthetics, Creat Arts.

2018;12(2):177.

128. Frame J, Mehl K, Head K, Belfi A. The influence of sensory modality on aesthetic judgments of poetry. 2023;

129. Mehl K, Gugliano M, Belfi AM. The role of imagery and emotion in the aesthetic appeal of music, poetry, and paintings. Psychol Aesthetics, Creat Arts. 2023;No Pagination Specified-No Pagination Specified.

130. Zhang JD, Schubert E. A Single Item Measure for Identifying Musician and Nonmusician Categories Based on Measures of Musical Sophistication. Music Percept [Internet]. 2019 Jun 1;36(5):457–67. Available from: https://doi.org/10.1525/mp.2019.36.5.457

131. Bergkvist L, Rossiter JR. The predictive validity of multiple-item versus single-item measures of the same constructs. J Mark Res. 2007;44(2):175–84.

132. Rhodes M. An analysis of creativity. Phi delta kappan. 1961;42(7):305–10.

133. Pinho AL, de Manzano Ö, Fransson P, Eriksson H, Ullén F. Connecting to create: expertise in musical improvisation is associated with increased functional connectivity between premotor and prefrontal areas. J Neurosci. 2014;34(18):6156–63.

134. Toivainen T, Madrid- Valero JJ, Chapman R, McMillan A, Oliver BR, Kovas Y. Creative expressiveness in childhood writing predicts educational achievement beyond motivation and intelligence: A longitudinal, genetically informed study. Br J Educ Psychol. 2021;91(4):1395–413.

135. Guenther A, Eisenbart B, Dong A. Creativity and successful product concept selection for innovation. Int J Des Creat Innov. 2021;9(1):3–19.

Figure 1. Simple slopes showing the interactions between openness as the moderator and aesthetic appeal, felt valence, and surprise as the predictors.

Click here to access/download;Figure;Figure 1_Openness_Interactions.png

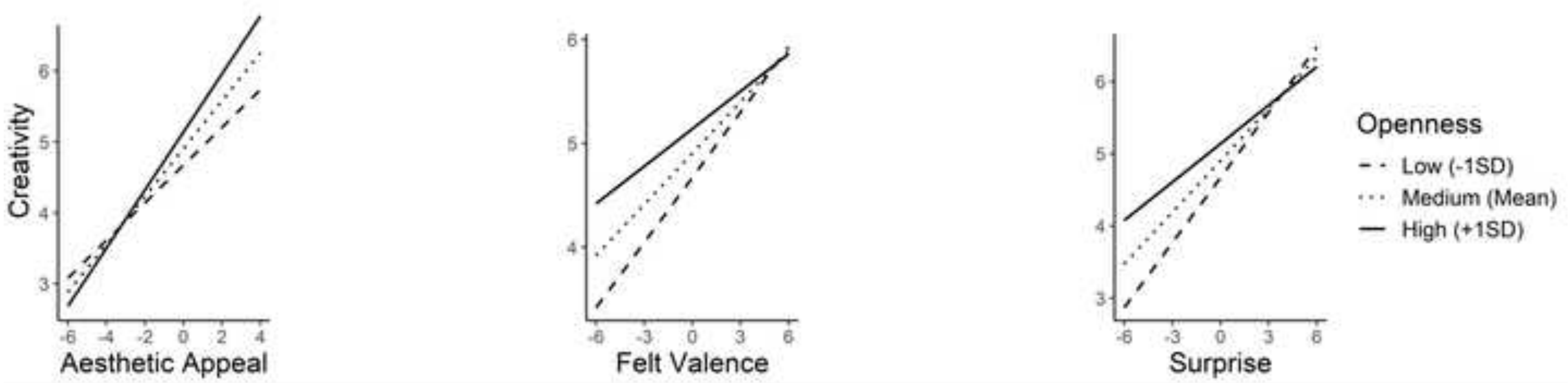

Figure 2. Simple slopes showing the significant interactions between intellect as the moderator and aesthetic appeal as the predictor.

Click here to access/download;Figure;Figure 2_Intellect_Interaction_Aesthetic_Appeal.png

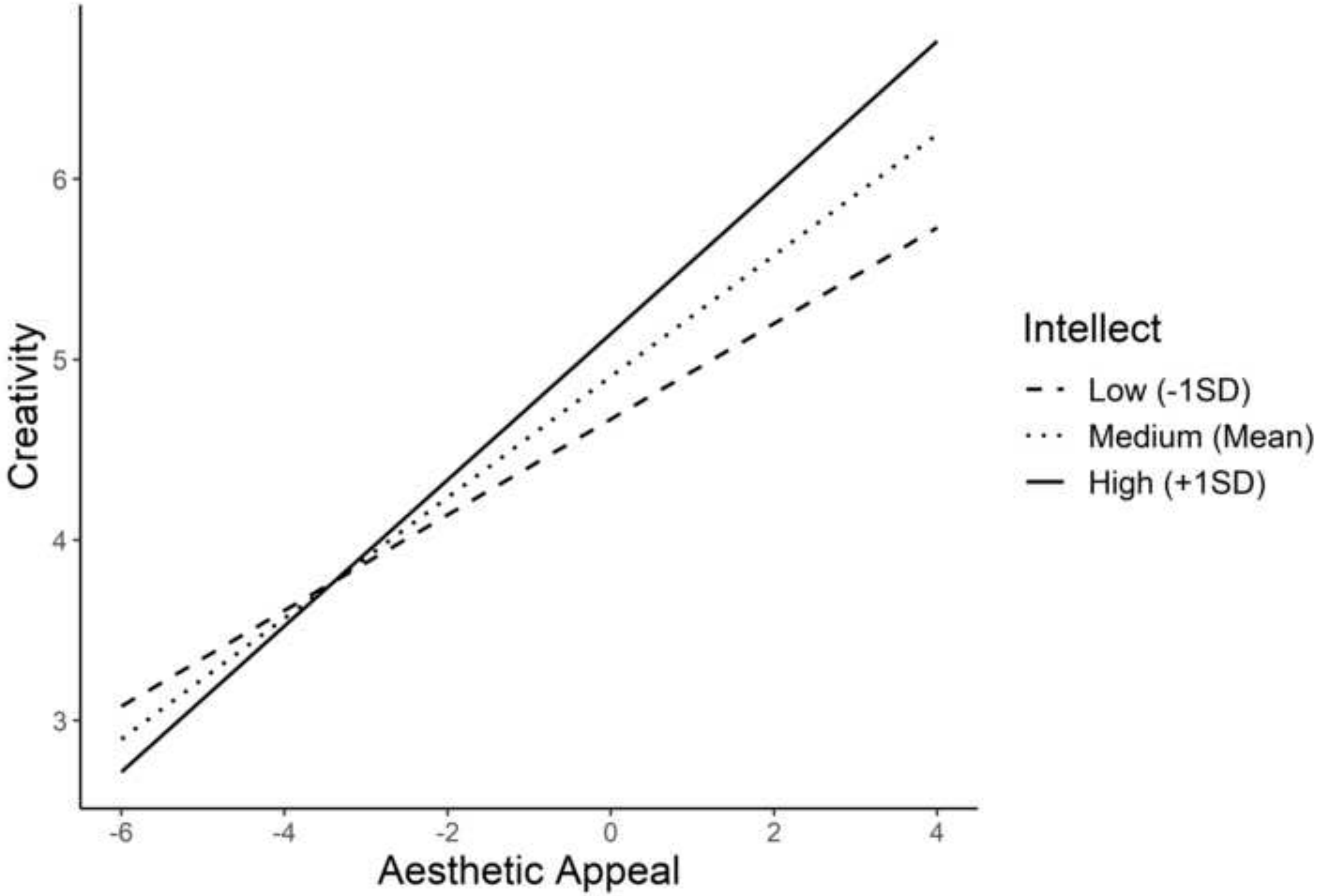

Figure 3. Simple slopes showing the interactions between awe-proneness as the moderator and aesthetic appeal and surprise as the predictors.

Click here to access/download;Figure;Figure 3_Awe_Interactions.png

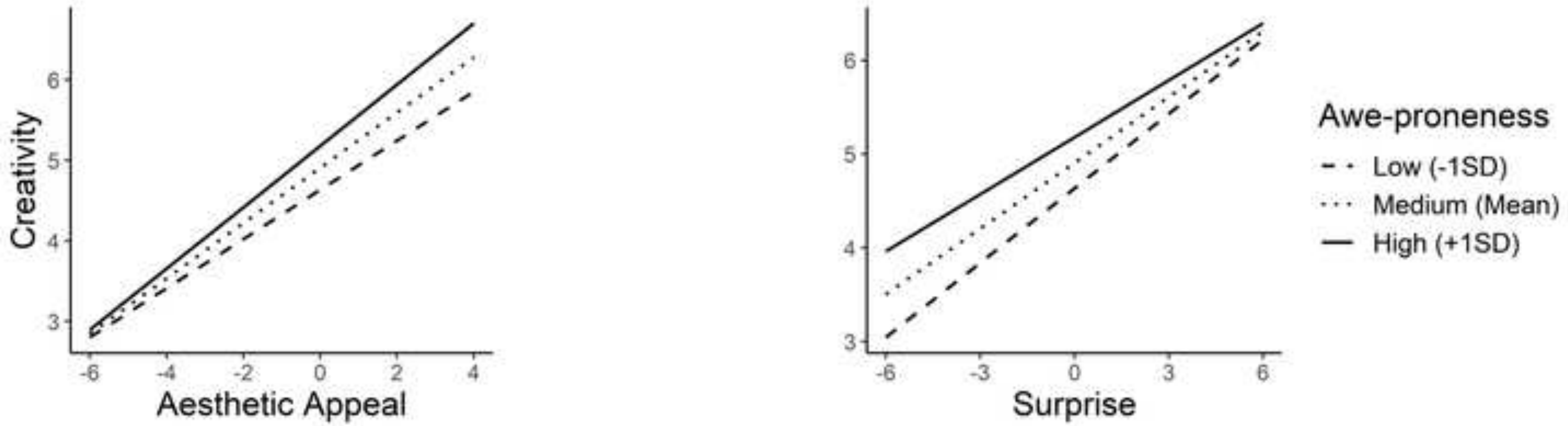

Figure 4. Simple slopes showing the interactions between curiosity as the moderator and aesthetic appeal and surprise as the predictors.

Click here to access/download;Figure;Figure 4_Curiosity_Interactions.png

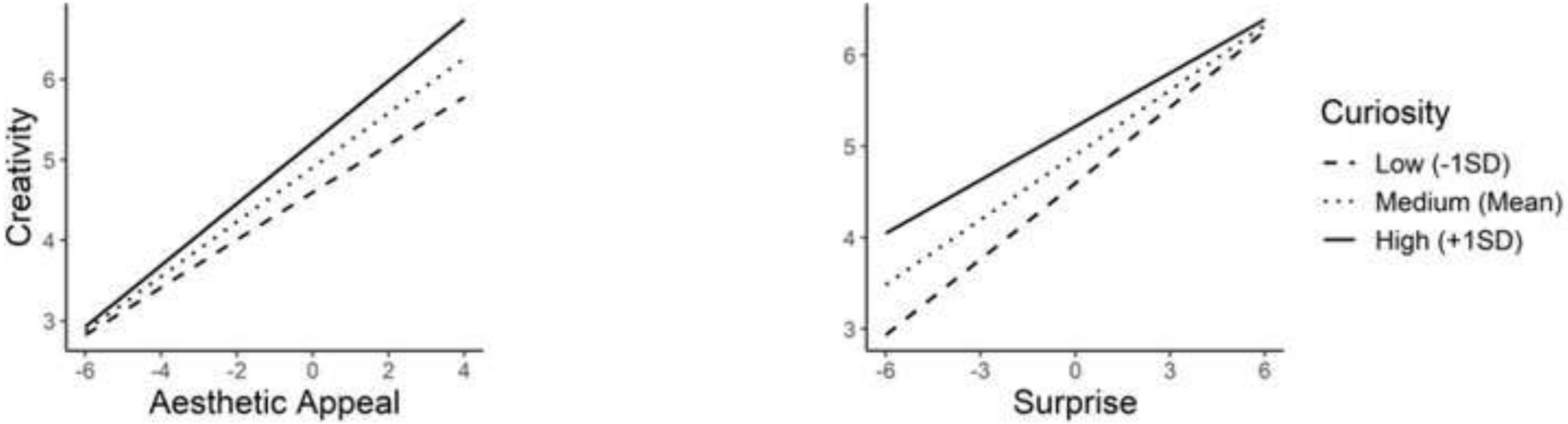

Beyond the words: Exploring individual differences in the evaluation of poetic creativity

Soma Chaudhuri[1], Alan Pickering[1], Maura Dooley[2], and Joydeep Bhattacharya[1]

[1]Department of Psychology, Goldsmiths, University of London

[2]Department of English and Creative Writing, Goldsmiths, University of London

*Corresponding author

Email: schau002@gold.ac.uk

Author Contributions

Conceptualization: Soma Chaudhuri, Joydeep Bhattacharya.

Data curation: Soma Chaudhuri.

Formal analysis: Soma Chaudhuri.

Methodology: Soma Chaudhuri, Alan Pickering, Joydeep Bhattacharya.

Supervision: Joydeep Bhattacharya.

Writing – original draft: Soma Chaudhuri

Writing - review and editing: Joydeep Bhattacharya, Alan Pickering, Maura Dooley

Authors have no known conflict of interest to disclose.

Abstract

Poetry is arguably the most creative expression of language that can evoke diverse subjective experiences, such as emotions and aesthetic responses, in different individuals, subsequently influencing their judgment of the creativity of poem. This study investigated how personality traits, specifically o~~o~~penness, i~~i~~ntellect, a~~a~~we-proneness, and e~~e~~pistemic c~~c~~uriosity, shape the implicit associations between subjective experiences and the judgment of creativity ~~creative judgment~~ of ~~**a broad range**~~**thirty-six** ~~of~~ English language poems. One hundred and twenty-nine participants rated each poem across six dimensions: clarity, aesthetic appeal, felt valence, felt arousal, surprise, and overall creativity. First, we obtained a parsimonious model of creativity comprising aesthetic appeal, felt valence, and surprise as potential predictors of poetic creativity. Subsequently, using multilevel analysis, we investigated the interactions between the four personality traits and these three predictors. Among the personality traits, o~~o~~penness emerged as the primary moderator in predicting judgments of poetic creativity, followed by c~~c~~uriosity and a~~a~~we-proneness. Among the predictors, aesthetic appeal was moderated by all four personality traits, while surprise was moderated by o~~o~~penness, a~~a~~we-proneness, and c~~c~~uriosity. Valence, on the other hand, was moderated by o~~o~~penness only. Our findings provide novel insights into how individual differences influence the evaluation of the creativity of poems.

Keywords: poetry, creativity, evaluation, personality traits, moderation

Formatted: Font: Bold, Font color: Accent 6

Introduction

Poetry is one of the most creative forms of linguistic expression used since ancient times to communicate emotions, thoughts, and ideas. However, despite its unique status in human lives, ~~poetry and its creative~~ evaluation of- creativity of poems has not been adequately characterized. The inherent subjectivity of poetry as an abstract form of literary art may be a possible reason for this limitation. ~~One possible reason for this limitation is the inherent subjectivity of poetry as an art form.~~ The adage "Beauty is in the eye of the beholder," (sourced from Margaret Wolfe Hungerford's novel "Molly Bawn" (1878)), aptly captures the subjective nature of aesthetics which also holds true for poetry. Individuals may possess divergent perspectives on creative potential of a particular poem, influenced by their subjective experiences. Readers comprehend the same poem differently depending on their knowledge and perceptual ability which leads to an idiosyncrasy in the assessment of its creativity. ~~The phrase "beauty is in the eye of the beholder" has long been used to describe the subjective nature of aesthetics. This adage holds particularly true for poetry, as different individuals may hold contrasting views on the creativity of a particular poem based on their subjective experiences. Poetry, being an abstract and intangible form of literary art, accentuates this subjectivity.~~ What one person perceives as creative, and captivating may be ordinary or unappealing to another. ~~As the creative evaluation of any product involves both the attributes of the product itself and the characteristics of the evaluator,~~ Therefore, individual differences in personality traits of readers are likely to cause variations in their assessments, subsequently influencing their overall creativity judgment of poetry. ~~we hypothesized that the assessment of poetic creativity would depend on the reader's personality traits and their subjective experiences with the poem. Therefore, this~~This study

Formatted: Font: (Default) Times New Roman, 12 pt

Formatted: Font: (Default) Times New Roman, 12 pt

Formatted: Font: (Default) Times New Roman, 12 pt

Formatted: Font: (Default) Times New Roman, 12 pt

Formatted: Font: (Default) Times New Roman, 12 pt

Formatted: Font: (Default) Times New Roman, 12 pt

Formatted: Font: (Default) Times New Roman, 12 pt

Formatted: Font: (Default) Times New Roman, 12 pt

Formatted: Font: (Default) Times New Roman, 12 pt

investigated how readers' internal models based on their personality attributes would impact their subjective feelings and experiences of reading poems while assessing poetic creativity.

~~how individual differences in readers' personality traits influence the assessment of a poem's creativity.~~

~~Readers comprehend the same poem differently depending on their knowledge and perceptual ability. What one person perceives as creative, and captivating may be ordinary or unappealing to another. When assessing a complex and nebulous construct like creativity, individuals' subjective experiences become more distinctively diverse and idiosyncratic compared to more generic constructs such as preference or liking. Therefore, individual differences in personality traits of readers are likely to cause variations in their assessments, subsequently influencing their overall creativity judgment of poetry. This study focused on how readers' internal models based on their personality attributes would impact their subjective feelings and experiences of reading poems while assessing poetic creativity.~~

Personality traits are basic dimensions on which people differ, reflecting their characteristic patterns of thoughts, feelings, and behaviours with consistency and stability (1,2). Several studies (3,4,5,6) have investigated the link between personality traits and creativity. Significant positive correlations have been observed between different measures of creativity and Big Five personality traits (7,8,9), especially openness to experience and extraversion (10,11,12). A meta-analysis (4) found o~~O~~penness to experience and e~~E~~xtraversion to be the most prominent personality traits associated with creativity in the Arts and Sciences.

When assessing creativity, raters often form their own mental criteria, which can vary depending on their personal preferences and individual differences (13). For example, recent research (14) suggests that o~~O~~penness to experience is associated with a higher discernment

Formatted: Font color: Auto

Formatted: Indent: First line: 0.5"

ability to distinguish creative from uncreative responses. Another study (15) highlights how an individual's consideration of the novelty and usefulness of creativity task responses is influenced by contextual factors and individual differences, such as o~~o~~penness and i~~i~~ntellect, in overall creativity judgment. These studies focused primarily on the relationship between personality traits and various creative idea-generation processes, such as divergent thinking, everyday creative behaviour, creative achievement, and self-rated creativity. However, the influence of personality traits on the evaluation of creativity of poetry has not been adequately explored. Of note, some studies (16,17,18) have found that individual differences in visual imagery abilities, ambiguity tolerance, awe-proneness, and nostalgia-proneness predict the aesthetic appeal of specific forms of poems like haiku and sonnets. In the present study, we addressed how readers' personality traits interact with predictors selected from five factors - clarity, aesthetic appeal, felt valence, arousal, and surprise – in evaluating the creativity of ~~a broad range of~~ English language poems. Specifically, we focused on four personality traits – o~~o~~penness, i~~i~~ntellect, a~~a~~we-proneness, and e~~e~~pistemic c~~c~~uriosity. We examined the moderating effects of these traits on the factors that predict the creativity judgment of a poem. In the subsequent sections, we provide a brief overview of these potential predictors, the personality traits themselves, and their prospective roles in evaluating creativity.

Clarity~~: It~~ refers to whether the text is lucid, understandable, and comprehensible to the readers. Any valued written communication, like poetry, is functional when readers comprehend it; clarity is a relevant dimension for evaluating the creativity of a poem (19).

Aesthetic appeal~~: It~~ refers to the artistic features, styles, and concepts of ~~evaluation of~~ any artwork.~~ involving aesthetic appreciation, frequently termed as aesthetic appeal~~. Research on the psychology of creativity and aesthetics has utilised stimuli like paintings and visual art

Formatted: Font: (Default) Times New Roman, 12 pt

Formatted: Font: Not Italic

Formatted: Font: Not Italic

(20,21,22,23,24), music (25, 26,27,28,29,30,31), films (32,33,34), poems (16,17,18,35,36,37,38,39).~~,~~ ~~.~~Previous empirical studies on poetry have primarily investigated aesthetic appreciation focusing on two broad aspects: (i) the objective properties of a poem and (ii) the subjective experiences during the perception of a poem. The former approach examines textual elements, e.g., rhythm, rhyme, meter (36, 40),~~,~~ metaphors (35~~)~~, ~~(~~41,42,43), and phonological constructs such as words and phrases (44, 45). The latter approach explores empathic reactions and emotional involvement (46), perceived emotional valence and vividness in imagery (16) cognitive and emotional ambiguity (e.g., awe and nostalgia) (38,17), openness to experience, visual imagery abilities, felt valence (17), expertise (47), gender and ethnicity (48). However, the potential interactions between these two approaches and how readers' characteristics influence their subjective evaluation of creativity remain unclear.

Felt valence~~:~~ refers to the extent to which an emotion felt by the perceiver is positive or negative, whereas arousal refers to its intensity, i.e., the strength of the associated emotional state. **According to the two-dimensional circumplex model of emotion, emotional states are represented along two orthogonal dimensions: valence (pleasure-displeasure: horizontal axis) and arousal (degree of arousal: vertical axis) (49). Poetry evokes strong emotions (50) and emotional states can influence creativity evaluation (51). A recent study suggests that poetry's content and prosodic features can evoke basic emotions, while the reader's intellectual evaluation of a poem evokes a complex aesthetic emotion that combines a basic emotion with their assessment of the poem (52). ~~As emotional states can influence creativity evaluation (51), we considered both dimensions of emotion – valence and arousal.~~ Of note, perceived and felt emotions may be distinct (53~~)~~,(54).~~(52,53)~~ ~~because~~ Perceived emotions are the emotions evoked by the stimuli whereas felt emotions are the emotions felt by the perceivers. In our study, we**

Formatted: Font: Not Italic

Formatted: Font: (Default) Times New Roman, 12 pt, Not Bold, Font color: Auto

Formatted: Font: (Default) Times New Roman, 12 pt

Formatted: Font: (Default) Times New Roman, 12 pt, Bold

Formatted: Font: (Default) Times New Roman, 12 pt

Formatted: Font: (Default) Times New Roman, 12 pt, Bold, Font color: Accent 6

Formatted: Font: (Default) Times New Roman, 12 pt

focused on the felt emotions, i.e., the emotions felt by the reader while reading the poem, rather than the perceived emotion, i.e., the emotion recognized in the poem. Therefore, felt valence reveals the extent to which the readers felt positive or negative emotions while reading the poems, whereas felt arousal here reveals how intense it was felt by the readers.~~emotions evoked by the stimuli may not always induce a feeling of identical emotions to the perceivers. In our study, we focused on the felt emotion, i.e., the emotion felt by the reader while reading the poem, rather than the perceived emotion, i.e., the emotion recognized in the poem.~~

Surprise is usually a short-lived emotion ~~Surprise: ~~elicited by events that deviate from an established schema (55~~)~~, ~~(~~56, ~~)~~(57). Of note, a schema, in this sense, refers to a component of the organism's knowledge structure, activated by a specific stimulus (58). Surprise is recognized as a key predictor of creativity (59~~)~~,~~(~~60) ~~Surprise is considered as a stronger predictor of creativity ~~and is also a robust predictor of aesthetic judgment of artwork (61). ~~than value after controlling for originality (59), which supports the 3-criterion definition of creativity (58). Further, it is also a robust predictor of aesthetic judgment of artwork (60). ~~In the context of our study, the term "surprise" specifically denotes the degree to which there is a sudden and unexpected change in the context or theme of the poem.~~In poetry, surprise offers an unexpected shift of concept or expectancy violation of readers.~~

Openness and Intellect

~~It ~~Openness to experience is a broad range of traits, from intellectual abilities to aesthetic and artistic interests (62~~)~~,~~(~~63~~)~~,~~(~~64) ~~(57,58,59)~~ and is most robustly associated with measures of creativity (65)~~(64)~~. It influences vivid fantasy (66), artistic sensitivity, novelty in

Formatted: Font: Bold, Font color: Accent 6, English (India)
Formatted: Font: (Default) Times New Roman, 12 pt
Formatted: Font: Bold, Not Italic, Font color: Accent 6
Formatted: Font: (Default) Times New Roman, 12 pt
Formatted: Font: Bold, Font color: Accent 6
Field Code Changed
Formatted: Font: (Default) Times New Roman, 12 pt
Formatted: Font: (Default) Times New Roman, 12 pt, Bold, Font color: Accent 6
Formatted: Font: (Default) Times New Roman, 12 pt
Field Code Changed
Formatted: Font: Bold
Formatted: Font: Bold
Formatted: Font: Bold, Font color: Accent 6
Formatted: Font: (Default) Times New Roman, 12 pt
Formatted: Font: Bold, Font color: Accent 6
Formatted: Font: (Default) Times New Roman, 12 pt
Formatted: Font: Bold, Font color: Accent 6
Formatted: Font: (Default) Times New Roman, 12 pt
Formatted: Font: Bold, Font color: Accent 6
Formatted: Justified
Formatted: Font: Bold, Font color: Accent 6
Field Code Changed

artworks, aesthetic emotions (67), intellectual curiosity (68), and unconventional attitudes (65). o~~O~~penness/i~~I~~ntellect, though characterized as a single dimension of personality, can be differentiated into two major ~~aspects~~aspects: openness and intellect (69).~~: openness and intellect .~~ Openness reflects the tendency to engage with aesthetic and sensory information, both in perception and imagination.~~,~~ On the other hand, intellect is a dispositional individual difference variable related to intellectual performance, such as problem-solving, thinking, information search, learning, or creativity (62~~), (~~70).~~whereas intellect reflects the tendency to engage with abstract and intellectual information (61).~~ Therefore, we predicted that o~~O~~penness and i~~I~~ntellect would separately moderate the relationship between aesthetic appeal and creativity ratings of a poem. Specifically, we predicted that individuals with higher levels of o~~O~~penness would experience a stronger positive impact of aesthetic appeal on creativity ratings compared to those with lower levels of o~~O~~penness.

Formatted: Font: (Default) Times New Roman, 12 pt

Formatted: Font: (Default) Times New Roman, 12 pt

Field Code Changed

Individuals with higher o~~O~~penness are reported to be more sensitive and attuned to their feelings (71). However, excessive emotional processes can inhibit higher cognitive functions in individuals with higher o~~O~~penness (72). Therefore, we predicted that o~~O~~penness would moderate the relationship between felt emotions (both valence and arousal) and creativity. Specifically, individuals with higher levels of o~~O~~penness would perceive the positive impact of felt emotions on creativity rating as less significant compared to those with lower levels of o~~O~~penness. Acknowledging that i~~I~~ntellect is related to processing of ~~abstract~~ complex and intellectual ~~and intellectual~~ information (7~~),(~~73), we assume that individuals with high intellect might rely less on emotional cues in their evaluation. Therefore, our prediction was, i~~I~~ntellect would not moderate the relationship between felt emotions and creativity. In other words, the positive impact of aesthetic appeal on creativity would remain unaffected by the levels of intellect in individuals.

Formatted: Font: (Default) Times New Roman, 12 pt

Surprise, interpreted as a form of conflict, arises in response to "collative variables" like novelty, complexity, uncertainty, and conflict (74). **As surprise is often experienced when individuals encounter unexpected or schema-discrepant events** (55,(56)**, we assumed that the assessment of expectancy violations in the judgment of poetic creativity necessitates a substantial degree of cognitive engagement. Consequently, we predicted that both openness and intellect would moderate the relationship between surprise and creativity**Earlier research has shown that openness influences attitudes toward targets that violate established schemas (75). Assuming that evaluating expectancy violation in the judgment of poetic creativity requires intellectual engagement and cognitive mastery, we predicted that both openness and intellect would moderate the relationship between surprise and creativity. Our specific prediction was that the positive impact of surprise on creativity scores would be more pronounced in individuals with higher openness and intellect. These individuals may be more receptive to and intrigued by unexpected elements of a poem, leading to heightened creativity judgment in response to surprises encountered in poems.

Awe-proneness

Awe, classified as an epistemic emotion, is a distinct emotional response to perceiving a stimulus that is vast, both literally and figuratively, and requires cognitive accommodation (75)(75). Poetry is likely to elicit awe due to its rich information content (76). Dispositional aawe- proneness is significantly correlated with oopenness to eexperience (r = .49), indicating a link between awe-proneness and the tendency to push one’s intellectual boundaries (76). Further, higher dispositional awe has been associated with aesthetic engagement and a tendency to experience aesthetic chill (77). **Aesthetic chills are transient emotional responses to aesthetical stimuli** (78)**. They can be described as experiencing a**

Formatted: Font: (Default) Times New Roman, 12 pt, Font color: Accent 6

Formatted: Font: (Default) Times New Roman, 12 pt

Formatted: Font: (Default) Times New Roman, 12 pt

Field Code Changed

Formatted: Font: (Default) Times New Roman, 12 pt

Formatted: Font: (Default) Times New Roman, 12 pt

Formatted: Font: (Default) Times New Roman, 12 pt

Formatted: Font: (Default) Times New Roman, 12 pt

chill or wave of excitement when engaging with poetry or looking at a work of art (79). Since awe is linked to surprise and amazement and is interpreted as a passive, receptive mode of attention in response to the unexpected (80),75); see also (75), we predicted that the aawe-proneness would moderate the effect of aesthetic appeal and surprise on creativity scores of a poem. Specifically, we predicted that the impact of aesthetic appeal and surprise on creativity ratings would be much stronger in individuals with higher levels of aawe-proneness. Due to their disposition towards experiencing awe, these individuals may be more receptive and responsive to a poem's aesthetic qualities and unexpected elements, thereby attributing higher levels of creativity to such poems.

Epistemic Curiosity

Curiosity is an intense desire to explore novel, complex and uncertain events (81). It is associated with learning and thinking processes and linked to various constructs such as interest, surprise, confusion, and awe (82,)(83)(73,74). CIt uriosity can be categorized into two broad types: pperceptual ceuriosity and eepistemic ceuriosity ; where pperceptual ceuriosity leads to increased perception of stimuli, and eepistemic ceuriosity is defined as a "drive to know" (84). EEpistemic curiosity motivates individuals to engage in exploratory behaviours to bridge the gap between their existing knowledge and their desire for further understanding (85),(86),(87)(77,78,79). Also, highly open individuals tend to be curious about the world, (88),(89),(90),(91)(80,81,82,83). Therefore, we predicted that eepistemic ceuriosity would significantly moderate the relationship between aesthetic appeal, surprise, and creativity. Specifically, we predicted that the positive impact of aesthetic appeal and surprise on creativity scores would be more pronounced in individuals with higher levels of eepistemic ceuriosity. These individuals, driven by their curiosity, would be more inclined to

Formatted: Font color: Text 1

Formatted: Font: (Default) Times New Roman, 12 pt

appreciate the aesthetic qualities and unexpected elements in a poem, thus attributing higher levels of creativity to such poems.

Materials and methods

~~**Participants**~~

~~Using G*power (version 3.1; (92), we found that a minimum sample size of 92 would be required to achieve an 80% power for a medium effect ($f^2 = 0.15$) with squared multiple correlation $\rho^2 = 0.13$. We recruited 129 healthy adult participants via Prolific®. As the task lasted approximately one hour, we excluded 30 participants who exceeded a two-hour time limit. Additionally, three participants were eliminated from the analyses due to their identical responses on the subjective rating measures across the poems. Our final sample consisted of 96 participants (32 males, 63 females, 1 preferred not to say; mean age = 31.94 years, SD = 13.09). Therefore, our final sample had adequate statistical power.~~

~~We described the main experimental procedures for this research to the participants. They were also informed that their data will be treated with full confidentiality (the study complied with the General Data Protection Regulation), and that, if published, it would not be identifiable as theirs. All participants provided informed consent (online) prior to data collection. Participants received a small cash incentive for participating in the study. Data were accessed for research purposes after the completion of the data collection which took place from 27 January 2022 to 23 June 2022.~~

Materials

Initially, we selected 108 poems from various online resources, including the Poetry.org (http://www.poetry.org/), the Poetry Foundation

Formatted: Font: (Default) Times New Roman, 12 pt, Bold, Font color: Accent 6

Formatted: Font: (Default) Times New Roman, 12 pt

Formatted: Font: Bold, Font color: Accent 6

Formatted: Font: (Default) Times New Roman, 12 pt

Formatted: Left

Formatted: Font: (Default) Times New Roman, 12 pt, Bold, Font color: Accent 6

Formatted: Font: (Default) Times New Roman, 12 pt

Formatted: Font: (Default) Times New Roman, 12 pt, Bold, Font color: Accent 6

(https://www.poetryfoundation.org/), and the Academy of American Poets (https://poets.org/). These poems were subsequently rated for "surprise" on a scale of 1 to 7 by M.D, who is a Professor of English and Creative Writing with domain-specific expertise. Following this evaluation, we shortlisted 36 poems as the experimental stimuli: 18 with low surprise ratings (4 or lower) and 18 with high surprise ratings (6 or above). The chosen poems varied in structures, contents, lines, and word count (mean number of lines = 11, SD = 3.24; mean word count = 71.25, SD = 28.99). To represent a broad spectrum of English poems, we consciously chose not to limit our selection to a particular genre or form, like haiku or sonnets as done in previous studies (16, 17, (92).

Lexical diversity (LD) of a text refers to its lexical richness, indicating the range and variety of vocabulary deployed in a text, (93). , For computing the LD of the poems, we used the type-token ratio (TTR) method which calculates the ratio of unique words (types) to the total word count (tokens) (94). It ranges from 0 to 1: a higher TTR indicates a greater lexical diversity. The average lexical diversity across the poems is 0.77 with standard a deviation of 0.09, suggesting that, on average, about 77% of the words used in the poems are unique or different. Semantic diversity, on the other hand, refers to the range of contexts (i.e., semantic richness) in which words are used (95). To calculate the semantic diversity, we used divergent semantic integration (DSI) (http://semdis.wlu.psu.edu/), representing the mean semantic distance between all word pairs in a text; DSI varies from 0 to 1, where a higher score indicates a broader collection of divergent ideas. The average DSI of our stimuli is 0.80 with a standard deviation of 0.03, indicating a high degree of semantic variety (see Table S1 in the Supplementary section for details). ,

We carefully selected a set of 36 original English poems for our study. These poems were selected to encompass a broad range of characteristics, including varying lengths, word counts, genres, and contents. Instead of choosing exclusively short and strictly structured poems, we aimed to capture the diversity and richness of English poetry. Poems were selected from various popular online poetry resource sites, such as Poetry.org (http://www.poetry.org/), Poetry Foundation (https://www.poetryfoundation.org/), and Academy of American Poets (https://poets.org/). Table 1 shows the aforementioned features of the selected poems. All poems are available in the OSF.

The average (SD) number of lines in the selected poems was 11 (3.2), and the average (SD) word count was 71.3 (29). To ensure varied readability, we assessed the Gunning fog index of readability for each poem, and the average (SD) fog index score was 13.84 (8.8), indicating that, on average, the poems had a comprehensibility level equivalent to college-level reading (mean fog index > 12). Furthermore, we computed other metrics related to the poems' text characteristics, such as lexical diversity (LD) and divergent semantic integration (DSI). LD measures the variety of words used in a text, providing insights into the complexity, lexical richness, and readability level (85,86,87). The average (SD) LD score for our stimuli set was 14.30 (1.7). DSI captures how much a text incorporates semantically diverging ideas, with higher scores indicating greater divergence (96). The average (SD) DSI score for our stimuli set was 0.8 (0.03). Poems were selected from various popular online poetry resource sites, such as Poetry.org (http://www.poetry.org/), Poetry Foundation (https://www.poetryfoundation.org/), and Academy of American Poets (https://poets.org/). Table 1 shows the aforementioned features of the selected poems. All poems are available in the OSF.

Table 1. Details of the stimuli including genres, themes, number of lines and words, readability, lexical diversity, and divergent semantic integration scores (DSI).

Poem Title	Genre	Theme	No. of lines	No. of words	Gunning-Fog inde	Unique Word	Characters	Lexical Diversity (%)	DSI
Funeral Blues	Modernist	Grief	16	136	12.94	101	708	14.26	0.81
At the Same Time	Contemporary	Transience of our existence	10	39	17.65	31	203	15.27	0.81
The Supreme Moment	Contemporary	Powerlessness of humans	16	63	12.65	54	350	15.43	0.84
Peace	Modernist: War pc	Futility of war	8	26	6.74	23	128	17.97	0.78
A Peck of Gold	Modernist	Illusion	12	86	11.47	45	406	11.08	0.76
The Freedom of the Moon	Contemporary	Awe inspiring Nature	12	91	10.86	68	493	13.79	0.81
Stopping by Woods on a Snowy Evenin	Modernist	Contemplation	16	108	7.94	74	527	14.04	0.79
When you Come to me	Contemporary	Nostalgia	10	41	8.16	34	224	15.18	0.83
Sonnet 116	Romantic	Eternity of love	14	109	10.19	81	566	14.31	0.8
Apparently with no Surprise	Nature poetry	Transience of life	8	36	10.36	33	203	16.26	0.83
Will there really be a "Morning"?	Lyric	Wonder and curiosity	12	76	3.04	53	377	14.06	0.78
Unable are the Loved to die	Lyric	Immortality of love	6	26	18	19	135	14.07	0.8
How happy is the little Stone	Nature	Independence and contentment	10	46	26.23	40	271	14.76	0.84
Ah! Sun-flower	Romantic	Individualism	8	50	23.2	41	280	14.64	0.82
The Smile	Romantic	Love and deceit	16	106	42.38	54	473	11.42	0.75
Love after Love	Contemporary	Self-love and self-discovery	15	98	6.4	61	518	11.78	0.78
The Last Laugh	War poem	Brutality of war	15	90	3.7	71	570	12.46	0.84
Clown in the Moon	Lyric	Melancholy	8	48	12.1	37	241	15.35	0.78
Remember	Lyric	Love, death, remembrance	14	111	17.68	66	539	12.24	0.74
When You Are Old	Romantic	True love	12	100	40.4	68	506	13.44	0.79
Aedh wishes for the Cloths of Heaven	Romantic	Love and dreams	9	60	24.67	35	320	10.94	0.78
Memory	Lyric	Transience of youth and beauty	6	33	13.2	25	163	15.34	0.79
The Arrow and the Song	Lyric	Power of words and friendship	12	88	12.64	47	412	11.41	0.76
There Will Come Soft Rains	Lyric	War	12	91	19.96	67	486	13.79	0.81
Alone	Romantic	Solitude	12	80	17	54	391	13.81	0.78
Ozymandias	Romantic	Transience of power	14	111	10.68	85	614	13.84	0.83
Good-Night	Romantic	Longing for togetherness	12	75	7.13	56	401	13.97	0.78
A Lament	Lyric	Nostalgia, sadness	10	65	4.33	51	320	15.94	0.81
Alas! This Is Not What I Thought Life V	Lyric	Challenges of life	9	75	7.6	60	366	16.39	0.8
Fragment: A Wanderer	Romantic	Imagination	4	25	14.8	22	167	13.17	0.84
Fragment: Apostrophe to Silence	Romantic	Existential longing	9	69	15.36	56	370	15.14	0.82
MEDITATION XVII	Romantic	Interconnectedness of humanity	13	81	10.08	57	377	15.12	0.78
Nothing Gold Can Stay	Nature	Impermanence of beauty	8	40	5.33	32	194	16.49	0.82
My Heart Leaps Up	Nature	Ecstasy and joy with nature's beauty	9	61	12.86	42	247	17	0.75
I taste a liquor never brewed –	Lyric	Joy of Nature	16	81	10.78	67	460	14.57	0.84
If You Should Go	Romantic	Transience of love and life	8	44	9.71	36	226	15.93	0.76

Note. Char = Characters, DSI = Divergent Semantic Integration scores (96). The formula used for lexical diversity: (LD = number of unique words/ total characters).**Participants**

By using the G*Power software (v. 3.1.9.4), (96) we found that a minimum sample size of 98 was required to detect a medium effect size ($f^2 = 0.15$) in a multiple linear regression, assuming a significance level of 0.05 and a statistical power of 80%. The criteria we used are widely-used conventional figures when estimating sample sizes. Additionally, we used the 'samplesize_mixed' function in R, which estimates the required sample size for linear mixed models (two-level-designs), based on power-calculation for standard design and adjusted for design effect for 2-level-designs (https://strengejacke.github.io/sjstats/). For our multilevel model, which considered 96 cluster groups, and a small to medium effect size (Cohen's d = 0.3)-, with and36 observations per cluster group (level-2-unit), we determined that a total of 965

Formatted: Indent: First line: 0.5", Space After: 0 pt, Line spacing: Double

Formatted: Font: Bold, Font color: Accent 6

Formatted: Font: (Default) Times New Roman, 12 pt

Formatted: Font: Bold, Font color: Accent 6

Formatted: Font: (Default) Times New Roman, 12 pt

Formatted: Font: Bold, Font color: Accent 6

Formatted: Font: (Default) Times New Roman, 12 pt

Formatted: Font: Bold, Font color: Accent 6

Formatted: Font: (Default) Times New Roman, 12 pt

Formatted: Font: Bold, Font color: Accent 6

Formatted: Font: (Default) Times New Roman, 12 pt

Formatted: Font: Bold, Font color: Accent 6

Formatted: Font: (Default) Times New Roman, 12 pt

Formatted: Font: Bold, Font color: Accent 6

Formatted: Font: (Default) Times New Roman, 12 pt

Formatted: Font: Bold, Font color: Accent 6

Formatted: Font: (Default) Times New Roman, 12 pt

Formatted: Font: Bold, Font color: Accent 6

observations would be necessary. This translates to a minimum of 27 participants (965/36).

We recruited 129 adult participants via Prolific®, a participant-recruiting platform. As the task lasted approximately one hour, we excluded 30 participants who exceeded a two-hour time limit. Additionally, three participants were eliminated from the analyses due to their identical responses on the subjective rating measures across the poems. Our final sample consisted of 96 participants resulting in a total of 3456 observations, ensuring sufficient statistical power for our study. Participants (32 males, 63 females, 1 preferred not to say; mean age = 31.94 years, SD = 13.09) were fluent in English (self-reported) and from a variety of educational backgrounds holding at least a bachelor's degree in any discipline. Additionally, our sample represented a diverse range of professions, and ethnicities.

Participants were briefed about the experimental procedure, which involved the assessment of a set of English poems on a 7-point Likert scale (1 = extremely low; 7 = extremely high) across various constructs including clarity, aesthetic appeal, felt valence, arousal, surprise, and overall creativity. Additionally, participants were instructed to complete demographic and personality-related questions. We assured participants of the full confidentiality of their data, in compliance with the General Data Protection Regulation, and clarified that any published results would be non-identifiable. All participants provided informed consent (online) before to data collection. Participants received a cash incentive of £7.50 per hour. The data collection period spanned from 27 January 2022 to 23 June 2022, and the data were accessed for research purposes only after this period.

Procedure

The experiment was created using Qualtrics®, and the link was disseminated through Prolific®, a platform for participant recruitment. ~~The experiment was created using Qualtrics®, and the link was distributed via Prolific® for recruiting participants.~~ Participants received a broad overview of the study and comprehensive instructions for ratings. In the beginning, a sample poem was provided to facilitate a clearer understanding of the ~~rating~~ process. **Participants were given a minimum of 30 seconds to read each poem. Following this period, they were allowed to proceed to the rating task. They were asked to rate the poems on six dimensions in the following order: clarity, aesthetic appeal, felt valence, felt arousal, surprise, and creativity, using a 7-point Likert scale (1: "Extremely Low" and 7: "Extremely High"). There was no time limit imposed for rating the poems. Of note, the poems remained visible during the rating process.** ~~In each trial, Participants read a poem for 30 seconds and then rated it on six dimensions in the following order: clarity, aesthetic appeal, felt valence, felt arousal, surprise, and creativity. They provided all ratings on a 7-point Likert scale (1: "Extremely Low" and 7: "Extremely High").~~ A brief demographic survey was conducted once 36 trials were finished. Finally, participants completed a set of ~~questions on~~questions on ~~four~~ personality traits – Ten Item Personality Inventory (TIPI: (97), o~~o~~penness/i~~i~~ntellect (69), a~~a~~we-proneness (76), and e~~e~~pistemic c~~e~~uriosity (86). It took an hour on average to finish the whole experiment. The study protocol was approved by the local Ethics Committee of the Department of Psychology, Goldsmiths University of London.

Analysis

Formatted: Font: (Default) Times New Roman, 12 pt, Bold, Font color: Accent 6

Formatted: Font: (Default) Times New Roman, 12 pt, Bold, Font color: Accent 6

Formatted: English (India)

The primary aim of our study was to ~~investigate~~ explore how four personality traits—openness, intellect, awe-proneness, and epistemic curiosity—moderate the impact of significant potential predictors on poetic creativity. ~~the moderating influence of four personality traits - openness, intellect, awe-proneness, and curiosity - on the potential predictors of poetic creativity, such as clarity, aesthetic appeal, felt valence, arousal, and surprise.~~ First, we determined the ~~best~~ significant predictors ~~for~~ of ~~assessing t~~the ~~overall~~ creativity of poems. To accomplish this task, five maximum likelihood linear mixed models (predictor models) were executed using the *lme4* package (98) in R (version 4.0.3). The models were run hierarchically, with the five potential predictors (group mean centered) progressively included as fixed effects, creativity as the response variable, and participants as the grouping variable. We incorporated random effects intercepts for participants in the analysis. The best model fit results identified the potential predictors of poetic creativity.

Formatted: Font: (Default) Times New Roman, 12 pt

Formatted: Space After: 8 pt, Pattern: Clear

Formatted: Font: (Default) Times New Roman, 12 pt

Formatted: Font: (Default) Times New Roman, 12 pt

Formatted: Font: (Default) Times New Roman, 12 pt

Formatted: Font: (Default) Times New Roman, 12 pt

Formatted: Font: (Default) Times New Roman, 12 pt

The overall data visualisation confirmed that the response variable follows a normal distribution, and there is no significant multicollinearity among the independent variables (Variance Inflation Factor < 3). Furthermore, the reliability of the measurement was established by assessing the internal consistency across items (Cronbach's alpha = 0.~~87~~80; McDonald's omega (Total) = 0.88).~~, McDonald's omega hierarchical= 0.77). Of note, as hierarchical omega is used to correctly estimate the reliability of the general factor, controlling the variance of the specific factors (95), we reported omega hierarchical (0.77) instead of omega total (0.91).~~

The dataset comprised 3456 responses and exhibited a common multilevel structure, with individual responses (Level-1) nested within participants (Level-2). The null model revealed that a significant 54% of the variance was attributed to the grouping variable (participants), affirming the necessity of employing a linear mixed model to accommodate the hierarchical nature of the data, over standard regression models. Furthermore, the

Formatted: Font: (Default) Times New Roman, 12 pt

Formatted: Indent: First line: 0.5", Line spacing: Double

Formatted: Font: (Default) Times New Roman, 12 pt

Formatted: Font: (Default) Times New Roman, 12 pt

intraclass correlation coefficient (ICC = 0.28), signifying the Level-2 clustering, revealed a significant level of clustering in the data. This implies that the Level-1 dependent variable (creativity) was not independent of the Level-2 grouping variable (participants). Hence, the use of multilevel modeling was considered appropriate.

~~Our data set, which included 3456 responses, had a common multilevel structure where individual responses (Level-1) were nested within participants (Level-2). The null model demonstrated that a substantial 54% variance was explained by the grouping variable (participants), supporting the use of a linear mixed model over standard regression models to account for the hierarchical structure of the data. Additionally, the intraclass correlation coefficient (ICC = 0.28), representing the Level-2 clustering, indicated a notable degree of clustering in the data, suggesting that the Level-1 dependent variable (creativity) was not independent of the Level-2 grouping variable (participants). Therefore, multilevel modeling was deemed appropriate.~~ To accurately estimate the within-group effects, the predictors were centered within clusters (CWC) before entering the models (99).~~(95).~~ Finally, we examined the impact of four personality traits (e.g., o~~o~~penness, i~~i~~ntellect, a~~a~~we-proneness, and e~~e~~pistemic curiosity) on potential predictors by conducting four separate linear mixed models (personality traits models). In these models, the personality traits and their interactions with the potential predictors were treated as fixed effects, with creativity as the response variable and participants as the grouping variable. To visualize the interaction effects of the moderators on the predictors, we followed the classical convention (100). Specifically, we plotted the mean value of the moderator and one standard deviation above and below the mean, allowing us to observe how the moderator influences the relationship between the predictors and creativity.

Formatted: Space After: 8 pt, Pattern: Clear

Formatted: Font: (Default) +Body (Calibri), 11 pt, Pattern: Clear

Results

Descriptive statistics

Descriptive statistics of the variables are shown in Table ~~2~~1, including the mean and standard deviation (SD) for each variable. The variables include five predictors (clarity, aesthetic appeal, felt valence, felt arousal, surprise), four personality traits (o~~o~~penness, i~~i~~ntellect, a~~a~~we-proneness, and e~~e~~pistemic c~~c~~uriosity), and the outcome variable (creativity). Distribution of variables are marginally left-skewed (excepting openness with skewness of 0.12), with very low kurtosis values~~ (excepting curiosity with kurtosis of 6.61)~~. Variation Inflation Factor (VIF<3) confirms~~ that there is no~~the absence of multicollinearity among the variables. Of note, throughout the article, epistemic curiositry is referred to as curiosity for the sake of clarity and ease of comprehension.

Table ~~2~~1. Descriptive statistics of the variables including mean, standard deviation, skewness, kurtosis, standard error, and variance inflation factor (VIF).

Variable	n	mean	sd	median	min	max	skewness	kurtosis	se	VIF
Clarity	3456	4.82	1.58	5	1	7	-0.46	-0.57	0.03	1.59
Aesthetic Appeal	3456	4.8	1.44	5	1	7	-0.48	-0.23	0.02	2.2
Felt Valence	3456	4.5	1.62	5	1	7	-0.41	-0.48	0.03	2.62
Felt Arousal	3456	3.86	1.73	4	1	7	-0.14	-0.92	0.03	2.01
Surprise	3456	3.78	1.68	4	1	7	-0.17	-0.92	0.03	1.69
Creativity	3456	4.91	1.38	5	1	7	-0.53	0.05	0.02	-
Openness	3456	5.02	0.74	4.9	3	6.4	0.12	-0.82	0.01	1.49
Intellect	3456	4.7	0.9	4.7	2.7	6.4	-0.04	-0.59	0.02	1.48
Awe-proneness	3456	5.11	1.14	5.17	1.83	7	-0.48	-0.04	0.02	1.76
Curiosity	3456	5.58	0.86	5.6	3.5	7	-0.19	-0.67	0.01	1.71

T

Variable	n	Mean	SD	Median	Min	Max	Skew	Kurtosis	SE	VIF
Clarity	3456	4.82	1.58	5	1	7	-0.46	-0.57	0.03	1.58
Aesthetic Appeal	3456	4.8	1.44	5	1	7	-0.48	-0.23	0.02	2.13
Felt Valence	3456	4.5	1.62	5	1	7	-0.41	-0.48	0.03	2.59
Felt arousal	3456	3.86	1.73	4	1	7	-0.14	-0.92	0.03	2
~~Surprise~~	~~3456~~	~~3.78~~	~~1.68~~	~~4~~	~~1~~	~~7~~	~~-0.17~~	~~-0.92~~	~~0.03~~	~~1.63~~
Openness	3456	5.02	0.74	4.9	3	6.4	0.12	-0.82	0.01	1.49
Intellect	3456	4.7	0.9	4.7	2.7	6.4	-0.04	-0.59	0.02	1.46
Awe proneness	3456	5.11	1.14	5.17	1.83	7	-0.48	-0.04	0.02	1.73
Curiosity	3456	5.52	1.03	5.6	0	7	-1.58	6.61	0.02	1.67
Creativity	3456	4.91	1.38	5	1	7	-0.53	0.05	0.02	-

~~T~~able ~~3~~ 2 shows the bivariate correlations between the predictors, personality traits, and creativity. Creativity was positively and significantly (all *p*<.01) correlated with five predictor variables: clarity (*r* = 0.42), aesthetic appeal (*r* = 0.66), felt valence (*r* = 0.60), arousal (*r* = 0.47), surprise (*r* = 0.52). Creativity was also significantly correlated (all *p*<.01), albeit weaker, with four personality traits: o~~O~~penness (*r* = 0.17), i~~I~~ntellect (*r* = 0.17), a~~A~~we-proneness (*r* = 0.20), and c~~C~~uriosity (*r* = ~~0.19~~0.22). No significant correlation was observed between o~~O~~penness and arousal (*r* = 0 .02, *p* = 0.27), i~~I~~ntellect and arousal (*r* = 0.03, *p* = 0.07), and i ~~I~~ntellect and surprise (*r* = -0.02, *p* = 0.33). Within personality measures, all were significantly correlated with each other, and the strongest correlation was observed between c~~C~~uriosity and a~~A~~we-proneness (*r* = 0.57, *p*<.01).

Table ~~3~~2. ~~Means, standard deviations, and~~Bivariate correlation coefficients for creativity, its predictors, and the personality measures of the readers.

Formatted: Font: (Default) Times New Roman, 12 pt

Formatted: Line spacing: Double

Formatted: Font: (Default) Times New Roman, 12 pt

Formatted: Font: (Default) Times New Roman, 12 pt

Formatted: Indent: First line: 0", Space After: 8 pt, Pattern: Clear

Variable	M	SD	1	2	3	4	5	6	7	8	9
1. Clarity	4.82	1.58									
2.Aesthetic Appeal	4.8	1.44	.57**								
3. Felt Valence	4.5	1.62	.54**	.68**							
4. Felt Arousal	3.86	1.73	.38**	.51**	.64**						
5. Surprise	3.78	1.68	.31**	.43**	.53**	.59**					
6. Creativity	4.91	1.38	.42**	.66**	.60**	.47**	.52**				
7. Openness	5.02	0.74	.09**	.12**	.04*	0.02	-.10**	.17**			
8. Intellect	4.7	0.9	.11**	.17**	.05**	0.03	-0.02	.17**	.43**		
9. Awe-proneness	5.11	1.14	.10**	.15**	.14**	.09**	.08**	.20**	.47**	.36**	
10. Curiosity	5.58	0.86	.12**	.16**	.13**	.07**	.08**	.22**	.33**	.47**	.57**

Note. M and *SD* are used to represent mean and standard deviation, respectively. * indicates *p* < .05. ** indicates *p* < .01.

Variable	M	SD	1	2	3	4	5	6	7	8	9
1. Clarity	4.82	1.58									
2.Aesthetic Appeal	4.8	1.44	.57**								
3. Felt Valence	4.5	1.62	.54**	.68**							
4. Felt Arousal	3.86	1.73	.38**	.51**	.64**						
5. Surprise	3.78	1.68	.31**	.43**	.53**	.59**					
6. Creativity	4.91	1.38	.42**	.66**	.60**	.47**	.52**				
7. Open	5.02	0.74	.09**	.12**	.04*	0.02	-.10**	.17**			
8. Intellect	4.7	0.9	.11**	.17**	.05**	0.03	-0.02	.17**	.43**		
9. Awe	5.11	1.14	.10**	.15**	.14**	.09**	.08**	.20**	.47**	.36**	
10. Curio	5.52	1.03	.10**	.14**	.09**	.04*	.05**	.19**	.34**	.47**	.57**

Note. Open = Openness; Awe = Awe-proneness; Curio = Curiosity; *M* and *SD* represent mean and standard deviation, respectively. ** indicates *p* < .01.

Parsimonious predictors

To determine the order of variable inclusion in the predictor model, we employed the forward selection method. The variables were added based on their correlation with the outcome variable, i.e., creativity. The variable with the highest correlation was included first in the null model, followed by the other variables in the descending order of their correlations with creativity, as shown in Table 32. Consequently, the predictor variables were entered into the model in the following order: aesthetic appeal, felt valence, surprise, arousal, and clarity. To compare five linear mixed models, various criteria such as Information Criteria , such as Akaike information criterion (*AIC*), Bayesian information criterion (*BIC*), (e.g., AIC, BIC),

Formatted: Font: Italic

Formatted: Font: Italic

the proportion of variance explained by fixed effects (R^2), and Likelihood ratio test statistic ($\Delta\chi^2$) were used, and the model comparison was presented in Table ~~4~~3. ~~Based on the results, the~~The model comprising aesthetic appeal, felt valence, and surprise demonstrated the ~~best~~ optimal fit and parsimony ~~with~~ as indicated by a significant likelihood ratio test statistic ($\Delta\chi^2$= 294.51, *p*<0.001) ~~and~~ along with a lower ~~value for the~~ Bayesian Information Criterion (*BIC*= 9069.6) compared to the ~~other~~ alternative models. Therefore, the model~~,~~ ~~including~~ that included aesthetic appeal, felt valence, and surprise, was selected as the most optimal ~~model~~ for predicting creativity.

Table ~~4~~3. Model comparison to identify the best model fit comprising aesthetic appeal, felt valence, and surprise.

Information Criteria	Null Model	Model 1	Model 2	Model 3	Model 4	Model 5
AIC	11160.26	9586.52	9325.25	9032.73	9016.77	9018.37
BIC	11178.7	9611.12	9356	9069.62	9059.81	9067.55
R^2	0	0.26	0.29	0.33	0.33	0.33
$\Delta\chi^2$		1575.73***	263.28***	294.51***	17.96***	0.4

Information Criteria	Null Model	Model 1	Model 2	Model 3	Model 4	Model 5
AIC	11160.3	9586.5	9325.2	9032.7	9016.8	9018.4
~~BIC~~	~~11178.7~~	~~9611.1~~	~~9356~~	~~9069.6~~	~~9059.8~~	~~9067.6~~
R^2	0	0.26	0.29	0.33	0.33	0.33
$\Delta\chi^2$		1575.73***	263.277***	294.514***	17.962***	0.401

Note: Aesthetic appeal, felt valence, surprise, arousal and clarity are included sequentially to Model 1 to Model 5; all models are compared hierarchically, i.e., Model 1 is compared to Null Model, Model 2 is compared to Model 1 and so on ; AIC = Akaike Information Criterion; BIC = Bayesian Information Criterion; R^2 = proportion of variation explained by fixed effects (101); $\Delta\chi2$ = Likelihood ratio test statistic for comparison of models.
Significance codes: 0 '***' 0.001 '**' 0.01 '*' 0.05 '.' 0.1 ' ' 1

Formatted: Line spacing: single

~~Note: Aesthetic appeal, felt valence, surprise, arousal and clarity are included sequentially to Model 1 to Model 5; all models are compared hierarchically, i.e., Model 1 is compared to Null Model, Model 2 is compared to Model 1 and so on ; AIC = Akaike's Information Criterion; BIC = Bayesian Information Criterion; R2 (m) = proportion of~~

~~variation explained by fixed effects (102); $\Delta\chi 2$ = Likelihood ratio test statistic for comparison of models. *** p < .001.~~

Formatted: Indent: First line: 0.5", Space After: 0 pt, Pattern: Clear (White)

Formatted: Indent: First line: 0.5"

The linear mixed model result for the best-fitting model ~~fit~~ is presented in Table ~~5~~4. Aesthetic appeal was found to be the best predictor (b = 0.34, SE = 0.02, t = 22.14, p<0.001), indicating a significant positive relationship with creativity. Following that, surprise significantly influenced creativity (b = 0.23, SE = 0.01, t = 17.54, p<0.001), showing a positive association with creativity. Felt valence, although demonstrating a relatively weaker but still significant effect on creativity (b = 0.16, SE = 0.01, t =11.56, p<0.001), was also positively associated with creativity. On the other hand, clarity did not significantly predict creativity (b = -0.01, SE = 0.01, t = - 0.63, p = 0.53) and was eliminated from subsequent analysis. Further, the model comparison revealed that felt arousal (b = 0.07, SE = 0.02, t =4.28, p<.001) did not contribute significantly to improving the model fit ($\Delta\chi^2$ =17.962 R^2 = 0.33) and was not considered to be the part of our parsimonious model. Of note, two alternative methods corroborated the appropriateness of this model. The first method involved a backward elimination approach, where the least correlated variable was progressively removed from the full model. The second approach involved variables entered in an arbitrary order (e.g., clarity, aesthetic appeal, felt valence, arousal, and surprise). Both methods consistently indicated that the model comprising aesthetic appeal, felt valence, and surprise yielded the best fit. Therefore, aesthetic appeal, surprise, and felt valence were identified as parsimonious predictors of poetic creativity. Next, we analysed the interaction of the four personality traits with these three predictors.

~~**Table 5. The linear mixed model results for the best model fit with aesthetic appeal, surprise, and felt valence as the predictors.**~~

**Random effects**						
Groups	Name		Variance			Std. Dev.
Participants	(Intercept)		0.54			0.74
Residual			0.72			0.85
Number of observations: 3456, groups: Participants, 96						
**Fixed effects**						
		Estimate	S.E.	df	t-value	p-value
(Intercept)		5.01	0.1	96	50.43	0
~~Expertise~~		~~-0.25~~	~~0.16~~	~~96~~	~~-1.58~~	~~0.12~~
Aesthetic appeal		0.31	0.02	3360	15.16	0
Felt valence		0.2	0.02	3360	10.27	0
Surprise		0.25	0.02	3360	14.72	0
Expertise*Aesthetic appeal		0.07	0.03	3360	2.33	0.02
Expertise*Felt valence		-0.08	0.03	3360	-2.76	0.01
Expertise*Surprise		-0.06	0.03	3360	-2.37	0.02
Grouping Variables						
Group		groups			ICC	
Participants		96			0.43	

~~Table 5. The linear mixed model results for the best model fit with aesthetic appeal, surprise, and felt valence as the predictors.~~

Table 4. The linear mixed model results for the best-fitting model, comprised of aesthetic appeal, surprise, and felt valence as the predictors of creativity judgment.

MODEL INFO:

Observations: 3456

Dependent Variable: Creativity

Type: Mixed effects linear regression

MODEL FIT:

AIC = 9032.7, *BIC* = 9069.6

Pseudo-R^2 (fixed effects) = 0.33

Pseudo-R^2 (total) = 0.62

Formatted: Font: Not Bold

Formatted: Font: (Default) Times New Roman, 12 pt, Bold

Formatted: Font: Bold

Formatted: Font: (Default) Times New Roman, 12 pt, Bold

Formatted: Font: Bold

Formatted: Line spacing: Multiple 1.08 li

Formatted: Font: (Default) Times New Roman, 12 pt, Bold

Formatted: Font: Bold

Formatted: Font: (Default) Times New Roman, 12 pt, Bold

Fixed Effects					
	Estimates	SE	df	t-value	p-value
Predictors					
(Intercept)	4.91	0.08	96	63.33	<0.001
Aesthetic appeal	0.34	0.02	3360	22.14	<0.001
Felt valence	0.16	0.01	3360	11.56	<0.001
Surprise	0.23	0.01	3360	17.54	<0.001
Random Effects					
Groups	Variance	SD			
Participants (Intercept)	0.56	0.75			
Residual	0.73	0.85			
ICC	0.43				
N(Participants)	96				
Observations	3456				
Marginal R^2	0.33				
Conditional R^2	0.62				

Note. ICC = Intraclass correlation coefficient

Moderating role of the personality traits

We explored the interaction of each of the four personality traits ~~–~~ – ~~o~~openness, ~~i~~intellect, ~~a~~awe-proneness, and ~~epistemic c~~curiosity ~~–~~ – with the three ~~potential~~ significant predictors of poetic creativity ~~–~~ – aesthetic appeal, surprise, and felt valence. Table ~~6~~ 5 displays the main effects of the moderators and their interactions with the predictors in the models involving ~~in~~ four personality traits. ~~models.~~

~~**Table 6. Personality traits models with main effects and interactions with the respective moderators.**~~ **Table 5. Models with Main Effects and Interactions of Personality Traits with Respective Moderators.**

Formatted: Font: (Default) Times New Roman, 12 pt

Formatted: Font: (Default) Times New Roman, 12 pt

Formatted: Font: (Default) Times New Roman, 12 pt, Bold

Formatted: Font: (Default) Times New Roman, 12 pt, Bold, English (United States)

Model	b	SE	t	p	Fit (R^2)
**Openness Model**					
Intercept	3.32	0.5	6.58	<0.001	
Openness	0.32	0.1	3.18	<0.001	
Aesthetic Appeal	-0.15	0.1	-1.46	0.14	
Felt Valence	0.47	0.1	4.89	<0.001	
Surprise	0.66	0.09	7.2	<0.001	
Openness*Aesthetic Appeal	0.1	0.02	4.83	<0.001	
Openness*Felt Valence	-0.06	0.02	-3.27	<0.001	
Openness*Surprise	-0.08	0.02	-4.76	<0.001	.36**
**Intellect Model**					
Intercept	3.67	0.39	9.35	<0.001	
Intellect	0.26	0.08	3.21	<0.001	
Aesthetic Appeal	-0.03	0.08	-0.35	0.72	
Felt Valence	0.22	0.08	2.8	0.01	
Surprise	0.31	0.08	3.92	<0.001	
Intellect*Aesthetic Appeal	0.08	0.02	4.51	<0.001	
Intellect*Felt Valence	-0.01	0.02	-0.74	0.46	
Intellect*Surprise	-0.02	0.02	-1	0.32	.36**
**Awe-proneness Model**					
Intercept	3.69	0.33	11.1	<0.001	
Awe-proneness Model	0.24	0.06	3.77	<0.001	
Aesthetic Appeal	0.17	0.06	2.71	0.01	
Felt Valence	0.18	0.06	2.96	<0.001	
Surprise	0.37	0.06	6.32	<0.001	
Awe-proneness*Aesthetic Appeal	0.03	0.01	2.67	0.01	
Awe-proneness*Felt Valence	0	0.01	-0.3	0.76	
Awe-proneness*Surprise	-0.03	0.01	-2.48	0.01	.37**
**Curiosity Model**					
Intercept	2.9	0.46	6.29	<0.001	
Curiosity	0.36	0.08	4.39	<0.001	
Aesthetic Appeal	0.06	0.1	0.63	0.53	
Felt Valence	0.03	0.09	0.37	0.71	
Surprise	0.5	0.08	5.99	<0.001	
Curiosity*Aesthetic Appeal	0.05	0.02	2.96	<0.001	
Curiosity*Felt Valence	0.02	0.02	1.43	0.15	
Curiosity*Surprise	-0.05	0.01	-3.27	<0.001	.38**

Variable	b	SE	t	p	Fit (R^2)
Openness Model					
Intercept	3.32	0.5	6.58	<0.001	
Openness	0.32	0.1	3.18	<0.001	
Aesth	-0.15	0.1	-1.46	0.14	
Felt valence	0.47	0.1	4.89	<0.001	
Surprise	0.66	0.09	7.2	<0.001	
Openness * Aesthetic Appea	0.1	0.02	4.83	<0.001	
Openness * Felt Valence	-0.06	0.02	-3.27	<0.001	
Openness * Surprise	-0.08	0.02	-4.76	<0.001	
					.362**
Intellect Model					
Intercept	3.67	0.39	9.35	<0.001	
Intellect	0.26	0.08	3.21	<0.001	
Aesthetic Appeal	-0.03	0.08	-0.35	0.72	
Felt Valence	0.22	0.08	2.8	0.01	
Surprise	0.31	0.08	3.92	<0.001	
Intellect *Aesthetic appeal	0.08	0.02	4.51	<0.001	
Intellect * Felt Valence	-0.01	0.02	-0.74	0.46	
Intellect * Surprise	-0.02	0.02	-1	0.32	
					.360**
Awe-Proneness Model					
Intercept	3.69	0.33	11.1	<0.001	
Awe-Proneness	0.24	0.06	3.77	<0.001	
Aesthetic Appeal	0.17	0.06	2.71	0.01	
Felt Valence	0.18	0.06	2.96	<0.001	
Surprise	0.37	0.06	6.32	<0.001	
Awe * Aesthetic Appeal	0.03	0.01	2.67	0.01	
Awe * Felt Valence	0	0.01	-0.3	0.76	
Awe * Surprise	-0.03	0.01	-2.48	0.01	
					.368**
Curiosity Model					
Intercept	3.5	0.39	8.86	<0.001	
Curiosity	0.26	0.07	3.63	<0.001	
Aesthetic appeal	0.12	0.09	1.38	0.17	
Felt Valence	0.09	0.09	1.03	0.3	
Surprise	0.52	0.08	6.58	<0.001	
Curiosity *Aesthetic Appeal	0.04	0.02	2.46	0.01	
Curiosity * Felt Valence	0.01	0.02	0.84	0.4	
Curiosity * Surprise	-0.05	0.01	-3.72	<0.001	

O~~O~~penness exhibited significant moderation effect on aesthetic appeal (*b* = 0.10, *SE* = 0.02, *t* = 4.83, *p*<.001), felt valence (*b* = -0.06, SE= 0.02, *t* = -3.27, *p*<.001), and surprise (*b* = -0.08, *SE*=0.02, *t* = -4.76, *p*<.001) (Fig 1). A significant moderation of i~~I~~ntellect was observed on aesthetic appeal (*b* = 0.08, *SE*= 0.02, *t* = 4.51, *p*<.001) with valence (*b* = -0.01, *SE* = 0.02, *t* = -0.74, *p* = 0.46) and surprise (*b* = -0.02, *SE* = 0.02, *t* = -1.00, *p* = 0.32) being unmoderated (Fig 2).

Figure 1. Simple slopes showing the interactions between o~~O~~penness as the moderator and aesthetic appeal, felt valence, and surprise as the predictors.

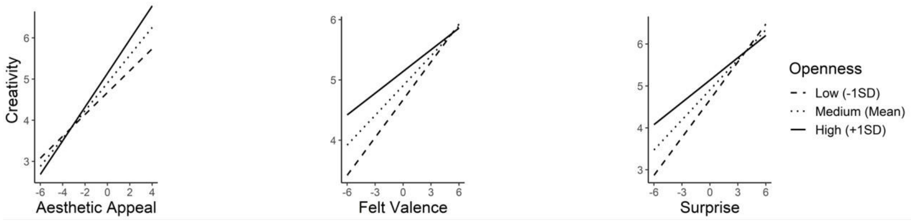

Figure 2. Simple slopes showing the significant interactions between i~~I~~ntellect as the moderator and aesthetic appeal~~, felt valence, and surprise~~ as the predictors.

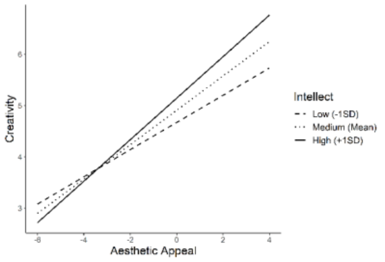

Formatted: Centered

Awe-proneness was found to be a significant moderator on the relationship between creativity and aesthetic appeal (*b* = 0.03, *SE*= 0.01, *t* = 2.67, *p* = 0.01), and surprise (*b* = -0.03, *SE* = 0.01, t = -2.48, *p*= 0.01), whereas no significant moderation with valence was observed (*b* = -0.00, *SE*= 0.01, *t* = -0.30, *p* = 0.76) (Fig 3). Finally, ~~epistemic c~~curiosity was found to significantly moderate aesthetic appeal (*b* = 0.04, *SE*= 0.02, *t* = 2.46, *p* = 0.01), and surprise (*b* = -0.05, *SE* = 0.01, *t* = -3.72, *p*<0.001), leaving felt valence unmoderated (*b* = 0.01, *SE* = 0.02, *t* = 0.84, *p* = 0.40) (Fig 4).

Figure 3. Simple slopes showing the interactions between ~~a~~awe-proneness as the moderator and aesthetic appeal~~, felt valence,~~ and surprise as the predictors.

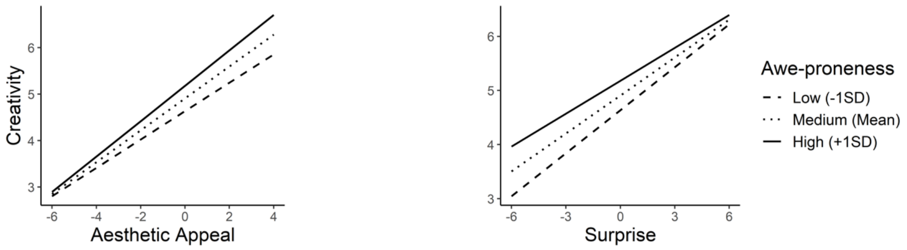

Figure 4. Simple slopes showing the interactions between ~~c~~curiosity as the moderator and aesthetic appeal~~, felt valence,~~ and surprise as the predictors.

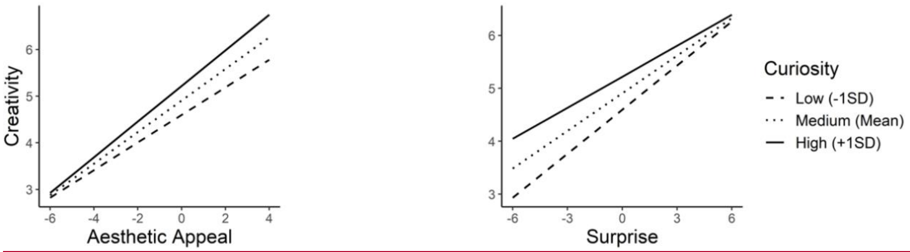

Consequently, all four personality traits exhibited significant moderation effects on both aesthetic appeal and surprise. However, distinct patterns were observed in the

moderation effects between these two predictors. The linear positive impact of aesthetic appeal on creativity was strengthened to a greater extent for higher values of the moderators. ~~In other words, the simple slopes of aesthetic appeal for higher values of all moderators consistently exceeded those for lower values of the moderators.~~ In contrast, the positive effect of surprise on creativity was attenuated for the higher moderator values~~; specifically, the simple slopes of surprise for higher moderator values consistently fell below those for lower moderator values, as indicated~~ The simple slopes analyses results are depicted in in Table ~~7~~6.

Table ~~7~~6. Results of simple slopes analyses of the predictors for the high and low levels of the moderators.

		High (+1 SD)				Low (-1SD)			
Predictor	Moderator	Est	*SE*	*t* -value	*p* -value	Est	*SE*	*t* -value	*p* -value
	Openness								
Aesthetic Appeal		0.41	0.02	19.51	<0.001	0.27	0.02	12.25	<0.001
Felt Valence		0.12	0.02	6.55	<0.001	0.21	0.02	9.92	<0.001
Surprise		0.18	0.02	10.55	<0.001	0.3	0.02	14.87	<0.001
	Intellect								
Aesthetic Appeal		0.41	0.02	19.31	<0.001	0.27	0.02	11.76	<0.001
	Awe-proneness								
Aesthetic Appeal		0.38	0.02	17.51	<0.001	0.31	0.02	15.18	<0.001
Surprise		0.2	0.02	11.73	<0.001	0.27	0.02	13.86	<0.001
	Curiosity								
Aesthetic Appeal		0.38	0.02	18.15	<0.001	0.3	0.02	13.91	<0.001
Surprise		0.2	0.02	11.48	<0.001	0.28	0.02	14.33	<0.001

A

Formatted: Font: Not Bold

Formatted: Indent: First line: 0.5"

		High (+1 SD)				Low (-1SD)			
**Predictors**	**Moderator Model**	**Est**	**SE**	**t-value**	**p-value**	**Est**	**SE**	**t-value**	**p-value**
	Openness Model								
Aesthetic Appeal		0.409	0.021	19.505	<0.001	0.266	0.022	12.253	<0.001
Felt Valence		0.12	0.018	6.552	<0.001	0.209	0.021	9.923	<0.001
Surprise		0.177	0.017	10.548	<0.001	0.301	0.02	14.866	<0.001
	Intellect Model								
Aesthetic Appeal		0.405	0.021	19.31	<0.001	0.265	0.023	11.755	<0.001
Felt Valence		0.153	0.019	8.093	<0.001	0.174	0.022	8.08	<0.001
Surprise		0.219	0.017	12.783	<0.001	0.247	0.022	11.378	<0.001
	Awe-Proneness Model								
Aesthetic Appeal		0.381	0.022	17.512	<0.001	0.305	0.02	15.183	<0.001
Felt Valence		0.156	0.02	7.901	<0.001	0.163	0.019	8.592	<0.001
Surprise		0.203	0.017	11.728	<0.001	0.265	0.019	13.862	<0.001
	Curiosity Model								
Aesthetic Appeal		0.378	0.022	17.286	<0.001	0.298	0.023	12.989	<0.001
Felt Valence		0.172	0.02	8.52	<0.001	0.146	0.022	6.592	<0.001
Surprise		0.186	0.018	10.457	<0.001	0.292	0.021	13.884	<0.001

rousal was not included in our parsimonious model as a potential predictor of creativity judgment of poetry. Nevertheless, we recognized the possibility that a predictor might not demonstrate main effect but could still show significant interaction when combined with another factor. Therefore, we examined the interaction effects on arousal. Results are as follows: openness interaction: b = -0.01, SE = 0.02, t = -0.46, p = 0.64; intellect interaction: b = 0.03, SE = 0.02, t = 1.86, p = 0.06; awe-proneness interaction: b = -0.01, SE = 0.01, t = -0.99, p = 0.32; curiosity interaction: b = 0.00, SE = 0.02, t = 0.17, p = 0.86). The findings indicated that influence of arousal on creativity remained unaltered by any of the four moderators.

Discussion

This study examined the influence of personality traits on the evaluation of creativity in English language poems. Specifically, we focused on four personality traits – openness, intellect, awe-proneness, and curiosity. First, we explored potential predictors of poetic creativity among five dimensions - aesthetic appeal, felt valence, surprise, arousal, and clarity – which were included hierarchically to model the overall creativity judgment. The results indicated that the model comprising aesthetic appeal, felt valence, and surprise was the most

Formatted: Font: (Default) Times New Roman, 12 pt

Formatted: Indent: First line: 0.5", Pattern: Clear (White)

Formatted: Font: (Default) Times New Roman, 12 pt

Formatted: Font: (Default) Times New Roman, 12 pt

Formatted: Font: (Default) Times New Roman, 12 pt

Formatted: Font: (Default) Times New Roman, 12 pt

Formatted: Font: 12 pt

parsimonious. Subsequently, we investigated how aesthetic appeal, felt valence, and surprise, as potential predictors of poetic creativity varied based on the rater's level of openness, intellect, awe-proneness, and curiosity. For this, we investigated the interactions between the four personality traits and these three predictors of poetic creativity. Among four personality traits, openness emerged as the most influential moderator in predicting judgments of poetic creativity. Regarding the three predictors, aesthetic appeal was moderated by all four personality traits, while surprise exhibited moderation effects by openness, awe-proneness, and curiosity. Valence, on the other hand, was solely moderated by openness.

In the present study, we found that both openness and intellect had shown distinct significant moderation effects on the aesthetic appeal of poems while judging poetic creativity. These findings support our initial prediction, indicating that the relationship between aesthetic appeal and creativity ratings varied depending on the levels of openness and intellect. Individuals with high scores in openness and intellect demonstrated a stronger positive relationship between aesthetic appeal and creativity ratings of poems relative to those with low scores in these traits. These results suggest that individuals with higher levels of imagination, a greater appreciation for aesthetic experiences, and a strong inclination towards art demonstrated a heightened sensitivity to the positive impact of aesthetic appeal on the judgment of poetic creativity relative to their low-scoring counterparts. Likewise, individuals who actively engage with abstract and intellectual information also recognized the positive influence of aesthetic appeal on creativity judgments to a greater extent than their low-scoring peers. Thus, consistent with prior research (69), we found a stable and distinct connection between openness, intellect, and aesthetic engagement.

Openness was found to have a significant moderating effect on the experience of felt valence and surprise. The simple slopes revealed that the positive influence of felt valence

and surprise on creativity ratings was greater for individuals with low levels of openness. This result indicates that individuals with lower levels of openness were more responsive to the impact of felt valence and surprise on creativity ratings than their higher counterparts. In contrast, intellect did not exhibit any moderating influence on either felt valence or surprise. This indicated that levels of intellect did not influence the assessment of creativity based on judgments of felt valence and surprise. The simple slopes confirmed that the relationship between creativity and felt emotion, as well as surprise, did not vary significantly across different levels of intellect. However, the significant moderation of intellect on aesthetic appeal indicated that individuals with greater abilities in processing complex intellectual information were more susceptible to the positive impact of aesthetic appeal rather than felt valence and surprise while judging the creativity of poems.

Acknowledging the possibility that a predictor may not show main effect but could still show significant moderation when combined with another factor, we tested the moderation effect of openness and intellect on arousal. We explored the significant moderation effect of awe-proneness, which represents an individual's propensity to experience awe-related emotions such as amazement, elevation, fascination, and wonder (69,26). Awe-proneness exhibited significant interactions with aesthetic appeal and surprise but not with felt valence. Highly awe-prone individuals demonstrated a more pronounced positive relationship between aesthetic appeal and creativity ratings than low awe-prone individuals. This result is reflected in the steeper gradient in slope for high awe-prone individuals than their low-scoring counterparts. This result is consistent with previous research suggesting that dispositional awe is associated with greater intellectual strengths, such as creativity, and theological strengths, such as an appreciation for beauty (95,96). In addition, some studies propose that awe-prone persons have a greater capacity to tolerate

ambiguity (76), which is required in art perception (97,98). Our study suggests that as the level of ambiguity increased with the rise in the aesthetic appeal of a poem, the higher tolerance for ambiguity among awe-prone readers facilitated their appreciation of poems. This, in turn, augmented the positive relationship between aesthetic appeal and creativity ratings of poems. Conversely, the relationship between surprise and creativity ratings exhibited a steeper gradient for individuals with low awe-proneness. This suggests that surprise had a more significant impact on creativity judgment among low awe-prone individuals compared to their higher counterparts. Considering that awe is characterized by vastness and accommodation as its main attributes, while surprise involves accommodation without vastness (75), we can infer that the positive influence of surprise on creativity judgment of poetry was less prominent among readers with higher levels of awe-proneness.

Similar to awe-proneness, epistemic curiosity exhibited significant interactions with aesthetic appeal and surprise, while no interaction effect was observed on felt valence. The simple slopes analysis revealed that individuals with high levels of curiosity displayed a pronounced positive relationship between aesthetic appeal and creativity ratings relative to their low-scoring counterparts. This indicates that individuals with a higher inclination towards acquiring knowledge-based information had a stronger impact of aesthetic appeal on creativity judgment than those with lower levels of curiosity. This result supports the notion that curious people feel more equipped to handle highly novel and complex stimuli, such as abstract poetry (107). The contribution of aesthetic appeal appreciation to the creativity judgment may have been more fostered by the intrinsic drive of highly curious readers to explore the unknown, in contrast to the individuals with lower levels of curiosity. Conversely, individuals with lower levels of curiosity displayed a steeper gradient in the positive relationship between surprise and creativity ratings. This result revealed that the positive influence of surprise on creativity was more pronounced among individuals with lower

curiosity compared to their high-scoring counterparts. The positive influence of high-surprise content in a poem in exploring creativity may have been less acknowledged by readers with a strong inclination for explorative inquisitiveness.

The comparable moderating effects of awe-proneness and curiosity on aesthetic appeal and surprise have several implications. Firstly, it suggests that emotions such as amazement, wonder, information-seeking curiosity, and knowledge hunger, when experienced at higher levels, have a stronger impact on the positive influence of aesthetic appeal appreciation while exploring poetic creativity. This indicates that individuals more prone to experiencing awe and possessing a higher level of curiosity are more receptive to the aesthetic aspects of poetry and perceive them as contributing significantly to its creativity. Conversely, when these emotions are experienced at lower levels, they exhibit a more prominent effect on the positive influence of surprise while exploring poetic creativity. This suggests that individuals with lower levels of awe-proneness and curiosity are particularly responsive to the element of surprise in poetry and perceive it as a significant contributor to creativity. Further, the finding that both awe-proneness and curiosity demonstrated identical moderation effects supports the notion that awe motivates curiosity (108). Overall, the comparable moderating effects of awe-proneness and curiosity suggest the intertwined nature of these emotions and their influence on aesthetic appeal and surprise in the context of creativity evaluation of poems.

Arousal was not included in our parsimonious model as a potential predictor of creativity judgment of poetry. Nevertheless, we recognized the possibility that a predictor might not demonstrate main effect but could still show significant interaction when combined with another factor. Therefore, we examined the interaction effects on arousal. However, the results revealed that none of the four moderators exhibited any interaction effects on arousal.

Results are as follows: openness interaction: b = -0.01, SE = 0.02, t = -0.46, p = 0.64; intellect interaction: b = 0.03, SE = 0.02, t = 1.86, p = 0.06; Awe-proneness interaction: b = -0.01, SE = 0.01, t = -0.99, p = 0.32; Curiosity interaction: b = -0.01, SE = 0.02, t = -0.87, p = 0.39). These results suggest that none of the selected personality traits of the readers had an impact their perception of stimulation or relaxation while evaluating a poem's creativity.

Furthermore, it is important to note that openness and intellect, although often being considered interconnected as part of a unified construct, demonstrated distinct patterns in their interactions with aesthetic appeal, felt valence, and surprise when assessing the creativity of poems. This finding reinforces the distinction between these two traits in the judgment of poetic creativity.

Discussion

Formatted: Font: 18 pt, Bold

The current study investigated the moderating effects of four personality traits — openness, iintellect, aawe-proneness, and ccuriosity - on the evaluation of creativity in English language poems: openness. Initially, we identified three predictors, out of the potential five, of creativity judgment of poems, as follows: aesthetic appeal, felt valence, and surprise. Subsequently, we investigated how these predictors varied concerning the participant's personality traits. We observed that individuals with higher levels of oopenness, intellect, curiosity, and awe-proneness gave more importance to aesthetic appeal, in descending order while assessing the creativity of poems. Notably, only the oopenness trait showed a moderating effect on felt valence, while the other traits did not demonstrate a significant effect.

The current study investigated the respective moderating role of four personality traits on the evaluation of creativity in English language poems: openness, intellect, awe-proneness, and curiosity. First, we explored the potential predictors of creativity judgment of

Formatted: Line spacing: Double

~~poems across five variables: clarity, aesthetic appeal, felt valence, arousal, and surprise. Aesthetic appeal was identified as the primary contributor to creativity judgment, followed by surprise and felt valence. Subsequently, we investigated how these predictors varied based on the rater's levels of personality. We observed that individuals with higher levels of openness, intellect, curiosity, and awe-proneness prioritized aesthetic appeal, in descending order while assessing the creativity of poems. Interestingly, only openness demonstrated impact on felt valence, with the other traits not showing a significant impact.~~

We identified distinct moderation effects of openness and intellect on the assessment of poetic creativity. ~~-~~Individuals with higher levels of both ~~openness and intellect~~ traits demonstrated a~~n elevated~~ pronounced emphasis on ~~prioritization of~~ a poem's aesthetic appeal ~~compared to those with lower levels of these traits~~ when evaluating poetic creativity, compared to those with lower levels of o~~o~~penness and i~~i~~ntellect. Despite being separate ~~distinct~~ traits with a correlation ranging from 0.3 to 0.5 (69), openness and intellect ~~displayed~~exhibited a shared tendency ~~-convergence~~ in ~~processing~~ appreciating a poem's ~~the~~ aesthetic appeal ~~of a poem~~. As ~~Considering~~ aesthetic experience ~~a~~is both style-related and art-specific, involving ~~both~~ cognitive and affective processing (102), individuals with higher levels of openness and intellect engaged more deeply ~~demonstrated increased engagement in~~ with both the cognitive and affective aspects during the evaluation process. We postulate that ~~T~~this heightened engagement led them to assign greater significance to the ~~the~~ aesthetic appeal of poems ~~when~~in their creativity assessments. ~~ingtheir creativity. Therefore, i~~In ~~alignment~~line with prior research (67), we found a stable and distinct connection between openness, Intellect, and aesthetic engagement; ~~where~~ both openness and intellect reflect a general ~~tendency~~ inclination towards aesthetic experiences—whether it be through sensory and aesthetic information processing (associated with openness) or abstract and complex semantic information processing (associated with intellect) (62). Open individuals, i.e., who

Formatted: Font: (Default) Times New Roman, 12 pt

Formatted: Font: (Default) Times New Roman, 12 pt

Formatted

Formatted: Indent: First line: 0.5"

were assumed to be ~~identified as~~ more ~~original,~~ unconventional, imaginative, and creative, (102,~~)(~~10) exhibited a more pronounced preference for ~~the~~ aesthetic appeal in their ~~of a poem in their~~ evaluation of poetic creativity than those with ~~compared to those with~~ lower levels.

Interestingly, we observed that individuals with lower levels of openness were more influenced ~~exhibited a greater influence of~~by felt valence i~~o~~n their creativity ratings of poems than those ~~compared to their~~ with higher levels of o~~o~~penness~~counterparts~~. ~~Emotional v~~Valence is a ~~crucial~~core aspect of ~~affect~~emotion (103,~~)(~~104,~~)(~~105), and aesthetic experience involve~~s~~s a cognitive process accompanied by evolving affective states~~continuously upgrading affective states~~, resulting in an aesthetic emotion (102). ~~In our study, t~~The ~~orthogonal~~ distinct moderating effects of openness on aesthetic appeal and felt valence in our study suggest that the aesthetic emotions evoked through ~~the~~ affective processing of poetry might differ ~~are distinct~~ from the emotional valence experienced during the reading of poetry ~~reading~~. Notably, levels of intellect did not exert any influence on felt valence.

Individuals with lower levels of openness were ~~more~~ found to be more influenced ~~responsive to the impact of~~by surprise in their ~~on~~ creativity ratings of poems than their higher counterparts. Surprise is often~~,~~ recognized as an interruption mechanism and a short-lived emotion (55), ~~and can be seen~~with an unclear positive or negative valence. Therefore, f~~F~~or more open individuals, ~~T~~the transient and ambiguous nature of ~~valence of~~ surprise ma~~y~~ight disrupt their affective states~~ of higher open individuals~~, leading to a diminished impact of surprise on their creativity judgment. In contrast, less open individuals ~~with lower levels of openness perceived~~ perceived surprise as a more ~~impactful and influential~~ significant factor in their evaluation of~~ng~~ poetic creativity than their higher counterparts. This finding speculates that the traditional ~~erefore, it could be argued that the consistency of the~~ 3-criterion definition of creativity (59), which includes ~~which considers~~ surprise as a key

Formatted

Formatted: Font: (Default) Times New Roman, 12 pt

Formatted

Formatted: Normal

Formatted

component, in the context of poetry ~~the third ingredient of creativity, aligns~~may resonate more ~~better with the perceptions of~~ with readers with lower levels of openness.Thus supporting the fact that openness/intellect is an aesthetically sensitive personality domain (67) and consistently serves as a predictor of both artistic creativity and aesthetic appreciation (4,26,~~(~~106) across a diverse form of art (20, ~~(~~64~~)~~, ~~(~~107), our study reveals that, individuals with higher openness and intellect, place particular emphasis on the positive impact of aesthetic appeal of poems when evaluating their creativity. However, our findings indicate distinct differences in the moderation effects of openness and intellect when assessing felt valence and surprise in poems during creativity evaluation emphasizing ~~s~~the nuanced distinction between openness and intellect (69~~)~~).

Awe-proneness, in our study, demonstrated significant interactions with aesthetic appeal and surprise, but ~~with~~ not with ~~observed effect on~~ felt valence~~, while assessing poetic creativity~~. Awe, a specific emotional response often triggered ~~to~~by beauty, is considered a key member of the self-transcendent emotions (108). In line with the model of apreciation of beauty and excellence (109), where the ability to perceive and appreciate beauty involves the experience of self-transcendent emotion like awe (108), our findings revealed that individuals with higher levels of awe-proneness placed greater emphasis on the aesthetic appeal of a poem when evaluating its creative potential. This suggests that readers predisposed to feeling awe might be more sensitive to the artistic and moral beauty of the poems (110). Further~~rmore~~, we observed ~~consistent with the~~a significant correlation (*r* = .4~~9~~7) between awe-proneness and o~~O~~penness, a finding corroborated by previous research ~~to Experience~~~~as also reported earlier (r = .49)~~ (111).~~-~~ O~~o~~ur moderation results suggest that dispositional awe is linked to creativity judgment, and appreciation for beauty (109~~)~~, ~~(~~112). ~~Conversely~~Interestingly, we observed that individuals with lower levels of awe-proneness were more influenced by surprise in their judgments of cretivity. ~~a more pronounced impact~~

Formatted: Font: (Default) Times New Roman, 12 pt
Formatted: Font: (Default) Times New Roman, 12 pt
Formatted: Font: (Default) Times New Roman, 12 pt
Formatted: Font: (Default) Times New Roman, 12 pt
Formatted: Font: (Default) Times New Roman, 12 pt
Formatted: Line spacing: Double
Formatted: Font: (Default) Times New Roman, 12 pt
Formatted: Font: (Default) Times New Roman, 12 pt
Formatted: Font: (Default) Times New Roman, 12 pt
Formatted: Font: (Default) Times New Roman, 12 pt
Formatted: Font: (Default) Times New Roman, 12 pt
Formatted: Font: (Default) Times New Roman, 12 pt
Formatted: Font: (Default) Times New Roman, 12 pt
Formatted: Font: (Default) Times New Roman, 12 pt
Formatted: Font: (Default) Times New Roman, 12 pt
Formatted: Font: (Default) Times New Roman, 12 pt
Formatted: Font: (Default) Times New Roman, 12 pt
Formatted: Font: (Default) Times New Roman, 12 pt
Formatted: Font: (Default) Times New Roman, 12 pt
Formatted: Font: (Default) Times New Roman, 12 pt
Formatted: Font: (Default) Times New Roman, 12 pt
Formatted: Font: Italic
Formatted: Font: (Default) Times New Roman, 12 pt
Formatted: Font: (Default) Times New Roman, 12 pt
Formatted: Font: (Default) Times New Roman, 12 pt
Formatted: Font: (Default) Times New Roman, 12 pt
Formatted: Font: (Default) Times New Roman, 12 pt
Formatted: Font: (Default) Times New Roman, 12 pt
Formatted: Font: (Default) Times New Roman, 12 pt
Formatted: Font: (Default) Times New Roman, 12 pt
Formatted: Font: (Default) Times New Roman, 12 pt
Formatted: Font: (Default) Times New Roman, 12 pt
Formatted: Font: (Default) Times New Roman, 12 pt
Formatted: Font: (Default) Times New Roman, 12 pt

~~of surprise on creativity judgment was observed among individuals with low awe-proneness compared to their higher counterparts. This finding aligns with p~~Previous research suggests that ~~indicating that the cognitive activity involved in~~ awe experiences do~~es~~ not require intensive ~~necessitate~~ effortful, controlled processing (111), and further, ~~, Additionally, the correlation between~~ dispositional awe is inversely correlated ~~and~~with the n~~N~~eed for c~~C~~ognitive c~~C~~losure ~~(r = -.39)~~ (76). Therefore, we suggest that ~~supports the notion that,~~ in the evaluation of poetic creativity, individuals with higher awe-proneness would prioritize ~~es~~ aesthetic appeal while ~~also~~ adopting a more passive and~~,~~ receptive stance towards unexpected elements in poetry ~~mode of attention in the face of the unexpected~~ (80). ~~The similar interaction patterns of openness and awe suggested that open people are more likely to experience awe (26).~~

Curiosity~~, a key dimension of curiosity (84) ,~~ exhibited significant moderating effects for ~~interactions with~~ aesthetic appeal and surprise, mirroring the ~~with no observed~~ interaction patterns of ~~with felt valence, similar to the interaction results of~~ awe-proneness. ~~Epistemic curiosity, one of the two dimensions of curiosity (a perceptual to epistemic axis and a specific to diversive axis) (84), drives behaviours aimed at acquiring new knowledge and experiences (116).~~ Individuals with heightened curiosity, driven by a desire for new knowledge and experiences (113), ~~-~~demonstrated a more pronounced ~~preference~~influence ~~for~~of the ~~the~~ aesthetic appeal of a poem on ~~in their judgment on~~ its ~~poetic~~ creativity ~~compared to those with lower curiosity levels~~. This ~~finding~~ reinforces~~d~~ the idea that curiosity is instrumental in facilitating ~~plays a crucial role in driving~~ aesthetic experiences and in the pursuit of ~~, motivating behaviours aimed at~~ understanding complex, abstract, and intellectually challenging stimuli (114). Additionally, our findings align with previous research indicating that individuals with high trait curiosity tend to find complex poems more comprehensible

Formatted: Font: (Default) Times New Roman, 12 pt
Formatted: Font: (Default) Times New Roman, 12 pt
Formatted: Font: (Default) Times New Roman, 12 pt
Formatted: Font: (Default) Times New Roman, 12 pt
Formatted: Font: (Default) Times New Roman, 12 pt
Formatted: Font: (Default) Times New Roman, 12 pt
Formatted: Font: (Default) Times New Roman, 12 pt
Formatted: Font: (Default) Times New Roman, 12 pt
Formatted: Font: (Default) Times New Roman, 12 pt
Formatted: Font: (Default) Times New Roman, 12 pt
Formatted: Font: (Default) Times New Roman, 12 pt
Formatted: Font: (Default) Times New Roman, 12 pt
Formatted: Font: (Default) Times New Roman, 12 pt
Formatted: Font: (Default) Times New Roman, 12 pt
Formatted: Font: (Default) Times New Roman, 12 pt
Formatted: Font: (Default) Times New Roman, 12 pt
Formatted: Font: (Default) Times New Roman, 12 pt
Formatted: Font: (Default) Times New Roman, 12 pt
Formatted: Font: (Default) Times New Roman, 12 pt
Formatted: Font: (Default) Times New Roman, 12 pt
Formatted: Font: (Default) Times New Roman, 12 pt
Formatted: Font: (Default) Times New Roman, 12 pt
Formatted: Font: (Default) Times New Roman, 12 pt
Formatted: pf0

~~easier to understand and, consequently, more interesting~~and engaging (115). **The contribution of aesthetic appeal appreciation to the creativity judgment may have been more fostered by the intrinsic behavioural traits of highly curious readers which compel them to delve more deeply into the unfamiliar aspects of the poems.** In contrast, **the influence of surprise on creativity judgment was more pronounced in those ~~among~~ individuals with lower levels of curiosity**, indicating that~~h~~ ~~. This outcome suggests that,~~ surprise, as a short-lived emotion and interruption mechanism (55), may interrupt the information-seeking tendencies of highly curious readers ~~in readers with higher levels of curiosity~~d ~~d~~uring their evaluation of poetic~~m~~ creativity.

~~Moreover, t~~The observed similarities in the interaction patterns ~~noted in~~of openness and curiosity ~~support~~ underscore a well-established ~~the consistent~~ link between openness and curiosity (89, ~~(~~116~~)~~, ~~(~~117~~)~~, ~~(~~118)~~119,120,121)~~, ~~(correlations ranging between r = .19 and r = .57),~~ indicating that individuals with high openness are more motivated to learn, ~~more~~ inclined to explore, and interested in acquiring information ~~(89), (119),(120),(121)~~. The~~i~~se tendenc~~y~~ies might enhance ~~ay contribute to the enhancement of~~ their ~~general~~ semantic knowledge (119)~~(122)~~, and subsequently, ~~influencing~~ their aesthetical experiences (114) and the judgment of poetic creativity. ~~Furthermore, the intertwined nature of awe and curiosity was evident in the parallel moderating effects of these emotions.~~ Our~~The~~ results also suggest ~~indicated~~ that awe-prone ~~people~~individuals are more curious~~, i.e., people who are higher in dispositional awe are on average higher in curiosity~~ (120), and that ~~also that~~ awe itself can stimulate ~~motivates~~ curiosity (121). Altogether, these suggest ~~Therefore, our result indicates~~ that higher levels of awe-proneness and curiosity might amplify the perceived ability to ~~ay enhance people's perceived ability to~~ comprehend complex stimuli like poetry (115).

Formatted ...

Formatted ...

Formatted ...

Field Code Changed ...

Field Code Changed ...

Formatted ...

~~Hence, the present study illustrates that, individuals with elevated levels of all four personality traits (e.g., openness, intellect, awe proneness, and curiosity) consistently recognized the aesthetic appeal of a poem as the most influential predictor of its creativity. Conversely, individuals with lower levels of all four personality traits regarded surprisingness in a poem as a positive factor influencing its creativity. Remarkably, felt valence consistently emerged as a positive contributor to the creativity of a poem, irrespective of the levels of the chosen personality traits.~~

Limitations

The current study is not without ~~has some~~ limitations ~~that should be acknowledged~~. First, we focused on felt emotions experienced by participants ~~, which refer to the emotions the participants felt~~ while reading ~~a~~ poems, rather than ~~. We did not assess~~ perceived emotions, which are the perceived ~~the emotions~~ emotional quality of the poems~~voked by the content of the poem~~. Perceived and felt emotions are not necessarily identical, as highlighted in studies on music (53)~~,~~(54)~~,~~(122) ~~(52,53,101)~~. We suggest that this is also likely to be the case for poems. For instance, ~~Likewise,~~ a poem with a 'sad' theme ~~while reading a poem, emotions evoked by the poem's content~~ may not necessarily induce ~~always elicit identical emotions in the reader; for example, a "sad" poem might not necessarily induce~~ sadness in the reader~~a sad feeling~~. Of note, ~~Previous~~ previous research has reported an association between perceived valence and aesthetic appeal of poetry (16). Therefore, ~~Future~~ future work could investigate perceived emotions' predictive power on a poem's creativity ~~explore whether perceived emotion would predict a poem's creativity and investigate~~ and the potential moderating role of traits like intellect. ~~in this relationship.~~ Second, we focused on trait-level personality characteristics rather than state-level personality features. However, ~~However, it is recognized that e~~contextualized personality traits are crucial ~~necessary to account~~ for capturing within-

Formatted: Indent: First line: 0.5", Line spacing: Double

Formatted: Font: (Default) Times New Roman, 12 pt

Formatted: Font: (Default) Times New Roman, 12 pt

Formatted: Font: (Default) Times New Roman, 12 pt

Formatted: Font: 18 pt, Bold

Formatted: Justified, Indent: First line: 0"

Formatted: Indent: First line: 0.5", Line spacing: Double

Field Code Changed

Field Code Changed

individual variability (123)(103),. Therefore, iindicating ncluding relevant state-level individual differences could provide a need for future studies to include more nuanced understanding ofstate-level within individual differences for a more comprehensive understanding variability inof poetry evaluation. Third, we did not examined the influence of context or appeal-based characteristics of poetry on the evaluation of creativity without controlling for various structural elements of poems such as other key features like structure, rhythm, form, and genre. WWe did not did not restrictt the poems to specific lengths, rhythmic patterns, or specific forms or genres, like sonnets, haiku, limericks, or others. Exploring the specific effects of genres and forms was not feasible due to the limited our study's limited number of poems in our study, so . NeverthelessHowever, we recognize acknowledge that the potential influence of these poem-based objective features inherent on to poems on the evaluation ofassessment of their creativity assessment could not be ruled outof poem cannot should not be overlookeddisregarded. Lastly, we allocated a fixed reading time of 30 seconds for each poem, which may not have been sufficient for participants to fully comprehend, synthesize and appreciate the nuances of every poem, especially for those less familiar with the poetic form. This time constraint may have influenced participants to rely more on their subjective impressions of the poem rather than engaging in a deeper analysis of its creative elements. This could result in evaluations that are more subjective or based on personal preferences rather than objective measures of creativity. In real-world settings, readers often take their time to read and reflect on a poem, potentially reading it multiple times before forming an opinion on its creativity. . Fourth, the representativeness of the selected poems may also be limited, potentially it is also worth mentioning that the chosen poems may not constitute a fully representative sample, which could limitimpacting the generalizability of our the results to a certain extentfindings. Fifth, concerning the diversity measures of the stimuli, **it is important to acknowledge that given the small word count of some of our poems and the**

Formatted: Font: Not Bold

Formatted: Font: (Default) Times New Roman, 12 pt

Formatted: Font: (Default) Times New Roman, 12 pt

Formatted: Font: (Default) Times New Roman, 12 pt

Formatted: Font: (Default) Times New Roman, 12 pt

Formatted: Font: (Default) Times New Roman, 12 pt

Formatted: Font: (Default) Times New Roman, 12 pt

Formatted: Font: (Default) Times New Roman, 12 pt

Formatted: Font: (Default) Times New Roman, 12 pt

implied limited vocabulary, the Type-Token Ratio (TTR) method might not yield reliable results due to constrained variability in word usage within small texts (124), (125). Sixth, **we assessed the variables using single item measures, a common practice in assessing aesthetics in visual art (126), (127), poetry (16),(17),(92,)(128), (129) and music (130). However, we also recognize the potential variability in individual interpretation of the questions remains unexplored. Employing multiple items for variable assessment could have offered psychometric advantages, particularly in enhancing reliability and validity (131). Finally, we acknowledge that the operationalization of 'creativity' is different from the more traditional view of creativity as a characteristic of individuals. Considering that creativity, as per the 4P Model (132), is defined through four lenses: person, product, process, and press, we strategically focused on the 'product' aspect of creativity, particularly in the context of poetry. We, here operationalized 'creativity' as the poem's creative potential. This choice was made to expand the understanding of creativity beyond the creator to the creation itself. Our approach is in line with numerous past studies which have investigated the creativity evaluation of various types of products/artefacts, such as ideas (15), musical compositions (133), (25), short stories (134), and product concepts (135), to name a few. This approach enabled us to explore the internal model of how individuals judge the creativity of poems, recognizing that such judgments may be inherently subjective and influenced by individual personality traits.**

Conclusions

In summary, our ~~present~~ study investigated ~~the influence of~~ how specific personality traits, namely openness, intellect, awe-proneness, and epistemic curiosity, influence the ~~on~~ evaluation~~ng~~ ~~the~~ of creativity of English language poetry. We focused on how these traits

Formatted: Font: Bold
Field Code Changed
Formatted: Font: Bold
Formatted: Font: (Default) Times New Roman, 12 pt, Not Bold, Font color: Auto
Formatted: Font color: Auto
Field Code Changed
Formatted: Font color: Auto
Formatted: Font: Bold, Font color: Accent 6
Field Code Changed
Field Code Changed
Field Code Changed
Formatted: Font: (Default) Times New Roman, 12 pt
Formatted: Font: (Default) Times New Roman, 12 pt
Formatted: Font: (Default) Times New Roman, 12 pt, Bold, Font color: Accent 6
Formatted: Font: (Default) Times New Roman, 12 pt, Bold, Font color: Accent 6
Formatted: Font: (Default) Times New Roman, 12 pt, Bold, Font color: Accent 6
Formatted: Font: (Default) Times New Roman, 12 pt
Formatted: Font: (Default) Times New Roman, 12 pt
Formatted: Font: (Default) Times New Roman, 12 pt
Formatted: Font: (Default) Times New Roman, 12 pt, Bold, Font color: Accent 6
Field Code Changed
Formatted: Font: Bold
Formatted: Font: Bold, Font color: Accent 6
Formatted: Font: (Default) Times New Roman, 12 pt, Bold, Font color: Accent 6

moderate the impact of three predictors - ~~Specifically, the study examined how personality traits such as openness, intellect, awe-proneness, and epistemic curiosity moderated the influence of~~ aesthetic appeal, felt valence, and surprise - ~~,~~ in ~~forming~~ forming a parsimonious model for evaluating poetic creativity. Among four traits, ~~We found that~~ openness exerted ~~had~~ the ~~strongest~~ most significant moderating effect ~~across~~ on all three predictors, and ~~.~~ ~~Furthermore,~~ among the predictors, aesthetic appeal was significantly moderated by all personality traits in assessing the creativity of poems. These results altogether demonstrate how specific personality traits moderate the ~~took precedence for individuals with elevated levels of openness, intellect, awe-proneness, and curiosity while~~ assessing the creativity of a ~~poem.~~

~~individuals with high levels of openness, intellect, awe-proneness, and epistemic curiosity were more likely to judge higher aesthetically appealing poems as more creative than individuals with lower levels of these traits. Despite~~ underlying model of creativity judgment of English poems, thereby explaining the variability in individual preferences and evaluations. ~~the apparent typical and idiosyncratic nature of an individual's preference for poems, these results show~~ ~~indicate~~ ~~that the underlying model for creativity judgment can be somewhat consistent across individuals, and~~~~individuals and~~ ~~specific personality traits influence this model explaining the individual variability.~~

Formatted: Font: (Default) Times New Roman, 12 pt

Formatted: Font: (Default) Times New Roman, 12 pt

Formatted: Font: (Default) Times New Roman, 12 pt

Formatted: Space After: 8 pt

Author Contributions

Conceptualization: Soma Chaudhuri, Joydeep Bhattacharya.

Stimuli selection and editing: Soma Chaudhuri, Maura Dooley

Data curation: Soma Chaudhuri.

Formal analysis: Soma Chaudhuri.

Methodology: Soma Chaudhuri, Alan Pickering, Joydeep Bhattacharya.

Supervision: Joydeep Bhattacharya.

Editing: Joydeep Bhattacharya, Alan Pickering, Maura Dooley

Data availability

All data and code are available in the OSF at:

https://osf.io/9mw7r/?view_only=07137f4871d146c790501f22bc7743d5

References

1. Diener E, Lucas RE. Personality traits. Gen Psychol Required Read. 2019;278.

2. Matthews G, Deary IJ, Whiteman MC. Personality traits. Cambridge University Press; 2003.

3. Barron F, Harrington DM. Creativity, intelligence, and personality. Annu Rev Psychol. 1981;32(1):439–76.

4. Feist GJ. A meta-analysis of personality in scientific and artistic creativity. Personal Soc Psychol Rev. 1998;2(4):290–309.

5. Feist GJ, Barron FX. Predicting creativity from early to late adulthood: Intellect, potential, and personality. J Res Pers. 2003;37(2):62–88.

6. Batey M, Furnham A. Creativity, intelligence, and personality: A critical review of the scattered literature. Genet Soc Gen Psychol Monogr [Internet]. 2006 Nov 1;132(4):355–429. Available from: https://doi.org/10.3200/MONO.132.4.355-430

7. DeYoung CG. Openness/intellect: A dimension of personality reflecting cognitive exploration. In: APA handbook of personality and social psychology, Volume 4: Personality processes and individual differences. Washington, DC, US: American Psychological Association; 2015. p. 369–99. (APA handbooks in psychology®.).

8. Kaufman SB, Quilty LC, Grazioplene RG, Hirsh JB, Gray JR, Peterson JB, et al. Openness to experience and intellect differentially predict creative achievement in the arts and sciences. J Pers [Internet]. 2016 Apr 1;84(2):248–58. Available from: https://doi.org/10.1111/jopy.12156

9. Silvia PJ, Nusbaum EC, Berg C, Martin C, O’Connor A. Openness to experience, plasticity, and creativity: Exploring lower-order, high-order, and interactive effects. J Res Pers [Internet]. 2009;43(6):1087–90. Available from: https://www.sciencedirect.com/science/article/pii/S0092656609001317

10. McCrae RR. Creativity, divergent thinking, and openness to experience. J Pers Soc Psychol. 1987;52:1258–65.

11. Tan C-S, Lau X-S, Kung Y-T, Kailsan RA. Openness to experience enhances creativity: The mediating role of intrinsic motivation and the creative process

engagement. J Creat Behav [Internet]. 2019 Mar 1;53(1):109–19. Available from: https://doi.org/10.1002/jocb.170

12. Dollinger SJ, Urban KK, James TA. Creativity and openness: Further validation of two creative product measures. Creat Res J [Internet]. 2004 Mar 1;16(1):35–47. Available from: https://doi.org/10.1207/s15326934crj1601_4

13. Bejar II. Rater cognition: Implications for validity. Educ Meas Issues Pract [Internet]. 2012 Sep 1;31(3):2–9. Available from: https://doi.org/10.1111/j.1745-3992.2012.00238.x

14. Ceh SM, Edelmann C, Hofer G, Benedek M. Assessing raters: What factors predict discernment in novice creativity raters? J Creat Behav. 2022;56(1):41–54.

15. Lloyd-Cox J, Pickering A, Bhattacharya J. Evaluating creativity: How idea context and rater personality affect considerations of novelty and usefulness. Creat Res J [Internet]. 2022 Oct 2;34(4):373–90. Available from: https://doi.org/10.1080/10400419.2022.2125721

16. Belfi AM, Vessel EA, Starr GG. Individual ratings of vividness predict aesthetic appeal in poetry. Psychol Aesthetics, Creat Arts. 2018;12(3):341–50.

17. Hitsuwari J, Nomura M. How individual states and traits predict aesthetic appreciation of haiku poetry. Empir Stud Arts. 2022;40(1):81–99.

18. Hitsuwari J, Nomura M. Ambiguity tolerance can improve through poetry appreciation and creation. J Creat Behav [Internet]. 2022 Dec 7;n/a(n/a). Available from: https://doi.org/10.1002/jocb.574

19. Amabile TM. Social psychology of creativity: A consensual assessment technique. J Pers Soc Psychol. 1982;43:997–1013.

20. Chamorro-Premuzic T, Burke C, Hsu A, Swami V. Personality predictors of artistic preferences as a function of the emotional valence and perceived complexity of paintings. Psychol Aesthetics, Creat Arts. 2010;4(4):196.

21. Tinio PPL. From artistic creation to aesthetic reception: The mirror model of art. Psychol Aesthetics, Creat Arts. 2013;7(3):265.

22. Cupchik GC, Vartanian O, Crawley A, Mikulis DJ. Viewing artworks: Contributions of cognitive control and perceptual facilitation to aesthetic experience. Brain Cogn [Internet]. 2009;70(1):84–91. Available from: https://www.sciencedirect.com/science/article/pii/S0278262609000098

23. Bao Y, Yang T, Lin X, Fang Y, Wang Y, Pöppel E, et al. Aesthetic preferences for Eastern and Western traditional visual art: identity matters. Front Psychol. 2016;7:1596.

24. Hagtvedt H, Patrick VM, Hagtvedt R. The perception and evaluation of visual art. Empir Stud Arts [Internet]. 2008 Jul 1;26(2):197–218. Available from: https://doi.org/10.2190/EM.26.2.d

25. Zioga I, Harrison PMC, Pearce MT, Bhattacharya J, Di Bernardi Luft C. From learning to creativity: Identifying the behavioural and neural correlates of learning to predict human judgements of musical creativity. Neuroimage [Internet]. 2020;206:116311. Available from: https://www.sciencedirect.com/science/article/pii/S1053811919309024

26. Silvia PJ, Fayn K, Nusbaum EC, Beaty RE. Openness to experience and awe in response to nature and music: Personality and profound aesthetic experiences. Psychol Aesthetics, Creat Arts. 2015;9:376–84.

27. Jackendoff R, Lerdahl F. The capacity for music: What is it, and what's special about it? Cognition [Internet]. 2006;100(1):33–72. Available from: https://www.sciencedirect.com/science/article/pii/S0010027705002210

28. Koelsch S. Brain correlates of music-evoked emotions. Nat Rev Neurosci [Internet]. 2014;15(3):170–80. Available from: https://doi.org/10.1038/nrn3666

29. Belfi AM. Emotional valence and vividness of imagery predict aesthetic appeal in music. . Psychomusicology Music Mind, Brain . 2019;29((2-3)):128–35.

30. Reybrouck M, Brattico E. Neuroplasticity beyond sounds: neural adaptations following long-term musical aesthetic experiences. Brain Sci. 2015;5(1):69–91.

31. Müller M, Höfel L, Brattico E, Jacobsen T. Aesthetic judgments of music in experts and laypersons — An ERP study. Int J Psychophysiol [Internet]. 2010;76(1):40–51. Available from: https://www.sciencedirect.com/science/article/pii/S0167876010000334

32. Hanich, J., Wagner, V., Shah, M., Jacobsen, T., & Menninghaus W. Why we like to watch sad films. The pleasure of being moved in aesthetic experiences. Psychol Aesthetics, Creat Arts. 2014;8(2):130–43.

33. Silvia PJ, Berg C. Finding movies interesting: How appraisals and expertise influence the aesthetic experience of film. Empir Stud Arts. 2011;29(1):73–88.

34. Plucker JA, Kaufman JC, Temple JS, Qian M. Do experts and novices evaluate movies the same way? Psychol Mark [Internet]. 2009 May 1;26(5):470–8. Available from: https://doi.org/10.1002/mar.20283

35. Jacobs AM, Kinder A. "The brain is the prisoner of thought": A machine-learning assisted quantitative narrative analysis of literary metaphors for use in neurocognitive

poetics. Metaphor Symb [Internet]. 2017 Jul 3;32(3):139–60. Available from: https://doi.org/10.1080/10926488.2017.1338015

36. Obermeier C, Menninghaus W, von Koppenfels M, Raettig T, Schmidt-Kassow M, Otterbein S, et al. Aesthetic and emotional effects of meter and rhyme in poetry [Internet]. Vol. 4, Frontiers in Psychology. 2013. Available from: https://www.frontiersin.org/articles/10.3389/fpsyg.2013.00010

37. Kraxenberger M, Menninghaus W. Affinity for poetry and aesthetic appreciation of joyful and sad poems [Internet]. Vol. 7, Frontiers in Psychology. 2017. Available from: https://www.frontiersin.org/articles/10.3389/fpsyg.2016.02051

38. Hitsuwari J, Nomura M. Ambiguity and beauty: Japanese-German cross-cultural comparisons on aesthetic evaluation of haiku poetry. Psychol Aesthetics, Creat Arts. 2022;No Pagination Specified-No Pagination Specified.

39. Scharinger M, Wagner V, Knoop CA, Menninghaus W. Melody in poems and songs: Fundamental statistical properties predict aesthetic evaluation. Psychol Aesthetics, Creat Arts. 2022;No Pagination Specified-No Pagination Specified.

40. Lau JH, Cohn T, Baldwin T, Brooke J, Hammond A. Deep-speare: A joint neural model of poetic language, meter and rhyme. 2018 Jul 10 [cited 2022 Nov 25]; Available from: http://arxiv.org/abs/1807.03491

41. Jacobs AM, Kinder A. What makes a metaphor literary? Answers from two computational studies. Metaphor Symb. 2018;33(2):85–100.

42. Steen GJ. From linguistic form to conceptual structure in five steps: Analyzing metaphor in poetry. Cogn Poet. 2009;197–226.

43. Rasse C, Onysko A, Citron FMM. Conceptual metaphors in poetry interpretation: A

psycholinguistic approach. Lang Cogn [Internet]. 2020/02/28. 2020;12(2):310–42. Available from: https://www.cambridge.org/core/article/conceptual-metaphors-in-poetry-interpretation-a-psycholinguistic-approach/E022AA06246E014936DB9329D6B99009

44. Jacobs AM. Quantifying the beauty of words: A neurocognitive poetics perspective [Internet]. Vol. 11, Frontiers in Human Neuroscience. 2017. Available from: https://www.frontiersin.org/articles/10.3389/fnhum.2017.00622

45. Aryani A, Kraxenberger M, Ullrich S, Jacobs AM, Conrad M. Measuring the basic affective tone of poems via phonological saliency and iconicity. Psychol Aesthetics, Creat Arts. 2016;10:191–204.

46. Lüdtke J, Meyer-Sickendieck B, Jacobs AM. Immersing in the stillness of an early morning: Testing the mood empathy hypothesis of poetry reception. Psychol Aesthetics, Creat Arts. 2014;8(3):363.

47. Kaufman JC, Baer J, Cole JC, Sexton∗ JD. A comparison of expert and nonexpert raters using the consensual assessment technique. Creat Res J [Internet]. 2008 May 7;20(2):171–8. Available from: https://doi.org/10.1080/10400410802059929

48. Kaufman JC, Niu W, Sexton JD, Cole JC. In the eye of the beholder: differences across ethnicity and gender in evaluating creative work. J Appl Soc Psychol [Internet]. 2010 Feb 1;40(2):496–511. Available from: https://doi.org/10.1111/j.1559-1816.2009.00584.x

49. Russell JA. A circumplex model of affect. J Pers Soc Psychol. 1980;39(6):1161.

50. Wassiliwizky E, Koelsch S, Wagner V, Jacobsen T, Menninghaus W. The emotional power of poetry: neural circuitry, psychophysiology and compositional principles. Soc

Cogn Affect Neurosci [Internet]. 2017 Aug 1;12(8):1229–40. Available from: https://doi.org/10.1093/scan/nsx069

51. Mastria S, Agnoli S, Corazza GE. How does emotion influence the creativity evaluation of exogenous alternative ideas? PLoS One [Internet]. 2019 Jul 5;14(7):e0219298. Available from: https://doi.org/10.1371/journal.pone.0219298

52. Johnson-Laird PN, Oatley K. How poetry evokes emotions. Acta Psychol (Amst) [Internet]. 2022;224:103506. Available from: https://www.sciencedirect.com/science/article/pii/S000169182200021X

53. Gabrielsson A. Emotion perceived and emotion felt: Same or different? Music Sci [Internet]. 2001 Sep 1;5(1_suppl):123–47. Available from: https://doi.org/10.1177/10298649020050S105

54. Marin MM, Bhattacharya J. Music induced emotions: some current issues and cross-modal comparisons. In: Hermida J, Ferrero M, editors. Music education. Hauppauge, NY: Nova Science Publishers; 2010. p. 1–38.

55. Meyer, W. U., Reisenzein, R., & Schützwohl A. Toward a process analysis of emotions: The case of surprise. Motiv Emot. 1997;21(3):251–74.

56. Meyer W-U, Niepel M, Rudolph U, Schützwohl A. An experimental analysis of surprise. Cogn Emot. 1991;5(4):295–311.

57. Noordewier MK, Breugelmans SM. On the valence of surprise. Cogn Emot [Internet]. 2013 Nov 1;27(7):1326–34. Available from: https://doi.org/10.1080/02699931.2013.777660

58. Rumelhart DE. Schemata and the cognitive system. 1984;

59. Simonton DK. Taking the U.S. patent office criteria seriously: A quantitative three-criterion creativity definition and its implications. Creat Res J [Internet]. 2012 Apr 1;24(2–3):97–106. Available from: https://doi.org/10.1080/10400419.2012.676974

60. Acar S, Burnett C, Cabra JF. Ingredients of creativity: Originality and more. Creat Res J [Internet]. 2017 Apr 3;29(2):133–44. Available from: https://doi.org/10.1080/10400419.2017.1302776

61. Pietras K, Ganczarek J. Aesthetic reactions to violations in contemporary art: The role of expertise and individual differences. Creat Res J. 2022;34(3):324–38.

62. Oleynick VC, DeYoung CG, Hyde E, Kaufman SB, Beaty RE, Silvia PJ. Openness/intellect: The core of the creative personality. In: The Cambridge handbook of creativity and personality research. New York, NY, US: Cambridge University Press; 2017. p. 9–27. (Cambridge handbooks in psychology.).

63. DeYoung CG, Grazioplene RG, Peterson JB. From madness to genius: The Openness/Intellect trait domain as a paradoxical simplex. J Res Pers [Internet]. 2012;46(1):63–78. Available from: https://www.sciencedirect.com/science/article/pii/S0092656611001644

64. Chamorro-Premuzic T, Reimers S, Hsu A, Ahmetoglu G. Who art thou? Personality predictors of artistic preferences in a large UK sample: The importance of openness. Br J Psychol [Internet]. 2009 Aug 1;100(3):501–16. Available from: https://doi.org/10.1348/000712608X366867

65. McCrae RR. Social consequences of experiential openness. Psychol Bull. 1996;120(3):323–337.

66. Sánchez-Bernardos ML, Avia MD. Personality correlates of fantasy proneness among

adolescents. Pers Individ Dif [Internet]. 2004;37(5):1069–79. Available from: https://www.sciencedirect.com/science/article/pii/S0191886903004434

67. Fayn K, MacCann C, Tiliopoulos N, Silvia PJ. Aesthetic emotions and aesthetic people: Openness predicts sensitivity to novelty in the experiences of interest and pleasure [Internet]. Vol. 6, Frontiers in Psychology. 2015. Available from: https://www.frontiersin.org/articles/10.3389/fpsyg.2015.01877

68. Silvia PJ, Christensen AP. Looking up at the curious personality: individual differences in curiosity and openness to experience. Curr Opin Behav Sci [Internet]. 2020;35:1–6. Available from: https://www.sciencedirect.com/science/article/pii/S2352154620300863

69. DeYoung CG, Quilty LC, Peterson JB. Between facets and domains: 10 aspects of the Big Five. J Pers Soc Psychol. 2007;93(5):880.

70. Mussel P. Intellect: a theoretical framework for personality traits related to intellectual achievements. J Pers Soc Psychol. 2013;104(5):885.

71. Costa PT, McCrae RR. Four ways five factors are basic. Pers Individ Dif [Internet]. 1992;13(6):653–65. Available from: https://www.sciencedirect.com/science/article/pii/019188699290236I

72. Siegel DJ. The developing mind: How relationships and the brain interact to shape who we are (2. utg.). New York Guilford Publ. 2012;

73. DeYoung CG, Quilty LC, Peterson JB, Gray JR. Openness to Experience, Intellect, and Cognitive Ability. J Pers Assess [Internet]. 2014 Jan 2;96(1):46–52. Available from: https://doi.org/10.1080/00223891.2013.806327

74. Berlyne DE. Conflict and the orientation reaction. J Exp Psychol. 1961;62:476–83.

75. Keltner D, Haidt J. Approaching awe, a moral, spiritual, and aesthetic emotion. Cogn Emot [Internet]. 2003 Jan 1;17(2):297–314. Available from: https://doi.org/10.1080/02699930302297

76. Shiota MN, Keltner D, Mossman A. The nature of awe: Elicitors, appraisals, and effects on self-concept. Cogn Emot [Internet]. 2007 Aug 1;21(5):944–63. Available from: https://doi.org/10.1080/02699930600923668

77. Williams PG, Johnson KT, Bride DL, Baucom BRW, Crowell SE. Individual differences in aesthetic engagement and proneness to aesthetic chill: Associations with awe. Psychol Aesthetics, Creat Arts. 2022;No Pagination Specified-No Pagination Specified.

78. McCrae RR. Aesthetic chills as a universal marker of openness to experience. Motiv Emot [Internet]. 2007;31(1):5–11. Available from: https://doi.org/10.1007/s11031-007-9053-1

79. Costa PT, McCrae RR. The revised neo personality inventory (neo-pi-r). SAGE Handb Personal theory Assess. 2008;2(2):179–98.

80. Frijda NH. The emotions. Cambridge University Press; 1986.

81. Berlyne DE. Curiosity and exploration. Science (80-) [Internet]. 1966 Jul 1;153(3731):25–33. Available from: https://doi.org/10.1126/science.153.3731.25

82. Keltner D, Shiota MN. New displays and new emotions: a commentary on Rozin and Cohen (2003). 2003;

83. Kashdan TB, Silvia PJ. Curiosity and interest: The benefits of thriving on novelty and challenge. Oxford Handb Posit Psychol. 2009;2:367–74.

84. Berlyne DE. A theory of human curiosity. Br J Psychol. 1954;45(3):180.

85. Loewenstein G. The psychology of curiosity: A review and reinterpretation. Psychol Bull. 1994;116(1):75.

86. Litman JA, Spielberger CD. Measuring epistemic curiosity and its diversive and specific components. J Pers Assess [Internet]. 2003 Feb 1;80(1):75–86. Available from: https://doi.org/10.1207/S15327752JPA8001_16

87. Litman J, Hutchins T, Russon R. Epistemic curiosity, feeling-of-knowing, and exploratory behaviour. Cogn Emot [Internet]. 2005 Jun 1;19(4):559–82. Available from: https://doi.org/10.1080/02699930441000427

88. McCrae, R. R., & Costa Jr PT. Conceptions and correlates of openness to experience. Handbook of personality psychology. Academic Press; 1997. 825–847 p.

89. Kashdan TB, Rose P, Fincham FD. Curiosity and exploration: facilitating positive subjective experiences and personal growth opportunities. J Pers Assess [Internet]. 2004 Jun 1;82(3):291–305. Available from: https://doi.org/10.1207/s15327752jpa8203_05

90. Kashdan TB, Gallagher MW, Silvia PJ, Winterstein BP, Breen WE, Terhar D, et al. Curiosity and Exploration Inventory--II. J Res Pers. 2009;43(6):987–998.

91. Kashdan TB, Stiksma MC, Disabato DJ, McKnight PE, Bekier J, Kaji J, et al. The five-dimensional curiosity scale: Capturing the bandwidth of curiosity and identifying four unique subgroups of curious people. J Res Pers [Internet]. 2018;73:130–49. Available from: https://www.sciencedirect.com/science/article/pii/S0092656617301149

92. Papp-Zipernovszky O, Mangen A, Jacobs A, Lüdtke J. Shakespeare sonnet reading:

An empirical study of emotional responses. Lang Lit [Internet]. 2021 Dec 7;31(3):296–324. Available from: https://doi.org/10.1177/09639470211054647

93. McCarthy PM, Jarvis S. vocd: A theoretical and empirical evaluation. Lang Test. 2007;24(4):459–88.

94. Chotlos JW. IV. A statistical and comparative analysis of individual written language samples. Psychol Monogr. 1944;56(2):75.

95. Johnson DR, Kaufman JC, Baker BS, Patterson JD, Barbot B, Green AE, et al. Divergent semantic integration (DSI): Extracting creativity from narratives with distributional semantic modeling. Behav Res Methods [Internet]. 2022; Available from: https://doi.org/10.3758/s13428-022-01986-2

96. Faul F, Erdfelder E, Lang A-G, Buchner A. G* Power 3: A flexible statistical power analysis program for the social, behavioral, and biomedical sciences. Behav Res Methods. 2007;39(2):175–91.

97. Gosling SD, Rentfrow PJ, Swann WB. A very brief measure of the Big-Five personality domains. J Res Pers [Internet]. 2003;37(6):504–28. Available from: https://www.sciencedirect.com/science/article/pii/S0092656603000461

98. Bates, D., Mächler, M., Bolker, B., & Walker S. Fitting linear mixed-effects models using lme4. J Stat Softw. 2015;67:1–48.

99. Enders, C. K., & Tofighi D. Centering predictor variables in cross-sectional multilevel models: a new look at an old issue. Psychol Methods. 2007;12(2):121.

100. Cohen, J. & Cohen P. Applied multiple regression/correlation analysis for the behavioral sciences,. 2nd ed. Hillsdale, NJ: Erlbaum.; 1983.

101. Nakagawa S, Schielzeth H. A general and simple method for obtaining R2 from generalized linear mixed- effects models. Methods Ecol Evol. 2013;4(2):133–42.

102. Leder H, Belke B, Oeberst A, Augustin D. A model of aesthetic appreciation and aesthetic judgments. Br J Psychol [Internet]. 2004 Nov 1;95(4):489–508. Available from: https://doi.org/10.1348/0007126042369811

103. Johnston V. The origin and function of pleasure. Cogn Emot. 2003;17(2):167–79.

104. Russell JA. Core affect and the psychological construction of emotion. Psychol Rev. 2003;110(1):145.

105. Barrett LF, Mesquita B, Ochsner KN, Gross JJ. The experience of emotion. Annu Rev Psychol. 2007;58:373–403.

106. Vessel EA, Rubin N. Beauty and the beholder: Highly individual taste for abstract, but not real-world images. J Vis. 2010;10(2):18.

107. Furnham A, Walker J. The influence of personality traits, previous experience of art, and demographic variables on artistic preference. Pers Individ Dif. 2001;31(6):997–1017.

108. Haidt J, Keltner D, Peterson C, Seligman ME. Appreciation of beauty and excellence. Character strengths and virtues. 2004;537–51.

109. Peterson C, Seligman MEP. Character strengths and virtues: A handbook and classification. Vol. 1. Oxford University Press; 2004.

110. Diessner R, Solom RC, Frost NK, Parsons L, Davidson J. Engagement with beauty: Appreciating natural, artistic, and moral beauty. J Psychol. 2008;142(3):303.

111. Shiota MN, Keltner D, John OP. Positive emotion dispositions differentially associated

with Big Five personality and attachment style. J Posit Psychol. 2006;1(2):61–71.

112. Güsewell A, Ruch W. Are only emotional strengths emotional? Character strengths and disposition to positive emotions. Appl Psychol Heal Well- Being. 2012;4(2):218–39.

113. Gross ME, Zedelius CM, Schooler JW. Cultivating an understanding of curiosity as a seed for creativity. Curr Opin Behav Sci. 2020;35:77–82.

114. Kenett YN, Humphries S, Chatterjee A. A Thirst for Knowledge: Grounding Curiosity, Creativity, and Aesthetics in Memory and Reward Neural Systems. Creat Res J. 2023;1–15.

115. Silvia PJ. Appraisal components and emotion traits: Examining the appraisal basis of trait curiosity. Cogn Emot [Internet]. 2008 Jan 1;22(1):94–113. Available from: https://doi.org/10.1080/02699930701298481

116. Kashdan TB, Gallagher MW, Silvia PJ, Winterstein BP, Breen WE, Terhar D, et al. The curiosity and exploration inventory-II: Development, factor structure, and psychometrics. J Res Pers [Internet]. 2009;43(6):987–98. Available from: https://www.sciencedirect.com/science/article/pii/S0092656609001275

117. Mussel P. Epistemic curiosity and related constructs: Lacking evidence of discriminant validity. Pers Individ Dif. 2010;49(5):506–10.

118. Silvia PJ. Exploring the psychology of interest. oxford university Press; 2006.

119. Christensen AP, Kenett YN, Cotter KN, Beaty RE, Silvia PJ. Remotely close associations: Openness to experience and semantic memory structure. Eur J Pers. 2018;32(4):480–92.

120. Anderson CL, Dixson DD, Monroy M, Keltner D. Are awe-prone people more curious? The relationship between dispositional awe, curiosity, and academic outcomes. J Pers [Internet]. 2020 Aug 1;88(4):762–79. Available from: https://doi.org/10.1111/jopy.12524

121. Izard CE. Human Emotions. New York: Plenum; 1977.

122. Schubert E. Emotion felt by the listener and expressed by the music: literature review and theoretical perspectives. Front Psychol. 2013;4:837.

123. Fleeson W. Toward a structure-and process-integrated view of personality: Traits as density distributions of states. J Pers Soc Psychol. 2001;80(6):1011.

124. Malvern DD, Richards BJ. A new measure of lexical diversity. Br Stud Appl Linguist. 1997;12:58–71.

125. McCarthy PM, Jarvis S. MTLD, vocd-D, and HD-D: A validation study of sophisticated approaches to lexical diversity assessment. Behav Res Methods [Internet]. 2010;42(2):381–92. Available from: https://doi.org/10.3758/BRM.42.2.381

126. Hassenzahl M. The interplay of beauty, goodness, and usability in interactive products. Human–Computer Interact. 2004;19(4):319–49.

127. Chamberlain R, Mullin C, Scheerlinck B, Wagemans J. Putting the art in artificial: Aesthetic responses to computer-generated art. Psychol Aesthetics, Creat Arts. 2018;12(2):177.

128. Frame J, Mehl K, Head K, Belfi A. The influence of sensory modality on aesthetic judgments of poetry. 2023;

129. Mehl K, Gugliano M, Belfi AM. The role of imagery and emotion in the aesthetic

appeal of music, poetry, and paintings. Psychol Aesthetics, Creat Arts. 2023;No Pagination Specified-No Pagination Specified.

130. Zhang JD, Schubert E. A Single Item Measure for Identifying Musician and Nonmusician Categories Based on Measures of Musical Sophistication. Music Percept [Internet]. 2019 Jun 1;36(5):457–67. Available from: https://doi.org/10.1525/mp.2019.36.5.457

131. Bergkvist L, Rossiter JR. The predictive validity of multiple-item versus single-item measures of the same constructs. J Mark Res. 2007;44(2):175–84.

132. Rhodes M. An analysis of creativity. Phi delta kappan. 1961;42(7):305–10.

133. Pinho AL, de Manzano Ö, Fransson P, Eriksson H, Ullén F. Connecting to create: expertise in musical improvisation is associated with increased functional connectivity between premotor and prefrontal areas. J Neurosci. 2014;34(18):6156–63.

134. Toivainen T, Madrid- Valero JJ, Chapman R, McMillan A, Oliver BR, Kovas Y. Creative expressiveness in childhood writing predicts educational achievement beyond motivation and intelligence: A longitudinal, genetically informed study. Br J Educ Psychol. 2021;91(4):1395–413.

135. Guenther A, Eisenbart B, Dong A. Creativity and successful product concept selection for innovation. Int J Des Creat Innov. 2021;9(1):3–19.

Formatted: Indent: Left: 0", Hanging: 0.44", No widow/orphan control, Don't adjust space between Latin and Asian text, Don't adjust space between Asian text and numbers

Our Responses to the Editor and the Reviewer(s)

Thank you very much for giving us an opportunity to revise the manuscript. Below are our responses to each comment of the Academic editor and the Reviewer. The Editor's and Reviewers' comments are shown in red, our responses are in regular font, and the inclusions are in **bold green.**

Our responses to the Editor's comments

1. Lines 28-29: "...of a broad range of English language poems ... rated each poem..." You have not stated yet how many poems were used. Instead of 'broad range', state the number

Our response: Thank you for your suggestion. The number of poems has now been mentioned in the manuscript, as follows: "This study investigated how personality traits, specifically openness, intellect, awe-proneness, and epistemic curiosity, shape the implicit associations between subjective experiences and the judgment of creativity of **thirty-six** English language poems."

2. Line 106: "meter (36, 40), , metaphors (35), (41,42,43), and" either there is bad formatting, or something is missing on this line.

Our response: Thank you. Formatting has been revised.

3. Line 117: "we considered both dimensions of emotion – valence and arousal." Both 'valence' and 'arousal' are technical term in psychology and related fields. Please be kind enough to explain those terms to the general audience (a footnote may suffice). Moreover, since 'felt valence' was found to be important in your work, explain to readers what it is.

Our response: Thank you for the suggestion. We explained the terms in the revised manuscript as suggested. The lines read as follows:

"According to the two-dimensional circumplex model of emotion, emotional states are represented along two orthogonal dimensions: valence (pleasure-displeasure: horizontal axis) and arousal (degree of arousal: vertical axis) (Russell, 1980). Poetry evokes strong emotions (Wassiliwizky et al., 2017) and emotional states can influence creativity evaluation (Mastria et al., 2019)…. Of note, perceived and felt emotions may be distinct (Gabrielsson, 2001; Marin & Bhattacharya, 2010). Perceived emotions are the emotions evoked by the stimuli whereas felt emotions are the emotions felt by the perceivers. In our study, we focused on the felt emotions, i.e., the emotions felt by the reader while reading the poem, rather than the perceived emotion, i.e., the emotion recognized in the poem. Therefore, felt valence reveals the extent to which the readers felt positive or negative emotions while reading the poems, whereas felt arousal here reveals how intense it was felt by the readers."

4. Lines 122-125: “Surprise is considered as a stronger predictor of creativity than value after...” The description of 'Surprise' dimension is very dry and technical (unlike the other descriptions), and it does not explain what is 'Surprise' in this context. Also what is 'value' in the above sentence? Did you mean valence?

Our response: Thank you very much for the comment. We have now expanded our explanation to clarify the specific meaning of “surprise’ within the context of our study. The section reads as: **“Surprise is usually a short-lived emotion elicited by events that deviate from an established schema (Meyer et al., 1997; Meyer et al., 1991; Noordewier & Breugelmans, 2013). Of note, a schema, in this sense, refers to a component of the organism’s knowledge structure, activated by a specific stimulus (Rumelhart, 1984). Surprise is recognized as a key predictor of creativity (Simonton, 2012; Acar et al., 2017) and is also a robust predictor of aesthetic judgment of artwork (Pietras & Ganczarek, 2022). In the context of our study, the term “surprise” specifically denotes the degree to which there is a sudden and unexpected change in the context or theme of the poem.”**

Response to your next question on “value”: Despite the lack of an agreed-upon definition, a general consensus over a standard definition of creativity (Runco & Jaeger, 2012; see also Stein, 1953; Barron, 1955) involves two factors: The first factor consists of characteristics such as novelty, originality, infrequency, or unusualness; and the second factor is related to usefulness, value, utility, effectiveness, adaptability, or appropriateness (Acar et al., 2017). **However, we excluded the line containing “value” to avoid confusion of readers as we did not explore any potential consequences of this term later in the article.**

Line 127: “It is a broad range of traits...” What is “It”? Why do you begin with “It”? Please formulate the description clearly.

Our response: The line has been amended as: **Openness to experience** is a broad range of traits, from intellectual abilities to aesthetic and artistic interests (Oleynick et al., 2017;DeYoung et al., 2012; Chamorro-Premuzic et al., 2009) and is most robustly associated with measures of creativity (McCrae, 1996).

Lines 130-132: “Openness/intellect … can be differentiated into two major aspects (64): openness and intellect.” Really? Isn't it a bit tautological? Please clarify – is it one predictor or two? If those are two predictors, why are they bundled together?

Our response: Actually, Costa Jr & McCrae, (1992) first introduced Openness to Experience as the fifth factor of personality traits. Later, DeYoung et al created the Openness/Intellect (O/I) model, which proposed that Openness to Experience has two major facets– the two “related but separable trait dimensions” (DeYoung et al., 2007, p.883): openness and intellect. Based on earlier work on the neurocognitive correlates of Openness to Experience (DeYoung et al., 2005), DeYoung et al., 2009 hypothesized that brain regions associated with working memory would predict the intellect

facets but not the openness facets. Showing distinct effects of openness and intellect would provide evidence for the validity of both constructs (see Nusbaum & Silvia, 2011).

Hence, in our study, we are interested to explore the distinct influence of openness and intellect on the evaluation of creativity of poems.

5. Lines 152-153: "Earlier research has shown that openness influences attitudes toward targets that violate established schemas (68)." This is absolutely unclear, needs some elaboration. What are 'targets'? Targets of what? Which established schemas? OF behavior? Of Social norms? Of poems? Of poetic structure? What is meant by 'violate' here?

Our response: We appreciate your comment. We revised the lines as: **"As surprise is often experienced when individuals encounter unexpected or schema-discrepant events (Meyer et al., 1997; Meyer et al., 1991), we assumed that the assessment of expectancy violations in the judgment of poetic creativity necessitates a substantial degree of cognitive engagement. Consequently, we predicted that both Openness and Intellect would moderate the relationship between surprise and creativity. "**

6. Line 168: "aesthetic chill" Please explain what is aesthetic chill.

Our response: We explained the term. The line now reads: **"Aesthetic chills are transient emotional responses (McCrae, 2007) to aesthetical stimuli. They can be described as experiencing a chill or wave of excitement when engaging with poetry or looking at a work of art (Costa & McCrae, 2008)."**

7. Line 196: Please explain what is "Prolific®"

Our response: Explained as suggested: **"The experiment was created using Qualtrics®, and the link was disseminated through Prolific®, a platform for participant recruitment."**

8. Section "Materials and methods" This section starts with 'Participants' section and it's quite awkward. The 'Participants' section should be moved after the 'Materials' section.

Our response: Thank you. "Participants" section is now shifted after the "Materials" section, as suggested.

9. Lines 194-196: The 'Participants' section begins with a description of minimum sample size. This section needs to be clarified. What led you to focus on "80% power for a medium effect (^2 = 0.15) with squared multiple correlation ^2 = 0.13." It seems rather arbitrary.

Our response: The section has been clarified as suggested. The section now reads as:

"By using the G*Power software (v. 3.1.9.4), (Faul et al., 2007) we found that a minimum sample size of 98 was required to detect a medium effect size (f^2 = 0.15) in a multiple linear regression, assuming a significance level of 0.05 and a statistical power of 80%. The criteria we used are widely-used conventional figures when estimating sample sizes. Additionally, we used the 'samplesize_mixed' function in R, which estimates the required sample size for linear mixed models (two-level-designs), based on power-calculation for standard design and adjusted for design effect for 2-level-designs (https://strengejacke.github.io/sjstats/). For our multilevel model, which considered 96 cluster groups and a small to medium effect size (Cohen's d = 0.3), with 36 observations per cluster group (level-2-unit), we determined that a total of 965 observations would be necessary. This translates to a minimum of 27 participants (965/36)."

10. Also, you did not specify where the participants came from, in terms of education, profession, English language proficiency (native/non-native) and age groups. Would it be possible to have some indications about those background variables?

Our response: As per the self-reported demographic questionnaires, all 96 participants (32 males, 63 females, 1 preferred not to say; mean age = 31.94 years, SD = 13.09) were fluent in English and came from a variety of educational backgrounds, with each holding at least a bachelor's degree in any discipline. Additionally, our sample represented a diverse range of professions and ethnicities.

We included this information in the "Participants" section.

11. Line 196: "healthy participants" What does 'healthy' mean here? Maybe specify in a footnote. What criteria were used? Is this aspect really important?

Our response: Thank you for your comment. Considering that the "health" aspect was not a significant focus of our study, we omitted the term "healthy."

12. Line 202: "We described the main experimental procedures for this research to the participants" It is not clear what was explained. Maybe add an appendix? How did you explain 'valence' and 'arousal' to the participants?

Our response: The instructions to the participants are now included in the main manuscript. The full paragraph now reads:

"Participants were briefed about the experimental procedure, which involved the assessment of a set of English poems on a 7-point Likert rating scale (1=extremely low; 7=extremely high) across various constructs, including clarity, aesthetic appeal, felt valence, arousal, surprise, and overall creativity. Additionally, participants were instructed to complete demographic and personality-related questions. We assured participants of the full confidentiality of their data, in compliance with the General Data Protection Regulation, and clarified that any published results would be non-identifiable. All participants provided informed consent (online) before data collection. Participants received a cash incentive of £7.50 per hour. The data collection period spanned from 27 January 2022 to 23 June 2022, and the data were accessed for research purposes only after this period."

How did you explain 'valence' and 'arousal' to the participants?

For felt valence, participants were asked: “How positive (higher Scores) or negative (lower Scores) did you feel when you read the poem?” For arousal, they were asked: “How stimulating (higher Scores) or relaxing (lower Scores) did you feel when you read the poem?”

13. Section 'Materials': The procedure of poem selection is not described well. Selection sources (lines 224-227) should be described before the lexical metrics of selected poems (lines 213-223).

Our response: As suggested, the sources for poem selection have been described in the “Material” section:

“Initially, we selected 108 poems from various online resources, including Poetry.org (http://www.poetry.org/), the Poetry Foundation (https://www.poetryfoundation.org/), and the Academy of American Poets (https://poets.org/). These poems were subsequently rated for "surprise" on a scale of 1 to 7 by M.D, one of the co-authors who is a Professor of Creative Writing with domain-specific expertise and an award-winning poet. Following this evaluation, we shortlisted 36 as the experimental stimuli: 18 with low surprise ratings (4 or lower) and 18 with high surprise ratings (6 or above). The chosen poems varied in structure, contents, lines, and word count (mean number of lines = 11, SD = 3.2; mean word count = 71.3, SD = 29). To represent a broad spectrum of English poems, we consciously chose not to limit our selection to a particular genre or form, like haiku or sonnets as done in previous studies (Belfi et al., 2018**;** Hitsuwari & Nomura, 2022**;** (Papp-Zipernovszky et al., 2021)**.**

13. How did you converge on the genres and themes that are reported in Table 1? Who provided the labels of genres and themes?

M.D, a co-author of our study who is a Professor of Creative Writing with domain-specific expertise and an award-winning poet, validated the genre and theme-specific information about the stimuli.

14. Lines 218-221. Concerning “lexical diversity (LD)”. Explain to readers what is LD, and why it is relevant for this study. There are many metrics for LD. Please describe which one you used. The legend of table 1 says “(LD = number of unique words/ total characters).” That should be explained in the text. Also, that is not a common metric of LD. Where did you adopt it from? “The average (SD) LD score for our stimuli set was 14.30 (1.7).” Switch “(SD) LD” to “LD (DS)”. Also explain to readers: is 14.30 high or low? What is the scale and what does the value mean?

Our response: Thanks for this valuable comment. While lexical diversity and semantic diversity may not be directly pertinent to our study, we decided to include them to capture the variedness and richness of vocabulary and semantics in our chosen stimuli. Of note, we rectified the lexical diversity scores (computed in R). Table1, originally presenting stimulus details, has been modified to incorporate updated Lexical Diversity and is now designated as Table S1. We intend to include it in the Supplementary section.The paragraph describing the diversity metrics has been modified as follows:

“Lexical diversity (LD) of a text refers to its lexical richness, indicating range and variety of vocabulary deployed in a text (McCarthy & Jarvis, 2007). For computing the LD of the poems, we

used the type-token ratio (TTR) method, which calculates the ratio of unique words (types) to the total word count (tokens) (Chotlos, 1944). It ranges from 0 to 1: a higher TTR indicates a greater lexical diversity. The average lexical diversity across the poems is 0.77 with a standard deviation of 0.09, suggesting that, on average, about 77% of the words used in the poems are unique or different. Semantic diversity, on the other hand, refers to the range of contexts (i.e., semantic richness) in which words are used (Johnson et al., 2022). To calculate the semantic diversity, we used divergent semantic integration (DSI) (http://semdis.wlu.psu.edu/), representing the mean semantic distance between all word pairs in a text; DSI varies from 0 to 1, where a higher score indicates that a broader collection of divergent ideas. The average DSI of our stimuli is 0.80 with a standard deviation of 0.03, indicating a high degree of semantic variety (see Table S1 in the Supplementary section for details).”

In the “Limitations” section, we added the limitations of TTR method for short texts as follows: **“it is important to acknowledge that given the small word count of some of our poems and the implied limited vocabulary, the Type-Token Ratio (TTR) method might not yield reliable results due to constrained variability in word usage within small texts (Malvern & Richards, 1997; McCarthy & Jarvis, 2010).”**

The table (**Table S1 in the Supplementary section**) containing the details of the stimuli will be submitted in the Supplementary section and OSF (https://osf.io/9mw7r/?view_only=07137f4871d146c790501f22bc7743d5).

TableS1. The details of the stimuli

Poem Title	Poet	Theme	Genre	Word Count	No. of lines	Lexical Diversity(TTR)	DSI_mean
Funeral Blues	W. H. Auden	Grief	Modernist	136	16	0.742647059	0.80700305
At the Same Time	W.S. Merwin	Transience of existence	Contemporary	39	10	0.794871795	0.81102673
The Supreme Moment	Charles Simic	Powerlessness of humans	Contemporary	63	16	0.888888889	0.83547634
Peace	Langston Hughes	Futility of war	War poetry	26	8	0.884615385	0.77777005
A Peck of Gold	Robert Frost	Illusion	Modernist	86	12	0.569767442	0.76179961
The Freedom of the Moon	Robert Frost	Awe inspiring nature	Contemporary	91	12	0.769230769	0.80886736
Stopping by Woods on a Snowy Ev	Robert Frost	Contemplation	Modernist	108	16	0.694444444	0.78485471
When You Come	Maya Angelou	Nostalgia	Contemporary	41	10	0.829268293	0.82847741
Sonnet 116	William Shakespear	Eternity of love	Romantic	109	14	0.752293578	0.80245092
Apparently with no Surprise	Emily Dickinson	Transience of life	Nature poetry	36	8	0.916666667	0.82995713
Will there really be a "Morning"?	Emily Dickinson	Wonder and curiosity	Lyric	76	12	0.710526316	0.77878051
Unable are the Loved to die	Emily Dickinson	Immortality of love	Lyric	26	6	0.8	0.79995234
How happy is the little Stone	Emily Dickinson	Independence and contentment	Nature	46	10	0.869565217	0.83859107
Ah! Sun-flower	William Blake	Individualism	Romantic	50	8	0.84	0.81487795
The Smile	William Blake	Love and deceit	Romantic	106	16	0.528301887	0.74556787
Love after Love	Derek Walcott	Self-love, self-discovery	Contemporary	98	15	0.673469388	0.78304363
The Last Laugh	Wilfred Owen	Brutality of war	War poem	90	15	0.833333333	0.84464281
Clown in the Moon	Dylan Thomas	Melancholy	Lyric	48	8	0.770833333	0.77714294
Remember	Christina Rossetti	Love, death, remembrance	Lyric	111	14	0.630630631	0.73742609
When You Are Old	W. B. Yeats	True love	Romantic	100	12	0.7	0.78672981
Aedh wishes for the Cloths of Hea	W. B. Yeats	Love and dreams	Romantic	60	9	0.7	0.78105161
Memory	W.B. Yeats	Transience of youth and beauty	Lyric	33	6	0.818181818	0.79140706
The Arrow and the Song	Henry Wadsworth L	Power of words and friendship	Lyric	88	12	0.613636364	0.75714821
There Will Come Soft Rains	Sara Teasdale	War	Lyric	91	12	0.747252747	0.81156949
Alone	Sara Teasdale	Solitude	Romantic	80	12	0.7	0.77622178
Ozymandias	P. B. Shelley	Transience of power	Romantic	111	14	0.787610619	0.8292663
Good-Night	P. B. Shelley	Longing for togetherness	Romantic	75	12	0.805194805	0.77736462
A Lament	P. B. Shelley	Nostalgia, sadness	Lyric	65	10	0.815384615	0.80580131
Alas! This Is Not What I Thought L	P. B. Shelley	Challenges of life	Lyric	75	9	0.815789474	0.79610722
Fragment: A Wanderer	P. B. Shelley	Imagination	Romantic	25	4	0.88	0.83606831
Fragment: Apostrophe to Silence	P. B. Shelley	Existential longing	Romantic	69	9	0.819444444	0.8170317
MEDITATION XVII	John Donne	Interconnectedness of humanity	Romantic	81	13	0.728395062	0.77863012
Nothing Gold Can Stay	Robert Frost	Impermanence of beauty	Nature	40	8	0.85	0.81230023
My Heart Leaps Up	William Wordswort	Ecstasy with nature’s beauty	Nature	61	9	0.704918033	0.75068272
I taste a liquor never brewed –	Emily Dickinson	Joy of Nature	Lyric	81	16	0.80952381	0.83325713
If You Should Go	Countee Cullen	Transience of love and life	Romantic	44	8	0.818181818	0.76151851

15. Lines 222-224: Explain to readers what is DSI, and why it is relevant for this study. What is the scale and where on the scale is your value (0.8) located?

Our response: We explained DSI in the "Materials" section as follows:

"Semantic diversity, on the other hand, refers to the range of contexts (i.e., semantic richness) in which words are used (Johnson et al., 2022). To calculate the semantic diversity, we used divergent semantic integration (DSI) (http://semdis.wlu.psu.edu/), representing the mean semantic distance between all word pairs in a text; DSI varies from 0 to 1, where a higher score indicates that a broader collection of divergent ideas. The average DSI of our stimuli is 0.80 with a standard deviation of 0.03, indicating a high degree of semantic variety (see Table S1 in the Supplementary section for details)."

16. Lines 241-242: "Participants read a poem for 30 seconds" Explain why such a time limit was set? Was the poem still visible during creativity rating?

Our response: Given the concise nature of the poems (average number of lines = 11), we opted for a minimum reading time of 30 seconds. Participants were given a minimum of 30 seconds to read each poem. Following this period, they were allowed to proceed to the rating task. They were asked to rate the poems on six dimensions in the following order: clarity, aesthetic appeal, felt valence, felt arousal, surprise, and creativity, using a 7-point Likert scale (1: "Extremely Low" and 7: "Extremely High"). There was no time limit imposed for rating the poems. Of note, the poems remained visible during the rating process. This information has been included in the "Procedure" section.

17. Regarding the questions/measures on four personality traits: as those were shown to be correlated with creativity (Table 3). Please briefly explain the scales and value ranges for the personality traits measures. Moreover, please describe the distributions of values for all personality measures in your study. For example, As described in Table 2, it seems the four personality traits are also on 7-point scale. Can you establish that you had sufficient variability on the personality scores? It is not clear whether you participants represent sufficient difference on an Openness scale (and other scales). The SD for Openness is just 0.74 – are all participants approximately equally 'open'?. Do you have sufficient distribution for levels of intellect? Sufficient in terms of representing the general public?

Our response:

Of note, there was a typo in our curiosity rating data. We have rectified this in the revised manuscript. Therefore, the curiosity data in the Tables had undergone some minor changes, but without any significant impact on the findings and their implications.

scales for the personality traits measures: All scales are 1-7

value ranges:

Openness: min = 3.0; max = 6.4; mean = 5.02; SD = 0.74

Intellect: min = 2.7; max = 6.4; mean = 4.7; SD = 0.9

Awe-proneness: min = 1.83; max = 7; mean = 5.11; SD = 1.14

Curiosity: min = 3.5; max = 7; mean = 5.58; SD = 0.86

Variability in openness scores: 0.55 (moderate, suggesting a reasonable spread of scores among participants.)

Variability in intellect scores: 0.81 (high, indicating a broader range of scores among participants.)

Variability in awe-proneness scores: 1.31 (high, suggesting a diverse range of responses among participants.)

Variability in curiosity scores: 0.76 (moderate, indicating a reasonable distribution of scores.)

Therefore, personality measures capture overall diverse range which indicates that participants do not uniformly represent specific traits.

18. Variable names for personality traits are different in Table 2 and 3. Please adjust.

Our response: Thanks for pointing this. The Tables have been rectified accordingly.

19. Table 5 includes a variable "Expertise" but that variable was not described in the texts. Please describe appropriately.

Our response: We apologize for this inadvertent inclusion. The variable "Expertise" has been excluded from Table 5.

20. Lines 431-433: "These results suggest that individuals with higher levels of imagination, a greater appreciation for aesthetic experiences, and a strong inclination towards art demonstrated..." This claim is not quite warranted. Did you measure imagination? "appreciation for aesthetic experiences" ? Did you measure inclination towards art? It seems you did not measure those, so how can you make any claims about those?

Our response: Thanks for your valuable comment. Openness, as operationalised by the NEO Personality Inventory-Revised (Costa & McCrae, 2008), is characterized by a range of attributes: a vivid imagination (O1: Fantasy), an appreciation of art and beauty (O2: Aesthetics), depth of emotions (O3: Feelings), an eagerness to try new things (O4: Actions), intellectual curiosity (O5: Ideas), and being liberal (O6: Values). Previous research has shown that openness involves a deep engagement with perceptual and aesthetic domains (DeYoung et al., 2014). Further, it is the best predictor of positive attitudes towards aesthetics and active participation in aesthetic activities, such as visiting museums, reading literature, and creating artwork (Meyer et al., 2023; McCrae & Costa, 1997; Chamorro-Premuzic & Furnham, 2004; Kuiken et al., 2021). Notably, greater involvement in aesthetic activities is often related to higher scores in the openness trait (McManus & Furnham, 2006).

Our findings reveal that individuals with higher levels of openness place more emphasis on aesthetic appeal when assessing the creativity of poems, in contrast to those with lower levels of openness.

21. Line 465-466; "Our study suggests that as the level of ambiguity increased with the rise in the aesthetic appeal of a poem,..." You did not measure levels of ambiguity. So why do you make such unwarranted claims?

Our response: We appreciate your comment. We removed the line: "In addition….appreciation of poems" from the Discussion section.

22. Lines 485-486: "may have been more fostered by the intrinsic drive of highly curious readers to explore the unknown" To 'explore the unknown' by reading a short poem in 30 seconds? Are you serious?

Our response: Thank you for your comment. However, we would like to clarify that the 30-second timeframe set in our study is the minimum, rather than the maximum, duration allotted for reading each poem. This approach ensures that participants must have spent at least 30 sec or more to engage with the poem. During the evaluation phase, there was no imposed time limit, and the poem remained visible, facilitating a more thorough assessment and appreciation of the poem's nuances without time pressure. Hence, this extended interaction provides an opportunity for the participants to delve into aspects of the unfamiliar depicted in the poem, potentially stimulating their natural curiosity and desire for knowledge.

Furthermore, our study focuses on epistemic curiosity, a personality trait, characterized by a desire for new information that motivates exploratory behaviour and knowledge acquisition (Berlyne, 1954; Loewenstein, 1994; Litman & Spielberger, 2003; Litman et al., 2005). This is distinct from curiosity as a transient state, which is a stimulus-driven motivational state to acquire new information(Berlyne, 1954; Collins et al., 2004). Therefore, while a brief poem may not necessarily provoke a perceptual or stimulus-driven state of curiosity in the participants, it has the potential to activate their intrinsic exploratory behavioral traits, encouraging a deeper intellectual engagement with the content.

Hence we rewrote the line as: "**The contribution of aesthetic appeal appreciation to the creativity judgment may have been more fostered by the intrinsic behavioural traits of highly curious readers which compel them to delve more deeply into the unfamiliar aspects of the poems."**

23. Lines 488-489 "the positive influence of surprise on creativity..." 'Positive influence' is a very ambiguous and term. Consider rephrasing.

Our response: Thanks for this suggestion. We have now modified the line as: **"the influence of surprise on creativity judgment was more pronounced among individuals with lower levels of curiosity."**

24. Lines 494-496: "Firstly, it suggests that emotions such as amazement, wonder, information-seeking curiosity, and knowledge hunger, when experienced at higher levels, have a stronger impact" You did not measure any of those. Why are you extrapolating?

Our response: We agree with your comment. We computed personality traits related to those emotions using self-reports on valid questionnaires. A substantial body of research supports the method of self-reports in personality research (Shiota et al., 2007; Belfi et al., 2018; Hitsuwari & Nomura, 2022; Paulhus & Vazire, 2007; Robins et al., 2007). However, we omitted those lines in the revised manuscript.

25. Please revise the Discussion section with relevant claims and avoid any unwarranted and whimsical claims. Please try to make the discussion more scientific and less poetic.

Our response: The "Discussion" section has been thoroughly revised and modified as suggested.

26. Regarding the lack of effect of 'arousal'. Could it be that participants did not understand what it is supposed to be?

Our response: In the instruction page, for arousal, participants were asked: "How stimulating (higher Scores) or relaxing (lower Scores) did you feel when you read the poem?" (Belfi et al., 2018; Mehl et al., 2023; Hitsuwari & Nomura, 2022; Frame et al., 2023). Thus, we anticipated that participants comprehended the concept of arousal.

Of note, despite exhibiting significance (b = 0.07, SE = 0.02, t = 4.28, p < 0.001), the inclusion of arousal did not substantially improve the explanatory predictive power of the model which led to its exclusion as a viable predictor (see Model 4 in the Table 3 below for comparison of models). Therefore, the lack of a univariate effect for arousal effect was not the actual reason to exclude arousal as a potential predictor.

Table 3. Model comparison to identify the best model fit comprising aesthetic appeal, felt valence, and surprise.

Information Criteria	Null Model	Model 1	Model 2	Model 3	Model 4	Model 5
AIC	11160.3	9586.5	9325.2	9032.7	9016.8	9018.4
BIC	11178.7	9611.1	9356	9069.6	9059.8	9067.6
R^2	0	0.26	0.29	0.33	0.33	0.33
$\Delta\chi^2$		1575.73***	263.277***	294.514***	17.962***	0.401

Note: Aesthetic appeal, felt valence, surprise, arousal and clarity are included sequentially to Model 1 to Model 5; all models are compared hierarchically, i.e., Model 1 is compared to Null Model, Model 2 is compared to Model 1 and so on ; AIC = Akaike Information Criterion; BIC = Bayesian Information Criterion; R^2 = proportion of variation explained by fixed effects (Nakagawa & Schielzeth, 2013); Δχ2 = Likelihood ratio test statistic for comparison of models. Significance codes: 0 '***' 0.001 '**' 0.01 '*' 0.05 '.' 0.1 ' ' 1

27. About limitations (from line 524) – please set a new section with title "Limitations"

Our response: Thanks for the suggestion. We set a new section with the title "Limitations" as advised.

Our responses to the Reviewer's comments

1: Used terminology: Most problematic is, in my view, the use of the term "creativity". In my opinion, creativity is a state of mind or a quality of people (or – more precisely – a quality of humans that allows them to create something new). Weisberg – for example – defines "creativity" as (a) "the capacity to produce [novel] works [with value]" and (b) "the activity of generating such products" (in: Robert W. Weisberg: Creativity, p. 4). But it is NOT a property of works of art. However, in the sentence: "... different individuals may hold contrasting views on the creativity of a particular poem ...", creativity is spoken of as if it were an inherent property of poems, not an ability of the person who wrote the poem. Similar is the use of the word throughout the article. This is relevant to the article in that it is not entirely clear what exactly is being measured. Particularly, as the concept and its operationalisation are not explained anywhere. The item used in the questionnaire to assess "creativity" again treats it as an inherent quality of the poems. For a better understanding of the study, it would therefore be imperative to discuss the unconventional use of the term, its implied meaning and its possible impact on the participants of the study. In the literature cited in the manuscript "creativity" is - as far as I know – also seen as a characteristic of people (i.e., the ability of people to be creative). The connection between creativity and basic character traits found in other studies is therefore only indirectly related to this study, if at all. In other studies, various characteristics of an individual were related to each other (e.g., is extroversion related to creativity?). The present study, on the other hand, investigated which character traits of the viewers had an influence on the evaluation of the poems as ... - well, what exactly? I think it is therefore urgent to **(a) explain how the term "creativity" is used in the article, (b) critically analyse the relationship between the use of the term in the article and in the cited literature**, and **(c) discuss in detail the potential effects of its unconventional operationalisation on the results of the study**. Note: Despite my reservations, I use the term "creativity" in a similar way as it is used in the manuscript. However, this is simply for lack of alternatives, as I am not quite sure what exactly was measured in the study.

Our response: We would like to thank you for your insightful comments. We understand your concern regarding the operationalization of creativity in our study. Let us provide further clarifications as follows.

The concept of creativity as articulated by Mel Rhodes in 1961, through his 4-P model of creativity, offers a multifaceted perspective. Rhodes described creativity as a phenomenon where an individual (Person) communicates a new concept (Product), underpinned by mental activity (Process) and environmental factors (Press) (Rhodes, 1961). In this model, creativity is defined through four lenses: person, product, process, and press. In our study, we specifically focused on the 'product' aspect of creativity, specifically poetry, and operationalized "creativity" as the poem's creative potential. Of note, our approach is in line with numerous past studies which have investigated the creativity evaluation of various types of products/artefacts, such as ideas (Lloyd-Cox et al., 2022) musical compositions (Pinho et al., 2014; Zioga et al., 2020) short stories (Toivainen et al., 2021) and product concepts (Guenther et al., 2021), to name a few. This approach enabled us to explore the internal

model of how individuals judge the creativity of poems, recognizing that such judgments may be inherently subjective and influenced by individual personality traits.

We acknowledge that this operationalization the reviewer has found is different from the more traditional view of creativity as a characteristic of individuals. This choice was made to expand the understanding of creativity beyond the creator to the creation itself. In this study (and in numerous others which have looked at the creativity evaluation of a diverse range of artefacts/ideas), the adopted approach seeks to explore how creativity can also be perceived as an inherent quality of artistic works, in this case, poems. This perspective aligns with our aim to understand how different individuals perceive and evaluate the creativity of a poem based on their personality traits.

Further, we believe that viewing creativity as a characteristic of poems rather than solely a human trait offers a novel insight into how aesthetic appreciation and personality traits interplay in the perception and evaluation of poems. Further, this approach also allowed us to investigate how individual differences in traits like openness, intellect, awe-proneness, and epistemic curiosity influence the creativity evaluation of poems.

In conclusion, our study is a deliberate choice aimed at enriching the understanding of creativity evaluation in the context of poetry. By focusing on the product and the individual's interaction with it, we aimed to uncover nuanced aspects of how creativity is perceived and evaluated in the realm of English poems.

We deeply acknowledge Reviewer's comment and included this point in the "Limitations" section as follows:

"Finally, we acknowledge that the operationalization of 'creativity' is different from the more traditional view of creativity as a characteristic of individuals. Considering that creativity, as per the 4P Model (Rhodes, 1961), is defined through four lenses: person, product, process, and press, we strategically focused on the 'product' aspect of creativity, particularly in the context of poetry. We, here operationalized 'creativity' as the poem's creative potential. This choice was made to expand the understanding of creativity beyond the creator to the creation itself. Our approach is in line with numerous past studies which have investigated the creativity evaluation of various types of products/artefacts, such as ideas (Lloyd-Cox et al., 2022), musical compositions (Pinho et al., 2014; Zioga et al., 2020) short stories (Toivainen et al., 2021), and product concepts (Guenther et al., 2021), to name a few. This approach enabled us to explore the internal model of how individuals judge the creativity of poems, recognizing that such judgments may be inherently subjective and influenced by individual personality traits."

2: Operationalisation of the factors: The other variables for recording the aesthetic quality of the poems were also - if I understand correctly - assessed by only one item. Here, too, the question arises as to whether the operationalisation of the question is appropriate. Do the study participants understand what is meant when they have to evaluate whether a poem is "clear"? To what extent does the interpretation of the question vary individually? Are there studies that have examined the validity of these items to capture the underlying concept?

Our response: We appreciate your concern about the operationalization of variables and the potential variability in how study participants interpreted single-item assessments. In our study, participants were asked to evaluate each poem based on the following single-item measures:

1. Clarity: How clear was the content of the poem?

2. Aesthetic appeal: How aesthetically appealing was the poem?

3. Felt valence: How positive (higher scores) or negative (lower scores) did you feel when you read the poem?

4. Felt arousal: How stimulating (higher scores) or relaxing (lower scores) did you feel when you read the poem?

5. Surprise: How Surprising was this poem? By "Surprise" we mean a contrast to expectation in the concept of the poem.

6. Creativity: How Creative was this poem? Please answer based on your intuitive understanding of creativity of poem.

We acknowledge that the individual interpretation of these questions was not explicitly studied in our experiment. However, the use of such single-tem measures is a common practice in the psychometrics of creativity and aesthetics (Lloyd-Cox et al., 2022; Chamberlain et al., 2018; Strijbosch et al., 2022); importantly, single-item measures have also been routinely used in the evaluation of poetry (Belfi et al., 2018; Frame et al., 2023; Mehl et al., 2023; Hitsuwari & Nomura, 2022a, 2022b, 2022c; Papp-Zipernovszky et al., 2021). Nevertheless, we have discussed this issue in the “Limitations” section of the manuscript as follows:

“…we assessed the variables using single-item measures, a common practice in assessing aesthetics in visual art (Hassenzahl, 2004; Chamberlain et al., 2018; Strijbosch et al., 2022), poetry (Belfi et al., 2018; Frame et al., 2023; Mehl et al., 2023; Hitsuwari & Nomura, 2022a, 2022b, 2022c; Papp-Zipernovszky et al., 2021), and music (Zhang & Schubert, 2019). However, we also recognize the potential variability in individual interpretation of the questions remains unexplored. Employing multiple items for variable assessment could have offered psychometric advantages, particularly in enhancing reliability and validity (Bergkvist & Rossiter, 2007).”

3: Selection of poems: The number of poems used for the study is rather small, 36, and - probably - not representative. Especially since the poems were "carefully selected" (by whom, actually?), it is to be feared that the choice of poems might have influenced the result. I understand that this is a necessary sacrifice to make in empirical studies. Especially since the study design almost certainly excludes an influence of the selection of the poems on the result. But I think the issue should at least be discussed as one of the shortcomings of the study. It would also be helpful if it could be shown what measures were taken to ensure that the selection was not biased.

Our response: Many thanks for this comment with which we agree. The chosen set of poems in our study does not constitute a fully representative sample of English poems, which could limit the generalizability of the results to a certain extent. In the revised manuscript, we have now included full details of the poem selection procedure as follows:

“Initially, we selected 108 poems from various online resources, including Poetry.org (http://www.poetry.org/), the Poetry Foundation (https://www.poetryfoundation.org/), and the Academy of American Poets (https://poets.org/). These poems were subsequently rated for "surprise" on a scale of 1 to 7 by M.D, one of the co-authors who is a Professor of Creative Writing with domain-specific expertise and an award-winning poet. Following this evaluation, we shortlisted 36 as the experimental stimuli: 18 with low surprise ratings (4 or lower) and 18 with high surprise ratings (6 or above). The chosen poems varied in structure, contents, lines, and word count (mean number of lines = 11, SD = 3.24; mean word count = 71.25, SD = 28.99). To represent a broad spectrum of English poems, we consciously chose not to limit our selection to a particular genre or form, like haiku or sonnets as done in previous studies (Belfi et al., 2018; Hitsuwari & Nomura, 2022; Papp-Zipernovszky et al., 2021).”

Minor issues: 1. Line 49: It is not clear to me what you mean by “poetry and its creative evaluation has not been adequately characterized”. First of all, it seems to me that it is not the "creative evaluation" that needs further investigation, but rather the "evaluation of creativity". It also seems that the authors have not considered a whole field of science that deals with this issue of creativity in poems. The authors could look at studies on foregrounding or the field of stylistics. Another research approach that would be relevant in this context is the work of Menninghaus and colleagues.

Our response: Thanks – you are right. We have changed the phrase “creative evaluation” to “evaluation of creativity”.

We appreciate your suggestion to explore foregrounding and the domain of stylistics. In future, conducting studies to investigate the influence of poetry affinity (Kraxenberger & Menninghaus, 2017), or the use of poetic language on various dimensions of emotional responses (e.g., joy, sadness, being moved, intensity, and positive affect), as well as aesthetic appreciation (e.g., beauty, liking, and melodiousness) would be valuable (Menninghaus et al., 2017).

2. Line 50 to 54: It is also not entirely clear to me why the subjectivity of aesthetic evaluation should be more pronounced in poetics than in other forms of art. Personal experience always plays a role in the reception of art, regardless of the medium or art form, doesn't it? Furthermore, it seems to me that the introduction does not clearly separate the concepts of "beauty", "aesthetics" and "creativity". “Beauty” is in the eyes of the beholder. Why then do different observers have different views of the “creativity” with which a poem was written? Are "beauty" and "creativity" the same thing? Or are they causally linked? And if we already know that what is the point of the study?

Our response: We highly appreciate and acknowledge your comment on the fundamental aspect of aesthetics. As mentioned in the article, the main focus of the study is to investigate how individual difference in specific personality traits, specifically Openness, Intellect, Awe-proneness, and Epistemic Curiosity, shape the implicit associations between subjective experiences and the judgment of creativity of English poems.

While we acknowledge the importance of the age-old questions mentioned by the reviewer, we have purposefully avoided answering them as they are beyond the scope of this current study.

3. Line 62-64: In much of the introduction, the authors make assertions without citing any valid references. For example, I do not understand why "creativity" should be a less tangible construct than "preference".

Our response: We acknowledge your comment. We omitted the lines in the revised manuscript.

4. Line 79 (and others): Some sentences end without a period or have commas before the period (line 102).

Our response: Thanks. We have corrected as appropriate.

5. Line 457-467: In the discussion, an indirect connection is made between the ambiguity of expression in a poem and aesthetic appeal via the evaluation of "awe-proneness". It is stated that "other studies" have reported a connection between "awe-proneness" and the ability to tolerate ambiguity. But, on the one hand, this seems to concern only one study, and on the other hand, this is an indirectly established connection. I therefore think it is appropriate to formulate the conclusion (which I do not want to doubt) more cautiously (lines 465 and 466). In order to substantiate the thesis a little, it might be helpful to support it a little with findings from the studies on "foregrounding" (British stylistics or Russian formalism).

Our response: We acknowledge your valuable advice. We have now excluded the lines reporting a connection between awe-proneness and ambiguity tolerance.

References:

Acar, S., Burnett, C., & Cabra, J. F. (2017). Ingredients of creativity: Originality and more. *Creativity Research Journal*, *29*(2), 133–144. https://doi.org/10.1080/10400419.2017.1302776

Barron, F. (1955). The disposition toward originality. *The Journal of Abnormal and Social Psychology*, *51*, 478–485. https://doi.org/10.1037/h0048073

Belfi, A. M., Vessel, E. A., & Starr, G. G. (2018). Individual ratings of vividness predict aesthetic appeal in poetry. *Psychology of Aesthetics, Creativity, and the Arts*, *12*(3), 341–350. https://doi.org/doi:10.1037/aca0000153

Bergkvist, L., & Rossiter, J. R. (2007). The predictive validity of multiple-item versus single-item measures of the same constructs. *Journal of Marketing Research*, *44*(2), 175–184. https://doi.org/https://doi.org/10.1509/jmkr.44.2.175

Berlyne, D. E. (1954). A theory of human curiosity. *British Journal of Psychology*, *45*(3), 180.

Chamberlain, R., Mullin, C., Scheerlinck, B., & Wagemans, J. (2018). Putting the art in artificial: Aesthetic responses to computer-generated art. *Psychology of Aesthetics, Creativity, and the*

Arts, *12*(2), 177. https://doi.org/https://psycnet.apa.org/doi/10.1037/aca0000136

Chamorro-Premuzic, T., & Furnham, A. (2004). Art judgment: a measure related to both personality and intelligence? *Imagination, Cognition and Personality*, *24*(1), 3–24. https://doi.org/https://doi.org/10.2190/U4LW-TH9X-80M3-NJ54

Chamorro-Premuzic, T., Reimers, S., Hsu, A., & Ahmetoglu, G. (2009). Who art thou? Personality predictors of artistic preferences in a large UK sample: The importance of openness. *British Journal of Psychology*, *100*(3), 501–516. https://doi.org/https://doi.org/10.1348/000712608X366867

Chotlos, J. W. (1944). IV. A statistical and comparative analysis of individual written language samples. *Psychological Monographs*, *56*(2), 75. https://doi.org/https://psycnet.apa.org/doi/10.1037/h0093511

Collins, R. P., Litman, J. A., & Spielberger, C. D. (2004). The measurement of perceptual curiosity. *Personality and Individual Differences*, *36*(5), 1127–1141. https://doi.org/https://doi.org/10.1016/S0191-8869(03)00205-8

Costa Jr, P. T., & McCrae, R. R. (1992). Four ways five factors are basic. *Personality and Individual Differences*, *13*(6), 653–665. https://doi.org/https://doi.org/10.1016/0191-8869(92)90236-I

Costa, P. T., & McCrae, R. R. (2008). The revised neo personality inventory (neo-pi-r). *The SAGE Handbook of Personality Theory and Assessment*, *2*(2), 179–198.

DeYoung, C. G., Shamosh, N. A., Green, A. E., Braver, T. S., & Gray, J. R. (2009). Intellect as distinct from openness: Differences revealed by fMRI of working memory. *Ournal of Personality and Social Psychology*, *97*(5), 883–892. https://doi.org/https://psycnet.apa.org/doi/10.1037/a0016615

DeYoung, C. G., Grazioplene, R. G., & Peterson, J. B. (2012). From madness to genius: The Openness/Intellect trait domain as a paradoxical simplex. *Journal of Research in Personality*, *46*(1), 63–78. https://doi.org/https://doi.org/10.1016/j.jrp.2011.12.003

DeYoung, C. G., Peterson, J. B., & Higgins, D. M. (2005). Sources of openness/intellect: Cognitive and neuropsychological correlates of the fifth factor of personality. *Journal of Personality*, *73*(4), 825–858. https://doi.org/https://doi.org/10.1111/j.1467-6494.2005.00330.x

DeYoung, C. G., Quilty, L. C., & Peterson, J. B. (2007). Between facets and domains: 10 aspects of the Big Five. *Journal of Personality and Social Psychology*, *93*(5), 880.

DeYoung, C. G., Quilty, L. C., Peterson, J. B., & Gray, J. R. (2014). Openness to Experience, Intellect, and Cognitive Ability. *Journal of Personality Assessment*, *96*(1), 46–52. https://doi.org/10.1080/00223891.2013.806327

Faul, F., Erdfelder, E., Lang, A.-G., & Buchner, A. (2007). G* Power 3: A flexible statistical power analysis program for the social, behavioral, and biomedical sciences. *Behavior Research Methods*, *39*(2), 175–191.

Frame, J., Mehl, K., Head, K., & Belfi, A. (2023). *The influence of sensory modality on aesthetic judgments of poetry*. https://doi.org/https://doi.org/10.31234/osf.io/cv6px

Gabrielsson, A. (2001). Emotion perceived and emotion felt: Same or different? *Musicae Scientiae*, *5*(1_suppl), 123–147. https://doi.org/10.1177/10298649020050S105

Guenther, A., Eisenbart, B., & Dong, A. (2021). Creativity and successful product concept selection for innovation. *International Journal of Design Creativity and Innovation*, *9*(1), 3–19. https://doi.org/https://doi.org/10.1080/21650349.2020.1858970

Hassenzahl, M. (2004). The interplay of beauty, goodness, and usability in interactive products. *Human–Computer Interaction*, *19*(4), 319–349. https://doi.org/https://doi.org/10.1207/s15327051hci1904_2

Hitsuwari, J., & Nomura, M. (2022). How individual states and traits predict aesthetic appreciation of haiku poetry. *Empirical Studies of the Arts*, *40*(1), 81–99. https://doi.org/10.1177/0276237420986420

Jacobs, A. M. (2017). Quantifying the beauty of words: A neurocognitive poetics perspective. In *Frontiers in Human Neuroscience* (Vol. 11). https://www.frontiersin.org/articles/10.3389/fnhum.2017.00622

Jacobs, A. M., & Kinder, A. (2018). What makes a metaphor literary? Answers from two computational studies. *Metaphor and Symbol*, *33*(2), 85–100.

Johnson, D. R., Kaufman, J. C., Baker, B. S., Patterson, J. D., Barbot, B., Green, A. E., van Hell, J., Kennedy, E., Sullivan, G. F., Taylor, C. L., Ward, T., & Beaty, R. E. (2022). Divergent semantic integration (DSI): Extracting creativity from narratives with distributional semantic modeling. *Behavior Research Methods*. https://doi.org/10.3758/s13428-022-01986-2

Kraxenberger, M., & Menninghaus, W. (2017). Affinity for poetry and aesthetic appreciation of joyful and sad poems. In *Frontiers in Psychology* (Vol. 7). https://www.frontiersin.org/articles/10.3389/fpsyg.2016.02051

Kuiken, D., Douglas, S., & Kuijpers, M. (2021). Openness to experience, absorption-like states, and the aesthetic, explanatory, and pragmatic effects of literary reading. *Scientific Study of Literature*, *11*(2), 148–195. https://doi.org/https://doi.org/10.1075/ssol.21007.kui

Lau, J. H., Cohn, T., Baldwin, T., Brooke, J., & Hammond, A. (2018). *Deep-speare: A joint neural model of poetic language, meter and rhyme*. https://doi.org/10.48550/arxiv.1807.03491

Litman, J. A., & Spielberger, C. D. (2003). Measuring epistemic curiosity and its diversive and specific components. *Journal of Personality Assessment*, *80*(1), 75–86. https://doi.org/10.1207/S15327752JPA8001_16

Litman, J., Hutchins, T., & Russon, R. (2005). Epistemic curiosity, feeling-of-knowing, and exploratory behaviour. *Cognition and Emotion*, *19*(4), 559–582. https://doi.org/10.1080/02699930441000427

Lloyd-Cox, J., Pickering, A., & Bhattacharya, J. (2022). Evaluating creativity: How idea context and rater personality affect considerations of novelty and usefulness. *Creativity Research Journal*, *34*(4), 373–390. https://doi.org/10.1080/10400419.2022.2125721

Loewenstein, G. (1994). The psychology of curiosity: A review and reinterpretation. *Psychological Bulletin*, *116*(1), 75.

Malvern, D. D., & Richards, B. J. (1997). A new measure of lexical diversity. *British Studies in Applied Linguistics*, *12*, 58–71.

Marin, M. M., & Bhattacharya, J. (2010). Music induced emotions: some current issues and cross-modal comparisons. In J. Hermida & M. Ferrero (Eds.), *Music education* (pp. 1–38). Hauppauge, NY: Nova Science Publishers.

Mastria, S., Agnoli, S., & Corazza, G. E. (2019). How does emotion influence the creativity evaluation of exogenous alternative ideas? *PLOS ONE*, *14*(7), e0219298. https://doi.org/10.1371/journal.pone.0219298

McCarthy, P. M., & Jarvis, S. (2007). vocd: A theoretical and empirical evaluation. *Language Testing*,

24(4), 459–488. https://doi.org/https://doi.org/10.1177/0265532207080767

McCarthy, P. M., & Jarvis, S. (2010). MTLD, vocd-D, and HD-D: A validation study of sophisticated approaches to lexical diversity assessment. *Behavior Research Methods*, *42*(2), 381–392. https://doi.org/10.3758/BRM.42.2.381

McCrae, R. R., & Costa Jr, P. T. (1997). Conceptions and correlates of openness to experience. In *Handbook of personality psychology*. Academic Press. https://doi.org/https://doi.org/10.1016/B978-012134645-4/50032-9

McCrae, R. R. (1996). Social consequences of experiential openness. *Psychological Bulletin*, *120*(3), 323–337. https://doi.org/https://psycnet.apa.org/doi/10.1037/0033-2909.120.3.323

McCrae, Robert R. (2007). Aesthetic chills as a universal marker of openness to experience. *Motivation and Emotion*, *31*(1), 5–11. https://doi.org/10.1007/s11031-007-9053-1

McManus, I. C., & Furnham, A. (2006). Aesthetic activities and aesthetic attitudes: Influences of education, background and personality on interest and involvement in the arts. *British Journal of Psychology*, *97*(4), 555–587. https://doi.org/https://doi.org/10.1348/000712606X101088

Mehl, K., Gugliano, M., & Belfi, A. M. (2023). The role of imagery and emotion in the aesthetic appeal of music, poetry, and paintings. *Psychology of Aesthetics, Creativity, and the Arts*, No Pagination Specified-No Pagination Specified. https://doi.org/10.1037/aca0000623

Menninghaus, W., Wagner, V., Wassiliwizky, E., Jacobsen, T., & Knoop, C. A. (2017). The emotional and aesthetic powers of parallelistic diction. *Poetics*, *63*, 47–59. https://doi.org/https://doi.org/10.1016/j.poetic.2016.12.001

Meyer, W. U., Reisenzein, R., & Schützwohl, A. (1997). Toward a process analysis of emotions: The case of surprise. *Motivation and Emotion*, *21*(3), 251–274. https://doi.org/https://doi.org/10.1023/A:1024422330338

Meyer, J., Thoma, G.-B., Kampschulte, L., & Köller, O. (2023). Openness to experience and museum visits: Intellectual curiosity, aesthetic sensitivity, and creative imagination predict the frequency of visits to different types of museums. *Journal of Research in Personality*, *103*, 104352. https://doi.org/https://doi.org/10.1016/j.jrp.2023.104352

Meyer, W.-U., Niepel, M., Rudolph, U., & Schützwohl, A. (1991). An experimental analysis of surprise. *Cognition & Emotion*, *5*(4), 295–311.

Meyer, W.-U., Reisenzein, R., & Schützwohl, A. (1997). Toward a process analysis of emotions: The case of surprise. *Motivation and Emotion*, *21*(3), 251–274. https://doi.org/10.1023/A:1024422330338

Nakagawa, S., & Schielzeth, H. (2013). A general and simple method for obtaining R2 from generalized linear mixed-effects models. *Methods in Ecology and Evolution*, *4*(2), 133–142.

Noordewier, M. K., & Breugelmans, S. M. (2013). On the valence of surprise. *Cognition and Emotion*, *27*(7), 1326–1334. https://doi.org/10.1080/02699931.2013.777660

Nusbaum, E. C., & Silvia, P. J. (2011). Are Openness and Intellect distinct aspects of Openness to Experience? A test of the O/I model. *Personality and Individual Differences*, *51*(5), 571–574. https://doi.org/https://doi.org/10.1016/j.paid.2011.05.013

Obermeier, C., Menninghaus, W., von Koppenfels, M., Raettig, T., Schmidt-Kassow, M., Otterbein, S., & Kotz, S. (2013). Aesthetic and emotional effects of meter and rhyme in poetry. In *Frontiers in Psychology* (Vol. 4). https://www.frontiersin.org/articles/10.3389/fpsyg.2013.00010

Oleynick, V. C., DeYoung, C. G., Hyde, E., Kaufman, S. B., Beaty, R. E., & Silvia, P. J. (2017). Openness/intellect: The core of the creative personality. In *The Cambridge handbook of creativity and personality research.* (pp. 9–27). Cambridge University Press. https://doi.org/10.1017/9781316228036.002

Papp-Zipernovszky, O., Mangen, A., Jacobs, A., & Lüdtke, J. (2021). Shakespeare sonnet reading: An empirical study of emotional responses. *Language and Literature*, *31*(3), 296–324. https://doi.org/10.1177/09639470211054647

Paulhus, D. L., & Vazire, S. (2007). The self-report method. *Handbook of Research Methods in Personality Psychology*, *1*(2007), 224–239.

Pietras, K., & Ganczarek, J. (2022). Aesthetic reactions to violations in contemporary art: The role of expertise and individual differences. *Creativity Research Journal*, *34*(3), 324–338.

Pinho, A. L., de Manzano, Ö., Fransson, P., Eriksson, H., & Ullén, F. (2014). Connecting to create: expertise in musical improvisation is associated with increased functional connectivity between premotor and prefrontal areas. *Journal of Neuroscience*, *34*(18), 6156–6163. https://doi.org/https://doi.org/10.1523/JNEUROSCI.4769-13.2014

Rasse, C., Onysko, A., & Citron, F. M. M. (2020). Conceptual metaphors in poetry interpretation: A psycholinguistic approach. *Language and Cognition*, *12*(2), 310–342. https://doi.org/10.1017/langcog.2019.47

Rhodes, M. (1961). An analysis of creativity. *The Phi Delta Kappan*, *42*(7), 305–310. https://doi.org/https://www.jstor.org/stable/20342603

Robins, R. W., Tracy, J. L., & Sherman, J. W. (2007). What kinds of methods do personality psychologists use. *Handbook of Research Methods in Personality Psychology*, 673–678.

Rumelhart, D. E. (1984). *Schemata and the cognitive system.*

Runco, M. A., & Jaeger, G. J. (2012). The Standard Definition of Creativity. *Creativity Research Journal*, *24*(1), 92–96. https://doi.org/10.1080/10400419.2012.650092

Russell, J. A. (1980). A circumplex model of affect. *Journal of Personality and Social Psychology*, *39*(6), 1161.

Shiota, M. N., Keltner, D., & Mossman, A. (2007). The nature of awe: Elicitors, appraisals, and effects on self-concept. *Cognition and Emotion*, *21*(5), 944–963. https://doi.org/10.1080/02699930600923668

Simonton, D. K. (2012). Taking the U.S. patent office criteria seriously: A quantitative three-criterion creativity definition and its implications. *Creativity Research Journal*, *24*(2–3), 97–106. https://doi.org/10.1080/10400419.2012.676974

Steen, G. J. (2009). From linguistic form to conceptual structure in five steps: Analyzing metaphor in poetry. *Cognitive Poetics*, 197–226.

Stein, M. I. (1953). Creativity and culture. *The Journal of Psychology*, *36*(2), 311–322. https://doi.org/http://dx.doi.org/10.1080/00223980.1953.9712897

Strijbosch, W., Vessel, E. A., Welke, D., Mitas, O., Gelissen, J., & Bastiaansen, M. (2022). On the neuronal dynamics of aesthetic experience: Evidence from electroencephalographic oscillatory dynamics. *Journal of Cognitive Neuroscience*, *34*(3), 461–479. https://doi.org/https://doi.org/10.1162/jocn_a_01812

Toivainen, T., Madrid-Valero, J. J., Chapman, R., McMillan, A., Oliver, B. R., & Kovas, Y. (2021).

Creative expressiveness in childhood writing predicts educational achievement beyond motivation and intelligence: A longitudinal, genetically informed study. *British Journal of Educational Psychology*, *91*(4), 1395–1413. https://doi.org/https://doi.org/10.1111/bjep.12423

Wassiliwizky, E., Koelsch, S., Wagner, V., Jacobsen, T., & Menninghaus, W. (2017). The emotional power of poetry: neural circuitry, psychophysiology and compositional principles. *Social Cognitive and Affective Neuroscience*, *12*(8), 1229–1240. https://doi.org/10.1093/scan/nsx069

Zhang, J. D., & Schubert, E. (2019). A Single Item Measure for Identifying Musician and Nonmusician Categories Based on Measures of Musical Sophistication. *Music Perception*, *36*(5), 457–467. https://doi.org/10.1525/mp.2019.36.5.457

Zioga, I., Harrison, P. M. C., Pearce, M. T., Bhattacharya, J., & Di Bernardi Luft, C. (2020). From learning to creativity: Identifying the behavioural and neural correlates of learning to predict human judgements of musical creativity. *NeuroImage*, *206*, 116311. https://doi.org/https://doi.org/10.1016/j.neuroimage.2019.116311
