# Supplementary material for: Beyond the words: Exploring individual differences in the evaluation of poetic creativity
Source: PLoS One. 2024 Oct 3;19(10):e0307298. doi: 10.1371/journal.pone.0307298 (PMC11449365; doi:10.1371/journal.pone.0307298)
Supplement: Attachment — Submitted filename: PloS1_R2_RESPONSE_REVIEWERS.docx [file pone.0307298.s004.docx]

**Our Responses to the comments of the Editor and the Reviewers**

Thank you very much for giving us an opportunity to revise the manuscript. Below are our responses to the comments made by the Academic Editor and the Reviewers. The Editor’s and the Reviewers’ comments are shown in dark red, our responses are in regular font, and the inclusions in the revised manuscript are in **bold black** (both here and in the revised manuscript).

**Major modifications made in the revised manuscript:**

1. We have integrated the definition of creativity, encompassing both the 4P model and the standard definition, into the Introduction, highlighting its relevance to our research.

2. Table1 containing the descriptive statistics has been separated into 2 tables: Table 1a and Table 1b, containing the descriptive statistics of the ratings on poems and the descriptive statistics of the personality traits, respectively.

3. Table 3 for bivariate correlations has been updated

4. Table 6 has been updated to include an additional section that presents the statistical findings for the slope differences.

5. The scales of the interaction plots have been adjusted.

6. The Discussion section has been restructured to enhance clarity and facilitate easier comprehension.

**Responses to the Editor’s comments**

**1.Lines 207-209:** *“we shortlisted 36 poems as the experimental stimuli: 18 with low surprise ratings (4 or lower) and 18 with high surprise ratings (6 or above).*

**Editor’s comment:** The ratings '4 or lower and 6 or above' – who gave them? M.D.? What scale was used? Please describe shortly.

**Our response:** Thank you for your valuable comment. The initially selected **poems were evaluated for their levels of "surprise" by M.D., a Professor of English and Creative Writing with domain-specific expertise, using a scale of 1 to 7, where 1 indicates “absolutely not surprising” and 7 indicates “absolutely surprising.”** This has now been incorporated in the Materials and Methods section.

**2.** **Line 273:** “onpersonality traits” - space missing

**Our response:** Thank you. This has been corrected.

**3. Major issue.** There is a problem with Table 1 "Descriptive statistics". It shows statistics for 10 variables, all of them measured with 7-point scales . However the variables are not of the same type. The first 6 variables are ratings on poems, each on a 7-point scale as stated in the manuscript earlier. Their count (n) values are 3456 data points, that is36 poems x 96 participants. However the last four variables are personality measures, their count (n) should be 96, not 3456. I presume that was a 'typo', but:..... Please recheck your statistics carefully!!! Please involve all co-authors in rechecking the manuscript. Please separate Table 1 into two (similar) tables (or table 1a and 1b), one for ratings on poems, and one for personality traits. Include those labels on table captions. Also mention in the manuscript explicitly that personality traits were also measured on 7-point scales (it is hidden in the extra materials).

**Our response:** Thank you for the valuable comment. To address this issue, we have made the following corrections:

(i) We have corrected the typo for count (N) for personality variables from 3456 to 96.
(ii) Table 1 has been split into two separate tables: Table 1a and Table 1b presenting the descriptive statistics for poem-related and personality variables, respectively.
(iii) In the “Procedure” section of the revised manuscript, we have clarified the scale used for scoring personality traits as follows: “**All personality questionnaires utilized a 7-point scale, with 1 representing “disagree strongly” and 7 representing “agree strongly”.**

The separate tables, i.e., Table1a, and Table 1b are as follows:

**Table 1a. Descriptive statistics of the creativity and its potential predictors including mean, standard deviation, skewness, kurtosis, standard error (SE), and variance inflation factor (VIF).**

Note: The VIF for a variable is defined for a set of predictor variables by 1/(1-R^2) where R^2 is for the model predicting the variable from all the other predictor variables.

**Table 1b. Descriptive statistics of readers’ personality trait variables including mean, standard deviation, skewness, kurtosis, standard error (SE)**

**4. Major issue.** Concerning figures 1-4 -all the figures with slopes. Please explain (also to the readers) what are the scales on X- and Y- axes. The original measurement scales were 7-point scales. On the figures we meet what seems to be re-centered scales, but they are not obvious, and in some cases the ranges are -6 to 4, which is strange. One would expect the ranges to be of size six. Explain your transformations.

**Our response:** Thank you for your valuable comments and suggestions. We have modified the scales of the interaction plots. This adjustment and the rationale behind the choice of scales have been mentioned in the “Analysis” section of the revised manuscript, as follows:

**“****The original measurement scales were based on 7-point scales. Before entering the model, five potential predictors were centered within each subject (i.e., group mean-centered) to obtain a clear estimate of the within-group effect (Enders, C. K., & Tofighi, 2007). For interaction plots, it is a standard practice to use a scale that reflects the original range of the variables rather than the centered range. Therefore, on the X-axis, the scales for the predictors (group mean-centered) vary from -7 to +7, while the outcome variable, which remains uncentered, is represented on the Y-axis with a range from 1 to 7.”**

**5. Lines463-465:** *"The current study investigated the moderating effects of four personality traits openness, intellect, awe-proneness, and curiosity - on the evaluation of creativity in English language poems: openness."*

**Editor’s comment:** What is 'openness' doing at the end of the sentence?

**Our response:** Thank you for pointing out this error. It has now been corrected.

**6. Lines 495-498:** *"The distinct moderating effects of openness on aesthetic appeal and felt valence in our study suggest that the aesthetic emotions evoked through affective processing of poetry might differ from the emotional valence experienced during the reading of poetry."*

**Editor’s comment:** I think I understand every word in this sentence, but I don't understand what it means. What is the difference between "affective processing of poetry" and "emotional valence experienced during the reading of poetry”? Please try to clarify this sentence, and the whole discussion section, stating things in very plain language, and taking in mind an audience who are not versed in your jargon.

**Our response:** Thank you for your valuable feedback. The part in the Discussion section has been clarified as follows:

“**Processing of any artwork, including literature, includes a component called “aesthetic emotion” (Chatterjee & Vartanian, 2014; Leder et al., 2004; Jacobs, 2015; Menninghaus et al., 2019). Aesthetic emotions are the discrete emotions that always include an aesthetic evaluation/appreciation and are further associated with subjectively felt pleasure or displeasure, i.e., felt valence, during any emotional episode (Menninghaus et al., 2019). Our study indicates that individuals with higher levels of openness may be less influenced by aesthetic emotions compared** **to those with lower levels of openness while assessing creativity of poems. On the flip side, higher open individuals seem to be more positively impacted by the overall aesthetic appeal of poems compared to those with lower levels of openness. This notion aligns with the understanding that aesthetic appeal appreciation and evaluation of artwork, beyond aesthetic emotions, involves processing of other inherent features of art, such as styles, experience of pleasure of generalization (Leder et al., 2004; Hartley & Homa, 1981; Gordon & Holyoak, 1983), and knowledge (Silvia, 2010; Lachapelle et al., 2003; Cupchik & László, 1992). ”**

The whole discussion section has been restructured for easy comprehension.

**7 Line 525:** *“a finding corroborated by previous research (111)."* Please reconsider your semantics. Your study might corroborate previous research but claiming that previous research corroborates your study is over the top.”

**Our response.** Thank you. This has now been rectified.

**8. Regarding lines 534-557:** There can be an alternative 'explanation': individuals with high openness that are more motivated to learn, might know more, and thus be less surprised in general. They might also be less surprised by the contents and forms in the poems. This could be checked with your data: are the average surprise ratings of open individuals lower than surprise rating of not open individuals? Do low-openness individuals put more emphasis on surprise, or maybe they are just more surprised? Or both? (the two things are not the same).

**Our response:**

Thank you. We appreciate your valuable comment. We specifically discussed the results by examining the simple slopes of the interaction effects involving the personality trait variables. As suggested, we revised the descriptions to include the slope differences for high and low openness as:

“**The statistically significant difference of the simple slopes for high and low open individuals indicates** **that, more open individuals, who are more motivated to learn, might be less influenced by the surprise in the contents of the poems compared to their lower counterpart while judging poetic creativity.The transient and ambiguous nature of surprise might disrupt their affective states, leading to a reduced impact of surprise on their creativity judgment.** **In contrast, less open individuals perceived surprise as a more significant factor in their evaluation of poetic creativity than their higher counterparts, contradicting our initial prediction. It is noteworthy that the interaction does not indicate that high openness readers were less surprised by the poems compared to low openness readers. Rather it suggests that their judgments of a poem’s creativity were less influenced by the surprise element of the poem compared to those with lower openness.** **Furthermore,**  **our focus was not on whether individuals with higher openness rated surprise more highly on average than those with lower openness. Instead, we focused on the differential level of surprise ratings for high and low openness. Our objective was to investigate whether there was a difference in how surprise was prioritized as a predictor of creativity judgment between the two levels of openness.”**

We have incorporated the results of the statistical test for the slope difference (highlighted in green) into the **Table 6** in the revised manuscript, as follows:

**Table 6. Results of simple slopes analyses for the high and low levels of the moderators and differences in slopes.**

**9. Major issue.** The discussion section contains too much speculation, some of which could actually be checked with existing data. Perhaps in another paper? For this paper, please tone down the speculations. I will restate it in different terms: when reading the discussion, I did not feel awe, just confusion. One of the reviewers complained on the lack of conceptual clarity. I think it acutely applies to the discussion. Make the discussion clearer and less speculative. Another aspect of the discussion: it contains mostly abstract words, many of them nominalizations, and many key nouns are modified by adjectives. This makes reading more difficult. Try writing this section in shorter sentences, it might help. Try using less subordinated clauses.

**Our response:** Thank you. The discussion section has been revised thoroughly and restructured accordingly.

**10. Lines 554-557:** *"Our results also suggest that awe-prone individuals are more curious (120), and that awe itself can stimulate curiosity (121)."*

**Editor’s comment:** When one says "results suggest" it implies something new; but you have references, so it is old. Consider rephrasing.

**Our response:** Thank you for the helpful comment. We rephrased the line as: **“our results indicate that awe-prone individuals are more curious and that awe itself can stimulate curiosity; these results are in line with previous research (Anderson et al., 2020; Izard, 1977).”**

**11. Lines590-591:** "*We, here operationalized ‘creativity’ as the poem’s creative potential."*

**Editor’s comment:** Regarding the issue, raised by a reviewer, on whether creativity is a property of people or of results. It is a difficult issue, but a notion of 'creative potential' is not likely be understood by the audience. Here is a suggestion, by twisting the 4P approach. Results can often be called 'creative', not by attributing them creativity per se, but just by implicitly denoting them as results of a creative process. Such implicit attribution is pervasive, so we call many things 'creative' without implying that the things can create something.

**Our response:** Thank you for your valuable suggestion. We removed the afore-mentioned lines from the limitations section. Instead, we integrated the explanation into the Introduction section, incorporating the 4P model as suggested. Here is the revised passage:

**“The 4P model of creativity, a seminal theoretical framework of creativity, proposed “The word creativity is a noun naming the phenomenon in which a person communicates a new concept (which is the product). Mental activity (or mental process) is implicit in the definition and of course no one could conceive of a person living or operating in a vacuum, so the term press is also implicit. The definition begs the questions as to how new the concept must be and to whom it must be new” (Rhodes, 1961, p. 305).**  **Among these 4P approaches, i.e., person, product, process, and press, the product or physical object, plays an important role. In common perceptions, creativity is often equated with its tangible outcome — the creative product. When asked to define creativity, many would instinctively describe it in terms of the final product (Gruszka et al. 2017). Literature suggests that a product-centered operational definition is the most useful for empirical research in creativity and presumably the most important feature of this definition is its reliance on subjective criteria (Amabile, 1982).**  **Despite debates and the difficulty of precisely defining creativity of a product (Sternberg & Lubert, 1999; Beghetto, & Dow, 2004; Simonton, 2018), the most widely accepted operational definition is the “standard definition” of creativity (Runco & Jaeger, 2012), which states that for a product or idea to be deemed creative, it must be both original or novel and useful or appropriate. Additionally, surprise is also added as the third ingredient of creativity (Simonton 2012). The process aspect of the 4P model usually involves two phases of cognitive processes: the generative phase and the evaluative phase (Fink et al., 1992).**

**The present study adopts a dual focus on both the product and process aspects of creativity using the poem as the product and its evaluation process as the measure of creativity. We operationalized the ‘creativity’ of a poem as its creative potential, aiming to broaden the understanding of creativity from the creator to the creation itself. Our approach is in line with past studies that have investigated the creativity evaluation of various types of products/artefacts, such as ideas (Lloyd-Cox et al., 2022), musical compositions (Pinho et al., 2014; Zioga et al., 2020), short stories (Toivainen et al., 2021), and product concepts (Guenther et al., 2021), to name a few. This approach allows us to investigate how individuals assess the creativity of poems, recognizing the subjective nature of such evaluations and how they may be influenced by individual personality traits. In summary, we aim to uncover how variations in reader personality may subtly influence the evaluation of a poem's creativity, thereby shaping an implicit model of evaluation.”**

**Responses to the comments of Reviewer 1:**

Reviewer #1: All my comments have been answered to my satisfaction. Nevertheless, I believe that it would be in the authors' own interest to explain their definition of the term "creativity" in more detail (and to do so at the beginning of the paper). I believe that a large proportion of readers do not know in which tradition the authors use the term. To avoid losing these readers, it would be helpful to explain what the "4P model" is and how to define creativity "through the lens of the product". Honestly, I believe that the majority of readers - like me - will not understand a single word when they read this. I would also recommend that you invest a little time to correct typos. To give some examples: Line 91: "artwork..", line 273: "onpersonality", line 465: "openess", ..

**Our response:** Thank you for your valuable comments and suggestions. Following your advice, we have provided an explanation of the definition of "creativity" in the introduction, along with an elucidation of the "4P model" for readers outside the field of creativity research. We have also corrected any typographical errors that were identified. The inclusion of the 4P model in the “Introduction” is as follows:

“**The 4P model of creativity, a seminal theoretical framework of creativity, proposed “The word creativity is a noun naming the phenomenon in which a person communicates a new concept (which is the product). Mental activity (or mental process) is implicit in the definition and of course no one could conceive of a person living or operating in a vacuum, so the term press is also implicit. The definition begs the questions as to how new the concept must be and to whom it must be new” (Rhodes, 1961).**  **Among these 4P approaches, i.e., person, product, process, and press, the product or physical object, plays an important role. In common perceptions, creativity is often equated with its tangible outcome—the creative product. When asked to define creativity, many would instinctively describe it in terms of the final product (Gruszka & Tang, 2017). Literature suggests that a product-centered operational definition is the most useful for empirical research in creativity and presumably the most important feature of this definition is its reliance on subjective criteria (Amabile, 1982). Despite debates and the difficulty of precisely defining creativity of a product (Sternberg & Lubart, 1999; Plucker et al., 2004; Simonton, 2018), the most widely accepted operational definition is the “standard definition” of creativity, which states that for a product or idea to be deemed creative, it must be both original or novel and useful or appropriate. Additionally, surprise is also added as the third ingredient of creativity (Simonton, 2012). The process aspect of the 4P model usually involves two phases of cognitive processes: the generative phase and the evaluative phase (Finke et al., 1996).**

**The present study adopts a dual focus on both the product and process aspects of creativity using poem as the product and its evaluation process as the measure of creativity. We operationalized the ‘creativity’ of a poem as its creative potential, aiming to broaden the understanding of creativity from the creator to the creation itself. Our approach is in line with past studies that have investigated the creativity evaluation of various types of products/artefacts, such as ideas (Lloyd-Cox et al., 2022), musical compositions (Pinho et al., 2014); (Zioga et al., 2020), short stories (Toivainen et al., 2021), and product concepts (Guenther et al., 2021), to name a few. This approach allows us to investigate how individuals assess the creativity of poems, recognizing the subjective nature of such evaluations and how they may be influenced by individual personality traits. In summary, we aim to uncover how variations in reader personality may subtly influence the evaluation of a poem's creativity, thereby shaping an implicit model of evaluation.**

**Responses to the comments of Reviewer 2 (as mentioned in the PDF)**

Corrections, modifications, explanations, and clarifications have been incorporated as suggested throughout the manuscript. These revisions were implemented at the specified line numbers as indicated by the reviewer in the PDF: 23, 28, 47, 48, 49, 51, 63, 67, 70, 80, 81,90, 93, 97, 100, 109, 115, 124, 133, 142, 147, 151, 163, 188, 202, 205, 268, 272, 283, 288, 325, 342, 465, 480, 484, 506, 508, 535, 546, 550, 586. All changes have been highlighted in bold in the revised manuscript.

We have addressed and corrected all typographical errors and structural inconsistencies in the lines mentioned above. The following explanations correspond to the highlighted line numbers indicated by the Reviewer 2 in the PDF:

**Line 70: …***recent research (14) suggests that openness to experience is associated with a higher discernment ability to distinguish creative from uncreative responses.*

**Reviewer’s comment:** “I'm not sure what that means exactly. This assumes that there is some kind of objective measure of creative vs uncreative and those with higher openness are better able to distinguish between them somehow, but isn't creativity assessment usually on some kind of continuum?”

**Our response:** Ceh et al. (2022) investigated the relationship between rater personality and creativity in determining discernment (i.e., the ability to distinguish between creative and uncreative responses) when evaluating others’ responses. In their study, a group of novice raters evaluated a pre-selected set of responses from a creativity task, e.g., the alternate uses task, while the rater’s own creativity and personality traits were also assessed. The study found that creativity in terms of creative potential and creative achievement independently predicted rater discernment. Regarding personality traits, individuals who were more creative and more open were found to be more discerning raters, and this finding is in line with previous work (Benedek et al., 2016; Silvia, 2008). Openness is often associated with an appreciation for new and unconventional ideas (DeYoung, 2015; McCrae, 1987). The research suggests that open people do not merely rate all responses as more creative rather, they are better at identifying genuinely creative ideas, thereby demonstrating higher overall discernment (Ceh et al., 2022).

**Line 81: …** *five factors - clarity, aesthetic appeal, felt valence, arousal, and surprise*

**Reviewer’s comment: “**Why these in particular? Is that explained later?”

**Our response:** The rationale behind selecting the four personality traits and the five predictors has been explained in the Introduction section of the revised manuscript.

**“Following prior research** (Amabile, 1982 **;** Leder et al., 2012**;** Conner & Silvia, 2015**;** Acar et al., 2017)**, we selected five potential predictors: clarity, aesthetic appeal, felt valence, arousal, and surprise.”**

We subsequently provided a concise overview of these potential predictors, the personality traits, and their respective roles in assessing creativity in the main Introduction section and its subsections.

**Line 147: *“****Specifically, individuals with higher levels of openness would perceive the positive impact of felt emotions on creativity rating as less significant compared to those with lower levels of openness.”*

**Reviewer’s comment:** Don't understand this. You've just said that those with higher openness are more influenced by their emotions, but expect that to have less impact on their creativity assessments than those with lower openness? Why?

**Our response:** Thank you. To enhance clarity and better comprehension, we rephrased the line as follows:

**“Individuals with higher openness are known to be more sensitive and attuned to their feelings (Costa & McCrae, 1992), yet intense emotional engagement can sometimes inhibit higher cognitive functions in these individuals (Siegel, 2020). Neurological studies suggest that heightened emotional states can inhibit the brain’s reflective processes, affecting intellectual openness (Siegel, 2020); see also (Jarvinen & Paulus, 2017). Hence, we expected that openness would moderate the relationship between felt emotions (both valence and arousal) and creativity. Specifically, the positive impact of felt emotions on creativity ratings may be perceived as less pronounced by individuals with higher levels of openness compared to those with lower levels of openness.”**

**Line 163: *“****Our specific prediction was that the positive impact of surprise on creativity scores would be more pronounced in individuals with higher openness and intellect. These individuals may be more receptive to and intrigued by unexpected elements of a poem, leading to heightened creativity judgment in response to surprises encountered in poems.”*

**Reviewer’s comment:** Those high in openness may also be less prone to expecting something in particular to begin with and therefore less likely to experience as much surprise?

**Our response:** We modified the sentence for clarity of understanding:

Our initial prediction was (mentioned in the Introduction section: Openness/Intellect subsection)

**“We expected that individuals high in open-mindedness and intellectual curiosity would exhibit a heightened receptivity and interest in unexpected elements within poems. This inclination would lead them to prioritize surprise when assessing the creativity of poems, in contrast to those with lower levels of openness and intellect.”**

However, our results contradict this initial prediction. In the Discussion, this has been mentioned as follows:

**“Individuals with lower levels of openness were found to be more influenced by surprise in their creativity ratings of poems than their higher counterparts. Surprise is often recognized as an interruption mechanism and a short-lived emotion with an unclear positive or negative valence (Meyer et al., 1997).** **The statistically significant difference of the simple slopes for high and low open individuals indicates** **that, more open individuals, who are more motivated to learn,** **might be less influenced by the surprise in the contents of the poems compared to their lower counterpart while judging poetic creativity.The transient and ambiguous nature of surprise might disrupt their affective states, leading to a reduced impact of surprise on their creativity judgment.** **In contrast, less open individuals perceived surprise as a more significant factor in their evaluation of poetic creativity than their higher counterparts, contradicting our initial prediction. It is noteworthy that the interaction does not indicate that high openness readers were less surprised by the poems compared to low openness readers. Rather it suggests that their judgments of a poem’s creativity were less influenced by the surprise element of the poem compared to those with lower openness.** **Furthermore, our focus was not on whether individuals with higher openness rated surprise more highly on average than those with lower openness. Instead, we focused on the differential level of surprise ratings for high and low openness. Our objective was to investigate whether there was a difference in how surprise was prioritized as a predictor of creativity judgment between the two levels of openness.”**

**Line 202:** (regarding initial selection of poems)

**Reviewer’s comment:** “I'm assuming at random somehow - how was the selection done?”

**Our response:** We appreciate the reviewer's comment. The initial selection process involved randomly choosing 108 English poems from a variety of online resources, including reputable poetry sites such as Poetry.org (http://www.poetry.org/), the Poetry Foundation (https://www.poetryfoundation.org/), and the Academy of American Poets (<https://poets.org/>). To ensure diversity in our sample, we employed specific criteria such as selecting poems from various genres, themes, and historical periods to capture a broad spectrum of creative expression. We believe this approach provided a comprehensive representation of English poetry and enriched the scope of our study.

**Line 205:** (regarding “surprise” ratings of the initially selected poems, by M.D.)

**Reviewer’s comment:** “Given that surprise kind of depends on the expectations the person reading it has and what constitutes a violation of that expectation for them, possibly it would make more sense to have the participant themself rate the 'surprisingness' of the poems? Otherwise, a poem that MD may have found not very surprising might have been quite surprising to a participant.”

**Our response:** We appreciate the reviewer's thoughtful comment regarding the rating of surprise levels in our stimuli poems. The decision to have M.D., a Professor of English and Creative Writing with expertise in the domain, rate the surprise level was based on the need for an informed and nuanced evaluation. As the reviewer rightly pointed out, the perception of surprise in poetry may vary depending on individual expectations and interpretations. However, we believe that M.D.'s expertise provided valuable insights into the nuanced elements of poetic expression and literary techniques that contribute to the experience of surprise. While participant ratings could certainly offer another perspective, we aimed to establish a baseline assessment of surprise levels that accounted for expert knowledge of literary devices, thematic content, and poetic conventions. This approach allowed us to ensure consistency and reliability in the identification of poems with low and high surprise ratings. However, we will consider this in our future studies to enrich the comprehensiveness of our experimental design.

**Line 268:** (regarding felt valence)

**Reviewer’s comment:** “How was this measured using low to high? Was there a positive and a negative valence question?”

**Our response:** In our study, participants were queried about felt valence by asking, *"How positive (higher scores) or negative (lower scores) did you feel when you read the poem?"* For arousal ratings, they were prompted with, *"How stimulating (higher scores) or relaxing (lower scores) did you feel when you read the poem?"* Responses were recorded on a 7-point scale, where 1 indicated "Extremely low" and 7 denoted "Extremely high."

**Line 272:** (regarding the scales of personality traits questionnaires)

**Reviewer’s comment:** “This could do with a bit more detail - how long were these scales?” (on personality traits)

**Our response:** The Openness/Intellect scale comprises 20 items (DeYoung et al., 2007), awe-proneness consists of 6 items (Shiota et al., 2006), and curiosity comprises 10 items (Litman & Spielberger, 2003). All personality questionnaires utilized a 7-point scale, with 1 representing “disagree strongly” and 7 representing “agree strongly”.

**Line 283:** (regarding hierarchical model)

**Reviewer’s comment:** “Why? My understanding (not a regression expert though) is that hierarchical regression models are usually used when there is a theoretical 'order of importance' assumed of the predictor variables. As this seems more exploratory, it would be good to understand why this approach was chosen and in what order the variables were entered into the model.”

**Our response:** We appreciate the reviewer's comment. While hierarchical models are typically employed when there is a theoretical "order of importance" assumed for predictor variables, our rationale for using this approach stems from the exploratory nature of our study and the desire to systematically assess the impact of each predictor on the outcome variable. Notably, the order in which our predictor variables were introduced into the model was based on their correlations with the dependent variable. This systematic inclusion, based on correlation strength, aimed to identify and assess the unique contribution of each predictor variable while controlling for the influence of other variables already included in the model. This approach allowed us to explore potential relationships between the independent and dependent variables in a structured manner, enabling us to observe how the addition of each variable incrementally contributes to the predictive power of the model. While our study may not have been driven by a strict theoretical hierarchy of predictor variables, the hierarchical modelling approach provided a systematic framework for examining these relationships in an exploratory context.

**Line 288:** (regarding multicollinearity)

**Reviewer’s comment:** “Are these IVs just the creativity assessment variables or also the personality variables? I'd be surprised if openness and awe-proneness weren't correlated. “

**AND**

**Line 342:** (regarding multicollinearity)

**Reviewer’s comment:** “The above test for multicollinearity seems to contradict this - as I suspected, there's a lot of correlation between the individual personality variables, hence multicollinearity... I'm not familiar with VIF however so possibly this can explain why the correlations between many of the variables isn't problematic? However, on a theoretical level, I have concerns about how different openness to experience, awe-proneness and curiosity in particular are as constructs.”

**Our response:** Thank you for your comment. Although a correlation greater than 0.80 or 0.90 is indicative of multicollinearity (Cohen et al., 2002), however, a more robust test for multicollinearity is the variance inflation factor (VIF). The VIF is a measure of multicollinearity in a regression model indicating whether there is a strong correlation between multiple independent variables in the regression model. The literature indicates that VIF values greater than 10 are cause for concern (Bowerman & O’connell, 1990; Myers & Myers, 1990; see also Field, 2013). Additionally, the tolerance statistic, which is the reciprocal of VIF, provides further insight: a tolerance below 0.1 indicates a serious problem, while below 0.2 indicates a potential problem (Bowerman & O’connell, 1990).

In our multiple regression model, the 5 predictor variables were the IVs, and creativity was the DV. Personality variables acted as moderators, they were not included in the multiple regression model predicting creativity. The maximum VIF for the predictors was 2.59, with a tolerance of 0.39. VIF<3 indicates no evidence of significant multicollinearity impacting the multiple regression model predicting creativity.

The bivariate correlation (Table2) suggests that openness and awe-proneness are correlated (*r* = 0.47, *p*<.01). Within personality measures, all were significantly correlated with each other, and the strongest correlation was observed between curiosity and awe-proneness (*r* = 0.57, *p*<.01).

**Line 506:** (regarding 3-criterion definition of creativity)

**Reviewer’s comment:** “Reiterate what that is for those who don't know. “

**Our response:** Thank you for the suggestion. It has been explained in the Discussion section as follows:

**“It is worth mentioning that to reach a consensus on how best to define the creativity phenomenon, the 3-criterion definition of creativity (Simonton, 2012) is proposed which is based on the three criteria used by the United States Patent Office to evaluate applications for patent protection. This modified definition uses the criteria of novelty or originality, utility or usefulness, and surprise to judge creativity of a product or idea.”**

**Line 535:**

**Reviewer’s comment:** Are curiosity and awe really different construct?

Ans. Thank you for your query. Awe and curiosity are distinct constructs amongst the emotional states of human flourishing, e.g., awe, wonder, inspiration, and curiosity, and have been studied as separate constructs in earlier research (Shiota et al., 2017; Valdesolo, 2021; Loewenstein, 1994; Kidd & Hayden, 2015; Reio Jr et al., 2006; Anderson et al., 2020).

Awe is typically triggered by something vast (either literally or metaphorically) and initiates processes of accommodation, in which existing mental schemas are revised to make sense of the awe-inspiring stimuli (Gottlieb et al., 2018). According to Keltner and Haidt (2003), awe is a destabilizing emotion: It is elicited by something *vast*, either physically or metaphorically.

Curiosity has been defined as a “drive to know” (Berlyne, 1954, p.187). Specifically, epistemic curiosity is regarded as a means to support learning in educational contexts (Von Stumm et al., 2011), and has been found to promote the exploration of new knowledge (Berlyne, 1954, 1960 ; Litman et al., 2005) and to enhance memory for new information (Gruber et al., 2014; Marvin & Shohamy, 2016).

However, research suggests that dispositional awe was positively related to people's self-rated curiosity; people who often feel awe are also more curious (Anderson et al., 2020); Our result also supports this notion.

**Line 546:** *“In contrast, the influence of surprise on creativity judgment was more pronounced in those individuals with lower levels of curiosity, indicating that surprise, as a short-lived emotion and interruption mechanism (55), may interrupt the information-seeking tendencies of highly curious readers during their evaluation of poetic creativity.”*

**Reviewer’s comment: “**I would expect that if a highly curious person encountered something surprising, their curiosity would make them more prone toward information-seeking tendencies, not less.”

**Our response:** Thank you, we appreciate your comment. Both surprise and curiosity are epistemic emotions, closely related to the knowledge-generating qualities of cognitive tasks and activities (Vogl et al., 2020; Berlyne, 1954 ; Ekman, 1992, 1999). Surprise is elicited by unexpected or schema-discrepant events (Berlyne, 1954,1960 ; Meyer et al., 1997; Noordewier & Breugelmans, 2013 ; Noordewier et al., 2016 ; Stiensmeier-Pelster et al., 1995). whereas epistemic curiosity is driven by a desire to acquire new knowledge (Berlyne, 1954,1960; Litman et al., 2005), supports learning in educational contexts (Von Stumm et al., 2011), and enhances memory for new information (Gruber et al., 2014; Marvin & Shohamy, 2016).

Our result shows individuals with higher levels of epistemic curiosity placed less emphasis on surprise while assessing poetic creativity compared to those with lower levels of curiosity. This may indicate that the presence of surprise in our selected poems did not effectively complement their knowledge-seeking nature. For these individuals, surprises might have felt more like interruptions than cognitive stimulation, hindering rather than aiding their exploratory and curious mindset.

Of note, our focus was not on whether individuals with higher curiosity rated surprise more highly on average than those with lower curiosity. Instead, we focused on the differential level of surprise ratings for high and low curiosity traits of the readers. Our objective was to investigate whether there was a difference in how surprise was prioritized as a predictor of creativity judgment between the two levels of curiosity.

**Line 550:**

**Reviewer’s comment:** “Are openness and curiosity justifiable as separate constructs?”

**Our response:** We appreciate your comment. Openness and curiosity are considered distinct personality traits. Curiosity is a key driver of human motivation (Berlyne, 1960, 1973; Izard, 1977) and is considered one of the fundamental strengths and traits studied by psychologists (Peterson & Seligman, 2004). Its significance is deeply rooted in evolutionary benefits: “The importance of curiosity to thought and memory are so extensive that the absence…would jeopardize intellectual development no less than the destruction of brain tissue…there is no human competence which can be achieved in the absence of a sustaining interest” (Tomkins, 1962, p. 347). Curiosity shares similarities with intrinsic motivation, flow, novelty-seeking, interest, and openness to experience; however, research has highlighted its unique characteristics, such as an inclination toward exploring new things and maintaining an open and receptive attitude toward the subject of interest (Kashdan et al., 2009), information seeking and positive framing (Harrison et al., 2011), socialization (Reio Jr & Wiswell, 2000), decision making (Maner & Gerend, 2007), creative performance and positive affect (Chang & Shih, 2019); see also (Wagstaff et al., 2021). Curious individuals tend to exhibit heightened attention to activities, process information more deeply, have better memory retention, and demonstrate increased persistence in tasks until objectives are achieved (Silvia, 2006). Furthermore, curiosity serves an immediate function in facilitating learning, exploration, and engagement with stimuli that captures attentional resources (Loewenstein, 1994).

Openness to experience emerges as a pivotal trait (Kashdan & Silvia, 2009). Prominent personality models, such as the Five Factor Model (Costa & McCrae, 1999), Big Five Model (Soto & John, 2017), HEXACO (Lee & Ashton, 2004), and Big Five Aspects Model (DeYoung et al., 2007) investigated the varying degrees of emphasis placed on curiosity as a component of openness across different frameworks. For example, in the Five Factor Model (Costa & McCrae, 1999), curiosity is captured most fully by the lower-level *Ideas* facet, in the Big Five Aspects Model, curiosity is tacit and not directly captured by either aspect, i.e., Openness and Intellect (DeYoung et al., 2007), in the HEXACO Model (Lee & Ashton, 2004), curiosity is captured by the lower-level Inquisitiveness facet, and in the Big Five Model (Soto & John, 2017), curiosity is captured in the lower-level Intellectual curiosity facet.(see also Silvia & Christensen, 2020)

Of note, our focus was not on whether individuals with higher personality traits rated the predictors more highly on average than those with lower traits. Instead, we focused on the differential level of ratings of the predictors for high and low traits of the readers. Our objective was to investigate whether there was a difference in how the predictors was prioritized between the two levels of personality traits while predicting creativity judgment of a poem. This has been incorporated into the Discussion section of the revised manuscript.

**Line 586:** *“Finally, we acknowledge that the operationalization of ‘creativity’ is different from the more traditional view of creativity as a characteristic of individuals.”*

**Reviewer’s comment:** “Is it really more 'traditional'? As you go on to say, it's one lens through which it has been examined - product-based creativity is well researched so arguably just as 'traditional'. Perhaps the 4P model should have been discussed in the introduction, along with a stronger explanation/definition of how creativity was being used in this study.”

**Our response:** Thank you for your valuable comment and feedback. The 4P model and the operational definition of creativity, has been removed from the Limitations section and integrated into the Introduction section as follows:

**“The 4P model of creativity, a seminal theoretical framework of creativity, proposed “The word creativity is a noun naming the phenomenon in which a person communicates a new concept (which is the product). Mental activity (or mental process) is implicit in the definition and of course no one could conceive of a person living or operating in a vacuum, so the term press is also implicit. The definition begs the questions as to how new the concept must be and to whom it must be new” (Rhodes, 1961).**  **Among these 4P approaches, i.e., person, product, process, and press, the product or physical object, plays an important role. In common perceptions, creativity is often equated with its tangible outcome—the creative product. When asked to define creativity, many would instinctively describe it in terms of the final product (Gruszka & Tang, 2017). Literature suggests that a product-centered operational definition is the most useful for empirical research in creativity and presumably the most important feature of this definition is its reliance on subjective criteria (Amabile, 1982). Despite debates and the difficulty of precisely defining creativity of a product (Sternberg & Lubart, 1999; Plucker et al., 2004; Simonton, 2018), the most widely accepted operational definition is the “standard definition” of creativity, which states that for a product or idea to be deemed creative, it must be both original or novel and useful or appropriate. Additionally, surprise is also added as the third ingredient of creativity (Simonton, 2012). The process aspect of the 4P model usually involves two phases of cognitive processes: the generative phase and the evaluative phase (Finke et al., 1996).**

**The present study adopts a dual focus on both the product and process aspects of creativity using poem as the product and its evaluation process as the measure of creativity. We operationalized the ‘creativity’ of a poem as its creative potential, aiming to broaden the understanding of creativity from the creator to the creation itself. Our approach is in line with past studies that have investigated the creativity evaluation of various types of products/artefacts, such as ideas (Lloyd-Cox et al., 2022), musical compositions (Pinho et al., 2014; Zioga et al., 2020), short stories (Toivainen et al., 2021), and product concepts (Guenther et al., 2021), to name a few. This approach allows us to investigate how individuals assess the creativity of poems, recognizing the subjective nature of such evaluations and how they may be influenced by individual personality traits. In summary, we aim to uncover how variations in reader personality may subtly influence the evaluation of a poem's creativity, thereby shaping an implicit model of evaluation.”**

**Reviewer’s comment:** Lexical diversity/divergent semantic integration measures - was this just to ensure that the stimuli set was diverse?

**Our response:** We included measures of lexical diversity (LD) and divergent semantic integration (DSI) to demonstrate the richness and variability in the selected poems. By assessing LD and DSI, we aimed to ensure that the stimuli set encompassed a wide range of lexical and semantic features, thereby enhancing the representativeness and comprehensiveness of our analysis.

**References:**

Acar, S., Burnett, C., & Cabra, J. F. (2017). Ingredients of creativity: Originality and more. *Creativity Research Journal*, *29*(2), 133–144. https://doi.org/10.1080/10400419.2017.1302776

Amabile, T. M. (1982). Social psychology of creativity: A consensual assessment technique. *Journal of Personality and Social Psychology*, *43*, 997–1013. https://doi.org/10.1037/0022-3514.43.5.997

Anderson, C. L., Dixson, D. D., Monroy, M., & Keltner, D. (2020). Are awe-prone people more curious? The relationship between dispositional awe, curiosity, and academic outcomes. *Journal of Personality*, *88*(4), 762–779. https://doi.org/https://doi.org/10.1111/jopy.12524

Benedek, M., Nordtvedt, N., Jauk, E., Koschmieder, C., Pretsch, J., Krammer, G., & Neubauer, A. C. (2016). Assessment of creativity evaluation skills: A psychometric investigation in prospective teachers. *Thinking Skills and Creativity*, *21*, 75–84. https://doi.org/https://psycnet.apa.org/doi/10.1037/1931-3896.2.3.139

Berlyne, D. E. (1954). A theory of human curiosity. *British Journal of Psychology*, *45*(3), 180.

Berlyne, D. E. (1960). Conflict, arousal, and curiosity. In *Conflict, arousal, and curiosity.* McGraw-Hill Book Company. https://doi.org/10.1037/11164-000

Berlyne, D. E. (1973). Aesthetics and psychobiology. *Journal of Aesthetics and Art Criticism*, *31*(4). https://doi.org/https://doi.org/10.2307/429334

Bowerman, B. L., & O’connell, R. T. (1990). Linear statistical models: An applied approach. In *(No Title)* (2nd ed.). Duxbury Press. https://lccn.loc.gov/89016367

Ceh, S. M., Edelmann, C., Hofer, G., & Benedek, M. (2022). Assessing raters: What factors predict discernment in novice creativity raters? *The Journal of Creative Behavior*, *56*(1), 41–54. https://doi.org/https://doi.org/10.1002/jocb.515

Chang, Y.-Y., & Shih, H.-Y. (2019). Work curiosity: A new lens for understanding employee creativity. *Human Resource Management Review*, *29*(4), 100672. https://doi.org/https://doi.org/10.1016/j.hrmr.2018.10.005

Chatterjee, A., & Vartanian, O. (2014). Neuroaesthetics. *Trends in Cognitive Sciences*, *18*(7), 370–375. https://doi.org/https://doi.org/10.1016/j.tics.2014.03.003

Cohen, J., Cohen, P., West, S. G., & Aiken, L. S. (2002). *Applied multiple regression/correlation analysis for the behavioral sciences*. Routledge. https://doi.org/https://doi.org/10.4324/9780203774441

Conner, T. S., & Silvia, P. J. (2015). Creative days: a daily diary study of emotion, personality, and everyday creativity. *Psychology of Aesthetics, Creativity, and the Arts*, *9*(4), 463. https://doi.org/https://psycnet.apa.org/doi/10.1037/aca0000022

Costa, P. T., & McCrae, R. R. (1992). Four ways five factors are basic. *Personality and Individual Differences*, *13*(6), 653–665. https://doi.org/https://doi.org/10.1016/0191-8869(92)90236-I

Costa, P. T., & McCrae, R. R. (1999). A five-factor theory of personality. *The Five-Factor Model of Personality: Theoretical Perspectives*, *2*, 51–87.

Cupchik, G. C., & László, J. (1992). *Emerging visions of the aesthetic process: In psychology, semiology, and philosophy*. Cambridge University Press.

DeYoung, C. G. (2015). Openness/intellect: A dimension of personality reflecting cognitive exploration. In *APA handbook of personality and social psychology, Volume 4: Personality processes and individual differences.* (pp. 369–399). American Psychological Association. https://doi.org/10.1037/14343-017

DeYoung, C. G., Quilty, L. C., & Peterson, J. B. (2007). Between facets and domains: 10 aspects of the Big Five. *Journal of Personality and Social Psychology*, *93*(5), 880–896. https://doi.org/https://psycnet.apa.org/doi/10.1037/0022-3514.93.5.880

Ekman, P. (1992). An argument for basic emotions. *Cognition & Emotion*, *6*(3–4), 169–200. https://doi.org/https://doi.org/10.1080/02699939208411068

Ekman, P. (1999). Basic emotions. In *Handbook of cognition and emotion* (Vol. 98, Issues 45–60). John Wiley & Sons, 2000.

Enders, C. K., & Tofighi, D. (2007). Centering predictor variables in cross-sectional multilevel models: a new look at an old issue. *Psychological Methods*, *12*(2), 121–138. https://doi.org/https://psycnet.apa.org/doi/10.1037/1082-989X.12.2.121

Field, A. (2013). *Discovering statistics using IBM SPSS statistics* (4th ed.). sage.

Finke, R. A., Ward, T. B., & Smith, S. M. (1996). *Creative cognition: Theory, research, and applications*. MIT press.

Gordon, P. C., & Holyoak, K. J. (1983). Implicit learning and generalization of the" mere exposure" effect. *Journal of Personality and Social Psychology*, *45*(3), 492–500. https://doi.org/https://psycnet.apa.org/doi/10.1037/0022-3514.45.3.492

Gottlieb, S., Keltner, D., & Lombrozo, T. (2018). Awe as a Scientific Emotion. *Cognitive Science*, *42*(6), 2081–2094. https://doi.org/https://doi.org/10.1111/cogs.12648

Gruber, M. J., Gelman, B. D., & Ranganath, C. (2014). States of curiosity modulate hippocampus-dependent learning via the dopaminergic circuit. *Neuron*, *84*(2), 486–496. https://doi.org/https://doi.org/10.1016/j.neuron.2014.08.060

Gruszka, A., & Tang, M. (2017). The 4P’s creativity model and its application in different fields. In *Handbook of the management of creativity and innovation: Theory and practice* (pp. 51–71). World Scientific Publishing Company.

Guenther, A., Eisenbart, B., & Dong, A. (2021). Creativity and successful product concept selection for innovation. *International Journal of Design Creativity and Innovation*, *9*(1), 3–19. https://doi.org/https://doi.org/10.1080/21650349.2020.1858970

Harrison, S. H., Sluss, D. M., & Ashforth, B. E. (2011). Curiosity adapted the cat: The role of trait curiosity in newcomer adaptation. *Journal of Applied Psychology*, *96*(1), 211–220. https://doi.org/https://psycnet.apa.org/doi/10.1037/a0021647

Hartley, J., & Homa, D. (1981). Abstraction of stylistic concepts. *Journal of Experimental Psychology: Human Learning and Memory*, *7*(1), 33–46. https://doi.org/https://psycnet.apa.org/doi/10.1037/0278-7393.7.1.33

Izard, C. E. (1977). *Human Emotions*. New York: Plenum.

Jacobs, A. M. (2015). Towards a neurocognitive poetics model of literary reading. In *Cognitive neuroscience of natural language use* (pp. 135–159). Cambridge University Press.

Jarvinen, M. J., & Paulus, T. B. (2017). Attachment and cognitive openness: Emotional underpinnings of intellectual humility. *The Journal of Positive Psychology*, *12*(1), 74–86. https://doi.org/10.1080/17439760.2016.1167944

Kashdan, T. B., Gallagher, M. W., Silvia, P. J., Winterstein, B. P., Breen, W. E., Terhar, D., & Steger, M. F. (2009). The curiosity and exploration inventory-II: Development, factor structure, and psychometrics. *Journal of Research in Personality*, *43*(6), 987–998. https://doi.org/https://doi.org/10.1016/j.jrp.2009.04.011

Kashdan, T. B., & Silvia, P. J. (2009). Curiosity and interest: The benefits of thriving on novelty and challenge. In *Oxford handbook of positive psychology* (Vol. 2, pp. 367–374). Oxford University Press.

Keltner, D., & Haidt, J. (2003). Approaching awe, a moral, spiritual, and aesthetic emotion. *Cognition and Emotion*, *17*(2), 297–314. https://doi.org/10.1080/02699930302297

Kidd, C., & Hayden, B. Y. (2015). The psychology and neuroscience of curiosity. *Neuron*, *88*(3), 449–460. https://doi.org/http://dx.doi.org/10.1016/j.neuron.2015.09.010

Lachapelle, R., Murray, D., & Neim, S. (2003). Aesthetic Understanding as Informed Experience: The Role of Knowledge in Our Art Viewing Experiences. *Journal of Aesthetic Education*, *37*(3), 78–98. https://doi.org/10.2307/3527305

Leder, H., Gerger, G., Dressler, S. G., & Schabmann, A. (2012). How art is appreciated. *Psychology of Aesthetics, Creativity, and the Arts*, *6*(1), 2. https://doi.org/https://psycnet.apa.org/doi/10.1037/a0026396

Leder, H., Belke, B., Oeberst, A., & Augustin, D. (2004). A model of aesthetic appreciation and aesthetic judgments. *British Journal of Psychology*, *95*(4), 489–508. https://doi.org/https://doi.org/10.1348/0007126042369811

Lee, K., & Ashton, M. C. (2004). Psychometric properties of the HEXACO personality inventory. *Multivariate Behavioral Research*, *39*(2), 329–358. https://doi.org/https://doi.org/10.1207/s15327906mbr3902_8

Litman, J. A., & Spielberger, C. D. (2003). Measuring epistemic curiosity and its diversive and specific components. *Journal of Personality Assessment*, *80*(1), 75–86. https://doi.org/10.1207/S15327752JPA8001_16

Litman, J., Hutchins, T., & Russon, R. (2005). Epistemic curiosity, feeling-of-knowing, and exploratory behaviour. *Cognition and Emotion*, *19*(4), 559–582. https://doi.org/10.1080/02699930441000427

Lloyd-Cox, J., Pickering, A., & Bhattacharya, J. (2022). Evaluating creativity: How idea context and rater personality affect considerations of novelty and usefulness. *Creativity Research Journal*, *34*(4), 373–390. https://doi.org/10.1080/10400419.2022.2125721

Loewenstein, G. (1994). The psychology of curiosity: A review and reinterpretation. *Psychological Bulletin*, *116*(1), 75–98. https://doi.org/https://psycnet.apa.org/doi/10.1037/0033-2909.116.1.75

Maner, J. K., & Gerend, M. A. (2007). Motivationally selective risk judgments: Do fear and curiosity boost the boons or the banes? *Organizational Behavior and Human Decision Processes*, *103*(2), 256–267. https://doi.org/https://doi.org/10.1016/j.obhdp.2006.08.002

Marvin, C. B., & Shohamy, D. (2016). Curiosity and reward: Valence predicts choice and information prediction errors enhance learning. *Journal of Experimental Psychology: General*, *145*(3), 266–272. https://doi.org/https://psycnet.apa.org/doi/10.1037/xge0000140

McCrae, R. R. (1987). Creativity, divergent thinking, and openness to experience. *Journal of Personality and Social Psychology*, *52*, 1258–1265. https://doi.org/10.1037/0022-3514.52.6.1258

Menninghaus, W., Wagner, V., Wassiliwizky, E., Schindler, I., Hanich, J., Jacobsen, T., & Koelsch, S. (2019). What are aesthetic emotions? *Psychological Review*, *126*(2), 171–195. https://doi.org/10.1037/rev0000135

Meyer, W. U., Reisenzein, R., & Schützwohl, A. (1997). Toward a process analysis of emotions: The case of surprise. *Motivation and Emotion*, *21*(3), 251–274. https://doi.org/https://doi.org/10.1023/A:1024422330338

Meyer, W.-U., Reisenzein, R., & Schützwohl, A. (1997). Toward a process analysis of emotions: The case of surprise. *Motivation and Emotion*, *21*(3), 251–274. https://doi.org/10.1023/A:1024422330338

Myers, R. H., & Myers, R. H. (1990). *Classical and modern regression with applications* (Vol. 2). Duxbury press Belmont, CA.

Noordewier, M. K., & Breugelmans, S. M. (2013). On the valence of surprise. *Cognition and Emotion*, *27*(7), 1326–1334. https://doi.org/10.1080/02699931.2013.777660

Noordewier, M. K., Topolinski, S., & Van Dijk, E. (2016). The Temporal Dynamics of Surprise. *Social and Personality Psychology Compass*, *10*(3), 136–149. https://doi.org/https://doi.org/10.1111/spc3.12242

Peterson, C., & Seligman, M. E. P. (2004). *Character strengths and virtues: A handbook and classification* (Vol. 1). Oxford University Press.

Pinho, A. L., de Manzano, Ö., Fransson, P., Eriksson, H., & Ullén, F. (2014). Connecting to create: expertise in musical improvisation is associated with increased functional connectivity between premotor and prefrontal areas. *Journal of Neuroscience*, *34*(18), 6156–6163. https://doi.org/https://doi.org/10.1523/JNEUROSCI.4769-13.2014

Plucker, J. A., Beghetto, R. A., & Dow, G. T. (2004). Why isn’t creativity more important to educational psychologists? Potentials, pitfalls, and future directions in creativity research. *Educational Psychologist*, *39*(2), 83–96. https://doi.org/https://doi.org/10.1207/s15326985ep3902_1

Reio Jr, T. G., Petrosko, J. M., Wiswell, A. K., & Thongsukmag, J. (2006). The measurement and conceptualization of curiosity. *The Journal of Genetic Psychology*, *167*(2), 117–135. https://doi.org/https://doi.org/10.3200/GNTP.167.2.117-135

Reio Jr, T. G., & Wiswell, A. (2000). Field investigation of the relationship among adult curiosity, workplace learning, and job performance. *Human Resource Development Quarterly*, *11*(1), 5–30.

Rhodes, M. (1961). An analysis of creativity. *The Phi Delta Kappan*, *42*(7), 305–310. https://doi.org/https://www.jstor.org/stable/20342603

Shiota, M. N., Keltner, D., & John, O. P. (2006). Positive emotion dispositions differentially associated with Big Five personality and attachment style. *The Journal of Positive Psychology*, *1*(2), 61–71. https://doi.org/https://doi.org/10.1080/17439760500510833

Shiota, M. N., Thrash, T. M., Danvers, A., & Dombrowski, J. T. (2017). Transcending the self: Awe, elevation, and inspiration. In *Handbook of Positive Emotions*. Guilford Publications.

Siegel, D. J. (2020). The developing mind: How relationships and the brain interact to shape who we are (2. utg.). In *New York: Guilford Publications*. Guilford Publications.

Silvia, P. J. (2006). *Exploring the psychology of interest*. oxford university Press.

Silvia, P. J. (2008). Discernment and creativity: How well can people identify their most creative ideas? *Psychology of Aesthetics, Creativity, and the Arts*, *2*(3), 139–146. https://doi.org/https://psycnet.apa.org/doi/10.1037/1931-3896.2.3.139

Silvia, P. J. (2010). Confusion and interest: The role of knowledge emotions in aesthetic experience. *Psychology of Aesthetics, Creativity, and the Arts*, *4*(2), 75–80. https://doi.org/10.1037/a0017081

Silvia, P. J., & Christensen, A. P. (2020). Looking up at the curious personality: individual differences in curiosity and openness to experience. *Current Opinion in Behavioral Sciences*, *35*, 1–6. https://doi.org/https://doi.org/10.1016/j.cobeha.2020.05.013

Simonton, D. K. (2012). Taking the U.S. patent office criteria seriously: A quantitative three-criterion creativity definition and its implications. *Creativity Research Journal*, *24*(2–3), 97–106. https://doi.org/10.1080/10400419.2012.676974

Simonton, D. K. (2018). Defining creativity: Don’t we also need to define what is not creative? *The Journal of Creative Behavior*, *52*(1), 80–90. https://doi.org/https://doi.org/10.1002/jocb.137

Soto, C. J., & John, O. P. (2017). The next Big Five Inventory (BFI-2): Developing and assessing a hierarchical model with 15 facets to enhance bandwidth, fidelity, and predictive power. *Journal of Personality and Social Psychology*, *113*(1), 117. https://doi.org/https://psycnet.apa.org/doi/10.1037/pspp0000096

Sternberg, R. J., & Lubart, T. I. (1999). The concept of creativity: Prospects and paradigms. *Handbook of Creativity*, *1*(3–15). https://doi.org/https://doi.org/10.1017/CBO9780511807916.003

Stiensmeier-Pelster, J., Martini, A., & Reisenzein, R. (1995). The role of surprise in the attribution process. *Cognition & Emotion*, *9*(1), 5–31. https://doi.org/https://doi.org/10.1080/02699939508408963

Toivainen, T., Madrid‐Valero, J. J., Chapman, R., McMillan, A., Oliver, B. R., & Kovas, Y. (2021). Creative expressiveness in childhood writing predicts educational achievement beyond motivation and intelligence: A longitudinal, genetically informed study. *British Journal of Educational Psychology*, *91*(4), 1395–1413. https://doi.org/https://doi.org/10.1111/bjep.12423

Tomkins, S. (1962). *Affect imagery consciousness: Volume I: The positive affects*. Springer publishing company.

Valdesolo, P. (2021). Awe, Approached. In *The Oxford Handbook of the Positive Humanities* (pp. 233–242). Oxford University Press.

Vogl, E., Pekrun, R., Murayama, K., & Loderer, K. (2020). Surprised–curious–confused: Epistemic emotions and knowledge exploration. *Emotion*, *20*(4), 625–641. https://doi.org/https://psycnet.apa.org/doi/10.1037/emo0000578

Von Stumm, S., Hell, B., & Chamorro-Premuzic, T. (2011). The hungry mind: Intellectual curiosity is the third pillar of academic performance. *Perspectives on Psychological Science*, *6*(6), 574–588. https://doi.org/https://doi.org/10.1177/1745691611421204

Wagstaff, M. F., Flores, G. L., Ahmed, R., & Villanueva, S. (2021). Measures of curiosity: A literature review. *Human Resource Development Quarterly*, *32*(3), 363–389. https://doi.org/https://doi.org/10.1002/hrdq.21417

Zioga, I., Harrison, P. M. C., Pearce, M. T., Bhattacharya, J., & Di Bernardi Luft, C. (2020). From learning to creativity: Identifying the behavioural and neural correlates of learning to predict human judgements of musical creativity. *NeuroImage*, *206*, 116311. https://doi.org/https://doi.org/10.1016/j.neuroimage.2019.116311
